# Pan-African review of cultural uses of carnivores

**DOI:** 10.1371/journal.pone.0315903

**Published:** 2025-03-25

**Authors:** Vivienne L. Williams, Marine Drouilly, Peter G. R. Coals, Gareth M. Whittington-Jones

**Affiliations:** 1 School of Animal, Plant and Environmental Sciences, University of the Witwatersrand, Johannesburg, South Africa; 2 Panthera, New York, NY, United States of America; 3 Institute for Communities and Wildlife in Africa, University of Cape Town, Cape Town, South Africa; 4 Centre for Social Science Research, University of Cape Town, Cape Town, South Africa; University of Bucharest, ROMANIA

## Abstract

The consumptive use of fauna, encompassing the extraction of skins and derivatives, undermines vulnerable species’ resilience to persistent offtake. Evidence of pervasive, Africa-wide hunting and trafficking of wildlife underscores the need to understand the drivers and extent of this utilisation and exploitation. Here, we investigated evidence for the cultural use of 33 African carnivore species (Felidae, Viverridae, Nandiniidae, terrestrial Mustelidae) across Africa, a hitherto under-explored consumptive use threat, by conducting a systematic mixed-methods review and analysis of incidence records from nearly 600 published accounts and 555 YouTube videos. Aims were to: (i) characterise the main types of documented cultural uses behind the extraction and trade of selected carnivore taxa and examine the Africa-wide occurrence and extent of these practices; (ii) identify regional and national nexuses of African trade and trafficking; and (iii) explore factors that may perpetuate utilisation of certain species and products. Results for 48 African countries show that traditional use is widespread, including for purposes like attire, zootherapy and bushmeat. The culturally endowed legacy of diverse traditions suggests that these mostly under-reported and unquantified customary practices exist on a spectrum of cultural importance, impact, and extirpation risk for species population decline. Most incidence records were of spotted carnivore skins worn by traditional leaders, healers and participants in thousands of annual cultural events. In particular, leopards serve as prominent symbols of power and are mostly sought after by higher-ranking individuals. Lions are widely used in the attire of royalty, healing practices, and are periodically killed due to human-wildlife conflict with their parts sometimes subsequently removed and used. While most incidence-based records linked larger felids to traditional use, the reporting and impact on smaller spotted carnivores should not be overlooked. Smaller species also hold intrinsic cultural value, including skins for regalia and serving as substitutes for declining larger spotted felids.

## Introduction

The consumptive use of wildlife – encompassing the extraction of skins, meat and other products – poses persistent threats to species resilience through unsustainable offtake driven by hunting, poaching, use, trade and trafficking across domestic, commercial and transnational supply chains, with adverse implications for species conservation. These pernicious practices exist on a spectrum of human importance, complexity, organisation (from opportunistic to coordinated acquisition), legality, impact, and sustainability – not only jeopardising species’ long-term persistence in their natural habitats but also putting them at varying degrees of risk for extirpation.

Driven partially by syndicates, economic inequality, community dynamics, and pressures on marginalised communities, the scale of poaching and illegal wildlife trade (IWT) has grown globally [1–12], (although assertions of illegality may be contested by stakeholders in some contexts [13]). IWT is further enabled by inconsistent law enforcement and governance in some source regions. While regional poverty-related socio-economic factors are recognised as major impetuses to use and exploit wildlife in Africa and supply international markets [2,5,14,15], the connection between the types of local consumer-bases across domestic markets for these products is often not explicitly recognised in the research discourse. Although some literature refers to the uses and ‘cultural value’ of African wildlife, the role of traditional uses in species’ risk assessments may be under-considered [16]. This raises the question: which species are being utilized, for what purposes, and in which intra-continental domestic markets for African wildlife products, thereby acting as potential drivers of trade and population decline?

Large carnivores are particularly vulnerable to human-driven population declines, such as inter- and intra-continental trade, which is often incentivised by financial, subsistence and complex socio-cultural factors [1,16–20]. While some anthropogenic causes, including direct persecution, alongside habitat modification and loss, are well documented primary causes of African large carnivore population declines and extirpations [1,17,21], other factors, like cultural demand drivers remain relatively poorly understood across multiple species and geographic regions [16]. These underexplored drivers have received comparatively less research consideration compared to other anthropogenic threats, such as conflict with communities and farmers, local hunting, and trophy hunting [16,18,19,22]. Moreover, research and public debates have predominantly focused on larger and more charismatic species, like large felids, exemplified by investigations on the inter-continental lion and tiger bone trade (e.g., [23–26]). This bias emphasizes the necessity for more studies to include smaller, less charismatic, and neglected carnivore species [27]. Addressing these gaps is crucial for accurate risk assessments and the development of appropriate conservation interventions in the face of current and future threats. Hence, with these considerations in mind, we set out to investigate the culturally-mediated threats to wildlife in Africa, using 33 carnivore species as focal taxa.

Carnivore use has long been embedded in diverse cultural and religious practices across the African continent and cultural uses continue to arise and evolve (e.g., [16,28–34]). Excavated zooarchaeological remains from African societies have been instructive for understanding pre-colonial economies, traditional practices, and the purposes for using wild animal products. In eastern Senegal, for example, evidence suggests that medieval societies engaged in hunting leopard (*Panthera pardus*), serval (*Leptailurus serval*) and wildcats (*Felis lybica*) as components of their wildlife economies [35]. Similarly, faunal remains from South Africa shed light on traditional practices in the Kalahari after 1600, revealing the use of taxa such as civet (*Civettictis civetta*), genet (*Genetta* sp.), striped polecat (*Ictonyx striatus*), African striped weasel (*Poecilogale albinucha*), wildcat (*Felis* sp.), lion (*Panthera leo*), and leopard for clothing, with leopard skin cloaks serving as symbols of royalty [36]. Larger African carnivores like leopard and lion are commonly revered across sub-Saharan Africa as powerful, fierce, and dominant animals symbolizing strength and courage [37,38]. Their body parts – like skins, claws, and teeth – are therefore often symbolically associated with empowering and protective qualities and regarded as icons of leadership, authority, wisdom, fear, spirituality, wealth and prestige [37,38]. In some regions, these large felids and their parts are ubiquitous among royalty and reserved for higher-ranking individuals, while in others, they are more broadly sought after by various societal groups. For example, leopard skins are highly prized for their ceremonial significance among traditional royalty, such as the amaZulu, Barotse, amaSwati, and Ngoni kingdoms, and more recently within the growing Nazareth Baptist Church (also known as Shembe) with an estimated 8.8 million followers [39], primarily in South Africa’s KwaZulu-Natal and Gauteng provinces. According to this Church, every man is the *inhlokoi* (head) of his household, which grants him the right to wear leopard skin, a symbol long associated with Zulu royalty ([40]; N.S. Mbongwa, personal communication, December 10, 2024). In contrast, smaller species perceived to be cunning are frequently used as symbols of good fortune (e.g., *P. albinucha*) [41–44]. Cultural uses of wildlife are, however, dynamic and not immune to changes in symbolism and significance over time [45,46].

To date, research into culturally-mediated uses and extractive practices involving carnivores have mostly focused on a limited suite of specific species, geographies, ethnic groups, purposes, supply chains and academic disciplines. However, there are over 2,000 ethno-linguistic groups across Africa [47,48], excluding clans, each with their own cultural identities and customs, some of which may overlap. Many of these cultures have traditions of using wildlife for purposes such as attire, zootherapy (medicine), and bushmeat, but the pervasive usage of carnivore parts, especially in traditional ceremonies, has not received the level of consideration it seems to warrant [16].

A broader understanding of carnivore trade systems is thus ideally needed to identify the cultural determinants driving trade and to characterise these systems more completely [16]. A synthesis of available information is also warranted to reveal the *status quo* and identify cross-cutting patterns, and continent-wide trends, influencing their cultural use and trade across Africa. This study therefore aimed to investigate evidence for the cultural uses of 33 carnivore species across Africa by conducting a systematic mixed-methods review and analysis of incidence records gleaned from published data sources, databases, and video footage. The specific objectives were to: (i) investigate, identify and characterise the main types of cultural uses behind the hunting, extraction and trade of selected carnivore taxa, and examine the Africa-wide occurrence and extent of these practices; (ii) identify regional and national nexuses of African trade and trafficking (source and sink countries); and (iii) explore factors that may perpetuate the utilisation and trade of certain species and products. Additionally, this study explored potential information biases within the publication and observation records.

In this review, the emphasis lies on geospatial evidence for cultural use and trade, particularly in the context of traditional attire worn in traditional ceremonies. Our intention here is not to unnecessarily vilify cultural and customary practices, but rather to promote an informed understanding of their potential repercussions for wildlife populations and the sustainability of these practices. We also aim to foster awareness and, where appropriate, encourage the integration of the cultural significance and implications of these practices into conservation frameworks and thereby avoid irreparable population depletion – without endorsing their utilisation as Trojan horses to conceal illicit, population-threatening activities.

## Methods

### Mixed-methods review

Traditional and customary uses of wildlife in Africa tend to be under-reported and fragmented in the scientific literature. A mixed-methods review was therefore conducted to retrieve qualitative and quantitative data from publications (journal, non-journal, newspapers) and visual media (videos). This review, along with the resultant meta-synthesis and bibliometric analysis, focused on selected African carnivore species and synthesized information related to diverse cultural practices, trade and incidents of trafficking. The review excluded (i) information not related to traditional or customary use and trading of these taxa in Africa, and (ii) reports of trade and trafficking linked to European and Asian markets and multi-national syndicates, as these are believed to typically reflect non-African demand for African products.

### Species identification and taxonomy

The review covered 33 carnivore species: all African Felidae (11 spp.), Viverridae (16 spp.), Nandiniidae (1 sp.), and terrestrial Mustelidae (5 spp.). The focal species, three of which are distinctively large, are typically characterised by recognisable spotted or striped skin patterns and colours, which facilitated species identification in the video records. To ensure consistency, taxonomic nomenclature was standardised across the review dataset following the taxonomy in Kingdon and Hoffmann [49], except for Felidae for which the updated classification in Kitchener et al. [50] was used.

Where data were obtained from textual sources, the accuracy of the original authors’ species identifications were generally accepted without further scrutiny. However, in cases where uncertainty arose, a ‘morphospecies’ designation was assigned – which involves categorizing a record based on morphological traits and distinctiveness, akin to a genus-level classification, such as genet (*Genetta* spp.), civet (*C. civetta*, *Nandinia binotata*), wildcat (*F. lybica*, *F. nigripes*), polecat (*Ictonyx* spp.), weasel (*Mustela* spp). When researchers encountered species identification challenges during their field observations of pelts and body parts (e.g., for wildcats and genets), or were non-specific, these records were often assigned to a morphospecies in those publications. For data gleaned from videos, species-level assignments were made if there was confidence in the identification, while unclear cases were treated as morphospecies.

Excluded from this review are 48 other African carnivore species, namely aquatic mustelids (4 spp.), herpestids (26 spp.), canids (12 spp.), hyaenas (4 spp.) and seals (2 spp.). Species inclusion and exclusion were guided by pragmatic practical considerations and research resource availability during the COVID-19 pandemic (when the review commenced). These considerations encompassed species detectability, species distinguishability, researchability, observed and anecdotal information on prevalence in wildlife markets and cultural events, the diversity of cultural perceptions and specific uses for the morphospecies, cultural substitutability of the taxa, similarity of cultural and ecological niches, data collection and manageability, resource constraints, time limitations, and cost effectiveness. Including all ± 81 African carnivore species in this review would have exponentially increased the volume and complexity of data to manage and analyse (see later), and would have constrained the feasibility and focus of the research. We thus made carefully considered decisions to exclude certain taxa and acknowledge the need for further research to likewise investigate their cultural uses.

### Publication search

A systematic search was conducted in English and French across various sources and platforms, including journal articles (peer-reviewed), non-journal publications (‘grey’ literature – i.e., articles, books, chapters, reports, dissertations, theses, conference proceedings, newsletters, government documents), as well as newspaper content. For the journal and non-journal source searches, keyword strings that included carnivore names (binomial and common), use categories (Table 1) and African countries, were employed to search bibliographic databases, online research portals and search engines such as SCOPUS, Google Scholar, Google, OpenGrey, as well as online repositories like the IUCN/Species Survival Commission (SSC) Cat Specialist Group library, Red Lists (Global and South Africa), and LAGA (https://www.laga-enforcement.org/en) records (S1A Appendix). There were no limitations on publication date, and we reviewed articles published from 1907 to 2020 (S2 Appendix). All selected publications were further cross-referenced to identify additional data sources. For inclusion in the review, publications had to include three parameters: (i) the name of at least one focal taxon *and* (ii) data for an African country that could be assigned to (iii) at least one of the 13 categories in Table 1.

**Table 1:** Categorisation and descriptions of traditional use, trade, and incidents of mutilation (for brevity, categories #2–4 are merged in some analyses).

Categories				Cat. #	Lit. #	Ctry #	MSp #
**Traditional Attire**	Skins, claws, teeth, bones, tails that are part of traditional/customary regalia, dress and decoration. Worn for ceremonial, ritual and daily reasons (incl. annual festivals and gatherings). Sub-category based on wearer.	Traditional leaders		**1**	119	22	7
		Other persons	Tribespersons	**2**	146	32	13
		Other persons	Political leaders	**3**	23		
		Other persons	Religious	**4**	17		
**Non-Attire**	Skins, claws, teeth, bones, meat, internal organs and other derivatives used for:	Traditional medicine, zootherapy, *umuthi*		**5**	175	36	13
		Bushmeat / food		**6**	119	30	13
		Musk		**7**	8	1	1
**Incidents**	Reports of part removed from animals, or reports of persons in possession of such parts	Poaching, hunting, human-wildlife conflict		**8**	112	32	10
		Confiscations, arrests		**9**	123	35	10
**Trade, markets, shops**	Records where the specific use of the parts cannot necessarily be determined, but they have been observed or recorded in trade	Market observations		**10**	60	32	13
		Curios, trinkets, tourism		**11**	28	14	7
		Skins (not specified)		**12**	50	24	9
**Cultural use** (not specified)				**13**	23	23	11

Cat. # = category number, described in notes below; Lit. # = number of article sources; Ctry # = number of countries (includes video evidence); MSp # = number of morphospecies recorded and/or observed (includes video evidence)

**Notes on categories and their parameters (Cat. #):**

1. Applies to Kings, tribal chiefs, higher-ranked individuals within a community.

2. Applies to traditional healers, dancers, lower-ranked persons within communities, dancers and attendees at festivals, civilians, royal servants.

3. Example: Hastings Kamuzu Banda’s lion tail fly whisk (Malawi).

4. Includes the Shembe (members of the Nazareth Baptist Church, based mostly in South Africa).

5. Encompasses traditional healthcare practices at a broad scale due to the spiritual nature of some of these practices. Hence, this category includes witchcraft, magic, fetish, and others, and also the paraphernalia used in these practices (e.g., divination sets, totemic symbols).

6. Meat consumed instead of livestock and poultry. Includes ritual and day-to-day consumption.

7. Obtained from the perineal glands of African civets.

8. Especially in connection with lions and the discovery of mutilated remains where skins, paws and muzzles were removed.

9. Confiscations of parts and/or the arrest and prosecution of persons found to be smuggling, trafficking and/or in possession of material (especially spotted-carnivore skins), and illegal wildlife trade reported outside of markets.

10. Observations of carnivore parts in traditional markets (e.g., bushmeat, medicine).

11. Material specifically recorded for sale to tourists.

12. Records of trade in skins where the traditional purpose was not specified, but which one would presume to be for traditional attire.

13. Cultural use where the specific uses, and parts used, are not stipulated and are only described in the publication as being for cultural/traditional purposes.

A comprehensive search for relevant international news media content was conducted using the Nexis Uni® academic search engine (www.nexisuni.com) through a systematic online newspaper search. A keyword string search was performed that included name terms for the focal taxa, African countries, uses and body parts (S1B Appendix). The initial search yielded > 3 million results, which were reduced to > 600,000 by applying search delimiters such as date (01 Jan 1979 to 31 December 2020), location (international), ‘geography by document’ (Africa), and language (English). To remove duplicate articles, the Nexis® ‘group’ function was selected to cluster and deduplicate articles pertaining to the same story. The remaining records (displayed online as article ‘snapshots’ with highlighted keywords) were then manually searched by decade, and articles referring to wildlife use (according to the categories defined in Table 1) were selected. Full-text articles of the selected results were downloaded and saved as PDFs and subsequently reviewed for direct relevance to the investigation; these downloads did not include the original photographs and graphics. Additionally, the website www.allAfrica.com (which aggregates articles from hundreds of newspapers across Africa) was searched for French-language articles using keyword strings that included focal species names, use categories and countries.

### Visual media search

The visual media search was limited to YouTube videos and focused on supplementing the literature with observations of the focal taxa being incorporated into African traditional attire. The search was in five stages. First, keywords for the names and places of 18 cultural events identified in the Nexis® newspaper review were investigated further by viewing video content recommended by YouTube – for example, *Lusata* and *Tulikonge* (Namibia); *Dimi* and *Irrecha* (Ethiopia); *Bene Mukuni* and *Ukusefya pa Ngwenya* (Zambia). Second, combinations of keywords like ‘*traditional, cultural, festival, ceremony*’ and the names of African countries were used. Third, events identified in Google, Wikipedia and Music in Africa (www.musicinafrica.net) were searched with combinations of the keywords ‘*traditional, cultural, festival, ceremony, Africa*’ for names of cultural events. Fourth, videos uploaded to the African Digital Ethnography Project channel were viewed (www.youtube.com/c/AfricanaDigitalEthnographyProject). Finally, to identify additional relevant content, YouTube’s auto-generated personalised video algorithms, which are based on previous viewing history and reinforcement learning, were used in conjunction with additional keyword searches on the platform, employing the snowball method. Observations on the presence and quantities of the focal taxa at the events depicted in the videos were recorded.

### Data classification

The 13 use, trade and incident categories (outlined in Table 1) to which evidence was assigned were informed by the content and search order of the publications. Initially, categorisation was based on content from journal and non-journal sources, with newspaper articles being considered later in the review. As the review progressed and other types of use became evident, the categories were refined and adjusted. Consequently, all previously categorised literature was re-examined and reclassified as necessary. Newspaper articles from Nexis® were particularly important for defining new categories because they provided detailed information on different issues and events compared to non-media publications. For instance, the category of ‘traditional attire’ was subsequently divided into four sub-categories based on the different types of wearers cited in these articles (traditional leaders, and three types of ‘other’ persons; Table 1) and usually in conjunction with specific cultural events that the wearer(s) attended; data analyses were conducted either with all four attire sub-categories or with the two broader sub-categories (leaders and other). Additionally, the category for incidents (poaching, hunting, and human-wildlife conflict (HWC)) was added based on recurrent media reports of these events – however, these articles were only included in the review if they mentioned body parts having been removed from animals for allegedly African cultural purposes.

Records (published and video footage) of cultural events that wearers of traditional attire attended were categorised according to the seven key types of ceremonies, festivals and rituals outlined by Mkandawire et al. [51]. These classifications are based on the distinctive characteristics and specific timing of the events, namely: (i) *calendrical or seasonal*: important annual cultural events taking place at specific times of the year or season, like planting or harvesting; (ii) *contingent*: events held to mark the transition from one phase of life to another, encompassing rituals performed at birth, puberty, marriage, death, coronations and elections; (iii) *affliction*: rituals aimed at appeasing or warding off supernatural beings or forces believed to have brought illness, bad luck, or physical injuries to individuals or the community; (iv) *divinatory*: ceremonies performed by traditional healers, spiritual elders, religious leaders, and political authorities to ensure the well-being and fertility of humans, animals, and crops within their territories; (v) *initiation*: events introducing younger individuals or generations to a new phase of life or a particular lifestyle, such as to adulthood, secret societies, or traditional healing and religious professions; (vi) *regular and daily*: performed on a regular or frequent, but not always predictable, basis, sometimes even daily, e.g., offerings, prayers or tributes to ancestors; (vii) *private or secret*: conducted privately by specific members of a community or secret societies. Events or dances that could not be assigned to one of these seven categories were labelled as ‘NC’ (Not Classified). Participants may incorporate skins of the focal taxa into their traditional attire at any of the events, depending on the conditions of their tradition of practice. Event details are summarised in S6 Table.

For insights into the relative amounts of skins worn in traditional attire (from video evidence only), these cultural event types were assigned to categories based on quantity classifications reflecting the combined numbers of people observed wearing products from the focal species. The quantity classifications are: (i) Low (L) =  up to 10 persons observed wearing either a full skin or skin fragments of varying sizes, with typically less than five people seen wearing parts from at least one of the taxa; (ii) Medium (M) =  more than 10, and up to 50, persons observed wearing full skins and skin pieces of varying sizes of the focal species, often including participants wearing more than one skin (typically male event participants); (iii) High (H) =  more than 50, and sometimes exceeding 100, participants (generally male) observed wearing full skins and skin pieces of varying sizes, with persons frequently wearing multiple full or half skins; and (iv) Range (R) =  events necessitating further investigation, and/or where the number of focal species skins involved is variable and could span the range from L to H, due to insufficient video footage to make quantity determinations. All records were verified through the review of 555 YouTube videos.

### Data collation and meta-synthesis

The data from the reviewed publications were disparate, qualitative and quantitative, and sometimes messy, depending on the original research purpose and record-keeping methods. Additionally, some data sources contribute to multiple taxa, countries and use categories. To effectively capture and analyse the data from all information sources, the relevant information was simplified, categorised, standardised and coded as presence/incidence-based data only (as the quantities of animals and parts were not always provided or evident). It should be noted that, in ‘decoupling’ the incidence-based data from the abundance-based data, some synthesized results are not always directly comparable. Consequently, resultant record frequencies do not necessarily always reflect the relative abundances of harvested, utilised and traded species and products.

As indicated earlier, for inclusion in the review, each publication had to have information for three parameters: at least one of 33 species (or 14 morphospecies) linked with data for one of 48 African countries (or ‘Africa’) that could be assigned to one of 13 use, trade and incident categories. This requirement allowed for up to 20,592 potential data points (33 species x 48 countries x 13 use categories) to be captured per publication. Each data point was entered in an Excel spreadsheet as ‘1’ for presence. Thus, each publication contributed to a multi-dimensional matrix with potentially numerous incidence-based records, reflecting the complex and varied nature of documented cultural use across species, countries, and use categories. Figures, tables and analyses are based on incidence totals for the number of records for species, countries and categories, and their combinations, unless otherwise specified, and were produced in Excel.

To elucidate geospatial and temporal patterns for specific parameters (taxa, countries, use and incident categories), bibliographic analyses were performed based on the number of publications. Additionally, to assess potential biases in the research and reporting relative to species body size, plots of the number of publications versus body size of species were created. The maximum adult body mass of each species was sourced from literature (ASCaris http://ascaris.org; [49,52,53]), and taxa were classified as small (<7 kg), medium (7–20 kg) or large (>35 kg) based on mean body size categories defined for felids in Nowell and Jackson [52] (S1 and S2 Tables). Regression lines were included in the species mass *vs* literature frequency plots primarily to visualise which species were above or below the line of regression rather than to assess the strength of the correlation between the parameters. The R^2^ values are included to provide additional context; however, we do not discuss them as they do not directly impact the primary objectives of the study.

To analyse the trading and trafficking of products and species, incidence-based network and nodal analyses of inter- and intra-regional trade were performed. A matrix was created to capture the source (exporting/supplying) and sink (importing/receiving) countries linked by trade. Significant nodes in the network analyses were identified through chi-squared tests, which assessed the frequency of connections for each country relative to the overall network. Countries with higher-than-expected frequencies of trade connections to other countries were deemed significant. However, the available information on trade connectivity between countries was also sometimes messy, inconsistent, and incomplete across the publications. Therefore, the qualitative (presence/absence) data were limited to reporting only on the number of linked countries involved, *not* the magnitude of trade (i.e., numbers of animals or parts) *nor* the frequency of trade reports for each pair of one- or two-way linked countries, *nor* the legality thereof. It should thus be emphasised that our analysis does not imply causality, and we do not infer that the most interconnected countries supply or receive (illegally or legally) proportionally more species and animal products more frequently, and vice versa.

### Mapping

The current and historic country ranges for each taxon were obtained from IUCN Red Lists, Kingdon and Hoffmann [49] and Kitchener et al. [50], and used to categorise the distribution status of species in each country as extant, extinct, no occurrence, or uncertain occurrence. The geographic classification and assignment of African countries to regions followed the United Nations (UN) geoscheme for Africa (https://unstats.un.org/unsd/methodology/m49/) (S1 Map), where ‘Middle’ Africa in the UN classification corresponds to ‘Central’ Africa.

## Results

### Data sources

#### Publication and video count.

**Fig 1:**
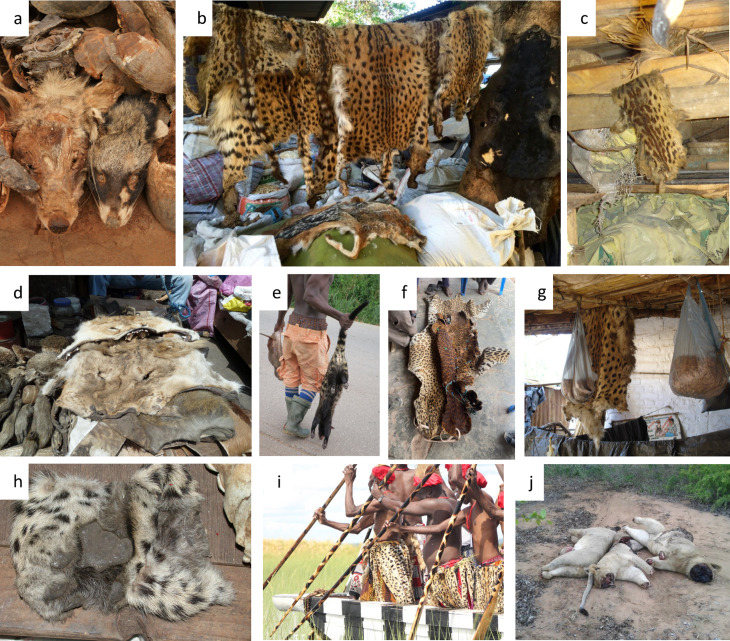
Examples of cultural use, trade and incidents in Africa for: (A) Black-backed jackal and African civet skulls in a traditional medicine market in Burkina Faso, Ouagadougou, April 2008 [© Tony Cunningham]; (B) Genet, serval and black-backed jackal skins, South Africa, Faraday market, October 2017, [© Jacob Calle]; (C) Large-spotted genet skin, Zambia, Mufuliwe market, 2005 [© Vivienne Williams]; (D) Lion skins, Senegal, Dakar, April 2011 [© Panthera/Philipp Henschel]; (E) African civet, Ghana, 2021 [© Maxwell Boakye]; (F) Ceremonial leopard skins, Ghana, Savannah Region, February 2022 [© Panthera/Marine Drouilly]; (G) Cheetah skin, Malawi, Balaka market, October 2005 [© Vivienne Williams]; (H) Leopard paws, South Africa, Faraday market, April 2011 [© Vivienne Williams]; (I) Lozi paddlers wearing synthetic Heritage Fur lipatelo skirts during the Kuomboka Ceremony, Zambia, 2022 [© Panthera/Gareth Whittington-Jones]; (J) Poisoned lions with missing paws and face, Mozambique, Massingir Velo, January 2018 [© Panthera/Kris Everatt]. Reprinted under a CC BY licence, with permission from (A) Tony Cunningham, original copyright [2008], (B) Jacob Calle, original copyright [2017]; (C) Vivienne Williams, original copyright [2005], (D) Philipp Henschel, original copyright [2011], (E) Maxwell Boakye, original copyright [2021], (F) Marine Drouilly, original copyright [2022], (G) Vivienne Williams, original copyright [2005], (H) Vivienne Williams, original copyright [2011], (I) Gareth Whittington-Jones, original copyright [2022], (J) Kris Everatt, original copyright [2018].

**Fig 2:**
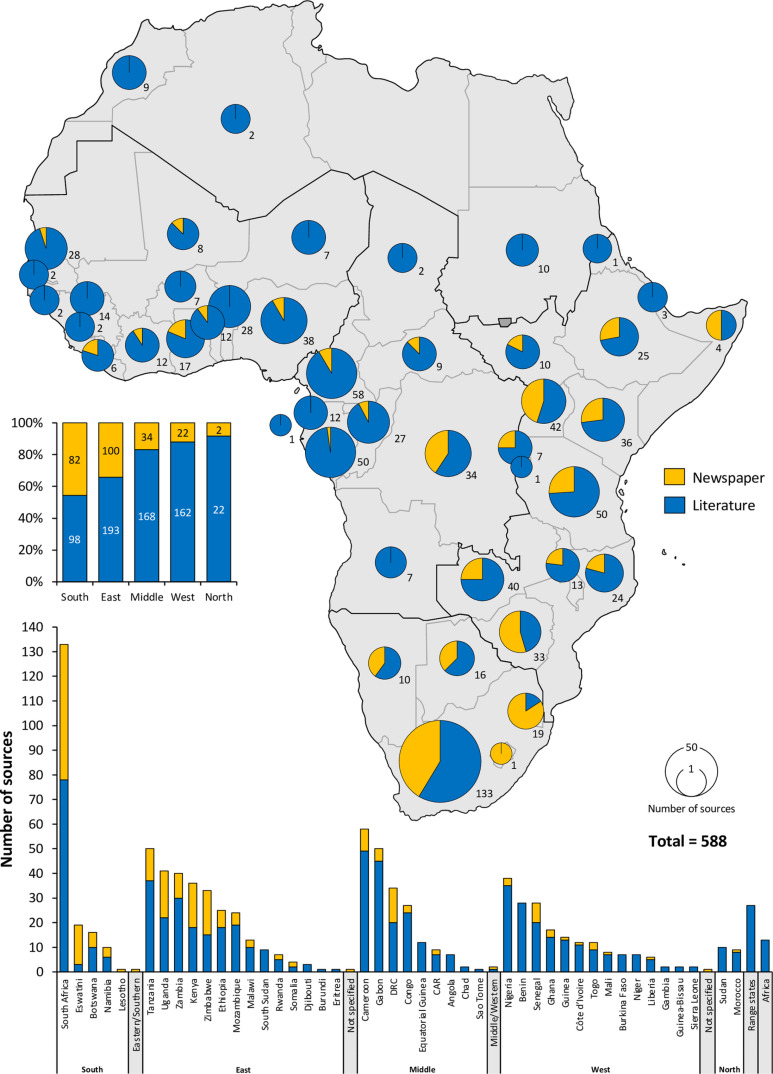
Number and proportion of publication evidence for the 33 species in 48 African countries in five regions, obtained from newspaper articles (yellow; N = 236) and literature (journal and non-journal) (blue; N = 352) between 1907 and 2021 (Total = 588 articles and information sources). Regions are delimited by heavier outlines.

**Fig 3:**
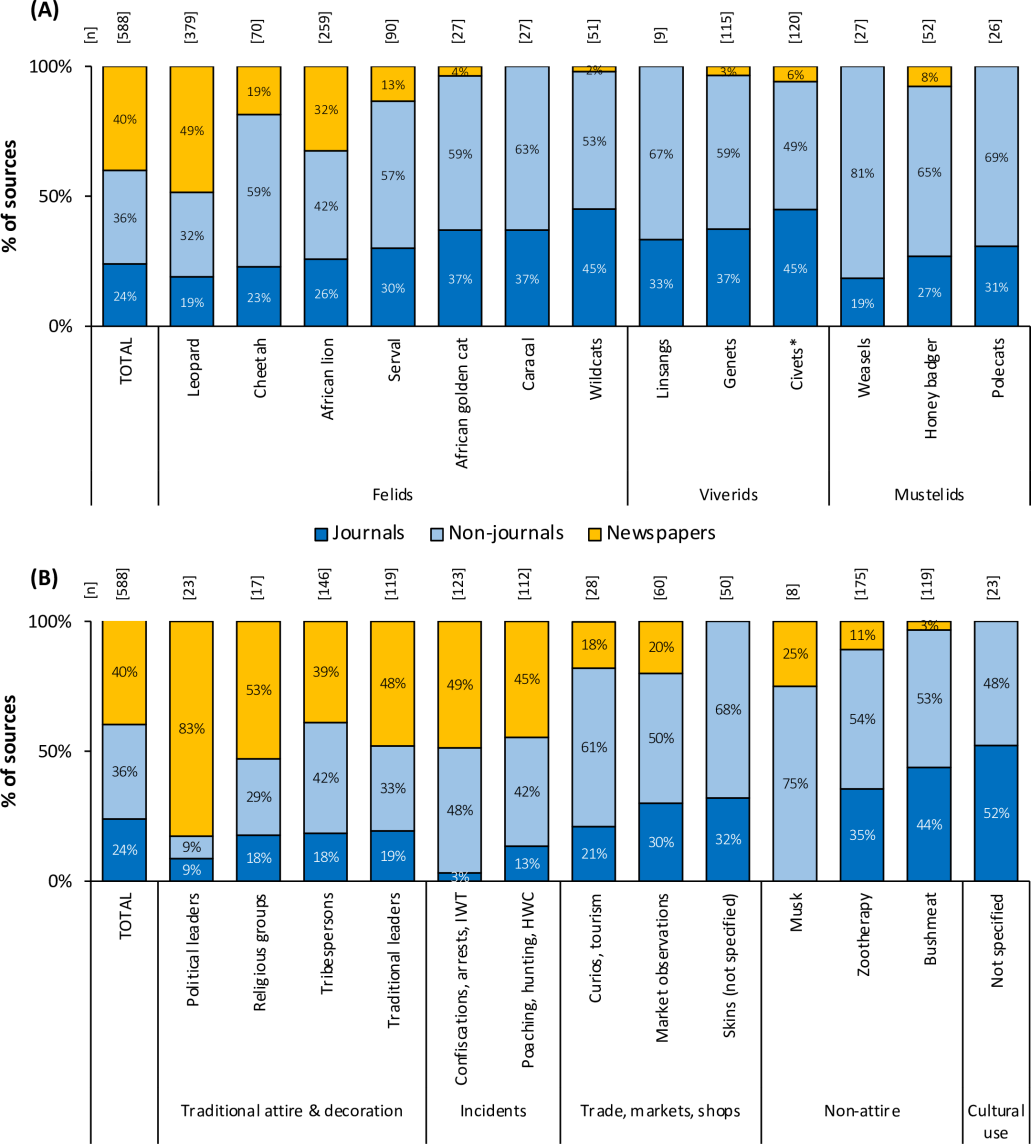
Percentage contributions of journal, non-journal, and newspaper sources to the data for (A) the 33 focal species, consolidated as 13 morphospecies, and (B) categories of cultural use, trade and incidents.

**Fig 4:**
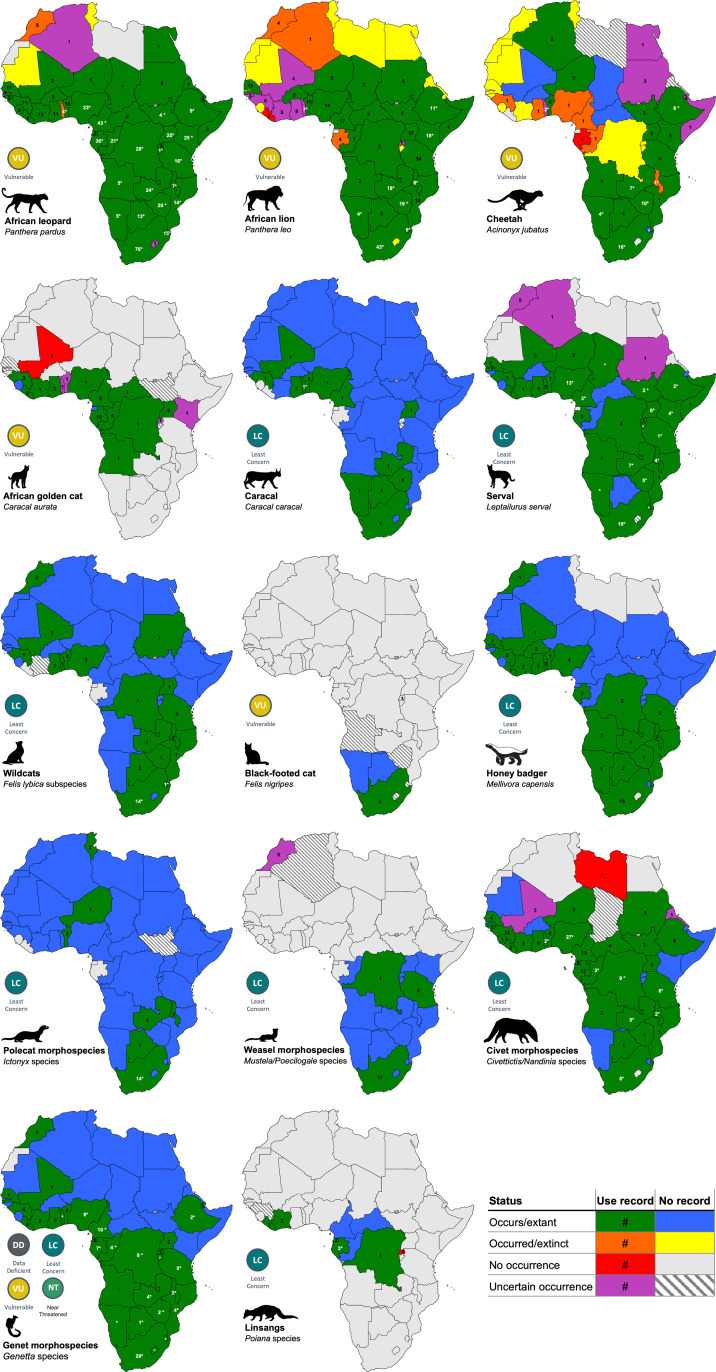
Geospatial mapping of the evidence for cultural use, trade, and incidents of the focal taxa across Africa: literature survey and video evidence (white font with asterisks) by country, with record counts. White asterisks only indicate video evidence. Country colours indicate species occurrence classes and whether records were found during the review. Global IUCN Red List status indicated. Full page versions of these maps are in S2.1 to S2.36 Maps.

**Fig 5:**
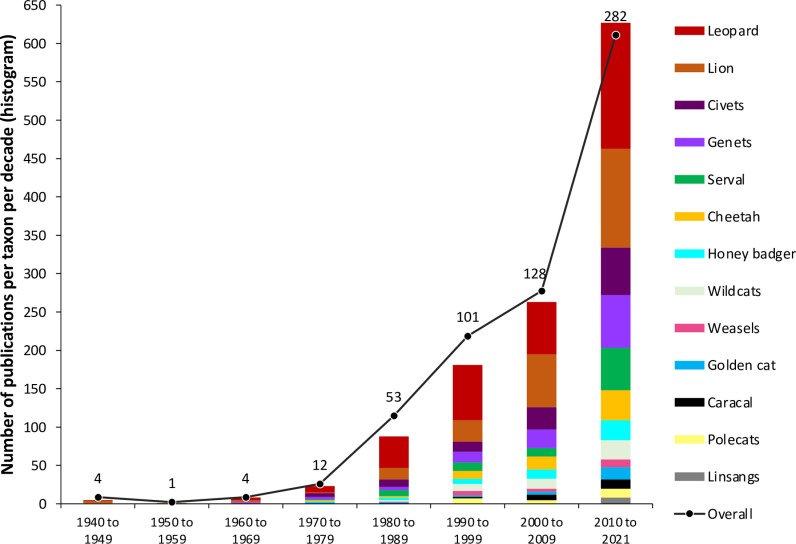
Temporal distribution of the cumulative number of publications and data sources for 33 species consolidated per morphospecies (histogram) and Overall (black line) per decade for N = 585 publications (excluding references from 1907 to 1938). Most publications had information for several species. Taxa listed in descending order of total publication counts in the legend.

The review compiled data from 588 published sources, covering 33 species (14 morphospecies), in 13 categories, across 48 African countries (Tables 1 and 2; Fig 1; S3 Table; S4 Table; S2.1–S2.36 Maps). These sources comprised of 138 (23%) journal articles and 214 (36%) non-journal publications (not media) published from 1907–2020, as well as 236 (40%) newspaper articles published from 1979–2020 (Figs 2 and 3). Additionally, 555 YouTube videos were reviewed (S3 Appendix), providing evidence of eight morphospecies incorporated into traditional attire in 23 countries (Fig 4, white font; S5 Table). The number of published sources with relevant data grew from 2010 to 2021 (Fig 5). Among the countries in each region, South Africa (Southern Africa), Tanzania (Eastern), Cameroon (Middle) and Nigeria (Western) had the highest number of published records (Fig 2). There were also relatively more information sources for Anglophone countries compared to Francophone and Lusophone countries in Western and Middle Africa (Fig 2), likely influenced by the predominantly English-based publication search.

**Table 2:** Number and percentage of publications per species and the number of African countries they were recorded in. Fourteen maps in Fig 4 give the geospatial distribution of this information for 14 focal taxa, simplified to the morphospecies level for wildcats, civets, genets, polecats and weasels. Detailed species-level maps for all 33 species are in S2 Maps.

Family	Species	Name	Total publications^(i)^ per species (%) (N = 588)	No. of countries^(ii)^ recorded in (incl. YouTube videos, YT) (N = 48)
Felidae	*Panthera pardus*	Leopard	379 (64%)	45^Y^
	*Panthera leo*	African lion	259 (44%)	37 ^YT^
	*Acinonyx jubatus*	Cheetah	70 (12%)	28^ YT^
	*Panthera & Acinonyx* sp.	‘Big cats’	6 (1%)	7
	*Caracal aurata*	African golden cat	27 (5%)	17
	*Caracal caracal*	Caracal	27 (5%)	13 ^YT^
	*Leptailurus serval*	Serval	90 (15%)	34^ YT^
	*Felis lybica cafra*	Southern African wildcat	21 (4%)	7
	*Felis lybica lybica*	African wildcat	16 (3%)	7
	*Felis lybica* subsp. ^**(iii)**^	Wildcat subsp.	49 (8%)	18 ^YT^
	*Felis nigripes*	Black-footed cat	2 (0.3%)	1
Mustelidae	*Ictonyx striatus*	Striped polecat	24 (4%)	6^YT^
	*Ictonyx lybicus*	Saharan striped polecat	2 (0.3%)	1
	*Mellivora capensis*	Honey badger	52 (9%)	22
	*Poecilogale albinucha*	African striped weasel	21 (4%)	3
	*Mustela nivalis*	Least weasel	5 (0.9%)	1
Nandiniidae	*Nandinia binotata*	African palm civet	41 (7%)	13
Viverridae	*Civettictis civetta*	African civet	92 (16%)	30
	*Civettictis*/*Nandinia* sp.^** (iii)**^	Civet sp.	26 (4%)	20^ YT^
	*Genetta* sp. ^**(iii)**^	Genet sp.	57 (10%)	23 ^YT^
	*Genetta abyssinica*	Ethiopian genet	2 (0.3%)	1
	*Genetta angolensis*	Angolan genet	3 (0.5%)	3
	*Genetta bourloni*	Bourlon’s genet	5 (0.9%)	3
	*Genetta cristata*	Crested genet	5 (0.9%)	3
	*Genetta genetta*	Small-spotted genet	26 (4%)	11
	*Genetta johnstoni*	Johnston’s genet	5 (0.9%)	2
	*Genetta maculata*	Common large-spotted genet	16 (3%)	10
	*Genetta pardina*	Pardine genet	8 (2%)	4
	*Genetta piscivora*	Aquatic genet	4 (0.7%)	1
	*Genetta poensis*	King genet	4 (0.7%)	1
	*Genetta servalina*	Servaline genet	14 (2%)	5
	*Genetta thierryi*	Hausa genet	4 (0.7%)	4
	*Genetta tigrina*	South African large-spotted genet	20 (3%)	1
	*Genetta victoriae*	Giant genet	7 (1.2%)	2
	*Poiana *spp.^** (iv)**^	African linsang	9 (1.5%)	6^ YT^

**Notes:**

(i) Total publications include journal, non-journal and newspaper sources.

(ii) Number of countries includes YouTube video (YT) evidence for traditional attire.

(iii) Entries for *F. lybica* subsp., *Civettictis* & *Nandinia* sp. and *Genetta* sp. are for where the publications and videos provided insufficient information for identifying a species. Consolidated records for the viverrids are: civets (110 publications across 33 countries), and genets (115 publications across 31 countries).

(iv) *Poiana leightoni* and *P. richardsonii.*

Newspapers tended to report more on larger felids, and especially confiscations and the use of skins, claws and teeth in traditional attire (Fig 3A). Peer-reviewed journals, however, provided relatively more data for smaller species such as wildcats and civets (45% each) compared to other sources (Fig 3A; S4 Table), and non-journal publications were the principal sources for documenting observations relating to bushmeat trade, zootherapeutic uses, market trade and curios (Fig 3B; S4 Table). Newspaper articles also provided valuable information that the other published sources generally overlooked (or did not mention in much detail), such as specific dateable observations and reports on (i) the widespread wearing of carnivore products by traditional leaders (Kings and chiefs), healers, tribespersons and political leaders in ceremonial events and rituals that take place across Africa annually, and (ii) details for incidents of HWC and wildlife trafficking, as well as arrests related to these incidents (Fig 3B; S4 Table).

YouTube videos depicting the incorporation of the focal taxa into attire served two main purposes. Firstly, the videos confirmed some of the published country records for the taxa (numbers in white font, Fig 4; S2 Maps), and secondly, they provided new country records for taxa that were not found in the literature search (asterisks only in white font in Fig 4 and S2 Maps). Corroborating video evidence was found for leopard use in 22 of 45 countries, for serval in 16 out of 34 countries, and for lion in 10 out of 37 countries (Fig 4; S5 Table). Notably, the video records confirmed proportionally more countries with people incorporating genet skins into regalia than the published records indicated (17 of 31 countries) (S5 Table). No videos confirmed the use of golden cat, honey badger (*Mellivora capensis*), polecat and weasel skins in traditional regalia, but we suspect their presence based on other evidence and personal observations. Moreover, the video evidence also provided new country records that were absent in the literature review for three taxa, namely genets (in five new countries), serval (four new countries) and cheetah (*Acinonyx jubatus*) (one new country) (asterisks in Fig 4 and S2.1–S2.36 Maps).

#### Body mass and publication numbers.

**Fig 6:**
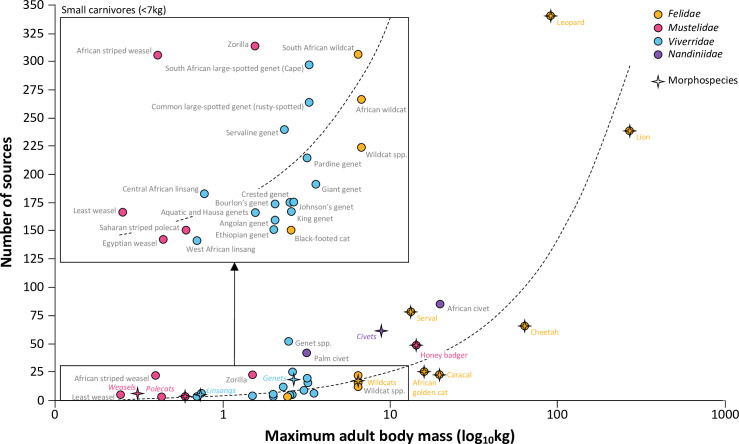
Relationship between number of publications and species and morphospecies adult body mass: Publications (N = 588; for journal, non-journal, and newspaper sources) and 33 species (circles) and morphospecies (stars) maximum adult body mass (as listed in S1 and S2 Tables). The positive relationship between research/reporting effort and body mass is consistent across all publication types (refer to S1 Fig). For specific relationships between body mass and the number of publications within use category types see S2A–I Figs. (R^2^ = 0.57).

Regardless of the information source and the use category, there was a positive correlation between body mass and the overall number of information sources (R^2^ = 0.57). Larger species were more frequently the subject of studies and observations in published sources (Fig 6; S1 and S2 Figs). Furthermore, leopards, servals, civets, and genets were cited relatively more frequently in relation to their body mass compared to other species (Fig 6), and leopards were particularly prominent in newspaper articles (S1 Fig). Conversely, smaller species like polecats, linsangs (*Poiana* spp.) and range-restricted genets received the fewest mentions in connection with cultural practices and related incidents (Fig 6 inset; S1 and S2 Figs).

While all 33 species were recorded in the literature, there exists potential research and reporting biases against smaller species (Fig 6). Newspaper articles reported on only six medium-to-large species (leopard, lion, cheetah, serval, African golden cat, honey badger) and three small morphospecies (civets, genets, wildcats), with notably more mentions for the larger species (S1 Fig). There was also more geospatial record coverage across a greater number of range states for larger species (Fig 8). For species like lion, leopard, cheetah, serval, golden cat, African civet and four species of genet (10 taxa with average weight of 40.8 kg), records were available for more than 50% of the range states they occur in (100% for lion and leopard). In contrast, for caracal (*Caracal caracal*), honey badger, palm civet, polecats, weasels and nine genet species (14 taxa combined with average weight 4.2 kg), we encountered no use records for more than 50% of the range states they occur in (Fig 4; S2.1–S2.36 Maps).

**Fig 7:**
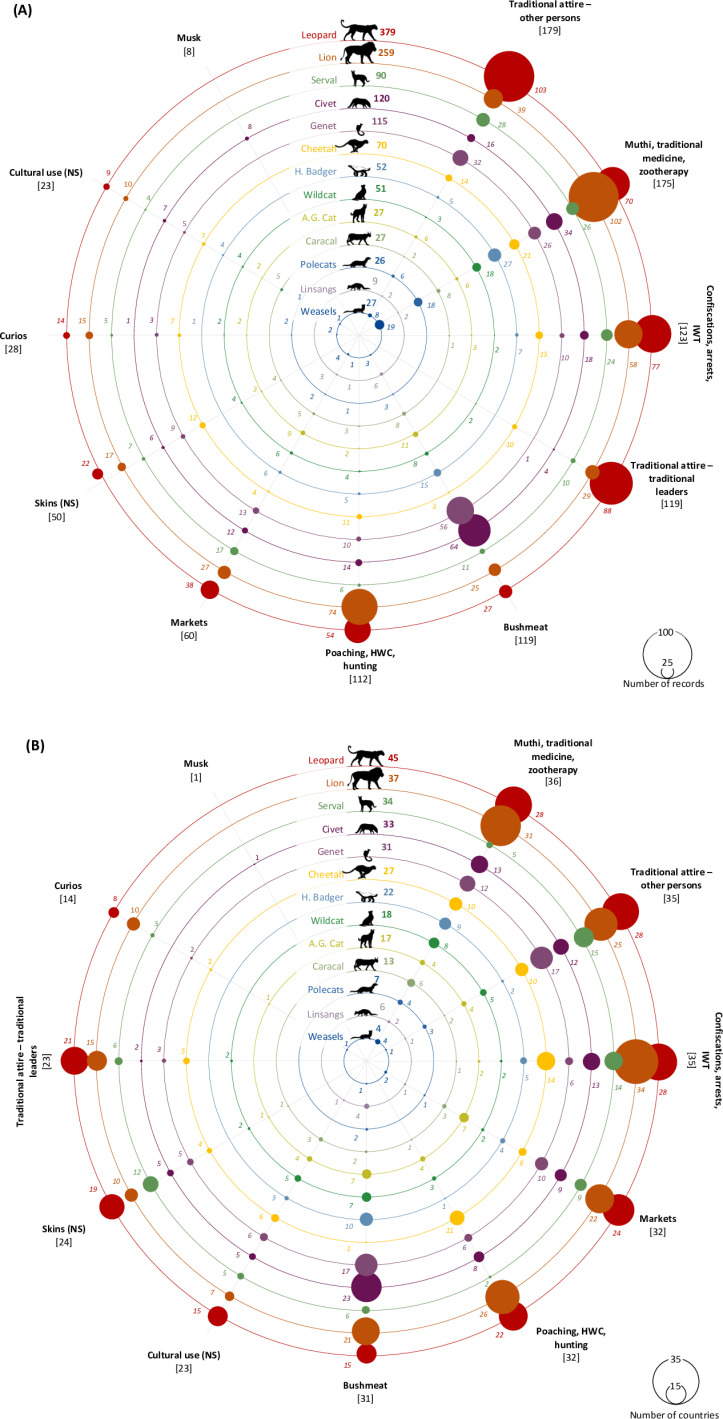
Cultural use, trade, and incidents of the focal taxa, organised radially by category, displaying: (A) *the number of published sources* (journals, non-journals, newspapers) (e.g., 88 records for leopard parts used in the traditional attire of traditional leaders). (B) *the* *number of countries* (e.g., leopards reported in 45 countries, with 28 countries having records for zootherapy). Includes records from YouTube videos. Categories are arranged clockwise, in descending order of the number of records and countries. Some data sources contribute to multiple taxa, countries and categories; hence, the sum of category values may not always equal the overall number of countries.

Large- and medium-size felids were the primary subjects of studies and observations in 11 categories of use, trade and incidents, except for bushmeat consumption (S1A–C, E–I Figs), where civets and medium to small species were more commonly reported (S1D Fig). Lions and cheetahs, however, were consistently reported in fewer sources than the body mass trends would have predicted (as their data point values were below the regression line).

### Geospatial coverage

**Fig 8:**
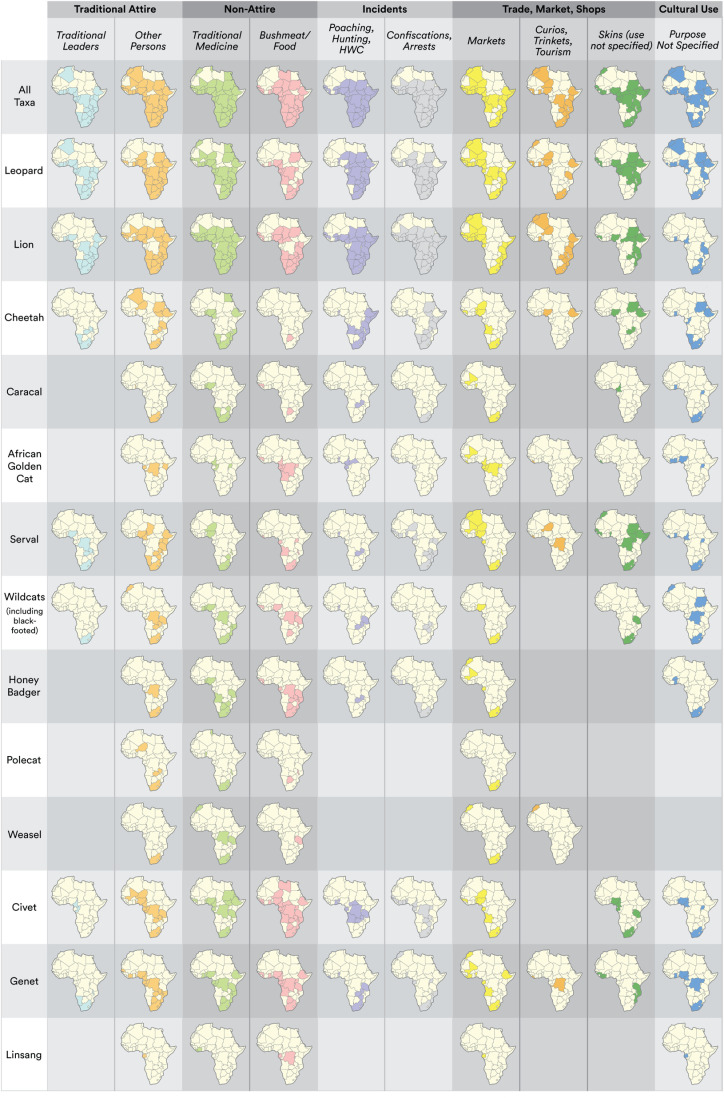
Geospatial coverage in 115 maps of where cultural use, trade and incidents have been documented for the focal taxa in 48 African countries, based on consolidated presence/incidence data from three evidence types (literature, newspapers, videos). Gaps in geospatial continuity for the species and categories do not imply absolute absence of evidence.

Leopard was documented in the most countries from the highest number of publications (45 of 48 countries; 379 of 588 published sources) and observed in videos to be a component of traditional attire in the greatest number of countries (22 of 23 countries with video records) (Table 2; Fig 4; Fig 7; S2.1 Map; S5 Table). Lion was reported in the second highest number of publications and countries, followed by serval and cheetah. Among the Mustelidae, honey badger was the most frequently reported species, particularly in South Africa and Niger, while the African civet was the most commonly reported species among the civets and Viverridae (Table 2; Fig 4; Fig 7; S2.1–S2.36 Maps). Although the small-spotted genet (*G. genetta*) was the most frequently reported genet species, this genus was infrequently identified to species-level. The least recorded species were the small Saharan striped polecat (*Ictonyx lybicus*), black-footed cat (*F. nigripes*) and Ethiopian genet (*Genetta abyssinica*), each with only two literature records (Table 2). (Note that the traditional attire country records in Fig 7B and Fig 8 are the only ones augmented with observations from video evidence.)

Leopard was also the prevalent species in most of the category records (Fig 7A) and was the overall leading documented species for 24 out of 48 countries (Fig 4). In contrast, countries where we found comparatively more records for taxa other than for leopard are: (i) lion, with more records than leopard in Chad, Ethiopia, Guinea Bissau, Malawi, Mali, Senegal, Sudan, South Sudan, Tanzania, Togo; (ii) cheetah, with more records than leopard in Algeria; (iii) serval, in Gambia; (iv) civet, in Nigeria and Libya; (v) genet, in Eritrea; (vi) polecat, in Tunisia; (vii) leopard was tied for records with lion in Burkina Faso, Mozambique and Niger; (viii) and leopard tied with cheetah in Egypt; (ix) leopard tied with golden cat and linsang in Burundi; (x) civet tied with genet in Equatorial Guinea; and (xi) golden cat tied with polecat for records in Morocco (Fig 4; S2 Map).

### Cultural uses, trade, and incidents

Traditional attire was the most documented use type, covering 35 countries and a total of 179 consolidated sources for the attire of tribespersons, political leaders and religious groups (excluding the separate category of attire for traditional leaders) (Fig 7A–B). This was followed by (i) products for zootherapy; (ii) incidents involving skins and parts in seizures, confiscations and arrests; (iii) bushmeat; (iv) attire for traditional leaders; (v) incidents of poaching, hunting and HWC resulting in parts being removed from animals; and (vi) species’ body parts sold in markets (Fig 7A–B; Table 1; S3 Table; S4B Table).

#### Traditional attire use.

The traditional attire incidence records from publications were dominated by information for leopards (Fig 7A; S3A Table). Indeed, citations of leopard in all subcategories of traditional attire far exceeded records for all other species. Other taxa such as lion, serval, cheetah, civets and genets were also reported multiple times, while taxa like golden cat, caracal, small cats (wildcat and black-footed cat) and mustelids were very rarely named (Fig 7A; S3A Table). Among the different sub-categories of traditional attire, the most frequently reported was the association of products with the attire of traditional leaders (119 of 305 publications). The records for attire worn by political leaders, and as part of the Shembe Church, was relatively limited in number (Political leaders 23 of 305; Shembe 17 of 305) and was predominantly limited to species with spotted pelage (Fig 7A; S3A Table). Attire associated with traditional leaders was recorded in publications for most of sub-Saharan Africa except Angola, and was absent from records for much of the northerly parts of West Africa and North Africa, except Algeria (leopard). Attire for other tribespersons was found more widely across the continent, with wider coverage into the Sahel and West Africa (Fig 8).

Newspaper articles reported details on the attire of various dignitaries and tribespersons, including six Kings from five countries, eight political leaders from five countries (e.g., the lion tail whisk of the late Malawian President Hastings Banda), and persons attending 18 annual events in seven countries where five focal taxa were observed being worn (leopard, lion, cheetah, serval, civet) (S6 Table). These 18 named events were used in subsequent keyword searches, which led to video information and results for 78 named events (sometimes tribe-specific) in 22 countries involving eight taxa (S6 Table). These named events excluded non-annual investitures, funerals and weddings of traditional leaders, and ritual ceremonies (including initiation) conducted by traditional health practitioners, which are performed as needed in specific circumstances.

The 78 cultural events where skins from the focal taxa were observed worn in regalia in 22 countries were mostly classified as calendrical/seasonal (44%), such as the *Nc’wala* ceremonies of the ethnonymous Ngoni/Nguni Kingdoms across Eastern and Southern Africa, the *Kuomboka* and *Kufuluhela* ceremonies in Zambia, and the *Irrecha* Festival in Ethiopia (S6 and S7 Table). For 39% of the events, there was insufficient information on purpose and timing to classify them, like the festivals of *Nzem Barom* in Nigeria and the Serengeti Festival in Tanzania. Contingent events, such as installations of traditional leaders, comprised 5% of the cultural occasions documented, followed by initiation events (calendrical and other; 7%) like the *Niembé* initiation dance for women in Gabon, the *Dimi* ceremony in Ethiopia and north Kenya, and the *Umkhosi woMhlanga* (reed dance) in South Africa. Private or secret events, which are conducted privately by specific community members, comprised 3% of the events – like the *Nyau* dancers of the *Gule Wamkulu* Secret Society in Malawi, Zambia and Mozambique. While videos we watched of divinatory and affliction events comprised 1% each of all the events, these practices are very widespread amongst traditional healer communities across the continent and are thus a notable unquantified component of cultural activities that take place in a year. The cultural events that leopard, serval, lion and cheetah are most likely to be observed at are calendrical – such as the *Kuomboka* and Kufuluhela ceremonies, and Eswatini’s *Incwala* ceremony, whereas genet skins were mostly observed at the NC events (S7 Table).

**Fig 9:**
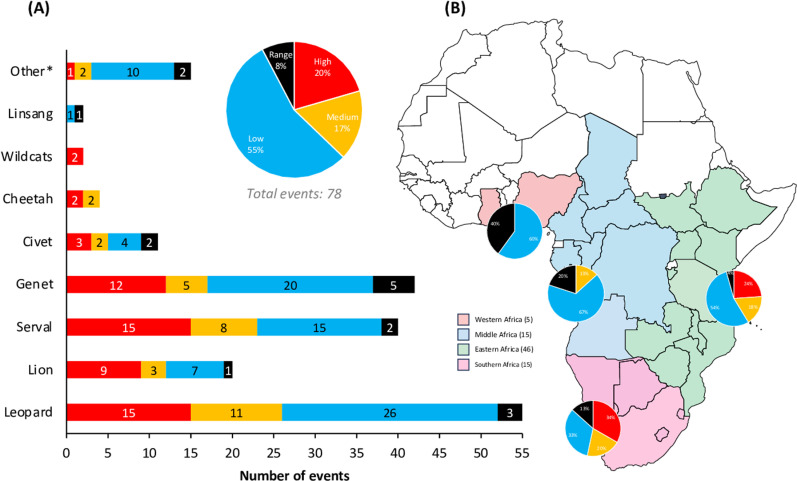
Relative quantities of the focal taxa observed to be worn by participants at 78 cultural events. (A) The number of categorised cultural event types per focal taxa, related to product quantity classifications (high, medium, low, range) reflecting the combined numbers of people wearing products for traditional attire at n = 78 events. (B) The regional relative proportion of these cultural events taking place that have high, medium, or low numbers of people wearing products from the focal taxa for traditional attire (max = 78 events; 8 confirmed taxa in 23 countries). All records verified through the review of 555 YouTube videos. ‘Other*’ include spotted carnivore taxa that were not identified due to poor video resolution and includes tentative evidence for polecat and honey badger. Data are summarised from S6 Table, and quantity classifications are defined in the Methods and S6 Table.

In terms of the relative quantities of focal taxa observed to be worn by participants at these events, 20% of what we viewed in the video footage were classified as having evidence for high quantities, 17% had medium quantities, 55% had low quantities, and 6% showed there to be either a range or indeterminable number (Fig 9A). Skins from leopard, serval, genet, and lion were the most likely to be observed in higher numbers compared to other taxa (Fig 9A). Regionally, cultural events held in Southern and Eastern Africa frequently featured numerous annual gatherings where it was likely that high quantities of the focal taxa would be worn (Fig 9B). Nevertheless, cultural events held across the continent typically involve only a few individuals wearing one or two skins (or parts thereof) at any given time (such as rituals performed by traditional healers, or community assemblies presided over by traditional leaders). However, it is important to note that the cumulative impact of a substantial number of such widespread low-quantity events (like divination practices, day-to-day use, and convened tribal meetings) is mostly underestimated. These practices contribute to the widespread, annual demand, for skins and other products across the continent – especially when older and/or inherited items, sometimes too degraded for reuse, are replaced with newer ones. Such demand is further complicated to quantify due to the private and concealed nature of certain cultural uses.

#### Non-attire use.

Non-attire uses encompassed zootherapeutic (traditional medicine, including rituals) and bushmeat categories, as well as musk derived from African civet and used in fragrances (cited in eight publications). Zootherapeutic uses were reported in the highest number of publications (175 out of 302), followed by bushmeat consumption (119 of 302) (Table 1). Within the publications citing zootherapeutic use, lion and leopard were the most prevalent, while civet and genet species were more commonly associated with bushmeat consumption (Fig 7A; S3B Table). Smaller felids and mustelids received fewer mentions in both these use categories. Zootherapeutic uses for the focal taxa were recorded across most of the African continent (36 countries), with lion having the widest coverage in Sahelian and sub-Saharan Africa. Leopard also had wide geospatial coverage, but other species’ records were patchier and more sporadic (Fig 8).

#### Trade, markets, and shops.

*Panthera* species (leopard and lion) were the most frequently reported in publications citing trade and had the highest observations in markets, with serval also ranking highly and African civet being cited in 10 publications. Publications citing the curio and trinket trade were also dominated by leopard (15 of 28) and lion (14 of 28) observations. Likewise, in the context of the skin trade, leopard (22 of 50) and lion (17 of 50) were the most frequently reported, with cheetah being referred to in 12 publications. However, small felids and mustelids received minimal to no mentions (Fig 7A; S3C Table). Leopard and lion also had the widest geospatial coverage reports in association with informal markets, predominantly clustering in West/Northwest and East/Southern Africa (Fig 8). Other species were recorded in more specific geographies, particularly South Africa (Fig 8). Lion had the widest distribution of mentions in relation to curio and trinket trade, mainly in East/Southern Africa (Fig 8), whereas the use of spotted felid skins was predominantly found in East, Middle, and the Horn of Africa (Fig 8). Unless otherwise specified as IWT, and where information on the legality of trade transactions between some suppliers, traders, markets and shops is absent in the consulted literature, the trade is presumed to be mostly illegal (i.e., not adhering to trade regulations), especially in unregulated informal markets.

#### Incidents.

*Panthera* species were also the most frequently reported in publications concerning poaching, hunting, and HWC, with leopard and lion accounting for 54 and 77 out of 112 publications, respectively. These species were also commonly reported in publications related to arrests, confiscations and smuggling. Cheetah, serval and civet were moderately represented in these publications, whereas mustelids and small cats were rare or absent (Fig 7A; S3D Table). Mentions of poaching, hunting and HWC, as well as confiscations/arrests, were widely reported across sub-Saharan Africa for big felids (Fig 8). Confiscations and arrests related to smaller species were mostly scattered in sub-Saharan Africa (except for genet in Morocco) (Fig 8).

#### Unspecified cultural use.

The utilisation of wildlife for cultural purposes where the specific uses were not stipulated and were described only as being for ‘cultural/traditional purposes’, was documented in 23 countries for 11 morphospecies (Table 1; Figs 7 and 8; S3D Table). Among the species, lions and leopards were the most frequently cited (Figs 7 and 8).

### Trade network analyses

**Fig 10:**
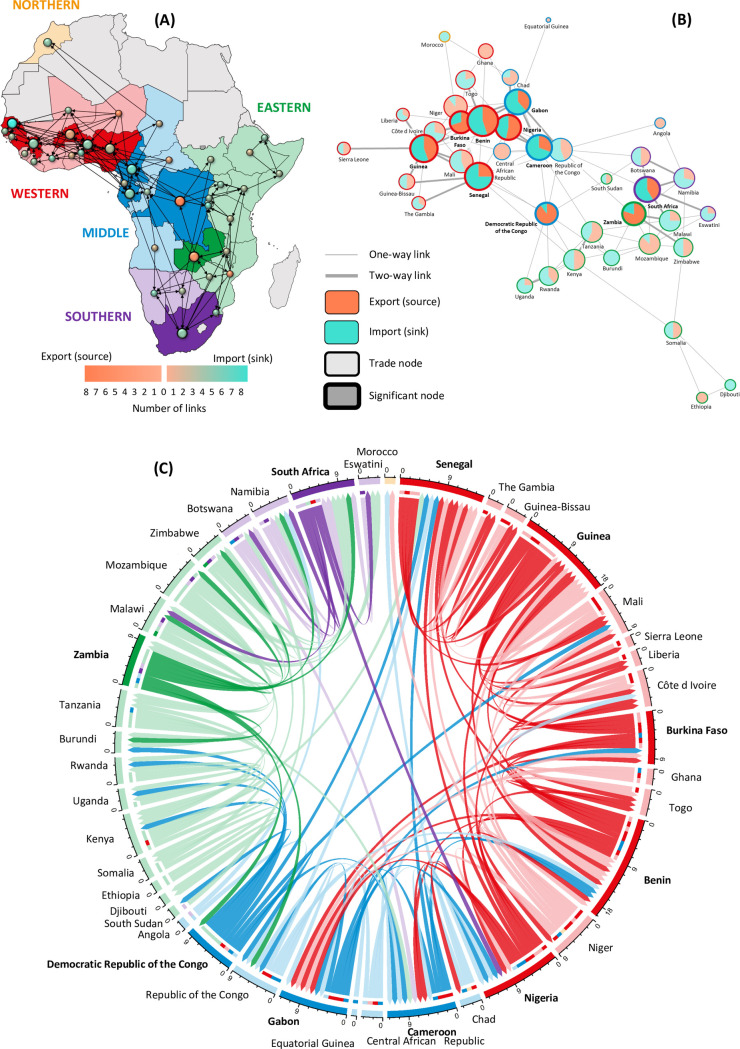
Incidence-based network analyses of inter- and intra-regional trade in carnivore skins and body parts: evidential links among 40 countries for 33 species. Data compiled from journal, non-journal, and newspaper articles (n = 588) were aggregated for source (exporting/supplying) and sink (importing/receiving) countries to present qualitative (presence/absence) evidence for one- or two-way trade links. In the absence of supporting evidence for legal transactions between source and sink countries in the consulted literature, this trade is presumed to mostly indicate illegal or informal activity. Three sub-figures: (A) network structure, (B) node (connectivity), and (C) chord diagrams, illustrate trade links and relative country distribution in Eigenspace based on their trade relationships. The network diagram (in A) highlights countries with mainly source (in orange) or sink (in turquoise) trade links to other countries. The node diagram (in B) highlights the proportion of source and sink links, with significant nodes encircled in bold. Significant nodes, determined by chi-squared tests, indicate countries with more or less connections than random across the trade network. The chord diagram (in C) displays trade links across regions and between countries, but without specifying source and sink roles. Circle and chord colours indicate regions (red=Western Africa, blue=Middle Africa, green=Eastern Africa, purple=Southern Africa). The qualitative (presence/absence) data were limited to reporting only on the number of linked countries involved and *not* the magnitude of trade (i.e., numbers of animals or parts) nor the frequency of trade for each pair of one- or two-way linked countries. We emphasise that the analysis does not imply causality, and we do not infer that the most interconnected countries export or import (legally or illegally) proportionally more species and animal products more frequently, and *vice versa*.

The incidence-based network and node analyses of links between 40 source (exporting/supplying) and sink countries (importing/receiving) for all focal taxa, indicative of trade and trafficking dynamics, revealed distinct patterns of regional and national clusters (of intra-regional trade), as well as longer-distance transfers across regions (for inter-regional trade) (Fig 10). In aggregate, the source countries with the highest number of links to sink countries per region (both proportionally and in number) are Nigeria, Burkina Faso and Niger (Western Africa; 8, 7, 8 sink country links respectively), Democratic Republic of Congo (DRC) (Middle Africa; 9 sink countries), and Zambia (Eastern Africa; 8 sink countries). Conversely, the sink countries per region with the most links to different source countries are Senegal, Guinea and Benin (Western Africa; 12, 10, 11 source country links respectively), Cameroon and Gabon (Middle Africa; 9 and 8 source countries respectively), and South Africa (Southern Africa; 7 source countries) (Fig 10A–B). Countries such as Botswana, Zimbabwe and Kenya had equal numbers of countries that they both supplied to (as sources) and received from (as sinks) (Fig 10B). Furthermore, the countries with the greatest number of links to other countries are Benin (20 countries, 55% of them linked to sinks), Guinea (18 countries, 56% linked to sinks), Senegal (16 countries, 75% linked to sinks), and Nigeria (15 countries, 53% linked to sources) (represented by bigger pie charts in Fig 10B).

The evidence of inter-regional trade in the focal taxa across 31 countries reveals that some of these links are between neighbouring countries in different regions (e.g., Zambia in Eastern Africa is connected to neighbouring DRC in Middle Africa) (Fig 10C). Among the countries with the most inter-regional trade links, excluding their neighbours, were the (i) DRC (source) to Kenya, Mali and Senegal (sink countries), and (ii) Gabon (source) to Nigeria, Benin and Burkina Faso (sink countries) (Fig 10C). There are several long-distance trans-regional links, involving trade between distant countries, which includes trade between: (i) Botswana (source) and Cameroon (sink); (ii) Burkina Faso (source and sink) and Gabon (sink and source); (iii) CAR (source) and Senegal (sink); (iv) Congo (source) and Senegal (sink); (v) DRC and Mali; (vi) Kenya and Senegal; (vii) South Sudan and Botswana; and (viii) South Africa and the DRC.

While it was not practical for this review to specifically identify and elaborate on all species involved in all documented trade links, nor the legality thereof, due to the high number of records assimilated, the records generally indicated that leopard, lion, serval and civets are among the most frequently reported species in the literature. However, we also note potential biases towards recording larger, identifiable and more charismatic species compared to smaller, less conspicuous ones. Furthermore, the available information suggests that the trade activities and transactions were mostly non-compliant with national regulations and international agreements such as CITES (e.g., without permits), and are thus presumed to be illegal. In summary, the trade network analysis focused on the number of linked countries without incorporating the information on the magnitude, frequency or legality of traded animal products, and we acknowledge the need for further focused research in this regard.

## Discussion

### Culture, complexity and context

There is a culturally endowed legacy of carnivore utilisation across the African continent, encompassing diverse traditions of practice and unique perspectives on wildlife use and conservation principles. These cultural practices are inherently complex and nuanced, and we are cognisant that, while the videos generously displayed these rich traditions, much of the existing literature we reviewed overlooked, generalised, disregarded, or oversimplified traditional knowledge and complexity, as well as the impact these practices have on species. Our approach to this review, shaped by the extensive body of material we consulted, also necessitated a certain degree of reductionism. This approach, while a requisite for syntheses and inherent in reviews, inevitably further attenuated some of the nuances and details that are integral to fully understanding cultural use and the magnitude thereof in Africa. Instead of vilifying these enduring practices, however, we sought to elucidate and draw attention to them as under-considered and underestimated drivers of hunting and poaching for domestic markets in Africa. We thus wish to endorse the need to conduct further research and probe ways to sustainably support and maintain the continuation of these practices where feasible, without exacerbating threats to species.

### Patterns across the African continent

The most reported large, medium and small taxa are leopard, serval, and genets, respectively. There are no standard practices of how and why these species and their products are used, and their usage varies across African countries. Cultural norms, regulations, and values associated with product use are multifaceted, species-specific, and vary among different ethnic groups and cultures (e.g., for lions in traditional healing practices in South Africa [32]). Any changes in customary product use and practices (e.g., adoption and revision) may be influenced by factors such as resource availability, substitutability, immutability, commutability, adaptability, new information from cross-cultural exchanges with fellow Africans and foreigners, guidance by traditional practitioners and their personal beliefs and preferences, and ‘mediation’ by ancestral spirit ‘gatekeepers’ [32,33,46]. The ‘Doctrine of Signatures’ in zootherapy and similar symbolic ‘signatures’ for other traditional practices can broadly help infer the potential uses and benefits of specific species [42] (although these inferences are not always conclusive). While the cultural practices involving carnivores used by one ethnic group may not always exhibit cultural synonymy with those of other ethnic groups, some larger geographical patterns of use were identified from the newspapers and video footage – for example, the use of lion skins and parts by the ethnonymous Nguni/Ngoni Kingdoms from Tanzania to Eswatini and South Africa appears similar and interconnected, demonstrating a level of cultural continuity that reflects a shared cultural heritage and mutual influences across these regions.

The scale of demand for products appears aggregately significant but was not always explicitly addressed or fully captured in all the published research reviewed, typically and understandably due to the varying foci of these studies. Established networks of suppliers and cross-border trade appear common for some species, but records are rarely able to list the source populations – partly due to the focus of each study and the inherent challenges of tracing supply chains and trade dynamics (e.g., [32,54]). The markets for carnivore products vary, from local to regional and international, depending on location and purpose. Markets closer to supply sources may support domestic trade but may also link with intermediaries who then sell the products to other markets and consumers further away from the resource catchments. These products are traded in both open markets and through individual procurement, which may involve specialist procurers, hunters and middlemen. Broad geographic- and cross-cultural comparisons thus requires independent, context-specific investigations tailored to each user group, purpose, market, and species. This underscores the complex and fragmented mosaic of the available information. It highlights the need for progressively more consolidation and focussed research on the purpose, pathways, and trade scales for products across species and countries.

### Cultural use of focal species

The focal species are widely utilised for diverse purposes. In particular, we focused on examining the skin and parts incorporated into attire worn by traditional leaders, tribespersons, religious congregants, and healers, in diverse customary events, rituals, investitures, initiations, ceremonies and festivals that take place across the continent (Figs 8 and 9; S6 and S7 Tables). Spotted skins from larger and medium-sized felids tend to be favoured more than those of plainer or smaller species, which can partly be attributed to their unique and visually attractive pelage that holds significant aesthetic, ancestral and cultural value for ceremonial or symbolic purposes. Through analysis of video footage, we observed the widespread use of leopard, other spotted carnivores, and lion in visually striking items of regalia. Observations of leopard skin products worn by higher-status individuals and dignitaries is quite commonly reported in newspaper and video records, largely due to their visibility and widespread significance to traditional leaders in ceremonial regalia. Traditional health practitioners across the continent use lion body parts in rituals and divination, mainly because they exemplify strength and fear [23,32,41], and their skins and claws are significant to Eastern and Southern African Kings (pers. obs., mainly via newspaper review and video footage).

The greater number of publication records for leopard and lion may not necessarily always correspond to proportionally higher levels of extraction, trade and use compared to smaller species like genets that have fewer published records. Indeed, video footage showing traditional attire seems to indicate that genet skins are worn more than lion in more cultural events, and in higher quantities, than indicated by the publication records (Figs 7A and 9A). We therefore suggest that potential risks to smaller (particularly spotted) carnivores posed by use in traditional regalia are likely to be routinely underestimated based on prior published literature. Hence, the consequences of customary use on medium and smaller-spotted species like servals, civets and genets should not be overlooked and underestimated. These smaller species also hold intrinsic cultural and subsistence value, notably for bushmeat [55] and skins incorporated in the attire of lower-status tribespersons (Fig 8) [56]. Furthermore, their skins are likely to serve as partial substitutes for larger and declining spotted felids (a potential factor in substitutions noted in Kenya by Torrents-Ticó et al. [33]). Considering the decline of large-carnivore populations across of much of the African continent [1], we urge vigilance for cascading impacts of availability on medium and small carnivores.

Annually, hundreds of thousands, if not millions, of people across Africa engage in daily customary practices and participate in events to express, live, and celebrate their cultural heritage. Assessment of these events is not without biases, and we are cognisant that we relied on those events that were more extensively documented. Nevertheless, video footage highlighted that traditional attire linked to cultural events have the potential to drive greater demand for these products, which in turn exerts more pressures on leopard, serval, genet, and, to an overall slightly lesser extent (but depending on the region), also on lion populations for this purpose. We observed that these species are also more likely to be worn in higher quantities at more calendrical cultural events in Eastern and Southern Africa, primarily from July to September (Fig 9; S6 Table). Cultural events in Zambia were particularly well documented, with over 60 seasonal ceremonies across the country [51] – thus making it a key location for investigating carnivore use in traditional attire, the associated trade networks, and the trialling of culturally sensitive conservation measures. Some conservation consideration has already been given to the use of skins in African traditional cultural ceremonies, particularly for the *Kuomboka* and Kufuluhela ceremonies in Zambia [56], and the Shembe Church of South Africa [40], whereby realistic synthetic skin alternatives (known as Heritage Furs) have been adopted with some success as part of Panthera’s Furs For Life initiative implemented in partnership with culturo-religious groups ([40,57]. This demonstrates the potential for continued observance of African spiritual and cultural traditions without unsustainable exploitation. However, we remain aware of the need for further context-specific research on complex cultural-spiritual use systems since the use of substitutes and synthetic alternatives, for example, may sometimes be viewed as taboo (especially if ancestral guidance is required in the selection of alternatives for traditional medicine, see [32]), and they are not automatically the more simple, acceptable, or conservation friendly options [54]. Thus, overcoming any hindrances related to the use of alternatives will require culturally sensitive research to be conducted, ideally with or by African investigators from these ethnic groups to address these cultural sensitivities.

The review findings indicated that conflict linked to use may be more widespread than previously considered, suggesting that HWC-related mortalities could be providing more body parts for cultural practices under certain circumstances. HWC is widely recognised as a threat to big cat populations, particularly when they encounter humans and livestock (e.g., [16,58,59]), and lion and leopard were most frequently documented in relation to this issue. In particular, lion mortality due to HWC was frequently reported in Eastern Africa, and it is plausible that lion body parts are routinely useable and tradeable by-products of these incidents [16,23,60,61].

### Trade

There is pervasive sub-Saharan intra-African trade in carnivore products, with published sources documenting mainly leopard and lion parts. However, regional trends in utilisation and trade indicate that lion parts may be more sought after than leopard and other carnivore parts in regions like West Africa, as shown in a 2024 study by Gerstenhaber et al. for Benin and Niger [12]. The animals and products listed in our review’s trade and trafficking records, seizure reports, and investigations (e.g., inventories of regional markets) did not always originate from the same country where the research was conducted or the incidents were reported (e.g., [12,32,62]). Therefore, the countries depicted in our review in the context of trade should be understood as documented sink countries and not necessarily always the actual sources of the animal products, unless otherwise specified. Nevertheless, the linked locations provide some insight into potential geospatial ‘hotspots’ and networks where higher levels of regional wildlife trade, trafficking and use activity might occur (such as southern and West Africa). These locations also provide the source regions that are the origins of the animals before they enter the trade networks. This geographic insight is essential for providing a more holistic view of the risks to carnivores within the cultural trade landscape, thereby informing threat assessments, policymaking, and the development of targeted measures to mitigate these risks.

While our review does not explicitly elaborate on species-country-use-specific carnivore trade (e.g., quantities of genets sold for bushmeat in West Africa, but see Gerstenhaber et al [12] for an investigation of carnivore products in West Africa), or differentiate between legal and illegal trade, it is important to acknowledge the intricate, nuanced layers surrounding these matters and the challenges with information gathering. Except for many of the arrest and confiscation records reviewed, most publications did not, for various valid reasons, explicitly address or ascertain the origins and legality of the product acquisitions and transactions. The presumption is, however, that use and trade is predominantly illegal according to statutory law and international regulations (particularly cross-border transactions), that illegal trade is generally underestimated and likely detrimental to affected populations in the harvesting catchments of certain regions, and that legal trade is not necessarily without negative consequences for carnivore conservation either.

### Customary practices, contention, and contemporary conservation challenges

The insights gained from the newspaper review, alongside published literature and video footage, indicate that the post-independence era of the last 40 to 50 years has witnessed a progressive resurgence, revival, and transformation of traditional practices and cultural expressions across Africa (VLW pers. obs. from the newspaper review; [2,63–70]), often involving wildlife use. Despite the suppression and erosion of cultural heritage and identity during colonial times [71–74], this revival reflects a deliberate effort to preserve traditions integral to the identity, history, beliefs, and values of diverse African communities [51,68,74–76]. Examples of this revival include festivals and ceremonies in Ethiopia and Zambia [51,68,77], and the restoration of certain Indigenous monarchies (e.g., in Uganda) [69,70,76,78,79]. These once-suppressed practices are being actively embraced by communities, and the repatriation of stolen artifacts of tangible cultural heritage [72,80,81] further symbolizes a reclamation of cultural heritage and an affirmation of post-colonial identity. However, the revival of these practices is occurring under changing socio-political and economic conditions, and some traditional cultural controls that once regulated species use have been lost or diminished in modern society; this erosion has led to more individualistic expressions of culture, as seen in the increased use of leopard skins by individuals who would not traditionally have been permitted to wear them under previously stricter cultural guidelines (N.S. Mbongwa, personal communication, December 10, 2024). This weakening of cultural principles has implications for species conservation, as the original responsibility embedded in these cultural norms and practices to preserve biodiversity and regulate use has also been diminished. Additionally, both revived and enduring customary practices face contemporary challenges, particularly in navigating current conservation laws and socio-economic pressures.

One significant manifestation of these challenges is the clash between traditional practices and contemporary wildlife regulations. These regulations often criminalise customary wildlife use involving the extraction, acquisition, and trade of certain bio-cultural resources (especially threatened and charismatic species, like large felids) without proper state permissions, such as hunting and possession permits. As a result, the sourcing and trade of species for these cultural practices are deemed illegal under statutory law and policy. This situation leads to persistent conflict and contention with customary law and traditional user rights [82,83], creating “*contested illegality*” with some stakeholders who believe that cultural practices should be exempt from regulations, particularly when these regulations are perceived as unfair and inequitable [13,84]. Furthermore, there is the issue of economic inequality that compels many community stakeholders to engage in IWT [2,7]. Irrespective of one’s worldviews on these matters, this regulatory barrier is likely to remain a contentious issue in the perpetuation of traditional practices involving wildlife, especially threatened species and larger carnivores. Therefore, it is essential to acknowledge the near-ubiquity of cultural wildlife uses in Africa, recognise species’ vulnerability to persistent offtake for these purposes, how the loss of cultural responsibility intersects with modern regulatory frameworks, and to consider the applicability of Western-based laws, policies and prohibitions [71,83] within various cultural contexts. This approach includes addressing conflicting interests and disputes by seeking to align modern conservation strategies with cultural practices that balance use and preservation, while also incentivising legal behaviour, stewardship, and sustainable wildlife management practices. These management practices could include community co-management approaches involving sharing State responsibilities with totemic- and taboo-based traditional African cultural wildlife management practices [2].

### Observation biases and reporting focus

A key finding of this review is that there is widespread and under-reported cultural utilisation of carnivores across the African continent. Insufficient research can stem from research biases that lead to data deficiencies and thus to: (i) certain geographic regions and larger and/or charismatic animals getting disproportionally more research attention, to the detriment of other regions and smaller species; (ii) incomplete and inaccurate predictions and assessments of extirpation risk and non-detriment findings, (iii) underestimation of the impacts of specific threats and drivers of population decline; (iv) underestimation of the impact and magnitude of trade; (v) inadvertent neglect and exclusions of species from management plans, policies and priorities; and (vi) imbalanced and inappropriate conservation interventions [85–94]. We found that all of the above appear to be true with this paucity of data on cultural practices. However, in addition to the “*researchability*” of a species (i.e., the ability to collect certain types of data on certain species in certain places at certain times [91]) and cultural practices, certain factors have to be overcome in order to conduct targeted research to address the identified data deficiencies, including country level research capacity, availability of sufficient research resources [91], and consent by certain stakeholders and communities to conduct the research.

A factor in recognising data deficiencies during this review is that while records from the reviewed sources provided dateable information on species, countries and categories of utilisation, a notable portion of the literature records varied in their data content and focus. They were often from once-off studies without a longitudinal element across multiple countries, or comparable quantitative data, making it challenging to consolidate and quantitatively estimate the absolute magnitude and range of pan-African utilisation. However, video footage evidence seemed to reduce the species-focus biases often seen in the peer-reviewed literature. An under-utilised information source, that we did not systematically employ in this study, that may help overcome some of the systemic observational biases in text-based publications in future is photographic records, as successfully demonstrated recently in the monitoring of carnivore hunting [95] and other web-scraping technology (e.g., [96,97].

Regarding species biases, smaller and less charismatic species received notably less coverage across all categories except for bushmeat consumption and tended to appear later in the publication records. Given the nature of the records contributing to this study and the methods used in compiling them, we cannot conclusively determine whether the predominance of large-bodied species reflects a genuine, real-world prevalence of cultural use and trade in these species over small-bodied ones. This result may also stem from biases in the scope and direction of data collection reflected in published sources. We suspect the latter as species size biases in the published literature have been noted across a range of reviews of carnivores and charismatic species (e.g., [85–89,91,92]). Nevertheless, we remain alert to the possibility that the results of this review *may* indicate widespread predominance of large-bodied species over small-bodied species for use in certain cultural practices and trade. However, we doubt that this is consistently the case.

Whilst it is important to remain cognisant of potential biases in the interpretation of the results, we reiterate the key finding of this review – which is that cultural use of carnivores, both large and small, is under-reported and on a spectrum of magnitude and impact. We thus concur with the statement of Strampelli et al. [89] that “*large parts of Africa remain under-represented in the literature, and opportunities exist for further research on most species and in most countries*”.

### Further research considerations

This review compiled evidence for the pan-African use and trade of products from the focal carnivore species, resulting in a robust geospatial inventory of incidence/presence records of what taxa are used for and where. The suite of taxa utilised is influenced by the unique cultural norms and customary practices that are peculiar to different tribes and ethnic groups in each country. While this review provides evidence of species use based on available records, it cannot account for potential data gaps (including due to bias) or undocumented trade networks. Therefore, the absence of evidence should not be construed as evidence of absence of cultural use in certain countries.

Data deficiencies and absence of evidence, however, presents opportunities to address gaps and biases in the existing evidence records. To this end, the following recommendations to guide future research are proposed: (i) enhance research and monitoring of under-reported and omitted species (e.g., canids and hyaenas), smaller carnivores, and cultural uses, particularly in overlooked and data deficient regions and source countries, to elucidate the threats and implications posed by human-mediated exploitation, and to predict extirpation risks; (ii) conduct more detailed studies of cultural ceremonies and user groups across Africa, with particular attention to quantifying the impacts on species; (iii) implement quantitative longitudinal studies on a wider range of markets and events (e.g., specific cultural ceremonies and festivals named in S6 Table), and species, ensuring that these studies are context-specific and that stakeholder communities are actively involved and provide consent; (iv) focus studies on specific user groups to determine which species can be substituted with other species or products, under certain conditions, and why, while also quantifying turnover rates; (v) explore the landscape of regulation, enforcement, legality, and notions of ‘fair usage’; and (vi) we advocate for a comprehensive future review that incorporates additional evidence, such as still photography analysis through online image scraping, and focused expert surveys, to complement the published evidence.

## Conclusion

Our study represents the most comprehensive compilation and analysis of the scale of pan-African carnivore use and trade within the context of cultural and customary practices. It reveals widespread uses of many carnivore species across multiple African countries and ethnic groups, and identifies a variety of culturally motivated factors as pervasive, yet often over-looked, under-reported, under-studied, and undervalued anthropogenic threats and demand drivers. Traditional/ceremonial attire emerged as a prominent driver of use and trade in certain regions, particularly for leopards and other species with spotted pelage. Given the significant implications, further research is warranted to fully understand the absolute impact on carnivore populations; this will help to inform appropriate conservation and mitigation measures that include cognizance of cultural-spiritual traditions while deterring the inadvertent endorsement and disguise of illicit activities that exacerbate over-exploitation.

Our focus on compiling evidence for the observed existence and geographical extent of carnivore trade identified key hotspots and multi-national links, thereby providing a base for understanding parts of the broader trade landscape. While delving into the details, nuances and impacts of species-, country-, and use-specific carnivore trade across Africa was beyond the scope of this paper, our findings underscore the necessity for such quantitative and qualitative research to capture the complexities and impacts of this trade and broaden the scope of anthropogenic threat assessments to carnivores. The specific inclusion of traditional use and linked trade networks is important, because existing risk appraisals and management plans may not be fully capturing the spectrum of potential detrimental impacts on these biocultural resources driven by this continent-wide trade.

## Supporting information

S1–S3 Appendix Supporting information on keywords, abridged reference list used in the review, and a list of links to 555 YouTube videos. (PDF)

S1–S2 Figs Maximum adult body mass relative to information sources. Additional supporting information on maximum adult body mass relative to the number of information sources per species per use category.
(PDF)

S1–S2 Maps Species maps. Full-size maps for 36 taxa, showing geospatial evidence for the cultural use and trade of carnivore species and morphospecies across Africa, relative to their occurrence in a country.
(PDF)

S1–S7 Tables Additional tabulated information. Supporting information on publications numbers, body mass, YouTube results, and a summary of observations and classifications of focal taxa incorporated into traditional attire.
(PDF)

## References

[1] Bodasing T. The decline of large carnivores in Africa and opportunities for change. Biological Conservation. 2022;274:109724. doi: 10.1016/j.biocon.2022.109724

[2] Kideghesho JR. The potentials of traditional African cultural practices in mitigating overexploitation of wildlife species and habitat loss: experience of Tanzania. International Journal of Biodiversity Science and Management. 2009;5 83–94.

[3] Challender D, MacMillan D. Poaching is more than an enforcement problem. Conservation Letters. 2014;7(5):484–94.

[4] Biggs D, Cooney R, Roe D, Dublin HT, Allan JR, Challender DWS, et al. Developing a theory of change for a community-based response to illegal wildlife trade. Conservation Biology. 2017;31(1):5–12. doi: 10.1111/cobi.12796 27411900

[5] Lunstrum E, Givá N. What drives commercial poaching? From poverty to economic inequality. Biological Conservation. 2020;245:108505.

[6] Ntuli H, Sundström A, Sjöstedt M, Muchapondwa E, Jagers S, Linell A. Understanding the drivers of subsistence poaching in the Great Limpopo Transfrontier Conservation Area: What matters for community wildlife conservation?. Ecology and Society. 2021;26(1):18.

[7] Wilson-Holt O, Roe D. Community-based approaches to tackling illegal wildlife trade - what works and how is it measured?. Frontiers in Conservation Science. 2021;2:765725.

[8] Costa J, Baez-Camargo C, Kassa S, Lugolobi R. The role of informal networks in promoting illegal wildlife trade: a qualitative analysis from Uganda. Trends in Organanized Crime. 2021;26(4):397–419. doi: 10.1007/s12117-021-09433-y

[9] Gamso J. Aiding animals: does foreign aid reduce wildlife crime? The Journal of Environment & Development. 2022;32(1):34–60. doi: 10.1177/10704965221134820

[10] Kuiper T, Altwegg R, Beale C, Carroll T, Dublin HT, Hauenstein S, et al. Drivers and facilitators of the illegal killing of elephants across 64 African sites. Proceedings of the Royal Society B. 2023;290(1990):20222270. doi: 10.1098/rspb.2022.2270 36629103 PMC9832558

[11] Mozer A, Prost S. An introduction to illegal wildlife trade and its effects on biodiversity and society. Forensic Science International: Animals and Environments. 2023;3:100064. doi: 10.1016/j.fsiae.2023.100064

[12] Gerstenhaber C, Ipavec A, Lapeyre V, Plowman C, Chabi-N’Diaye Y, Tevoedjre F, et al. Illegal wildlife trade: An analysis of carnivore products found in markets in Benin and Niger. Global Ecology and Conservation. 2024;51:e02880. doi: 10.1016/j.gecco.2024.e02880

[13] Hübschle A. Contested illegality: processing the trade prohibition of rhino horn. In: Becket J, Dewey M, editors. The Architecture of Illegal Markets. Oxford: Oxford University Press; 2017; pp 177–197.

[14] Knapp E, Peace N, Bechtel L. Poachers and poverty: assessing objective and subjective measures of poverty among illegal hunters outside Ruaha National Park, Tanzania. Conservation and Society. 2017;15(1):24–32.

[15] Kassa S, Baez-Camargo C, Costa J, Lugolobi R. Determinants and drivers of wildlife trafficking: A qualitative analysis in Uganda. *Journal of International Wildlife Law & Policy*. 2021;24(3-4):314–342.

[16] Arias M, Coals P, Ardiantiono, Powell J, Rizzolo JB, Ghoddousi A, et al. Reflecting on the role of human-felid conflict and ‘local’ use in big cat trade. *Cons**ervation* *Sci**ence and* *Prac**tice*. 2024; e13030.

[17] Woodroffe R. Predators and people: using human densities to interpret declines of large carnivores. Animal Conservation. 2000;3(2):165–73. doi: insert_doi_here

[18] Inskip C, Zimmermann A. Human-felid conflict: a review of patterns and priorities worldwide. Oryx. 2009;43(01):18. doi: 10.1017/s003060530899030x

[19] Balme G, Lindsey P, Swanepoel L, Hunter L. Failure of research to address the rangewide conservation needs of large carnivores: leopards in South Africa as a case study. Conservation Letters. 2014;7 3–11.

[20] Braczkowski A, Ruzo A, Sanchez F, Castagnino R, Brown C, Guynup S, et al. The ayahuasca tourism boom: An undervalued demand driver for jaguar body parts?. Conservation Science and Practice. 2019;1(12):e126.

[21] Nicholson S, Bauer H, Strampelli P, Sogbohossou E, Ikanda D, Tumenta PF, et al. *Panthera leo* (amended version of 2024 assessment). The IUCN Red List of Threatened Species 2024: e.T15951A266696959. Accessed on 12 November 2024.

[22] Dickman A, Johnson PJ, van Kesteren F, Macdonald DW. The moral basis for conservation: how is it affected by culture?. Frontiers in Ecology and the Environment. 2015;13(6):325–31. doi: 10.1890/140056

[23] Williams VL, Loveridge AJ, Newton DJ, Macdonald DW. Questionnaire survey of the pan-African trade in lion body parts. PLoS One. 2017;12(10):e0187060. doi: 10.1371/journal.pone.0187060 29073202 PMC5658145

[24] Williams VL, Loveridge AJ, Newton DJ, Macdonald DW. A roaring trade? The legal trade in *Panthera leo* bones from Africa to East-Southeast Asia. PLoS One. 2017;12(10):e0185996. doi: 10.1371/journal.pone.0185996 29065143 PMC5655489

[25] Coals P, Moorhouse T, D’Cruze N, Macdonald D, Loveridge A. Preferences for lion and tiger bone wines amongst the urban public in China and Vietnam. Journal for Nature Conservation. 2020;57:125874.

[26] Uddin N, Enoch S, Harihar A, Pickles RS, Hughes AC. Tigers at a crossroads: Shedding light on the role of Bangladesh in the illegal trade of this iconic big cat. Conservation Science and Practice. 2023;5(7):e12952.

[27] Marneweck CJ, Allen BL, Butler AR, Do Linh San E, Harris SN, Jensen AJ, et al. Middle‐out ecology: small carnivores as sentinels of global change. Mammal Review. 2022;52(4):471–9. doi: 10.1111/mam.12300

[28] Adeola MN. Importance of wild animals and their parts in the culture, religious festivals, and traditional medicine, of Nigeria. *Environ**mental* *Conserv**ation*. 1992;19: 125–34.

[29] Alves RRN, Pinto LCL, Barboza RRD, Souto WMS, Oliveira REMCC, Vieira WLS. A global overview of carnivores used in traditional medicines. In: Alves RRN, Rosa IL, editors. Animals in Traditional Folk Medicine: Implications for Conservation. Berlin Heidelberg: Springer-Verlag; 2013. pp. 171–206.

[30] Somerville K. Humans and lions: conflict, conservation and coexistence. Abingdon: Routledge; 2019.

[31] Val A, Porraz G, Texier P-J, Fisher JW, Parkington J. Human exploitation of nocturnal felines at Diepkloof Rock Shelter provides further evidence for symbolic behaviours during the Middle Stone Age. Scientific Reports. 2020;10(1):6424. doi: 10.1038/s41598-020-63250-x 32286396 PMC7156369

[32] Coals PGR, Mbongwa NS, Naude VN, Williams VL. Contemporary Cultural Trade of Lion Body Parts. Animals (Basel). 2022;12(22):3169. doi: 10.3390/ani12223169 36428396 PMC9686618

[33] Torrents-Ticó M, Fernández-Llamazares Á, Burgas D, Nasak JG, Cabeza M. Biocultural conflicts: understanding complex interconnections between a traditional ceremony and threatened carnivores in north Kenya. Oryx. 2022;57(4):435–44. doi: 10.1017/s0030605322000035

[34] Dheer A, Davidian E, Jacobs M, Ndorosa J, Straka T, Höner O. Emotions and cultural importance predict the acceptance of large carnivore management strategies by Maasai pastoralists. Frontiers in Conservation Science. 2021;6(2):691975. doi: 10.3389/fcosc.2021.691975

[35] Dueppen SA, Gokee C. Hunting on the margins of medieval West African states: a preliminary study of the zooarchaeological record at Diouboye, Senegal. Azania: Archaeological Research in Africa. 2014;49(3):354–85. doi: 10.1080/0067270x.2014.931628

[36] Morton F, Hitchcock R. Tswana hunting: Continuities and changes in the Transvaal and Kalahari after 1600. South African Historical Journal. 2013;66(3):418–39. doi: 10.1080/02582473.2013.855809

[37] McCall DF. The prevalence of lions: kings, deities and feline symbolism in Africa and elsewhere. *Paideuma*. 1973;Jan:130–145.

[38] Pickenpaugh TE. Symbols of rank, leadership, and power in traditional cultures. International Journal of Osteoarchaeology. 1997;7(5):525–41. doi: 10.1002/(sici)1099-1212(199709/10)7:5<525::aid-oa364>3.0.co;2-5

[39] South African Government Communications. Minister Blade Nzimande: Nazareth Baptist Church of South Africa Career Expo. 2023 Jul 29. Available from: https://www.gov.za/news/speeches/minister-blade-nzimande-nazareth-baptist-church-south-africa-career-expo-29-jul-2023

[40] Naude V, Balme G, Rogan M, Needham M, Whittington‐Jones G, Dickerson T. Longitudinal assessment of illegal leopard skin use in ceremonial regalia and acceptance of faux alternatives among followers of the Shembe Church, South Africa. Conservation Science and Practice. 2020;2(11):e289.

[41] Simelane T, Kerley G. Conservation implications of the use of vertebrates by Xhosa traditional healers in South Africa. South African Journal of Wildlife Research. 1998;28(4):121–6.

[42] Williams VL, Whiting MJ. A picture of health? Animal use and the Faraday traditional medicine market, South Africa. Journal of Ethnopharmacology. 2016;179 265–73. doi: 10.1016/j.jep.2015.12.024 26727647

[43] Nieman WA, Leslie AJ, Wilkinson A. Traditional medicinal animal use by Xhosa and Sotho communities in the Western Cape Province, South Africa. Journal of Ethnobiology and Ethnomedicine. 2019;15(1):34. doi: 10.1186/s13002-019-0311-6 31288841 PMC6617652

[44] Morris B. The Powers of Animals. An Ethnography. Abingdon: Routledge; 2020.

[45] Cunningham AB, Zondi AS. Use of animals for the commercial trade in traditional medicines. Unpublished Report: Pietermaritzburg: Institute of Natural Resources; 1991.

[46] Coals PGR, Williams VL, Benítez G, Chassagne F, Leonti M. Ethnopharmacology, ethnomedicine, and wildlife conservation. Journal of Ethnopharmacology. 2024;333:118399. doi: 10.1016/j.jep.2024.118399 38824978

[47] Tishkoff SA, Reed FA, Friedlaender FR, Ehret C, Ranciaro A, Froment A, et al. The genetic structure and history of Africans and African Americans. Science. 2009;324(5930):1035–44. doi: 10.1126/science.1172257 19407144 PMC2947357

[48] Eberhard DM, Simons GF, Fennig CD, editors. Ethnologue: Languages of the World. Twenty-sixth edition. Dallas, Texas: SIL International. 2023. Online version: https://www.ethnologue.com/insights/continents-most-indigenous-languages/

[49] Kingdon J, Hoffmann M, editors. The Mammals of Africa. V. Carnivores, Pangolins, Equids and Rhinoceroses. London: Bloomsbury; 2013.

[50] Kitchener AC, Breitenmoser-Würsten C, Eizirik E, Gentry A, Werdelin L, Wilting A, et al. A revised taxonomy of the Felidae. The final report of the Cat Classification Task Force of the IUCN/ SSC Cat Specialist Group. Cat News Special Issue. 2017; 11: 80 p.

[51] Mkandawire SB, Simooya SM, Monde PN. Zambian Culture: Harnessing Cultural Literacy with a Focus on Selected Myths and Taboos. Lusaka: UNZA Press, Lusaka; 2019.

[52] Nowell K, Jackson P. Wild Cats: Status Survey and Conservation Action Plan. Gland, Switzerland: IUCN; 1996.

[53] Stuart C, Stuart M. Stuarts‘field guide to mammals of southern Africa, including Angola, Zambia and Malawi. 5th ed. Cape Town: Struik; 2015.

[54] Coals P, Loveridge A, Kurian D, Williams V, Macdonald D, Ogden R. DART mass spectrometry as a potential tool for the differentiation of captive-bred and wild lion bones. Biodiversity and Conservation. 2021;30(7):1825–54. doi: 10.1007/s10531-021-02182-3

[55] Doughty HL, Karpanty SM, Wilbur HM. Local hunting of carnivores in forested Africa: a meta-analysis. Oryx. 2014;49(1):88–95. doi: 10.1017/s0030605314000179

[56] Dickerson T, Naude V. Exploratory Report: Kuomboka Ceremony, Mongu, Zambia, 25^th^ April–1^st^ May 2018. Unpublished Internal Report for Panthera; 2018.

[57] Panthera. Saving Spots. Tackling leopard poaching for ceremonial leopard skin trade in western Zambia. Submission Report for 2023 Herman Goldstein Awards. December 2023. Unpublished Report; 2023. Available from: https://popcenter.asu.edu/sites/default/files/saving_spots_hg_submission_panthera_final_for_pop_centre_portal.pdf

[58] Bauer H, Dickman A, Chapron G, Oriol-Cotterill A, Nicholson SK, Sillero-Zubiri C, et al. Threat analysis for more effective lion conservation. Oryx. 2020;56(1):108–15. doi: 10.1017/s0030605320000253

[59] Krafte Holland K, Larson LR, Powell RB. Characterizing conflict between humans and big cats *Panthera* spp: A systematic review of research trends and management opportunities. PLoS One. 2018;13(9):e0203877. doi: 10.1371/journal.pone.0203877 30226894 PMC6143230

[60] Coals P, Dickman A, Hunt J, Grau A, Mandisodza-Chikerema R, Ikanda D, et al. Commercially-driven lion part removal: What is the evidence from mortality records?. Global Ecology and Conservation. 2020;24:e01327. doi: 10.1016/j.gecco.2020.e01327

[61] Everatt KT, Kokes R, Lopez Pereira C. Evidence of a further emerging threat to lion conservation; targeted poaching for body parts. Biodiversity and Conservation. 2019;28(14):4099–114.

[62] Drouilly M, Horion R, Pryce-Fitchen K, Pickles R, Whittington-Jones G, Asamoah Boateng B. Wild cat species in flux: from tradition to trade in Ghanaian markets. Cat News. 2023;78:26–31.

[63] Anyinam C. Availability, accessibility, acceptability, and adaptability: four attributes of African ethno-medicine. Social Science and Medicine. 1987;25(7):803–11. doi: 10.1016/0277-9536(87)90038-4 3317889

[64] Balzar J. In Mali, lizard blood may fix what ails you; medicine: Traditional cures enjoy a comeback in some African lands where drugs are unaffordable. Los Angeles Times. 1995.

[65] Ooman B. We must now go back to our history; retraditionalisation in a Northern Province Chieftaincy. African Studies. 2000;59(1):71–95.

[66] Simbao R. Dialectics of dance and dress: the performative negotiation of Soli Girl Initiates (Moye) in Zambia. African Arts. 2010;43(3):64–85. doi: 10.1162/afar.2010.43.3.64

[67] Shange N. Shembe Religion’s Integration of African Traditional Religion and Christianity: a Sociological Study. MA thesis, Rhodes University. 2013.

[68] Bulcha M. Oromia’s Irreecha Festival – a revival of an ancient African culture – an attempt to understand and explain [Internet]. Advocacy for Oromia. 2015 Sep 27. https://advocacy4oromia.org/2015/09/27/oromias-irreecha-festival-a-revival-of-an-ancient-african-culture-an-attempt-to-understand-and-explain/

[69] Denisova TS. The role of traditional leaders in the political life of West Africa: the case of Ghana. In: Bondarenko DM, Kowalewski SA, Small DB (editors). The Evolution of Social Institutions: Interdisciplinary Perspectives. Publisher. 2020. pp 371–384.

[70] Baral A. Keeping culture clean: ‘nested redistribution’ as a path to moral redemption in Kampala (Uganda). Journal of Contemporary African Studies. 2023;70(1):1–7.

[71] Zivave W. De-coloniality and de-minoritization of indigenous cultural heritage in Africa: an exploration of Nambya religion. In: Barnabas SG, editor. Indigenous populations – perspectives from scholars and practitioners in contemporary times [Working Title]. IntechOpen; 2022. doi: 10.5772/intechopen.105727

[72] Ingelson A, Owosuyi I. Reviewing the experience with the repatriation of sacred ceremonial objects: a comparative legal analysis of Canada and South Africa. International Journal of Cultural Property. 2022;29:217–41.

[73] Arowolo DE. Dancing on a knife-edge: European colonisation of Africa and Nigeria’s cultural crisis. African Identities. 2022;22(2):430–46. doi: 10.1080/14725843.2022.2040422

[74] Chakunda CJ. Healing the African Cultural Heritage [Internet]. IQOQO. 2024 May 6. https://iqoqo.org/healing-the-african-cultural-heritage

[75] Van Kessel I, Oomen B. ‘One chief, one vote’: the revival of traditional authorities in post-apartheid South Africa. African Affairs. 1997;96(385):561–85.

[76] Thondhlana T, Machiridza L. Restoration and restitution of cultural heritage, the case of the Ndebele monarch: the post-colonial dilemma in Zimbabwe. Journal of African Cultural Heritage Studies. 2020;2(2):52–84.

[77] Zeit D. Ethiopia’s Oromo People Celebrate Once-Banned Festival. Agence France-Presse (English). 2010 Oct 23. https://advance.lexis.com/api/permalink/9939c156-6f56-436b-9148-b42d8f378e22/?context=1519360&identityprofileid=W7VP8K60346

[78] Wasswa H. King Of Ethnic Group Crowned; One Celebrant: ‘I Will Die Happy’. The Associated Press. 2013 Jul 31. https://advance.lexis.com/api/permalink/a2ad7594-2e9b-472b-9501-662a1657460e/?context=1519360&identityprofileid=W7VP8K60346

[79] Peterson DR. Introduction: heritage management in Colonial and contemporary Africa. In: Peterson DR, Gavua K, Rassool C (editors). The Politics of Heritage in Africa. Economies, Histories, and Infrastructure. Cambridge University Press, Cambridge. 2015. pp 1–36.

[80] Batt F. The repatriation of African heritage: shutting the door on the imperialist narrative. African Human Rights Yearbook. 2021;5:328–350.

[81] Martinet L. Longing, belonging and owning: How to untangle competing claims over colonial cultural objects? In: Fabri HR, Rosoux V, Donati A (editors). Representing the Absent. Nomos Verlagsgesellschaft mbH & Co. KG., Baden-Baden. 2020. pp 143–162 (chapter 6).

[82] Mkumbukwa AR. The evolution of wildlife conservation policies in Tanzania during the colonial and post-independence periods. Development Southern Africa. 2008;25(5):589–600. doi: 10.1080/03768350802447875

[83] Sifuna N. The future of traditional customary uses of wildlife in modern Africa: a case study of Kenya and Botswana. Advances in Anthropology. 2012;2(1):31–8.

[84] Dore A, Hübschle A, Batley M. Towards environmental restorative justice in South Africa: How to understand and address wildlife offences. The Palgrave Handbook of Environmental Restorative Justice. n.d.333–60.

[85] Brodie J. Is research effort allocated efficiently for conservation? Felidae as a global case study. Biodiversity and Conservation. 2009;18(10):2927–39.

[86] Macdonald DW, Loveridge AJ, Nowell K. *Dramatis personae*: an introduction to the wild felids. In: Macdonald DW, Loveridge AJ, editors. Biology and Conservation of Wild Felids. Oxford: Oxford University Press; 2010. pp 3–58.

[87] Brooke ZM, Bielby J, Nambiar K, Carbone C. Correlates of research effort in carnivores: body size, range size and diet matter. PLoS One. 2014;9(4):e93195. doi: 10.1371/journal.pone.0093195 24695422 PMC3973602

[88] Monsarrat S, Kerley G. Charismatic species of the past: biases in reporting of large mammals in historical written sources. Biological Conservation. 2018;223:68–75.

[89] Strampelli P, Campbell LA, Henschel P, Nicholson SK, Macdonald DW, Dickman AJ. Trends and biases in African large carnivore population assessments: identifying priorities and opportunities from a systematic review of two decades of research. PeerJ. 2022;10:e14354. doi: 10.7717/peerj.14354 36452072 PMC9703985

[90] Bland LM, Bielby J, Kearney S, Orme CDL, Watson JEM, Collen B. Toward reassessing data-deficient species. Conservation Biology. 2017;31(3):531–9. doi: 10.1111/cobi.12850 27696559

[91] dos Santos JW, Correia RA, Malhado ACM, Campos‐Silva JV, Teles D, Jepson P, et al. Drivers of taxonomic bias in conservation research: a global analysis of terrestrial mammals. Animal Conservation. 2020;23(6):679–88. doi: 10.1111/acv.12586

[92] Marneweck C, Butler A, Gigliotti L, Harris S, Jensen A, Muthersbaugh M. Shining the spotlight on small mammalian carnivores: global status and threats. Biological Conservation. 2021;255:109005.

[93] Wang Z-N, Yang L, Fan P-F, Zhang L. Species bias and spillover effects in scientific research on Carnivora in China. Zoological Research. 2021;42(3):354–61. doi: 10.24272/j.issn.2095-8137.2021.033 33998183 PMC8175954

[94] Hoffmann CF, Montgomery RA. Implications of taxonomic bias for human–carnivore conflict mitigation. Oryx. 2022;56(6):917–26. doi: 10.1017/s0030605321000582

[95] Muller J, Selier S, Drouilly M, Broadfield J, Leighton G, Amar A. The hunter and the hunted: using web‐sourced imagery to monitor leopard (*Panthera pardus pardus*) trophy hunting. Conservation Science and Practice. 2022;4(11):e12789. doi: 10.1111/csp2.12789

[96] Stringham OC, Toomes A, Kanishka AM, Mitchell L, Heinrich S, Ross JV, et al. A guide to using the internet to monitor and quantify the wildlife trade. Conservation Biology. 2021;35(4):1130–9. doi: 10.1111/cobi.13675 33277940

[97] Kulkarni R, Di Minin E. Towards automatic detection of wildlife trade using machine vision models. Biological Conservation. 2023;279:109924.

