# Supplementary material for: Pan-African review of cultural uses of carnivores
Source: PLoS One. 2025 Mar 25;20(3):e0315903. doi: 10.1371/journal.pone.0315903 (PMC11936259; doi:10.1371/journal.pone.0315903)
Supplement: S1–S3 Appendix — (PDF) [file pone.0315903.s001.pdf]

**Pan-African review of cultural uses of carnivores**

Vivienne L. Williams, Marine Drouilly, Peter Coals, Gareth Whittington-Jones
(PLOS ONE)

# Supporting Information

# Appendix: S1 to S3

## Table of Contents:

**S1A APPENDIX. NEXIS KEYWORDS** .......... 1

**S1B APPENDIX. NEXIS EXCLUSION TERMS** .......... 2

**S2 APPENDIX. LITERATURE REVIEWED** .......... 3

**S3 APPENDIX. YOUTUBE VIDEOS WATCHED** .......... 16

**S1A Appendix.** The terms used to search Nexis® Uni in 2021 for news articles on carnivore uses in Africa.

**AND**: *(bushmeat OR "bush meat" OR muthi OR muti OR zootherapy OR fetish OR medicine OR medicinal OR attire OR regalia OR clothing OR decorative OR amulet OR curio OR trinket OR tourist OR ritual OR ceremony OR ceremonial OR status OR religious OR culture OR cultural OR initiation OR craft OR divination OR church OR heal OR chief OR traditional OR tradition OR spiritual OR skin OR fur OR souvenir OR claw OR meat OR food OR witch OR magic OR magique OR medico OR market OR trade OR urine OR fat OR organ OR brain OR pelt OR hunt OR poach OR trap OR kill OR snare OR hat OR spirit OR ailment OR witchcraft OR shembe OR diviner OR sangoma OR hide OR teeth OR tooth OR bone OR skeleton OR carcass OR skeleton OR dress OR "internal organ") AND (Acinonyx OR cheetah OR Nandinia OR Civettictis OR Viverridae OR civet OR Genetta OR genet OR Panthera OR leopard OR lion OR Leptailurus OR serval OR caracal OR "honey badger" OR Mellivora OR ratel OR zorilla OR weasel OR polecat OR wildcat OR "wild cat" OR Felis OR Mustela) AND (Africa OR Afrique OR Algeria OR Angola OR Benin OR Bénin OR Botswana OR "Burkina Faso" OR Burundi OR Cameroon OR Cameroun OR "Central African Republic" OR Chad OR Congo OR "Côte d'Ivoire" OR Djibouti OR Egypt OR Eritrea OR Eswatini OR Ethiopia OR Gabon OR Gambia OR Ghana OR Guinea OR Guinée OR "Ivory Coast" OR Kenya OR Lesotho OR Liberia OR Libya OR Malawi OR Mali OR Mauritania OR Morocco OR Mozambique OR Namibia OR Niger OR "République Centrafricaine" OR Rwanda OR Senegal OR Sierra Leone OR Somalia OR "South Africa" OR Swaziland OR Sudan OR Tanzania OR Togo OR Tunisia OR Uganda OR Zaire OR Zambia OR Zimbabwe OR Rhodesia)*

**S1B Appendix.** The exclusion terms used to filter and reduce the Nexis® Uni search results in 2021 for news articles on carnivore uses in Africa.

**AND NOT:** *(rugby OR theatre OR soccer OR cricket OR "world cup" OR championship OR tennis OR kaizer OR aviation OR petroleum OR genkor OR mintek OR qualifier OR comic OR album OR chocolate OR baseball OR basketball OR hockey OR speedway OR astrology OR mining OR genocide OR telephone OR supertanker OR nuclear OR godola OR cookhouse OR barnum OR actress OR actor OR coetsee OR bentsen OR snow OR klerk OR mutiny OR panama OR iraq OR Nicaragua OR pyramid OR sphinx OR OPEC OR rally OR pharaoh OR "misa luba" OR wilbur OR aspinall OR israel OR quarter-final OR semi-final OR champions OR league OR first-leg OR yacht OR Sasol OR alcatel OR lucent OR bandwidth OR telecom OR Vodacom OR Telkom OR seacom OR penmarch OR submarine OR toamasina OR offshore OR etisalat OR globacom OR unitel OR cellulaire OR tunisiana OR millicom OR sonatrach OR Bechtel OR broadband OR mcel OR "BMI" OR "logistics risk" OR armscor OR sabmiller OR castle OR AGOA OR oil-rich OR nampower OR Eskom OR zesco OR kengen OR hydroelectric OR escom OR tanesco OR alwaleed OR hemp OR webbox OR AFRIMA OR qalaa OR Vatican OR convair OR parenthood OR tullow OR Amstel OR capex OR "MTN" OR Wimax OR livebox OR grandprix OR gridiron OR wimax OR football OR gallman OR Texaco OR Hemmingway OR Kwagga OR gordimer OR lonrho OR mutola OR cabernet OR jihad OR Kiwanis OR shebeen OR wrestling OR lager OR Disney OR breweries OR golf OR smoking OR paperback OR brewer OR batsman OR equestrian OR bosnia OR emmy OR dreamworks OR cinema OR movie OR shipowner OR obituary OR castle OR ferry OR lottery OR "trade union" OR mosquito OR Judea OR Syria OR Manchester OR temple OR ASX OR bushel OR "conde nast" OR "british lion" OR ytl OR avis OR "general motors" OR Suzuki OR hotel OR crusader)*

**S2 Appendix.** Abridged list of journal (peer-reviewed) and non-journal (reports, books, dissertations, theses, newsletters, etc.) articles used to conduct the review and analyses. This list excludes newspaper articles from Nexis© Uni and other online sources.

| **First author_initials** | Year | Title | Journal or source | Volume |
|---|---|---|---|---|
| Abebe_YD | 2003 | Sustainable utilisation of the African Civet (*Civettictis civetta*) in Ethiopia. | Conference/symposium | |
| Acha_A | 2017 | Wildlife depletion and Its Associated Factors in Chebera-Churchura National Park, South Ethiopia | *World Journal of Zoology* | 12 |
| Adeola_MO | 1987 | Utilization of Wildlife Resources in Nigeria | Thesis - PhD | |
| Adeola_MO | 1992 | Importance of wild animals and their parts in the culture, religious festivals, and traditional medicine of Nigeria | *Environmental Conservation* | 19 |
| Aebischer_T | 2020 | Apex predators decline after an influx of pastoralists in former Central African Republic hunting zones | *Biological Conservation* | 241 |
| Afolayan_TA | 1980 | A synopsis of wildlife conservation in Nigeria | *Environmental Conservation* | 7 |
| Ahmin_M | 2015 | Ictonyx libycus. The IUCN Red List of Threatened Species 2015: e.T41645A45212347 | Red List - global | |
| Ajagun_EJ | 2017 | A survey of the traditional medical and non-medical uses of animal species and parts of the indigenous people of Ogbomoso, Oyo State | *International Journal of Herbal Medicine* | 5 |
| Ajari_SS | 1979 | Utilization of Forest Wildlife in West Africa | Report | |
| Akani_GC | 2015 | Are mammal communities occurring at a regional scale reliably represented in "hub" bushmeat markets? A case study with Bayelsa State (Niger Delta, Nigeria) | *Folia Zool.* | 64 |
| Al Safi_A | 2006 | Traditional Sudanese Medicine. A Primer for health care providers, researchers and students | Book | |
| Allebone-Webb_SM | 2011 | Use of market data to assess bushmeat hunting sustainability in Equatorial Guinea | *Conservation Biology* | 25 |
| Alves_RRN | 2013 | A global overview of carnivores used in traditional medicine | Book chapter | Chapter 9 |
| Amadi_N | 2015 | Distribution, habitat ecology and conservation status of the Two-spotted Palm Civet *Nandinia binotata* (Carnivora, Nandiniidae) in south-eastern Nigeria | *Small Carnivore Conservation* | 52 & 53 |
| Amir_OG | 2006 | Wildlife trade in Somalia | Report | |
| Anadu_PA | 1988 | The bushmeat trade in Southwestern Nigeria: a case study | *Human Ecology* | 16 |
| Angelici_FM | 1999 | Bushmen and mammal-fauna: a survey of the mammals traded in bush-meat markets of local people in the rainforests of South-eastern Nigeria. | *Anthropozoologica* | 30 |
| Angelici_FM | 2016 | *Genetta maculata*. The IUCN Red List of Threatened Species 2016: e.T41699A45218948 | Red List - global | |
| Anthony_BP | 2007 | Importance value of landscapes, flora and fauna to Tsonga communities in the rural areas of Limpopo province, South Africa | *South African Journal of Science* | 103 |
| Anthony_BP | 2010 | Sitting on the fence? Policies and practices in managing human-wildlife conflict in Limpopo Province, South Africa | *Conservation & Society* | 8 |

| | | | | |
|---|---|---|---|---|
| Anyinam_C | 1995 | Ecology and ethnomedicine: exploring links between current environmental crisis and indigenous medical practices | *Social Science and Medicine* | 40 |
| APPF | 2009 | Projet d'étude des Grands Félins et de leur Environnement Naturel et Humain (PFNH) | Report | |
| Asibey_EOA | 1970 | The present status of wildlife conservation in Ghana | Conference/symposium | |
| Asibey_EOA | 1974 | Wildlife as a source of protein in Africa south of the Sahara | *Biological Conservation* | 6 |
| Asibey_EOA | 1977 | Expected effects of land-use patterns on future supplies of bushmeat in Africa south of the Sahara | *Environmental Conservation* | 4 |
| Avenant_NL | 2016 | A conservation assessment of *Caracal caracal*. In The Red List of Mammals of South Africa, Swaziland and Lesotho: Child et al. | Red List - ZA | |
| BA_CO | 2006 | The economic value of wild resources in Senegal. A preliminary investigation of non-timber forest products, game and freshwater fisheries | Report | |
| Bahaa-el-din_L | 2013 | Notes on the distribution status of small carnivores in Gabon | *Small Carnivore Conservation* | 48 |
| Bahaa-el-Din_L | 2015 | The African golden cat *Caracal aurata*: Africa's least-known felid | *Mammal Review* | 45 |
| Bahaa-el-din_L | 2015 | Caracal aurata. The IUCN Red List of Threatened Species 2015: e.T18306A50663128 | Red List - global | |
| Bahaa-el-Din_L | 2015 | Ecology and Conservation of the African Golden Cat *Caracal aurata* | Thesis - PhD | |
| Barnett_R | 2000 | Food for thought: the utilization of wild meat in Eastern and Southern Africa. TRAFFIC East/Southern Africa | Report | |
| Bauer_H | 2016 | *Panthera leo*. The IUCN Red List of Threatened Species 2016: e.T15951A115130419 | Red List - global | |
| Begg_CM | 2016 | A conservation assessment of *Mellivora capensis*. In The Red List of Mammals of South Africa, Swaziland and Lesotho: Child et al. | Red List - ZA | |
| Beinart | 1990 | Empire, hunting and ecological change in southern and central Africa | *Past and Present* | 128 |
| Beninguisse_G | 2004 | Tradition and modernity in Cameroon: the confrontation between social demand and biomedical logics of health services | *African Journal of Reproductive Health* | 8 |
| Bergin_D | 2014 | Illegal and open wildlife trade in Morocco's capital | *SWARA* | July-September |
| Bergin_D | 2014 | Open, unregulated trade in wildlife in Morocco's markets | *TRAFFIC Bulletin* | 26 |
| Bergin_D | 2015 | Potential benefits of impending Moroccan wildlife trade laws, a case study in carnivore skins | *Biodiversity and Conservation* | ? |
| Bersacola_E | 2014 | Hunted in Angola: surveying the bushmeat trade | *SWARA* | Jan-Mar |
| Bird_TLF | 2013 | A preliminary survey of the presence and distribution of small carnivores in the Lower Zambezi Protected Area Complex, Zambia | *Small Carnivore Conservation* | 48 |
| Bizimana_S | 2018 | Le culte de Ryangombe au Rwanda - étude ethnographique | Book | |
| Bobo_KS | 2010 | Mammals and birds for cultural purposes and related conservation practices in the Korup area, Cameroon | *Life Sciences Leaflets* | 9 |
| Bobo_KS | 2011 | Sacred plants and animals in the Batoufam and Bansoa communities in West Cameroon | *Life Sciences Leaflets* | 18 |
| Bobo_KS | 2014 | Wildlife use and the role of taboos in the conservation of wildlife around the Nkwende Hills Forest Reserve; South-west Cameroon | *Journal of Ethnobiology and Ethnomedicine* | 11 |
| Born Free | 2008 | Too much pressure to handle? Lion derivatives used in traditional medicine in Nigeria, West Africa | Report | |

| | | | | |
|---|---|---|---|---|
| Bowland_JM | 1990 | Diet, home range and movement patters of serval in farmland in Natal | Dissertation MSc | |
| Braga_Pereira_F | 2017 | First record of Angola's medicinal animals: a case study on the use of mammals in local medicine in Quicama National Park | *Indian Journal of Traditional Knowledge* | 16 |
| Bryant_ | 1966 | Zulu medicine and medicine men | Book | |
| Burton_AC | 2011 | The decline of lions in Ghana's Mole National Park | *African Journal of Ecology* | 49 |
| Butynski_TM | 2012 | Identification, distribution and conservation status of the African golden cat *Caracal aurata* in Kenya | *Journal of East African Natural History* | 101 |
| Carpeneto_GM | 1989 | The mammals in the zoological culture of the Mbuti pygmies in north-eastern Zaire | *Hystrix* | 1 |
| Carpeneto_GM | 1989 | Mustelidae and Viverridae from north-eastern Zaire ethnozoological research and conservation | *Small Carnivore Conservation* | 1 |
| Carvalho_F | 2016 | A conservation assessment of *Genetta genetta*. In The Red List of Mammals of South Africa, Swaziland and Lesotho: Child et al. | Red List - ZA | |
| Carvalho_M | 2015 | What motivates hunters to target exotic or endemic species on the island of Sao Tome? | *Oryx* | 49 |
| Chabi-N'Diaye_Y | 2014 | Exploitation et commerce des sous-produits de grands carnivores dans les communes proches de la Réserve de Biosphère de la Pendjari | BSc Hons thesis | |
| Chabi-N'Diaye_Y | 2014 | Utilisation et commerce des sous-produits de grands carnivores dans les communs périphériques de la Reserve de Biosphere de la Pendjari | Report | |
| Chauque_MD | 2010 | Analysis of merchantability or animal parts thereof for medical purposes in the city of Maputo | Report | |
| Child_GS | 1970 | Wildlife utilization and its relevance in West Africa | Conference/symposium | |
| Child_MF | 2016 | A conservation assessment of *Poecilogale albinucha*. In The Red List of Mammals of South Africa, Swaziland and Lesotho: Child et al. | Red List - ZA | |
| CITES | 2002 | Implementation of Decision 11.165 on trade in traditional medicines. List of species traded for medicinal purposes: AC18 Doc. 13.1 | CITES Annex | |
| CITES | 2014 | Illegal trade in cheetahs. SC64 Doc. 39 (Rev 2) | CITES Doc | |
| Coals_PG | 2020 | Commercially-driven lion part removal: what is the evidence from mortality records? | *Global Ecology and Conservation* | 24 |
| Cocks_M | 2002 | Use of indigenous and indigenised medicines to enhance personal well-being: a South African case study | *Social Science and Medicine* | 54 |
| Cole Burton_AC | 2011 | The decline of lions in Ghana's Mole National Park | *African Journal of Ecology* | 49 |
| Cole Burton_AC | 2011 | Evaluating persistence and its predictors in a West African carnivore community | *Biological Conservation* | 144 |
| Colyn_M | 2004 | The importance of small carnivores in forest bushmeat hunting in the Classified Forest of Diecke, Guinea | *Small Carnivore Conservation* | 31 |
| Cory_H | 1946 | The Buyeye: a secret society of snake-charmers in Sukumaland, Tanganyika Territory | *Africa: Journal of the International African Institute* | 16 |
| Cory_H | 1949 | The ingredients of magic medicines | *Africa: Journal of the International African Institute* | 19 |
| Crump_CM | 2001 | Need to assess animals used by traditional healers in South Africa | Report | |
| Cunningham_AB | 1991 | Use of animal parts for the commercial trade in traditional medicines | Report | |

| | | | | |
|---|---|---|---|---|
| Cunningham_AB | 1992 | Striped weasels. Traditional medicine and conservation. | *Endangered Wildlife* | 11 |
| Cunningham_AB | 1992 | Striped weasels, traditional medicines and conservation | Working paper | |
| D'Cruze_N | 2020 | Snake oil and pangolin scales: insights into wild animal use at "Marche des Fétiches" traditional medicine market, Togo | *Nature Conservation* | 39 |
| Danino_D | 2009 | Little Ethiopia: an ethnopharmacological study of the Ethiopian community in Israel | *International Journal of Ethiopian Studies* | IV |
| Davenport_TRB | c.2000 | Observations on the bushmeat exploitation and ecology of the African golden cat (*Profelis aurata*, Temminck) in south east Cameroon | *Not cited* | |
| de Luca_DW | 2013 | Small carnivores of the Mt Rungwe-Kitulo landscape, southwest Tanzania: presence, distributions and threats | *Small Carnivore Conservation* | 48 |
| de Merode_E | 2004 | The value of bushmeat and other wild foods to rural households living in extreme poverty in Democratic Republic of Congo | *Biological Conservation* | 118 |
| de Smet_K | 2003 | Cheetah in and around Hoggar National Park in Central Sahara (Algeria) | *CATnews* | 38 |
| Dedeke_GA | 2005 | Pilot survey of ethnozoological utilisation of vertebrates in southwestern Nigeria | *Indilinga African Journal of Indigenous Knowledge Systems* | 5 |
| Dedeke_GA | 2006 | Ethnozoological trade and practices among the Ijebu people of south-western Nigeria and the impact on some mammalian species | *Indilinga African Journal of Indigenous Knowledge Systems* | 5 |
| Des Bois_R | 2013 to 2019 | [No specific article title. Any information on the focal taxa were extracted using a keyword search] | On the Trail | Vol 1–28 |
| Dickman_A | 2015 | The moral basis for conservation: how it is affected by culture | *Front. Ecol. Environ.* | 13 |
| Dillon-Mallone_C | 1988 | Mutumwa Nchimi healers and wizardry beliefs in Zambia | *Social Science and Medicine* | 26 |
| Djagoun_CAMS | 2009 | Small carnivorans from southern Benin: a preliminary assessment of diversity and hunting pressure | *Small Carnivore Conservation* | 40 |
| Djagoun_CAMS | 2013 | Wild mammals trade for zootherapeutic and mythic purposes in Benin (West Africa): capitalizing species involved, provision sources, and implications for conservation | Book chapter | Chapter 17 |
| Djame_FN | 2018 | Droit et politique de l'environnement au Cameroun - Afin de faire de l'Afrique l'arbre de vie. Chapitre 22 "La protection de la faune en droit Camerounais" | Book chapter | Chapter 22 |
| Do Linh San_E | 2013 | Conservation status, distribution and species richness of small carnivores in Africa | *Small Carnivore Conservation* | 48 |
| Do Linh San_E | 2016 | *Mellivora capensis*. The IUCN Red List of Threatened Species 2016: e.T41629A45210107 | Red List - global | |
| Do Linh San_E | 2019 | *Civettictis civetta* (amended version of 2015 assessment). The IUCN Red List of Threatened Species 2019: e.T41695A147992107 | Red List - global | |
| Doughty_HL | 2014 | Local hunting of carnivores in forested Africa: a meta-analysis | *Oryx* | 49 |
| Duonamou_L | 2020 | Temporal evolution of bushmeat traded in High Niger National Park, Guinea, West Africa | *Oryx* | |
| Durant_S | 2015 | Acinonyx jubatus. The IUCN Red List of Threatened Species 2015: e.T219A50649567. | Red List - global | |
| EAGLE Network | 2015 to 2020 | The EAGLE Network: Annual Report 2015 to 2020 | EAGLE Network Report | 6 volumes |

| | | | | |
|---|---|---|---|---|
| Edwards_IB | 2003 | The fetish markets and animal parts trade of Mali, West Africa: an ethnographic investigation into cultural use and significance | Dissertation MA | |
| El-Kamali_HH | 2000 | Folk medicinal use of some animal products in Central Sudan | *Journal of Ethnopharmacology* | 72 |
| Eniang_EA | 2008 | Bush meat trading in the Oban Hills region of South-Eastern Nigeria: implications for sustainable livelihoods and conservation | *Ethiopian Journal of Environmental Studies and Management* | 1 |
| Ethiopian Wildlife Authority | 2012 | National action plan for the conservation of the African Lion *Panthera leo* in Ethiopia | Report | |
| Everatt_KT | 2019 | Evidence of a further emerging threat to lion conservation; targeted poaching for body parts | *Biodiversity and Conservation* | 29 |
| Fa_JE | 1995 | Impact of market hunting on mammal species in Equatorial Guinea | *Conservation Biology* | 9 |
| Fa_JE | 2002 | Bushmeat consumption and preferences of two ethnic groups in Bioko Island, West Africa | *Human Ecology* | 30 |
| Fa_JE | 2005 | Hunting vulnerability, ecological characteristics and harvest rates of bushmeat species in afrotropical forests | *Biological Conservation* | 121 |
| Fa_JE | 2006 | Getting to grips with the magnitude of exploitation: bushmeat in the Cross-Sanaga rivers region, Nigeria and Cameroon | *Biological Conservation* | 129 |
| Feely_JM | 2012 | isiXhosa name of leopard | *African Zoology* | 47 |
| Fischer_C | 2013 | Diversity and distribution of small carnivores in a Miombo woodland within the Katavi region, Western Tanzania | *Small Carnivore Conservation* | 48 |
| Fogg_W | 1938 | A tribal market in the Spanish Zone of Morocco | *Africa: Journal of the International African Institute* | 11 |
| Fuashi_NA | 2019 | An evaluation of poaching and bushmeat off takes in the Ebo Forest Reserve (EFR), Littoral Region, Cameroon | *Journal of Ecology and the Natural Environment* | 11 |
| Gandiwa_E | 2013 | Illegal hunting and law enforcement during a period of economic decline in Zimbabwe: A case study of northern Gonarezhou National Park and adjacent areas | *Journal for Nature Conservation* | 21 |
| Gandiwe_E | 2011 | Preliminary assessment of illegal hunting by communities adjacent to the northern Gonarezhou National Park, Zimbabwe | *Tropical Conservation Science* | 4 |
| Gaubert_P | 2015 | Bushmeat genetics: setting up a reference framework for the DNA typing of African forest bushmeat | *Molecular Ecology* | 15 |
| Gaubert_P | 2015 | *Genetta bourloni*. The IUCN Red List of Threatened Species 2015: e.T136223A45220931 | Red List - global | |
| Gaubert_P | 2015 | *Genetta cristata*. The IUCN Red List of Threatened Species 2015: e.T8998A45198406 | Red List - global | |
| Gaubert_P | 2015 | *Genetta* genetta. The IUCN Red List of Threatened Species 2015: e.T41698A45218636 | Red List - global | |
| Gaubert_P | 2015 | *Genetta piscivora*. The IUCN Red List of Threatened Species 2015: e.T15628A45201673 | Red List - global | |
| Gaubert_P | 2015 | *Genetta poensis*. The IUCN Red List of Threatened Species 2015: e.T136435A45221269 | Red List - global | |
| Gaubert_P | 2015 | *Genetta thierryi*. The IUCN Red List of Threatened Species 2015: e.T41701A45219325 | Red List - global | |
| Gaubert_P | 2015 | Genetta tigrina. The IUCN Red List of Threatened Species 2015: e.T41702A45219459 | Red List - global | |
| Gaubert_P | 2015 | *Nandinia binotata*. The IUCN Red List of Threatened Species 2015: e.T41589A45204645 | Red List - global | |
| Gaubert_P | 2015 | *Poiana richardsonii*. The IUCN Red List of Threatened Species 2015: e.T41704A45219609 | Red List - global | |

| | | | | |
|---|---|---|---|---|
| Gaubert_P | 2015 | *Poiana leightoni*. The IUCN Red List of Threatened. Species 2015: e.T44165A45220840 | Red List - global | |
| Gaubert_P | 2016 | *Genetta abyssinica*. The IUCN Red List of Threatened Species 2016: e.T8994A45198149 | Red List - global | |
| Gaubert_P | 2016 | *Genetta angolensis*. The IUCN Red List of Threatened Species 2016: e.T41696A45218468 | Red List - global | |
| Gaubert_P | 2016 | *Genetta johnstoni*. The IUCN Red List of Threatened Species 2016: e.T8997A45198265 | Red List - global | |
| Gaubert_P | 2016 | *Genetta pardina*. The IUCN Red List of Threatened Species 2016: e.T136437A45221360 | Red List - global | |
| Gaubert_P | 2016 | *Genetta servalina*. The IUCN Red List of Threatened Species 2016: e.T41700A97163789 | Red List - global | |
| Gaubert_P | 2016 | *Genetta victoriae*. The IUCN Red List of Threatened Species 2016: e.T41703A45219531 | Red List - global | |
| Gbogbo_F | 2019 | Trade in wildlife for traditional medicine in Ghana: therapeutic values, zoonoses considerations, and implications for biodiversity conservation | *Human Dimensions of Wildlife* | 24 |
| Gebresenbet_F | 2018 | Beyond the numbers: Human attitudes and conflict with lions (*Panthera leo*) in and around Gambella National Park, Ethiopia | *PLOS ONE* | 13 |
| Gelfand_M | 1955 | The nganga of Mashonaland | *The Central African Journal of Medicine* | 1 |
| Gerstenhaber_C | 2021 | Illegal wildlife trade: An analysis of carnivore products found in markets in Benin and Niger | MSc Thesis | |
| Goldman_MJ | 2010 | Maintaining complex relations with large cats: Maasai and Lions in Kenya and Tanzania | *Human Dimensions of Wildlife* | 15 |
| Goldman_MJ | 2013 | Beyond ritual and economics: Maasai lion hunting and conservation politics | *Oryx* | 47 |
| Groom_R | 2018 | Quiçama National Park Angola. A large and medium sized mammals survey. [Version for review] INBAC/RWCP | Report | |
| Hamissou_G | 2000 | Collecte et analyse de données pour l'management durable des forets - joindre les efforts nationaux et internationaux OR Les produits Forestiers Non-Ligneux au Niger Connaissances actuelles et tendances | Report | |
| Harries_P | 1993 | Imagery, symbolism and tradition in a South African Bantustan: Mangosuthu Buthelezi, Inkatha, and Zulu History | *History and Theory* | 32 |
| Haule_KS | 2002 | Striving for sustainable wildlife management: the case of Kilombero Game Controlled Area, Tanzania | *Journal of Environmental Management* | 66 |
| Henschel_P | 2003 | Leopards in African rainforests: survey and monitoring techniques | Report | |
| Henschel_P | 2010 | Lion status updates from five range countries in West and Central Africa | *CATnews* | 52 |
| Henschel_P | 2012 | Improving the status of lions and cheetahs in their last stronghold in West Africa: the W-Arly-Pendjari Complex. Preliminary Report, November 2012 | Report | |
| Henschel_P | 2019 | Countering big cat trafficking in Senegal. Panthera report | Report | |
| Herbst_M | 2016 | A conservation assessment of Felis silvestris. In The Red List of Mammals of South Africa, Swaziland and Lesotho: Child et al. | Red List - ZA | |
| Hewat_ML | 19xx | Bantu Folklore | Book | |
| Hoffmann_M | 2015 | A review of evidence for the presence of Two-spotted Palm Civet *Nandinia binotata* and four other small carnivores on Bioko, Equatorial Guinea | *Small Carnivore Conservation* | 52 & 53 |
| Houpline_C | 2010 | Lion d'Afrique de l'Ouest: statut de conservation en Guinée | *CEPA Magazine* | 22 |

| | | | | |
|---|---|---|---|---|
| Huntley_BJ | 2019 | Biodiversity of Angola. Science & Conservation: a Modern Synthesis. Springer | Book | |
| Ikanda_DK | 2008 | Non-detriment report under CITES regarding the export of African Lions *Panthera leo* from the United Republic of Tanzania | Report | |
| Ingram_DJ | 2019 | Characterising trafficking and trade of pangolins in the Gulf of Guinea | *Global Ecology and Conservation* | 17 |
| Ipavec_A | 2018 | Illegal wildlife trade in Benin. NCC training course | Presentation | |
| IUCN SSC Cat Specialist Group | 2006 | Conservation strategy for the lion in West and Central Africa | Report | |
| IUCN SSC Cat Specialist group | 2006 | Stratégie de conservation du lion en Afrique de l'Ouest et du Centre | Report | |
| Jacobson_AP | 2016 | Leopard (*Panthera pardus*) status, distribution, and the research efforts across its range | *PeerJ* | 4 |
| Jambiya_G | 2007 | Night Time Spinach': Conservation and livelihood implications of wild meat use in refugee situations in north-western Tanzania. TRAFFIC East/Southern Africa, Dar es Salaam, Tanzania. | Report | |
| Jeffrey_S | 1977 | How Liberia uses wildlife | *Oryx* | ? |
| Jorge_AA | 2012 | Costs and benefits of the presence of leopards to the sport-hunting industry and local communities in Niassa National Reserve, Mozambique | *Conservation Biology* | 27 |
| Juste_J | 1995 | Market dynamics of bushmeat species in Equatorial Guinea | *Journal of Applied Ecology* | 32 |
| Kamatensei-Mugisha_M | 2007 | Medicinal plants used to induce labour during childbirth in western Uganda | *Journal of Ethnopharmacology* | 109 |
| Kaschula_SA | 2009 | Quantity and significance of wild meat off-take by a rural community in the Eastern Cape, South Africa | *Environmental Conservation* | 36 |
| Kelleher_S | 2020 | Comprendre les menaces qui pèsent sur la biodiversité en Afrique de l'Ouest et les liens avec le trafic des espèces sauvages - Rapport de la mission d'évaluation pour le Bénin | Government Report | |
| Kendie_FA | 2018 | Ethnozoological study of traditional medicinal appreciation of animals and their products among the indigenous people of Metema Woreda, North-Western Ethiopia | *Journal of Ethnobiology and Ethnomedicine* | 14 |
| Khalid_HS | 2007 | Trade of Sudanese natural medicines and their role in human and wildlife healthcare | *Cropwatch Newsletter* | |
| Kideghesho_JR | 2008 | Co-existence between the traditional societies and wildlife in western Serengeti, Tanzania: its relevancy in contemporary wildlife conservation efforts | *Biodiversity & Conservation* | 17 |
| Kiffner_C | 2009 | Plenty of prey | *Oryx* | 43 |
| Kingdon_J | 2013 | Mammals of Africa | Book | |
| Kunzel_T | 2000 | Status assessment of wildlife in Djibouti. Final report | Report | |
| Kuste_J | 1995 | Market dynamics of bushmeat species in Equatorial Guinea | *Journal of Applied Ecology* | 32 |
| LAGA | 2012 | GALF Project. Wildlife Law Enforcement in Guinea. March - November 2012 report | Report | |
| Lindsey_P | 2012 | Illegal hunting and the bush-meat trade in savanna Africa: drivers, impacts and solutions to address the problem | Report | |
| Lindsey_P | 2012 | Illegal hunting and the bushmeat trade in central Mozambique: a case study from Coutada 9, Manica Province | Report | |

| | | | | |
|---|---|---|---|---|
| Lindsey_PA | 2013 | The bushmeat trade in African savannas: Impacts, drivers, and possible solutions | *Biological Conservation* | 160 |
| Loeb_EM | 1949 | The Kuanyama Ambo and other tribes of South West Africa | *Anthropos* | 41/44 |
| Lyth_RE | 1947 | The Suri tribe | *Sudan Notes and Records* | 28 |
| Mabaso_XX | 2019 | Under the skin of a culture: perceptions of fake leopard skin alternatives in the Shembe Baptist Church | Dissertation MSc | |
| Macdonald_DW | 2011 | Association of body mass with price of bushmeat in Nigeria and Cameroon | *Conservation Biology* | 25 |
| Maisels_F | 2001 | The extirpation of large mammals and implications for montane forest conservation: the case of the Kilum-Ijim Forest, North-west Province, Cameroon | *Oryx* | 35 |
| Mallon_DP | 2015 | An IUCN situation analysis of terrestrial and freshwater fauna in West and Central Africa | Report | |
| Manqele_NS | 2017 | Assessing the drivers and impact of illegal hunting for bushmeat and trade on serval (*Leptailurus serval*, Schreber 1776) and oribi (Ourebia ourebi, Zimmermann 1783) in South Africa. | Dissertation MSc | |
| Manqele_NS | 2018 | Drivers of the illegal hunting of serval (*Leptailurus serval*) and oribi (*Ourebia ourebi*) in the KwaZulu-Natal Midlands, South Africa | *African Journal of Wildlife Research* | 48 |
| Marshall_NT | 1998 | Searching for a cure: conservation of medicinal wildlife resources in East and Southern Africa | Report | |
| Martin_E | 2010 | The status if the retail ivory trade in Addis Ababa in 2009 | *TRAFFIC Bulletin* | 22 |
| Martin_GHG | 1983 | Bushmeat in Nigeria as a natural resource with environmental implications | *Environmental Conservation* | 10 |
| Martin_RB | 1988 | Survey of the Status and Distribution of Leopard in Sub-Saharan Africa. Lausanne: CITES Secretariat. | Report | |
| Mathot_L | 2012 | The leopards of Gabon, the national team's symbol in danger | *Wildlife Justice* | 8 |
| Mayr_Fr | 1907 | The Zulu Kafirs of Natal V. Clothing and ornaments (continued) | *Anthropos* | 2 |
| Mazzocchetti_F | 2005 | Utilisations et Représentations de la Faune Sauvage (tachetée) chez les Bakota de la région de Makokou (Gabon) | Dissertation MSc | |
| McDonald_RA | 2019 | *Mustela nivalis* (amended version of 2016 assessment). The IUCN Red List of Threatened Species 2019: e.T70207409A147993366 | Red List - global | |
| McMillen_HL | 2008 | Conserving the roots of trade: local ecological knowledge of ethnomedicines from Tanga, Tanzania markets | Thesis - PhD | |
| Mesochina_P | 2010 | Conservation status of the lion (*Panthera leo* Linnaeus, 1758) in Tanzania | Report | |
| Mfonu_VG | 2006 | Lion skin dealer arrested in the north | *Wildlife Justice* | 6 |
| Milledge_S | 2005 | A model for Africa: Ethiopia's efforts to close unregulated domestic ivory markets in Addis Ababa | *TRAFFIC Bulletin* | 20 |
| Miller_S | 2016 | A conservation assessment of *Panthera leo*. In The Red List of Mammals of South Africa, Swaziland and Lesotho: Child et al. | Red List - ZA | |
| Mills_D | 2012 | Systematic survey efforts of the African golden cat Ð Part 2. Results from Uganda | *CATnews* | 57 |
| Mogharo_MM | 2009 | A Survey of Queen Elizabeth National Park (QENP) Communities’ Attitudes Toward Human-Lion Conflict and Lion Conservation. PhD Dissertation. George Mason University, Fairfax, VA, USA | Thesis - PhD | |
| Moore_J | 1985 | Chimpanzee survey in Mali, West Africa | *Primate Conservation* | 6 |
| Moore_JF | 2018 | First records of the Central African oyan (*Poiana richardsonii*) in Rwanda | *African Journal of Ecology* | 56 |

| | | | | |
|---|---|---|---|---|
| Morris_B | 1986 | Herbalism and divination in southern Malawi | *Social Science and Medicine* | 23 |
| Morris_B | 1998 | The powers of nature | *Anthropology & Medicine* | 5 |
| Morris_B | 1998 | The Power of Animals: an Ethnography | Book | |
| Mulder_MB | 2019 | Lions, Bylaws, and Conservation Metrics | *BioScience* | 69 |
| Myers_N | 1971 | The spotted cats: in danger? - or in danger of danger? | Conference/symposium | |
| Myers_N | 1975 | The Cheetah *Acinonyx jubatus* in Africa - Report of a survey in Africa from the Sahara southwards. [4], 1-90. Morges, Switzerland, IUCN. IUCN Monographs. | Report | |
| Myers_N | 1976 | The Leopard *Panthera pardus* in Africa. IUCN Monograph No. 5. | Monograph | |
| Mzimela_T | 1995 | The muthi business | *Indicator SA* | 12 |
| Nadembega_P | 2011 | Medicinal plants in Baskoure, Kourittenga Province, Burkina Faso: An ethnobotanical study | *Journal of Ethnopharmacology* | 133 |
| Naude_VN | 2020 | Longitudinal assessment of illegal leopard skin use in ceremonial regalia and acceptance of faux alternatives among followers of the Shembe Church, South Africa | *Conservation Science and Practice* | |
| Nevadomsky_J | 1988 | Kemwin-Kemwin: the apothecary shop in Benin City | *African Arts* | 22 |
| Ngoufo_R | 2014 | Social norms and cultural services - community belief system and use of wildlife products in the Northern periphery of the Korup National Park, South-West Cameroon | *Change Adaptation Socioecological System* | 1 |
| Ngwenya_MP | 2001 | Implications of the medicinal animal trade for nature conservation in KwaZulu-Natal | Report | |
| Nielsen_MR | 2006 | Importance, cause and effect of bushmeat hunting in the Udzungwa Mountains, Tanzania: Implications for community-based wildlife management | *Biological Conservation* | 128 |
| Nielson_MR | 2014 | How could the bushmeat trade in the Kilombero Valley of Tanzania be regulated? Insights from the rural value chain | *Oryx* | |
| Nieman_WA | 2019 | Traditional medicinal animal use by Xhosa and Sotho communities in the Western Cape Province, South Africa | *Journal of Ethnobiology and Ethnomedicine* | 15 |
| Nijman_V | 2019 | Illegal wildlife trade Ð surveying open animal markets and online platforms to understand the poaching of wild cats | *Biodiversity* | |
| Nimir_MB | 1983 | Wildlife Values and Management in Northern Sudan. Ph.D. Thesis | Thesis - PhD | |
| Noss_AJ | 1998 | Cable snares and bushmeat markets in a central African forest | *Environmental Conservation* | 25 |
| Noumi_E | 2004 | Animal and plant poisons and their antidotes in Eseka and Mbalmayo regions, Centre Province, Cameroon | *Journal of Ethnopharmacology* | 93 |
| Nowell_K | 1996 | Status survey and conservation action plan. Wild Cats. IUCN/SSC Cat Specialist Group | Report | |
| Nowell_K | 2014 | An assessment of conservation impacts of legal and illegal trade in cheetahs *Acinonyx jubatus*. SC65 Doc. 39 (Rev 2) Annex 1 | CITES Doc | |
| Ntiamoa-Badu_Y | 1987 | West African wildlife: a resource in jeopardy | Report | |
| Nyrop_RF | 1969 | Area Handbook for Rwanda | Book | Vol 550 |
| Oduntan_OO | 2012 | Survey of wild animals used in zootherapy at Ibadan, Oyo State, Nigeria | *International Journal of Molecular Zoology* | 2 |

| | | | | |
|---|---|---|---|---|
| Olayemi_A | 2011 | Contribution of DNA-typing to bushmeat surveys: assessment of a roadside market in south-western Nigeria | *Wildlife Research* | 38 |
| Olupot_W | 2009 | An analysis of socioeconomics of bushmeat hunting at major hunting sites in Uganda | Report | |
| Ondoua_GO | 2017 | An assessment of poaching and wildlife trafficking in the Garamba-Bili-Chinko transboundary landscape | Report | |
| Onuegbu_GC | 2020 | Biotic homogenisation in the Niger Delta (Nigeria): Evidence from small carnivores in bushmeat markets | *African Journal of Ecology* | 58 |
| Oomen_B | 2000 | "We must now go back to our history"; Retraditionalisation in a Northern Province Chieftaincy | *African Studies* | 59 |
| Overton_J | 2017 | The illegal bushmeat trade in the Greater Kafue Ecosystem, Zambia Drivers, impacts and potential solutions. FAO/Department of National Parks and Wildlife/Panthera/Game Rangers International, Zambia. | Report | |
| Papini_R | 2004 | Dance uniform history in the church of Nazareth Baptists: the move to tradition | *African Arts* | 27 |
| Pellerin_M | 2009 | Statut de conservation du lion (*Panthera leo* Linnaeus, 1758) au Benin | Report | |
| Pescheux_G | 2003 | Le Royaume Asante (Ghana): parenté, pouvoir, histoire, XVIIe-XXe siècles | Book | |
| Plug_I | 2009 | Ethnography and southern African archaeozoology | Book chapter | |
| Prins_FE | 1994 | Expressions of fertility in the rock art of the Bantu-speaking agriculturalists | *The African Archaeological Review* | 12 |
| Pujol_J | 1999 | NaturAfrica. The herbalist handbook | Book | |
| Ramesh_T | 2016 | A conservation assessment of *Leptailurus serval*. In The Red List of Mammals of South Africa, Swaziland and Lesotho: Child et al. | Red List - ZA | |
| Ray_JC | 2005 | Setting Conservation Priorities for Larger African Carnivores. WCS Working Paper No. 24. WCS, New York | Working paper | |
| Republic of Zambia | 2009 | Zambia's conservation strategy and action plan for the African Lion | Report | |
| Rogan_MS | 2015 | Illegal Bushmeat hunting in the Okavango Delta, Botswana: Drivers, Impacts and potential solutions. FAO/Panthera/Botswana predator conservation trust, Harare. | Report | |
| Rossi_A | 2018 | Uganda wildlife trafficking assessment | Report | |
| Roux_R | 2016 | A conservation assessment of Genetta maculata | Red List - ZA | |
| Roux_R | 2016 | A conservation assessment of *Genetta maculata*. In The Red List of Mammals of South Africa, Swaziland and Lesotho: Child et al. | Red List - ZA | |
| Rovero_F | 2012 | Hunting or habitat degradation? Decline of primate populations in Udzungwa Mountains, Tanzania: An analysis of threats | *Biological Conservation* | 146 |
| Rowe-Rowe_D | 2016 | A conservation assessment of *Ictonyx striatus*. In The Red List of Mammals of South Africa, Swaziland and Lesotho: Child et al. | Red List - ZA | |
| Rowe-Rowe_DT | 1990 | The African weasel: a Red Data Book species in South Africa | *Small Carnivore Conservation* | 90 |
| Rowe-Rowe_DT | 1992 | The carnivores of Natal | Report | |
| RWCP & IUCN/SSC | 2015 | Regional Conservation Strategy for the Cheetah and African Wild Dog in Southern Africa; Revised and Updated, August 2015 | Report | |
| Sa_RMM | 2013 | The trade and ethnobiological use of chimpanzee body parts in Guinea-Bissau: implications for conservation | *TRAFFIC Bulletin* | 24 |
| Sayer_JA | 1984 | The distribution and status of large mammals in Benin | *Mammal Review* | 14 |

| | | | | |
|---|---|---|---|---|
| Segniagbeto_GH | 2013 | Reptiles traded in the fetish market of Lomé, Togo (West Africa) | *Herpetological Conservation and Biology* | 8 |
| Shipp_A | 2002 | Wildlife for sale in Marrakech, Morocco. | *TRAFFIC Bulletin* | 19 |
| Sifuna_N | 2012 | The future of traditional customary uses of wildlife in modern Africa: a case study of Kenya and Botswana | *Advances in Anthropology* | 2 |
| Simelane_T | 2011 | Are traditionally used resources within conservation areas a function of their sizes? | *Natural Resources* | 2 |
| Simelane_TS | 1996 | | Thesis - PhD | |
| Simelane_TS | 1998 | Conservation implications of the use of vertebrates by Xhosa traditional healers in South Africa | *South African Journal of Wildlife Research* | 28 |
| Sliwa_A | 2016 | Felis nigripes. The IUCN Red List of Threatened Species 2016: e.T8542A50652196. | Red List - global | |
| Sodeinde_OA | 1999 | Pilot study of the traditional medicine trade in Nigeria | *TRAFFIC Bulletin* | 18 |
| Sodi_T | 2011 | Indigenous healing practices in Limpopo Province of South Africa: A qualitative study | *International Journal of Health Promotion and Education* | 49 |
| Soewu_DA | 2008 | Wild animals in ethnozoological practices among the Yorubas of south-western Nigeria and the implications for biodiversity conservation | *African Journal of Agricultural Research* | 3 |
| Soewu_DA | 2012 | Trade in wild mammalian species for traditional medicine in Ogun State, Nigeria | *Global Journal of Medical Research* | 12 |
| Soewu_DA | 2013 | Zootherapy and biodiversity conservation in Nigeria | Book chapter | Chapter 16 |
| Sogbohossou_EA | 2006 | Conservation des grands carnivores en Afrique de l'Ouest: perception par les populations et commerce des sous-produits rapport final | Report | |
| Stein_AB | 2020 | *Panthera pardus* (amended version of 2019 assessment). The IUCN Red List of Threatened Species 2020: e.T15954A163991139 | Red List - global | |
| Stuart_C | 2015 | *Poecilogale albinucha*. The IUCN Red List of Threatened Species 2015: e.T41662A45215258 | Red List - global | |
| Sugwara_K | 2001 | Cognitive space concerning habitual thought and practice toward animals among the central san (\|gui and \|\|gana): deictic/indirect cognition and prospective/retrospective intention | *African Study Monographs, Suppl* | 27 |
| Swanepoel_LH | 2016 | A conservation assessment of *Genetta tigrina*. In The Red List of Mammals of South Africa, Swaziland and Lesotho: Child et al. | Red List - ZA | |
| Swanepoel_LH | 2016 | A conservation assessment of *Panthera pardus*. In The Red List of Mammals of South Africa, Swaziland and Lesotho: Child et al. | Red List - ZA | |
| Tah_EK | 2012 | Regional wildlife enforcement: a vision for Central Africa | *Wildlife Justice* | 7 |
| Taye_T | 2008 | The African civet cat (*Viverra civetta*) and its life supporting role in the livelihood of smallholder farmers in Ethiopia | Conference/symposium | |
| Tchibozo_S | 2004 | Animaux médicinaux du Bénin: des drogues anciennes toujours actuelles. | *Bulletin de Liaison de l'Association des Amis du Musée de la Pharmacie* | 29 |
| Tee_TN | 2012 | Bush meat trade in Makurdi Metropolis; implications for the conservation of wildlife in Nigeria | *Journal of Applied Biosciences* | 52 |
| Tehou_CA | 2013 | Plan d'actions pour la conservation du lion au Benin. MEHU/CENAGREF. | Report | |
| Tchibozo_S | 2004 | Animaux médicinaux du Bénin: des drogues anciennes toujours actuelles | *Bulletin* | |

| | | | | |
|---|---|---|---|---|
| Thibault_M | 2003 | The oil industry as an underlying factor in the bushmeat crisis in Central Africa | *Conservation Biology* | 17 |
| Thiel_C | 2011 | Ecology and population status of the Serval *Leptailurus serval* (Schreber, 1776) in Zambia. | Thesis - PhD | |
| Thiel_C | 2019 | *Leptailurus serval* (amended version of 2015 assessment). The IUCN Red List of Threatened Species 2019: e.T11638A156536762 | Red List - global | |
| Thompson_EE | 1965 | Primitive African medical lore and witchcraft | *Bull Med Libr Assoc* | 53 |
| Tricorache_P | 2017 | Pets and pelts: understanding and combating poaching and trafficking in cheetahs | Book chapter | |
| van Bockhaven_V | 2009 | Leopard-men of the Congo in literature and popular imagination | *Tydskrif vir Letterkunde* | 46 |
| van Mensch_PJA | 1969 | On the African golden cat, *Profelis aurata* (Temminck, 1827) | *Extrait de la revue Biologia Gabonica* | 5 |
| van Vliet_N | 2019 | From the forest to the dish: a comprehensive study of the wild meat value chain in Yangambi, Democratic Republic of Congo | *Frontiers in Ecology and Evolution* | 7 |
| Vats_R | 2015 | A study on use of animals as traditional medicine by Sekuma tribe of Busega district in North-western Tanzania | *Journal of Ethnobiology and Ethnomedicine* | 11 |
| Vincent_JF | 1986 | Afrique plurielle, Afrique actuelle: hommage à Georges Balandier. Chapitre "L'œil de la panthère sied au chef" | Book chapter | |
| Walsh_M | 1998 | Mammals in Usangu: miscellaneous notes from Utengule, 1980-81. 1st draft, December 1998 | Working paper | |
| Warchol_GL | 2004 | The transnational illegal wildlife trade | *Criminal Justice Studies* | 17 |
| Waterland_S | 2015 | Illegal wildlife trade review Malawi May 2015 | Report | |
| West_H | 2001 | Sorcery of construction and socialist modernization: ways of understanding power in postcolonial Mozambique | *American Ethnologist* | 28 |
| White_RM | 2004 | People and forest fauna: a case study from coastal dune forest in the Transkei region of the Eastern Cape | Book chapter | |
| White_RM | 2004 | Traditional medicines from forest animals | Book chapter | |
| Whiting_JW | 2011 | Animals traded for traditional medicine at the Faraday market in South Africa: species diversity and conservation implications | *Journal of Zoology* | 284 |
| Widdows_CD | 2016 | A conservation assessment of *Civettictis civetta*. In The Red List of Mammals of South Africa, Swaziland and Lesotho: Child et al. | Red List - ZA | |
| Wilkie_DS | 1989 | Impact of roadside agriculture on subsistence hunting in the Ituri forest of northeastern Zaire | *American Journal of Physical Anthropology* | 78 |
| Wilkie_DS | 1992 | Mechanized logging, market hunting, and bank loan in Congo | *Conservation Biology* | 6 |
| Willcox_AS | 2007 | Wildlife hunting practices and bushmeat dynamics of the Banyangi and Mbo people of Southwestern Cameroon | *Biological Conservation* | 134 |
| Williams_VL | 1995 | The Witwatersrand muti trade | *Veld & Flora* | March |
| Williams_VL | 2016 | A picture of health? Animal use and the Faraday traditional medicine market, South Africa | *Journal of Ethnopharmacology* | 179 |
| Williams_VL | 2017 | Questionnaire survey of the pan-African trade in lion body parts | *PLOS ONE* | 12 |

| | | | | |
|---|---|---|---|---|
| Wilson_B | 2016 | A conservation assessment of *Felis negripes*. In The Red List of Mammals of South Africa, Swaziland and Lesotho: Child et al. | Red List - ZA | |
| Wright_JH | 2010 | Hunting and trapping in Lebialem Division, Cameroon: bushmeat harvesting practices and human reliance | *Endangered Species Research* | 11 |
| Yalden_DW | 1980 | Catalogue of the mammals of Ethiopia | *Monitore Zoologico Italiano* | Sup 13 |
| Yamaguchi_N | 2015 | *Felis silvestris*. The IUCN Red List of Threatened Species 2015: e.T60354712A50652361 | Red List - global | |
| Yohannes_DW | 2014 | Ethnozoological study of traditional medicinal animals used by the Kore people in Amaro Woreda, Southern Ethiopia | *International Journal of Molecular Evolution and Biodiversity* | 4 |
| Zambia Wildlife Authority | 2009 | Zambia's conservation strategy and action plan for the African lion. Report: 1-16 | Report | |

**S3 Appendix.** List of 514 of the 555 YouTube reviewed for the study. Not all these videos yielded usable information for the review. Map showing the number of videos watched per country on the last page. *[Table excludes links to 41 YouTube videos for Francophone countries because their URLs were not saved, and none of these 41 videos contributed usable information for the review.]*

| Country | Video title | Link |
|---|---|---|
| **Africa** | Top 10 Best Traditional Dances in Africa - African Traditional Dances | https://www.youtube.com/watch?v=_KsbkwzD5-U&t |
| **Africa** | How Men Live With Lions... | https://www.youtube.com/watch?v=3nbu2GrU30U |
| **Africa** | Top 10 countries African that practice witchcraft | https://www.youtube.com/watch?v=izT9yrg23PM&t |
| **Angola** | Traditional Angolan dancing | https://www.youtube.com/watch?v=cCyPhuJqEcs |
| **Angola** | Women of the 'lost' tribes of Angola | https://www.youtube.com/watch?v=ikixaj1xvIE |
| **Angola** | Sassa Tchokwe, Muita dance tchianda | https://www.youtube.com/watch?v=OVsnXltIztM |
| **Angola** | The Mumuila tribe of Angola | https://www.youtube.com/watch?v=QZ-92mHHkgU |
| **Angola, DRC, Namibia, Zambia** | Makishi dance - Kanda unguveta | https://www.youtube.com/watch?v=MK0BNQSxv68 |
| **Angola, DRC, Namibia, Zambia** | Makishi dance - Neha Tala video | https://www.youtube.com/watch?v=PziFso3uw50 |
| **Angola, DRC, Namibia, Zambia** | Makishi dance - | https://www.youtube.com/watch?v=tOm9ndDPnEo |
| **Botswana** | Tswana \| Botswana traditional dance \| Kala Tsa Kgale | https://www.youtube.com/watch?v=-KeIUxGMHxw&t |
| **Botswana** | Tjilenje The Ngwao - Hosana Dance | https://www.youtube.com/watch?v=006eggsaPDg |
| **Botswana** | Traditional dance competition Botswana 2 | https://www.youtube.com/watch?v=3L7V4qBDPfw |
| **Botswana** | Kuru dance | https://www.youtube.com/watch?v=4nz_npVcxjU |
| **Botswana** | Maitisong Festival documentary | https://www.youtube.com/watch?v=4y9eiAywr0Q&t |
| **Botswana** | The beauty of Tswana wedding | https://www.youtube.com/watch?v=BFIJRTLTZuM&t |
| **Botswana** | Traditional dance competition Botswana 3 | https://www.youtube.com/watch?v=CIHs6D0kZ3A |
| **Botswana** | San Dance VR @Kuru Dance festival | https://www.youtube.com/watch?v=e4-tdvUTXVU |
| **Botswana** | Traditional dance competition Botswana 1 | https://www.youtube.com/watch?v=eHv7uqt5P8k&t |
| **Botswana** | Kuru dance festival vlog | https://www.youtube.com/watch?v=EkKzIaTmceM |
| **Botswana** | Traditional group \| Botswana \| Parakarungu | https://www.youtube.com/watch?v=FYWPTupTaUs |
| **Botswana** | Dikgafela 1981 - harvest celebrations | https://www.youtube.com/watch?v=IIPyz_CUdpI |
| **Botswana** | Botswana traditional Dance by Mbukushu tribe | https://www.youtube.com/watch?v=KJ6RU8UxVVY |

| | | |
|---|---|---|
| **Botswana** | Mbukushu of Botswana performing diware dance | https://www.youtube.com/watch?v=lmPFU19SZkI&t |
| **Botswana** | Dikopelo folk music of Bakgatla ba Kgafela in Kgatleng District | https://www.youtube.com/watch?v=nYWQFf_n3xU |
| **Botswana** | San Dance VR @Kuru Dance festival | https://www.youtube.com/watch?v=qFLsdF_hKxI |
| **Botswana** | Tjilenje the ngwao cultural dance group (Kamanyangwe) | https://www.youtube.com/watch?v=ufjiupH1pt8&t |
| **Botswana** | Botswana traditional dances | https://www.youtube.com/watch?v=wjfFZDrPmpA |
| **Botswana, Zimbabwe** | African Traditional Bakalanga Hhoso Dance | https://www.youtube.com/watch?v=qyW9m77HbQ8&t |
| **Burundi** | Twaguye gitumo wamupfumu ufite inkoko ivuga nkabantu \| Salongo nabibona arumirwa | https://www.youtube.com/watch?v=7Elr2trsJ9s |
| **Burundi** | private | https://www.youtube.com/watch?v=GsQUSKNoh2k |
| **Burundi** | Inkoko ivuga nkabantu itubwiye ibintu biteye ubwoba | https://www.youtube.com/watch?v=qiNDoRgklSQ |
| **Cameroon** | Dance Toupouri | https://www.youtube.com/watch?v=b8EVmmx-wYM&t |
| **Cameroon** | Yelli - Baka women yodellers | https://www.youtube.com/watch?v=cATZe_jlc9g |
| **Cameroon** | Baka pygmies performing the 'booma' dance | https://www.youtube.com/watch?v=IDYKuhwT2ww |
| **Cameroon** | Bangoulap, Funérailles d'un grand notable à l'Ouest Cameroun | https://www.youtube.com/watch?v=mppupbda1H4&t |
| **Cameroon** | Le toupouri mange chien. danse Gourna au festival | https://www.youtube.com/watch?v=Sy3KJ3GcVM8&t |
| **Cameroon** | Tupuri dancing | https://www.youtube.com/watch?v=wHSKDgpzvd8 |
| **Cameroon** | Zheu Dance | https://www.youtube.com/watch?v=ycEY8fXafQY&t |
| **CAR** | African Bayaka Pygmies Music Part 1 | https://www.youtube.com/watch?v=v8kobQSd8C8&t |
| **CAR** | Polyphonic Singing of the Aka Pygmies of Central Africa | https://www.youtube.com/watch?v=yKLxFmnYO_I |
| **Chad** | Chants e danse chrétiens tupuri du Tchad.AVI | https://www.youtube.com/watch?v=YuRqbOlV6H8&t |
| **Congo** | Mbuti pygmies of the rain forest | https://www.youtube.com/watch?v=-X5cD6jd914&t |
| **Congo** | Musique et danse Traditionnelle Région du Niari | https://www.youtube.com/watch?v=d3zbVyZNDIA |
| **Congo** | CULTURE TEKE CONGO BRAZZAVILLE | https://www.youtube.com/watch?v=ywEotJyAs24&t |
| **Congo / West Africa** | 'The soul of the Congolese': Rumba | https://www.youtube.com/watch?v=F2zaWwWSU_M&t |
| **DRC** | Ngoma Pende 04 | https://www.youtube.com/watch?v=-S6UKi88v0A |
| **DRC** | In the land of giants and pygmies 1925 | https://www.youtube.com/watch?v=4Hos9aZuPjE |
| **DRC** | Traditional Congolese Dance - Ballet Arumbaya Ndendeli | https://www.youtube.com/watch?v=5mMsXM-4USM&t |
| **DRC** | Spectacle de percussion traditionnelle congolaise par Ballet Arumbaya Ndendeli | https://www.youtube.com/watch?v=iV19EcfFUD8&t |

| | | |
|---|---|---|
| **DRC** | CONGO: KINSHASA: REBEL LEADER LAURENT KABILA IS WORN IN AS PRESIDENT | https://www.youtube.com/watch?v=KaAs-rYEjkQ |
| **DRC** | Tchokwe traditional song - Sheketa (Tchianda dance) | https://www.youtube.com/watch?v=Na3O6hjqcFI |
| **DRC** | Pende Dance Troupe 3 from Congo Kinshasa | https://www.youtube.com/watch?v=OZqjt4GkK00 |
| **DRC** | Tribu Mongo / Équateur DR Congo | https://www.youtube.com/watch?v=PCnBf0QJTzo |
| **DRC** | Folklore Mongo | https://www.youtube.com/watch?v=ty-ztcNhrsg&t |
| **DRC** | ba Mongo | https://www.youtube.com/watch?v=vsFT-TeUrP4 |
| **DRC** | Musique et Danse Traditionnelle " Département de la Lékoumou" Congo Bzv | https://www.youtube.com/watch?v=YZaann8qOAU&t |
| **East Africa** | Ngoni migration in East Africa | https://www.youtube.com/watch?v=5x7JNPlIekg |
| **East Africa** | Ujenzi wa Stand mpya mugumu | https://www.youtube.com/watch?v=6UWsXHonPRU&t |
| **Eswatini** | Swazi nation show excitement towards the sacred Incwala | https://www.youtube.com/watch?v=Db9-VeIz0Mc |
| **Eswatini** | Weeding royal fields | https://www.youtube.com/watch?v=HWta5eT5sbY&t |
| **Eswatini** | Warriors Incwala | https://www.youtube.com/watch?v=iA4v5wlxomA |
| **Eswatini** | Ingatja has been commissioned | https://www.youtube.com/watch?v=j-veus4Ezns&t |
| **Eswatini** | thousands of Tingatja regiments have arrived at Ngabezwini | https://www.youtube.com/watch?v=LL4uzuzWPWA |
| **Eswatini** | Maidens from South Africa at the Umhlanga reed dance in Swaziland | https://www.youtube.com/watch?v=MbVnWT5Mg3I&t |
| **Eswatini** | Kingdom of Swaziland Africa Inkanyezi Yezulu | https://www.youtube.com/watch?v=vuuGa330tns&t |
| **Ethiopia** | Dassench tribe dimi ceremony Omo valley Ethiopia | https://www.youtube.com/shorts/0UlI8dHGOIo |
| **Ethiopia** | Ethiopian Oromo's Irreecha festival | https://www.youtube.com/watch?v=25fNwxELEoo&t |
| **Ethiopia** | Oromo Protest after Irrecha Massacre | https://www.youtube.com/watch?v=2rEP5MwPRq4&t |
| **Ethiopia** | Ethiopia: full coverage of Irreecha celebration 2016 | https://www.youtube.com/watch?v=8xuuVIcfdVg |
| **Ethiopia** | Dassench Tribe DIMI CEREMONY | https://www.youtube.com/watch?v=EbL6qt-nWKo&t |
| **Ethiopia** | Ethiopian tribes traditional dance | https://www.youtube.com/watch?v=fVCJJE9DDWc |
| **Ethiopia** | Dassanech tribe | https://www.youtube.com/watch?v=GfXwUMinohE |
| **Ethiopia** | Donga January 2018 | https://www.youtube.com/watch?v=kGWA0Xl6vRg&t |
| **Ethiopia** | Dassenech village - Omorate - Ethiopia | https://www.youtube.com/watch?v=kXPtwCNNnrA&t |
| **Ethiopia** | Hamar tribe, market day, Ethiopia | https://www.youtube.com/watch?v=mXulS_C3w10 |
| **Ethiopia** | Ethiopia's capital hosts Oromo cultural event | https://www.youtube.com/watch?v=N-lTUBHCIv0 |
| **Ethiopia** | Ethiopia's capital hosts Oromo cultural event | https://www.youtube.com/watch?v=PCNxgEFSJbE |

| | | |
|---|---|---|
| **Ethiopia** | Surma . Donga, Ethiopia | https://www.youtube.com/watch?v=TxmQ4MUewKc |
| **Ethiopia** | Ethiopia: The Dimi rite of the Dasanech tribe. | https://www.youtube.com/watch?v=uCgSdyXfFDw&t |
| **Ethiopia** | Ethiopia's Irrecha festival stuns fans with its vibrant colours | https://www.youtube.com/watch?v=V05YU6RfHCE |
| **Ethiopia** | Dassanech tribe | https://www.youtube.com/watch?v=ybzlWWJaVrw |
| **Ethiopia** | Surma tribe - near Kibish, Omo Valley, Ethiopia | https://www.youtube.com/watch?v=YfR39xQ0VWo |
| **Ethiopia** | Fichee-Chambalaalla, New Year festival of the Sidama people | https://www.youtube.com/watch?v=zsmAzpxBOJY&t |
| **Gabon** | Ceremonie bwiti a moughenda village | https://www.youtube.com/watch?v=-3oXMLWJVUE |
| **Gabon** | Bwete Gabon, sortie mwengue nganga | https://www.youtube.com/watch?v=2kKTTwiBlNE&t |
| **Gabon** | DSC 0075 | https://www.youtube.com/watch?v=3ouVSk-_ENk |
| **Gabon** | Ya kendo | https://www.youtube.com/watch?v=9gFFv37bt-I |
| **Gabon** | Cérémonie Niembé | https://www.youtube.com/watch?v=a_YEOZbPvdE |
| **Gabon** | Spectacle danse traditionelle moviso part 2 - gabon | https://www.youtube.com/watch?v=BzTggiCiWs0 |
| **Gabon** | Marius Ngombi | https://www.youtube.com/watch?v=Db3vkOji7tk |
| **Gabon** | Mutamba grand chef et Bwitiste Akele-Ungom (Bakele Gabon) part 1 | https://www.youtube.com/watch?v=dEXOHdtAgfc |
| **Gabon** | vyckos ekondo - bomongo | https://www.youtube.com/watch?v=diYq2j6kuig |
| **Gabon** | Gabon: danses Mbeng-Ntam | https://www.youtube.com/watch?v=IaMqA-oTHcA |
| **Gabon** | VTS 01 1 | https://www.youtube.com/watch?v=JuuyEq7UtcI |
| **Gabon** | dance ya kendo 2 | https://www.youtube.com/watch?v=KWP7jYwdhoY |
| **Gabon** | Mutamba grand chef et Bwitiste Akele-Ungom (Bakele Gabon) part 2 | https://www.youtube.com/watch?v=LaaZYf0Hf-8&t |
| **Gabon** | Mamidi 14-02-2020 | https://www.youtube.com/watch?v=OJdjytjmAWs |
| **Gabon** | DSC 007 | https://www.youtube.com/watch?v=RpCYljIfg3s |
| **Gabon** | Missoko Bwiti ceremony | https://www.youtube.com/watch?v=tNKuU9wYbq0 |
| **Gabon** | Le Chamanisme dans ma Vie par le Chaman Moussodou | https://www.youtube.com/watch?v=vPnxT4-wCRk |
| **Gabon** | Danse Lisimbu ou Lechembe des Akele de la Ngounie G4 Gabon | https://www.youtube.com/watch?v=WUeJB_lg8oo |
| **Gabon** | bwiti chez papa a ciel | https://www.youtube.com/watch?v=y1YLz4t78YE&t |
| **Gambia, Senegal** | Kankurang, Manding Initiatory Rite | https://www.youtube.com/watch?v=3gNtkPUuxl0 |
| **Gambia, Senegal** | Kankurang, Manding Initiatory Rite Clip | https://www.youtube.com/watch?v=rdF17Z0db5M |
| **Ghana** | Arrival of Asantehene Otumfuo Osei Tutu,Hogbetsotso Festival in Volta. | https://www.youtube.com/watch?v=_J7bkMzj_GM |

| | | |
|---|---|---|
| **Ghana** | Odwira festival ends with grand durbar of chiefs | https://www.youtube.com/watch?v=_YGNvNXKcxA |
| **Ghana** | Zangbeto Snake Chanting | https://www.youtube.com/watch?v=anWWCGJzDZg&t |
| **Ghana** | The grand durbar | https://www.youtube.com/watch?v=CBCNjLhLaNo |
| **Ghana** | Hogbetsotso Za Festival 2022 _ Grand Durbar Highlights | https://www.youtube.com/watch?v=F84fAhOa_70 |
| **Ghana** | GMB2018 Ashanti region durbar | https://www.youtube.com/watch?v=NZz21x5HLXY&t |
| **Ghana** | ODWIRA FESTIVAL '19 | https://www.youtube.com/watch?v=Os6CosJ5qRo&t |
| **Ghana** | Nana hemanhene at Manhiya palace | https://www.youtube.com/watch?v=pzKwUxkbEu0 |
| **Ghana** | Hemanhene represents Otumfuo Osei Tutu II | https://www.youtube.com/watch?v=qRPEF62JW48&t |
| **Ghana** | Anlo Hogbetsotso Za2019 | https://www.youtube.com/watch?v=RR8fc3L81CM&t |
| **Ghana** | Amazing Ashanti cultural dance | https://www.youtube.com/watch?v=x7R4OjNzQDU&t |
| **Ghana** | Offinso Ahenkro gets new chief | https://www.youtube.com/watch?v=YJlSBv5BBjo&t |
| **Guinea** | Cultural space of Sossa-Bala | https://www.youtube.com/watch?v=27Bo2_e_bVk&t |
| **Ivory Coast** | 5 Impossible African Traditional Dances \| Zaouli - Ivory Coast | https://www.youtube.com/watch?v=BIPtAQLaq0k&t |
| **Ivory Coast** | Gbofe of Afounkaha - the Music of the Transverse Trumps of the Tagbana Community | https://www.youtube.com/watch?v=C8-Y6HdnQlk&t |
| **Kenya** | Gokeh-Araka Traditional Dancers, Part 5, Fourth Dance | https://www.youtube.com/watch?v=3W3LKNnMoBQ |
| **Kenya** | Major ethnic groups in Kenya and their peculiarities | https://www.youtube.com/watch?v=5tZYWy4Wxd4 |
| **Kenya** | Kurya dance at Serengeti cultural centre | https://www.youtube.com/watch?v=5uJbjhdn9tk |
| **Kenya** | Gokeh-Araka Traditional Dancers, Part 6, Fifth Dance | https://www.youtube.com/watch?v=6PP2ngH_BRg |
| **Kenya** | Maasai with Kudu Horn and Lion Skin hat, Kenya | https://www.youtube.com/watch?v=G1GttCbOiNQ |
| **Kenya** | Gokeh-Araka traditional dancers, part 2 | https://www.youtube.com/watch?v=i_QRK0LaWY8&t |
| **Kenya** | Kina Mama Ntimaru, part 1 | https://www.youtube.com/watch?v=IA8xmWMRngA |
| **Kenya** | Music of Kuria district, Kenya | https://www.youtube.com/watch?v=IfZl6uLzlXk |
| **Kenya** | Gokeh-Araka Traditional Dancers, Part 4, Third Dance | https://www.youtube.com/watch?v=K_q1hfBVIrg |
| **Kenya** | Gokeh-Araka Traditional Dancers, Part 8, Seventh Dance | https://www.youtube.com/watch?v=mHMmlT_g-Fs |
| **Kenya** | Gokeh-Araka Traditional Dancers, Part 9, Exit | https://www.youtube.com/watch?v=oMvmFdEyXgQ |
| **Kenya** | Kina Mama Ntimaru, part 2 | https://www.youtube.com/watch?v=PqVAY4qqiTg |
| **Kenya** | Nyabigaka of Banyabasi clan, part 7 | https://www.youtube.com/watch?v=QiqqHRiQKck |
| **Kenya** | Nyabigaka of Banyabasi clan, part 1 | https://www.youtube.com/watch?v=qMuXGAbScms |

| | | |
|---|---|---|
| **Kenya** | Gokeh-Araka Traditional Dancers, Part 7, Sixth Dance | https://www.youtube.com/watch?v=r4fJ6ZZKWPE |
| **Kenya** | Men and Women from Pokot Traditional Dance | https://www.youtube.com/watch?v=rB1CDCBEkkA&t |
| **Kenya** | Nyabigaka of Banyabasi clan, part 5 | https://www.youtube.com/watch?v=V2C08HE-FIw |
| **Kenya, Tanzania** | Music of Kuria, Kenya and Tanzania: Ntimaru musicians | https://www.youtube.com/watch?v=Df451chThuo |
| **Kenya, Tanzania** | Maasai village - folk dance and song | https://www.youtube.com/watch?v=o0cBP81CVqQ |
| **Lesotho** | Mehahlaula Sotho Dance Group | https://www.youtube.com/watch?v=8LdSmDT-Npo |
| **Lesotho** | Lesotho women performing a traditional dance | https://www.youtube.com/watch?v=ezHxxFDZd00 |
| **Malawi** | Mwinhoghe, joyous dance | https://www.youtube.com/watch?v=_axKOaqhOMU |
| **Malawi** | Vimbuza dance | https://www.youtube.com/watch?v=_w788uSWhOs |
| **Malawi** | Chadzunda Nyau dance from Kasungu Malawi | https://www.youtube.com/watch?v=_YO2v0OsHP8&t |
| **Malawi** | Angoni satha onse | https://www.youtube.com/watch?v=0f3jB0ZGMlM |
| **Malawi** | Kutali (Malawi Group) | https://www.youtube.com/watch?v=45hZqxqeLGM |
| **Malawi** | Tchopa, sacrificial dance of Lhomwe | https://www.youtube.com/watch?v=4x3_UA02GuU&t |
| **Malawi** | Vimbuza \| healing dance \| Malawi culture, Tumbuka | https://www.youtube.com/watch?v=5OPSwXXTeJo |
| **Malawi** | Coronation of Inkosu yamakhosi Gomani IV | https://www.youtube.com/watch?v=6JQ60uWWtX8 |
| **Malawi** | Malawi mzimba centenary celebrations | https://www.youtube.com/watch?v=bcNkbJGBCGY&t |
| **Malawi** | Malipenga dance in Nkhata bay | https://www.youtube.com/watch?v=bMHm_kxXM8s&t |
| **Malawi** | Kamuzu Academy Gule Wamkulu Project Part 2 | https://www.youtube.com/watch?v=Bo_t9hwrgZU |
| **Malawi** | Mwinhoghe dance | https://www.youtube.com/watch?v=fVEznoOBmJE |
| **Malawi** | Zakumudzi umhlangano wa maseko - 2018 | https://www.youtube.com/watch?v=gxX1NtMNIvk&t |
| **Malawi** | Angoni series | https://www.youtube.com/watch?v=HqSYIld4kag&t |
| **Malawi** | Ngoma of Ntcheu | https://www.youtube.com/watch?v=JcJzSaI_emM |
| **Malawi** | Nkhata Bay Tonga Malipenga | https://www.youtube.com/watch?v=KgJz5w6qako&t |
| **Malawi** | Ingoma dance from Mchinji, Malawi | https://www.youtube.com/watch?v=LrF_y2K2rWw |
| **Malawi** | Gule Wamkulu ceremony Malawi | https://www.youtube.com/watch?v=Lw8duw_W0OU&t |
| **Malawi** | Nya dance of the Gule Wamkulu secret society in Malawi | https://www.youtube.com/watch?v=O4EDUcpb9FU&t |
| **Malawi** | 11 Mchinji In'goma Troupe 05 | https://www.youtube.com/watch?v=uG2Bkx3CwX4 |
| **Malawi** | Umthetho cultural festival 2017 | https://www.youtube.com/watch?v=uV6S2dEWtPo&t |

| | | |
|---|---|---|
| **Malawi** | Ngoni culture | https://www.youtube.com/watch?v=vc-VpWymdxg |
| **Malawi** | Mbelwa Gomani and Bingu dancing Ingoma | https://www.youtube.com/watch?v=wMfFg9mJgB4&t |
| **Malawi** | Vimbuza healing dance | https://www.youtube.com/watch?v=XInX8QdfOeU&t |
| **Malawi, Mozambique** | History of the Maseko Ngoni of Alawi and Mozambique | https://www.youtube.com/watch?v=zBqmd8VasZc |
| **Malawi, Mozambique, Zambia** | Nyau dancers | https://www.youtube.com/shorts/zmofDtDuR-A |
| **Malawi, Zambia** | Zambia Ngoni singing, 'ukhuluma kanjani' an Ingoma song | https://www.youtube.com/watch?v=EC91RcgoPd4 |
| **Mali** | Dogon African dance | https://www.youtube.com/watch?v=g6998rIxyhg&t |
| **Mali** | Secret society of the Kôrêdugaw, the rite of wisdom in Mali | https://www.youtube.com/watch?v=X2rcBlT87UQ |
| **Mozambique** | Nganda ya anyanja Niassa Mozambique | https://www.youtube.com/watch?v=4f23bhKM4pU |
| **Mozambique** | Dance tradicional Nampula | https://www.youtube.com/watch?v=69t79N_UE4Q |
| **Mozambique** | Chopi Timbila | https://www.youtube.com/watch?v=IgZV9nR-m2o&t |
| **Mozambique** | Traditional music from Mozambique | https://www.youtube.com/watch?v=mkENSAukbgo |
| **Mozambique** | Pemba Mozambique, Tambo international festival 2009 | https://www.youtube.com/watch?v=X_X2kgHptrU&t |
| **Namibia** | The Ondonga King - the king's speech | https://www.youtube.com/watch?v=_2fLD6_XSxE |
| **Namibia** | Batsara, Our heritage | https://www.youtube.com/watch?v=0_j4jw7uFwA&t |
| **Namibia** | Mashi traditional authority celebrates Tulikonge cultural… | https://www.youtube.com/watch?v=1quLd8AjNLo |
| **Namibia** | Batsara 2018 | https://www.youtube.com/watch?v=4qKa265jnPw |
| **Namibia** | Mafwe traditional authority crowns Ngambela | https://www.youtube.com/watch?v=6eclfvCf5m8&t |
| **Namibia** | Behind the scenes - Himba Namibia | https://www.youtube.com/watch?v=7NKxGhgibAI |
| **Namibia** | Zambezi cultural festival Pt 2 | https://www.youtube.com/watch?v=aNB3AbPj9ZU |
| **Namibia** | Batsara Batsapi Cultural Festival August 2018 | https://www.youtube.com/watch?v=b1od8OuOxzU |
| **Namibia** | The coronation of the new Ondonga king | https://www.youtube.com/watch?v=bA8r2SiS-rY |
| **Namibia** | Olufuko cultural festival | https://www.youtube.com/watch?v=bNSQGwbr46Y |
| **Namibia** | Lusata, our roots | https://www.youtube.com/watch?v=CDgi99gmF6U |
| **Namibia** | Zambezi cultural festival 2016 | https://www.youtube.com/watch?v=DnHBzxt3y84&t |
| **Namibia** | Himba dance | https://www.youtube.com/watch?v=FBe_8Px_w6I |
| **Namibia** | Traditional Oshiwambo wedding | https://www.youtube.com/watch?v=inolMl3iisI&t |
| **Namibia** | Batsara basapi | https://www.youtube.com/watch?v=JAT8kD-S4OM |

| | | |
|---|---|---|
| **Namibia** | Mayeyi cultural festival | https://www.youtube.com/watch?v=kN6vyVTtA4s |
| **Namibia** | Women of the Himba tribe that offer sex to visitors | https://www.youtube.com/watch?v=Lgw5zdFnesI&t |
| **Namibia** | Batsara Part 1 | https://www.youtube.com/watch?v=MnmNaPrjQs8 |
| **Namibia** | Mayeyi Ngambela | https://www.youtube.com/watch?v=MR0M_dp8qMA |
| **Namibia** | Zambezi Namushasha culture | https://www.youtube.com/watch?v=MUVIMV5wAI8 |
| **Namibia** | Showelela group \| Lusata cultural festival | https://www.youtube.com/watch?v=NkQQveDHyIA&t |
| **Namibia** | Tulikonge festival-NBC | https://www.youtube.com/watch?v=OlScuao4e7M |
| **Namibia** | Kuomboka group \| Lusata cultural festival | https://www.youtube.com/watch?v=OyqcoSGXjM8 |
| **Namibia** | Lubuta cultural group, Lusata cultural festival | https://www.youtube.com/watch?v=q1zYgdQNbR0&t |
| **Namibia** | Succession battle continues at the Ondonga traditional authority | https://www.youtube.com/watch?v=RMJ6liF-zJs |
| **Namibia** | Ti Shangani in Sangwali | https://www.youtube.com/watch?v=SYHdDAkNsnU |
| **Namibia** | Mayeyi Shikati says Batshara festival promotes unity | https://www.youtube.com/watch?v=Tf8Qjygj4KQ |
| **Namibia** | Freestyle Lusata cultural festival | https://www.youtube.com/watch?v=TJXcjZf-u1c |
| **Namibia** | Batshara festival in 2018 | https://www.youtube.com/watch?v=uCo_3S5Hi8E |
| **Namibia** | Tulikonge express to hit Zambezi river | https://www.youtube.com/watch?v=Up4FhrRw_8s&t |
| **Namibia** | Himba tribal women & their lifestyle | https://www.youtube.com/watch?v=v1gm-W4biGU&t |
| **Namibia** | Chipelu cultural group Lusata cultural festival | https://www.youtube.com/watch?v=W0vhSfADNdc |
| **Namibia** | Busubia bwetu | https://www.youtube.com/watch?v=WGJAu1b0A4M&t |
| **Namibia** | Namibia: the epasha ritual | https://www.youtube.com/watch?v=XbXAIAlg6qc&t |
| **Namibia** | Munitenge | https://www.youtube.com/watch?v=YC_aKOOWZx0&t |
| **Namibia** | Lusata procession \| Lusata cultural festival | https://www.youtube.com/watch?v=Z5ZN0QH4LjI&t |
| **Namibia** | Oshituthi shomangongo, marula fruit festival | https://www.youtube.com/watch?v=ZtF6q7GXtzo |
| **Niger Delta** | Cultural space of Yaaral and Degal | https://www.youtube.com/watch?v=lDoRhqhAEkE&t |
| **Nigeria** | Ogu culture series, part 5 | https://www.youtube.com/watch?v=_5vyPi-mY6I&t |
| **Nigeria** | Ijele Masquerade; Chief Chukwuemeka | https://www.youtube.com/watch?v=1EglFF-MJ38 |
| **Nigeria** | Igbo Ikorodo dance at St Theresa Church dedication | https://www.youtube.com/watch?v=1Mg8e8-tQmU |
| **Nigeria** | Igbu Oja (playing the flute) | https://www.youtube.com/watch?v=1VFY5JN5v6Y |
| **Nigeria** | Ejagham men processing to Chief Norok in old Netim | https://www.youtube.com/watch?v=2znOoLjIyEk&t |

| | | |
|---|---|---|
| **Nigeria** | Ogu culture series, part 4 | https://www.youtube.com/watch?v=3hQj04gDlFk&t |
| **Nigeria** | Live fuji in Oyingbo market (part 15) | https://www.youtube.com/watch?v=4uxps9gfBHM |
| **Nigeria** | Unveiling Ifeadigo Ijele masquerade | https://www.youtube.com/watch?v=6VKjgT1Av4A&t |
| **Nigeria** | Solomon Dalung Tarok cultural | https://www.youtube.com/watch?v=8izlu-vOicI&t |
| **Nigeria** | Tarok cultural festival 2015 | https://www.youtube.com/watch?v=a_eBwpM9bWQ&t |
| **Nigeria** | Igbo Ikorodo dance: obimo parish traditional dance team | https://www.youtube.com/watch?v=aD2qTgkVdpc |
| **Nigeria** | Ejagham women processing to Chief Norok in old Netim | https://www.youtube.com/watch?v=AnEyvVHy0Vw&t |
| **Nigeria** | Nwoge (xylophone), part 3 | https://www.youtube.com/watch?v=Bcf9sR4T_7k&t |
| **Nigeria** | 2013, Tarok Cultural Dance(Nzhem Ichir) | https://www.youtube.com/watch?v=cY_Uq-HJPRE |
| **Nigeria** | Igbo Ikorodo dance: egwu a yara ozo | https://www.youtube.com/watch?v=dAFSwHNI_l4 |
| **Nigeria** | Reconciliation of the Kalara and Donggon clans | https://www.youtube.com/watch?v=ExW2fCJK4Ls&t |
| **Nigeria** | Ejagham 'moni-nkim' dance, part 3 | https://www.youtube.com/watch?v=F0gOi0rsiX8 |
| **Nigeria** | Live fuji in Oyingbo market (part 4) | https://www.youtube.com/watch?v=FvxUA-NxIPs |
| **Nigeria** | Mwaanlee, gurum mo! | https://www.youtube.com/watch?v=fWuK19eEf8E&t |
| **Nigeria** | Igbo Ikorodo dance: maidens dance | https://www.youtube.com/watch?v=hAkh1yZvMLg |
| **Nigeria** | Ikorodo: an Igbo music and dance tradition | https://www.youtube.com/watch?v=HdmY2uqUhww&t |
| **Nigeria** | Nzem Berom 2019 | https://www.youtube.com/watch?v=HqtYeLgs5F8&t |
| **Nigeria** | Tarok cultural festival 2017 | https://www.youtube.com/watch?v=Hs5XR5kocys |
| **Nigeria** | Uwaifo junior and prof Vector Uwaifo | https://www.youtube.com/watch?v=HUVctcPOd9g&t |
| **Nigeria** | Igbo Ikorodo dance at the African studies association | https://www.youtube.com/watch?v=IIphUpa0vko |
| **Nigeria** | Ogene music from Awka Anambra, Nigeria, Part 5 | https://www.youtube.com/watch?v=J5_e9ojD1WI&t |
| **Nigeria** | Top 11 of the biggest cultural festivals in Nigeria | https://www.youtube.com/watch?v=jHFu1qKhiNY |
| **Nigeria** | Live fuji in Oyingbo market (part 16) | https://www.youtube.com/watch?v=JKXnjf5_RKg |
| **Nigeria** | Interview with Laz Ekwueme, part 07a | https://www.youtube.com/watch?v=JQT6vP6wj8k&t |
| **Nigeria** | Raphael Ukpatu (Chairman) Agbani-Nguru Ikorodo group | https://www.youtube.com/watch?v=jYufY5MZ_dA |
| **Nigeria** | Nwoge (xylophone) | https://www.youtube.com/watch?v=L0R1475MBPU |
| **Nigeria** | Masquerades; Ajofia Nnewi | https://www.youtube.com/watch?v=lf8xRHS2L1Q&t |
| **Nigeria** | Izam Icir Otarok | https://www.youtube.com/watch?v=mOi1M8LgSUc |

| | | |
|---|---|---|
| **Nigeria** | Igbo Ikorodo dance with masquerade | https://www.youtube.com/watch?v=MQ1vQqygv3c&t |
| **Nigeria** | Igbo dance: Ije New Nne, part 2 | https://www.youtube.com/watch?v=mz2F2ylcpaI |
| **Nigeria** | Ojile cultural dance, part 3 | https://www.youtube.com/watch?v=mZFcQMfgqXc&t |
| **Nigeria** | Ogene singers in Okigwe | https://www.youtube.com/watch?v=O5-kOcv9N3A |
| **Nigeria** | Igbo Ikorodo dance at St Theresa Church dedication (part ..) | https://www.youtube.com/watch?v=OZRwEyZ1zMk |
| **Nigeria** | Tarok music amutu live concert | https://www.youtube.com/watch?v=pAMuOHbuoaU |
| **Nigeria** | Dundun ensemble in Oyo State | https://www.youtube.com/watch?v=QVFkd6eXwY0 |
| **Nigeria** | Ikpamodo Women's group, part 3 | https://www.youtube.com/watch?v=qw3KLSRiyEM&t |
| **Nigeria** | Ogene music from Awka Anambra, Nigeria, Part 2 | https://www.youtube.com/watch?v=RkBygYS-s-w&t |
| **Nigeria** | ADEPt centre @UNN: St Theresa Ikorodo | https://www.youtube.com/watch?v=Rn3mE5NuIIE |
| **Nigeria** | Igbo atilogwu dance, part 10 | https://www.youtube.com/watch?v=rn5fdMiUEwI&t |
| **Nigeria** | Igbo 'egedege dance by Ebube-Eze, Part 3 | https://www.youtube.com/watch?v=saMzNGsvU9A |
| **Nigeria** | Cultural festivals in Nigeria | https://www.youtube.com/watch?v=SsU6v0K9qw0&t |
| **Nigeria** | Ogene praise singers and Igbo masquerade | https://www.youtube.com/watch?v=tBkQwZoGQH4 |
| **Nigeria** | Nsukka folk songs, part 5 | https://www.youtube.com/watch?v=TZvxBVpBMWY |
| **Nigeria** | Ogene music from Awka Anambra, Nigeria, Part 4 | https://www.youtube.com/watch?v=u6uA2bE_sJM |
| **Nigeria** | Lions of the Nsukka 'aghamgbele' | https://www.youtube.com/watch?v=WRdqxE4SjaI |
| **Nigeria** | Alabi Ogundepo: 'Oriki Ogun' | https://www.youtube.com/watch?v=XPf8_pxFki4&t |
| **Nigeria** | Igbo Ikorodo dance: agbani-nguru | https://www.youtube.com/watch?v=XT_K6inGoMI&t |
| **Nigeria** | A di oro awitunwi | https://www.youtube.com/watch?v=xY45uHMzhhI&t |
| **Nigeria** | Blue roots in Nigeria | https://www.youtube.com/watch?v=zP7ddCDSeas |
| **Rwanda** | Ryangombe wari imana y' u Rwanda | https://www.youtube.com/watch?v=I_Sx5xeQhvg&t |
| **Senegal** | Xooy, divination ceremony of Serer of Senegal | https://www.youtube.com/watch?v=iG4ISBIQdAc&t |
| **Sierra Leone** | Kamajoh | https://www.youtube.com/watch?v=FGV2fY_dcL4 |
| **South Africa** | TOP 4 CRAZIEST XITSONGA DANCERS | https://www.youtube.com/watch?v=14QNJ5ZEd88&t |
| **South Africa** | Shembe: isigeke | https://www.youtube.com/watch?v=4qLbRnuvbT8&t |
| **South Africa** | HERBAL HEALER BUYS OWN PLANE AND PLANS AIRPORT | https://www.youtube.com/watch?v=5QmrIiUbKwU |
| **South Africa** | Inkosi Mabhudu Tembe on Umthayi Marula festival | https://www.youtube.com/watch?v=7-jq0IWZUZo&t |

| | | |
|---|---|---|
| **South Africa** | ubkhosi showed up | https://www.youtube.com/watch?v=8346UWrf9Fk&t |
| **South Africa** | The Shangaan Dancers Show us their Best Moves! | https://www.youtube.com/watch?v=atjreI9zFxY |
| **South Africa** | Khekhekhe first fruits & snake ceremony | https://www.youtube.com/watch?v=AZfsRXouPUU |
| **South Africa** | Omang vhaVenda Fundudzi | https://www.youtube.com/watch?v=Bf-jZeWREz4 |
| **South Africa** | Venda cultural dance | https://www.youtube.com/watch?v=D-OAz6T-krc |
| **South Africa** | Koma…initiation ceremony | https://www.youtube.com/watch?v=fyOi65ib4h0 |
| **South Africa** | Omang? Dikgafela - Mafikeng | https://www.youtube.com/watch?v=G79U2LnqlSM&t |
| **South Africa** | Venda traditional dance (Malende) | https://www.youtube.com/watch?v=Gcfxl8hPR0E |
| **South Africa** | Amabhukudwana - Yithi ama Ndebele | https://www.youtube.com/watch?v=gSHDU_nGdB0 |
| **South Africa** | King Shaka day 2015 | https://www.youtube.com/watch?v=hkb4PkPHyTI&t |
| **South Africa** | Ndebele Kingdom Mr and Miss Indoni Cultural South Africa 2019. | https://www.youtube.com/watch?v=IvKxLOb5P5s&t |
| **South Africa** | Amandebele from Dr JS Moroka | https://www.youtube.com/watch?v=lLZddm89xPg |
| **South Africa** | Dance ceremony at Shembe gathering | https://www.youtube.com/watch?v=LnQfXc4EhAQ |
| **South Africa** | Shembe: ikhethelo lonyazi (2) | https://www.youtube.com/watch?v=O2HkFncEFHo&t |
| **South Africa** | King Ndzundza Top five royal legacy message of support | https://www.youtube.com/watch?v=qO09ZxCsyV0 |
| **South Africa** | The domba dance or python dance in Venda Limpopo | https://www.youtube.com/watch?v=R29xw7h4Ye0 |
| **South Africa** | UNDZUNDZA NEENKHALI ZEBHOSA - ISKHWELE | https://www.youtube.com/watch?v=RhxT3-1ADEE |
| **South Africa** | Shangana Cultural Village | https://www.youtube.com/watch?v=SXsxUL8oNpk |
| **South Africa** | Omang? Koma (initiation) | https://www.youtube.com/watch?v=VtlnChnK9Q8 |
| **South Africa** | The Venda | https://www.youtube.com/watch?v=xgo2GX_ay60&t |
| **South Africa** | Shembe | https://www.youtube.com/watch?v=Yg98wgkLoIo |
| **South Africa** | Discussion: the role of umthayi marula festival | https://www.youtube.com/watch?v=zfae0uXdupI&t |
| **South Africa, Zambia** | Ngoni vs Zulu (Cultural dances similarities) | https://www.youtube.com/watch?v=8eq2g_RJSQs&t |
| **South Sudan** | Abii vs Aboudit wrestling match | https://www.youtube.com/watch?v=0_Ssn7oIpAo&t |
| **South Sudan** | Mundari Dance | https://www.youtube.com/watch?v=0A5P2F_oUVM&t |
| **South Sudan** | The best of Dinka Bor wrestlers Loor in Juba, South Sudan | https://www.youtube.com/watch?v=1TxGb1R-GBA&t |
| **South Sudan** | South Sudan Taposa People | https://www.youtube.com/watch?v=2ifrRBDE0ZY |
| **South Sudan** | How Murle dominant age-set in Pibor town organized dances in their traditional regalia | https://www.youtube.com/watch?v=9quxkaoy6Zk&t |

| | | |
|---|---|---|
| **South Sudan** | Sudan - Pibor 2007 - Generation Dance | https://www.youtube.com/watch?v=ETCMh5QkeFg |
| **South Sudan** | The Titi Generation from the Murle Tribe, dancing in Pibor, | https://www.youtube.com/watch?v=lN6jald3GTg |
| **South Sudan** | Thithi Generation (The Green Warriors) are dancing in Pibor Town | https://www.youtube.com/watch?v=Lr2I4y70e0w&t |
| **South Sudan** | Murle traditional dance in Pibor | https://www.youtube.com/watch?v=lusdReGhSZk |
| **South Sudan** | Mundari the nomad cattle herders from South Sudan | https://www.youtube.com/watch?v=n8PDm4BVNa0 |
| **South Sudan** | Mundari cultural dancers | https://www.youtube.com/watch?v=ofFEOfOIoC8&t |
| **South Sudan** | IMG 0513 (Titi generation) | https://www.youtube.com/watch?v=UApHDYU7x8U |
| **South Sudan** | Dinka Bor Wrestling between Paleek vs Aboudit | https://www.youtube.com/watch?v=ZnsnI36tJv8&t |
| **Southern Africa** | Umshado | https://www.youtube.com/watch?v=CvIiRKuyO5g |
| **Southern Africa** | Ndau-Ukuthwasa the journey | https://www.youtube.com/watch?v=ehTGLQOYLYE&t |
| **Southern Africa** | Ndau ancestral dance | https://www.youtube.com/watch?v=GtTrGvApK88&t |
| **Southern Africa** | Umshado | https://www.youtube.com/watch?v=QqnTXpI6qZk |
| **Southern Africa** | Ndauwee Dungamazi mundau | https://www.youtube.com/watch?v=YVtr9_FGYT8&t |
| **Sudan** | Expedition across Africa - Kambala dance in Kadugli | https://www.youtube.com/watch?v=dNh_YqF1Usc&t |
| **Tanzania** | Guruguru's Troupe, part 3 | https://www.youtube.com/watch?v=1FAG1uqEwkc |
| **Tanzania** | Makonde Cultural Festival, Chikundi, Tanzania, Oct 2013 | https://www.youtube.com/watch?v=3X8UOr0q6Mg&t |
| **Tanzania** | Guruguru's Troupe, part 4 | https://www.youtube.com/watch?v=7aokYIgikP8 |
| **Tanzania** | Shikati Shufu of Mayeyi tribe says COVID-19 has affected the traditional authority's activities | https://www.youtube.com/watch?v=7CLlD5mNK3k |
| **Tanzania** | NGOMA ASILIA YA WANYAMBO WA KARAGWE KAGERA NCHINI TANZANIA | https://www.youtube.com/watch?v=Gxf_aulNK70 |
| **Tanzania** | Historia ya kabila la Wanyambo na sifa za Wanawake wa Kinyambo | https://www.youtube.com/watch?v=hwYdHjcHcwo&t |
| **Tanzania** | Python snake dance | https://www.youtube.com/watch?v=jhoztTq-EMY&t |
| **Tanzania** | Serengeti cultural festival 2022 | https://www.youtube.com/watch?v=pEkDnB6cP9U |
| **Tanzania** | Serengeti cultural festival | https://www.youtube.com/watch?v=qrhd8gUYIko |
| **Tanzania** | Dancing with snake tribe of Tanzania | https://www.youtube.com/watch?v=r6hYmGscR_Q |
| **Tanzania** | Tanzania Traditional Music/African Best Village Dance Style - Makumbusho Village Museum | https://www.youtube.com/watch?v=rtd-thZLq1M&t |
| **Tanzania** | Documentary portrait 'Ngorongoro Masai woman' | https://www.youtube.com/watch?v=X9nMlzB1Y68&t |
| **Tanzania** | HISTORIA YA KABILA LA WANYAMBO | https://www.youtube.com/watch?v=zvUk0R92sAU&t |

| | | |
|---|---|---|
| **Togo** | Tour of Lomé Fetish market | https://www.youtube.com/watch?v=p3g3WgUtPyc&t |
| **Togo** | Inside the World's Biggest Voodoo Market | https://www.youtube.com/watch?v=wh9jd8qghtY |
| **Uganda** | Acoli cultural festival 2019 - video 4 | https://www.youtube.com/watch?v=2-Uw_6d1PXo |
| **Uganda** | Bwola - the Acholi royal dance | https://www.youtube.com/watch?v=2FYXw2I726M |
| **Uganda** | Mwel Bwola (Acholi royal dance) | https://www.youtube.com/watch?v=A1Oxe6AJ0mw |
| **Uganda** | Gichama's Troupe, part 2 | https://www.youtube.com/watch?v=asrOsGvC1Cg&t |
| **Uganda** | African traditional dance Bwola (2019) | https://www.youtube.com/watch?v=bHpyWWuV3hU&t |
| **Uganda** | Gichama's Troupe, part 1 | https://www.youtube.com/watch?v=dwoNxyVB7s4&t |
| **Uganda** | Acholi cultural festival 2018 | https://www.youtube.com/watch?v=hFj1CmcWxuk |
| **Uganda** | Acholi cultural dance bwola | https://www.youtube.com/watch?v=hG9ogLsWSac |
| **Uganda** | Most Loved KING in Africa - Ronald Muwenda Mutebi II, KABAKA of Buganda Kingdom | https://www.youtube.com/watch?v=nGQXPwAjan4 |
| **Uganda** | Bugumbwe troupe, part 4 | https://www.youtube.com/watch?v=qmtF4i9QCpg |
| **Uganda** | Africa's best traditional dance // Acholi dance | https://www.youtube.com/watch?v=QNs_u9h0W-w&t |
| **Uganda** | Acoli cultural festival 2019 video 5 | https://www.youtube.com/watch?v=SFis2QYrcW8&t |
| **Uganda** | Acholi traditional dance (Bwola dance) | https://www.youtube.com/watch?v=sXC80VKFao8 |
| **Uganda** | Muganda dance | https://www.youtube.com/watch?v=v47kNLyxlxY&t |
| **Uganda** | The Amahamba - hunting dance of the A'Batwa | https://www.youtube.com/watch?v=YBGxrAr5jx4 |
| **Uganda** | Myel Bwola AMSA Oct 2016 | https://www.youtube.com/watch?v=YEY_IDQgHQk |
| **Uganda** | Acoli Rwot Onen Achana arrives at Gul cultural festival 2017 | https://www.youtube.com/watch?v=zmm3he2FXvw |
| **Uganda** | Mwel Bwola (Acholi royal dance) | https://www.youtube.com/watch?v=ZpYzQknU7AI |
| **West Africa** | The Toura | https://www.youtube.com/watch?v=CO1z4gC20zU |
| **West Africa** | Ifa divination system | https://www.youtube.com/watch?v=k9lGVF6jYN4 |
| **Zambia** | Chief mumena blesses land during Lubinda Ntogo traditional ceremony | https://www.facebook.com/watch/?v=637937750035149 |
| **Zambia** | Mutomolo ceremony of Namwanga people | https://www.youtube.com/shorts/2_lgots9NQ0 |
| **Zambia** | Bene Mukuni traditional ceremony Livingstone, Zambia | https://www.youtube.com/shorts/7VMcX0VLi1Q |
| **Zambia** | Chief Mwewa of Abene Ng'umbo at Manga during Kwanga | https://www.youtube.com/shorts/J21rUa6BtUQ |
| **Zambia** | KULAMBAKUBWALO traditional ceremony of the Lenje speaking people. | https://www.youtube.com/shorts/oqxJnwwOcHY |
| **Zambia** | The Ngoni of Eastern Zambia | https://www.youtube.com/shorts/otwR0cCKJR8 |

| | | |
|---|---|---|
| **Zambia** | Woman singing during the Kwanga traditional ceremony | https://www.youtube.com/shorts/Qes-jS-udUI |
| **Zambia** | Unveiling of the new senior chief Mukuni Ng'ombwe VI | https://www.youtube.com/shorts/X4muHeu4OcQ |
| **Zambia** | Kulamba ceremony of Chewa people | https://www.youtube.com/shorts/zZIy44bpmRc |
| **Zambia** | Kulamba kubwalo cultural performance 3 | https://www.youtube.com/watch?v=0XbLwEoxVbk |
| **Zambia** | Makishi dance - Valizanga video mix | https://www.youtube.com/watch?v=13G-FYlHwfQ&t |
| **Zambia** | The amazing ethnic groups of Zambia | https://www.youtube.com/watch?v=1bPjEzA1qGU&t |
| **Zambia** | Reason Why Zambian folk dance is the best! | https://www.youtube.com/watch?v=2JBGynOUA0U |
| **Zambia** | Kawale a luvale traditional dance performed during the 2019… | https://www.youtube.com/watch?v=3qj_z5PSpTc |
| **Zambia** | 2022 Malaila Rehearsal in Kasamanda, Msoro Chiefdom | https://www.youtube.com/watch?v=41-7_LkP-gw&t |
| **Zambia** | Ntongo ceremony 2022 | https://www.youtube.com/watch?v=4NIRhFRlkL4&t |
| **Zambia** | Mooba dance of the Lenje ethnic group of Central Province of.. | https://www.youtube.com/watch?v=4t3W_ngcUnA |
| **Zambia** | Lenga Navo culture group - woman | https://www.youtube.com/watch?v=50TG7a364Fk |
| **Zambia** | The Day before the 2022 Malaila Traditional Ceremony | https://www.youtube.com/watch?v=5I7z1BBZryg |
| **Zambia** | Praise singing for Mpenzi | https://www.youtube.com/watch?v=5krfI5gdU-0 |
| **Zambia** | TONGA DANCE -2014-09-13 | https://www.youtube.com/watch?v=71YjbewNbTs |
| **Zambia** | Mindmater-quiz-S03-e07-shimunenga ceremony | https://www.youtube.com/watch?v=75G05DlQfyM |
| **Zambia** | Charles Likumbi - Mama lena | https://www.youtube.com/watch?v=7hvcxMsRytM |
| **Zambia** | Kazanga ceremony of the Nyoka people in Zambia | https://www.youtube.com/watch?v=7o7rsxMurak&t |
| **Zambia** | Paramount chief Mpenzi of the Ngoni people | https://www.youtube.com/watch?v=85Ybl9vRjDI |
| **Zambia** | Kuomboka 2017 in-depth documentary | https://www.youtube.com/watch?v=8A8ULjcToPk |
| **Zambia** | Ngoma dance | https://www.youtube.com/watch?v=A8FEPNKAyV8 |
| **Zambia** | Kufuluela: return of the King - Welcom ceremony | https://www.youtube.com/watch?v=AARzuroWKYM&t |
| **Zambia** | His Royal highness Mwatakazembe XIX | https://www.youtube.com/watch?v=AEsw-gOvNgA |
| **Zambia** | Shimunenga ceremony | https://www.youtube.com/watch?v=aIil9D4h8Bk |
| **Zambia** | Makishi dance - ou mwana wayami kaha | https://www.youtube.com/watch?v=Am_pwnQvL_c |
| **Zambia** | DANTHO TRADITIONAL CEREMONY | https://www.youtube.com/watch?v=AT9Qm-lgmmM |
| **Zambia** | Shimunenga ceremony of the Ila people. Maala, Namwala | https://www.youtube.com/watch?v=bSpldVtHKcg |

| | | |
|---|---|---|
| **Zambia** | The Ncwala Ceremony of the Ngoni People of Eastern Zambia, Mozambique and Malawi | https://www.youtube.com/watch?v=BYzmJ9C07eQ |
| **Zambia** | Makishi dance - tuejilila mahina | https://www.youtube.com/watch?v=CdZcDEgai-g |
| **Zambia** | Chakwela makumbi ceremony 19 10 14 | https://www.youtube.com/watch?v=cFE-V8vsPoA&t |
| **Zambia** | Chief Mukumbi Burial-2023 | https://www.youtube.com/watch?v=dPzuktbti-Q&t |
| **Zambia** | Makishi dance - videos mix | https://www.youtube.com/watch?v=DSiSSu7vgug&t |
| **Zambia** | Chivweka lighting of fire using sticks from nature | https://www.youtube.com/watch?v=eLsltilJaD0 |
| **Zambia** | Zambia gold | https://www.youtube.com/watch?v=EpVYE92mxC0&t |
| **Zambia** | A Zambian traditional dance by the Chikunda people | https://www.youtube.com/watch?v=eShuDaBEx1M |
| **Zambia** | Shimunenga | https://www.youtube.com/watch?v=ezGdbJo9i0c |
| **Zambia** | Motomboko traditional festival dance of Zambia | https://www.youtube.com/watch?v=F2fXhgWo1xs |
| **Zambia** | Traditional dancing | https://www.youtube.com/watch?v=f4CB0Fxz9JU |
| **Zambia** | Kuomboka ceremony - Zambia | https://www.youtube.com/watch?v=FNy3xOazYKE&t |
| **Zambia** | Tchianda dance - cota ca chokwe dance group at Likumbi | https://www.youtube.com/watch?v=FrSVWYOuUjQ |
| **Zambia** | Ngoni Dance in Zambia | https://www.youtube.com/watch?v=GFS_bO2W_1k |
| **Zambia** | N'cwala ceremony/festival | https://www.youtube.com/watch?v=GGPMTYV5vKo |
| **Zambia** | Senior chief puta's bulile ceremony 06 08 2017 | https://www.youtube.com/watch?v=GhKnu3TALnc |
| **Zambia** | Kulamba kubwalo | https://www.youtube.com/watch?v=gIZtn55eV3Q |
| **Zambia** | KULAMBA KUBWALO | https://www.youtube.com/watch?v=HA4rmzYzJaU |
| **Zambia** | Lunda dance | https://www.youtube.com/watch?v=HaF6S7M2_lU&t |
| **Zambia** | Nkoya dance - kazanga ceremony dance | https://www.youtube.com/watch?v=hIFeVBWwgtg |
| **Zambia** | Moya Nandao - Bwishi part 2 | https://www.youtube.com/watch?v=hlaoV4i2YjM&t |
| **Zambia** | Kuomboka Ceremony - 'to get out of water' | https://www.youtube.com/watch?v=HPvnTOP-eA8 |
| **Zambia** | Chivweka 2015 ceremony of the luchazi people of northwestern Zambia | https://www.youtube.com/watch?v=Hs9wEKN34V8 |
| **Zambia** | lwiindi ceremony of tonga people in monze ( nsabata) Zambia | https://www.youtube.com/watch?v=IHf8NLmRjUY |
| **Zambia** | Kufukwila at chief mukumbi | https://www.youtube.com/watch?v=ijx7cI_qs7g |
| **Zambia** | Mindmaster-quiz-s03-e16-lunda lubanza | https://www.youtube.com/watch?v=iLAmlanvDoE |
| **Zambia** | E7 The Mukuni Royal Dynasty, Zambia | https://www.youtube.com/watch?v=iR0u-i79rzY |

| | | |
|---|---|---|
| **Zambia** | Kulamba kubwalo cultural performance | https://www.youtube.com/watch?v=iSpzMjkzL7I |
| **Zambia** | Chikuni Tonga Concert | https://www.youtube.com/watch?v=j4ICM-ZDdmc |
| **Zambia** | Nyau dancers - Chipata (Mkanda) | https://www.youtube.com/watch?v=jn-vcFDQjA0 |
| **Zambia** | Lwindi ceremony Mukuni Zambia Jan 4, 2010 | https://www.youtube.com/watch?v=JPUxtARujA8 |
| **Zambia** | Traditional ceremonial dancing of Sala people | https://www.youtube.com/watch?v=JUT2Xn9rez4 |
| **Zambia** | Lukwakwa dancing group are ready | https://www.youtube.com/watch?v=Kbe1xCYeASU |
| **Zambia** | Muvi TV ukusefya pangwena report 1 | https://www.youtube.com/watch?v=KgDyu8cFMYo |
| **Zambia** | ZAMBIA: African traditional village of MUKUNI | https://www.youtube.com/watch?v=kWtYIU_fQd8 |
| **Zambia** | Likumbi lya mize | https://www.youtube.com/watch?v=l0l8XbNehnY |
| **Zambia** | Ncwala traditional ceremony 2020 | https://www.youtube.com/watch?v=lgGsZ19ogZM |
| **Zambia** | ncwala ceremony Zambia | https://www.youtube.com/watch?v=lgH0ZK0_a8M |
| **Zambia** | Mutomboko ceremony 2012 | https://www.youtube.com/watch?v=LHAjzTa6GhI&t |
| **Zambia** | Dantho traditional ceremony part 2 | https://www.youtube.com/watch?v=lTueFgbCfdc&t |
| **Zambia** | Traditional ceremony 2011 in Lusaka Zambia Vol4 | https://www.youtube.com/watch?v=luoYdgXdqLE |
| **Zambia** | Waitwikia on Mutomolo ceremony | https://www.youtube.com/watch?v=lV9L5Bm0Us0 |
| **Zambia** | Zambian n'cwala ceremony | https://www.youtube.com/watch?v=m8a4kq-PkAQ&t |
| **Zambia** | Makishi dancers Northwestern province | https://www.youtube.com/watch?v=mdh0aVW7ZzY&t |
| **Zambia** | Makishi Dance - Lipumpu | https://www.youtube.com/watch?v=mRDuSYa25cQ |
| **Zambia** | Lala people cultural dance | https://www.youtube.com/watch?v=MsAt-oQms7U&t |
| **Zambia** | ncwala ceremony 2019 | https://www.youtube.com/watch?v=MUeCarQShg0&t |
| **Zambia** | Major Ethnic groups in Zambia and their peculiarities | https://www.youtube.com/watch?v=Mw4jAha6EUo&t |
| **Zambia** | Kulamba kubwalo cultural performance 2 | https://www.youtube.com/watch?v=n0PSKKDD98I |
| **Zambia** | Nc'wala ceremony Zambia 2020 | https://www.youtube.com/watch?v=n4omeHit7L0 |
| **Zambia** | Kulamba Ceremony 2009 Part 1 (Promo Only) | https://www.youtube.com/watch?v=N8LZ9L3Hk6g |
| **Zambia** | Oolichoolwe | https://www.youtube.com/watch?v=nE91J4TAuic |
| **Zambia** | Chakwela Makumbi Ceremony of Soli people | https://www.youtube.com/watch?v=Ng1xerDeL2k |
| **Zambia** | King kuku - Mitende Yenu | https://www.youtube.com/watch?v=nL_S4GniZfY |
| **Zambia** | Kulamba Ceremony 2009 Part 2 (Promo Only) | https://www.youtube.com/watch?v=NMJk0Psc6tU |

| | | |
|---|---|---|
| **Zambia** | Mupala Makishi by M. Wabei Siyolwe | https://www.youtube.com/watch?v=NnijpAdx-so&t |
| **Zambia** | Makishi masquerade 3 | https://www.youtube.com/watch?v=noC27QXUcMY&t |
| **Zambia** | Lwiindi Gonde ceremony 2011 | https://www.youtube.com/watch?v=NYOQmbCp4lA |
| **Zambia** | Likumbi Lya Mize | https://www.youtube.com/watch?v=nyQzWXGB8NY |
| **Zambia** | Ngoni Dance in Zambia on the grounds of Victoria Falls | https://www.youtube.com/watch?v=O9R_3ixj0lM |
| **Zambia** | The Ngoni warriors performing at the Bene Mukuni Traditional Ceremony \| Chief Mukuni joins | https://www.youtube.com/watch?v=oAbUJI9gwt4 |
| **Zambia** | Nakachuka ne kambimbi (lunda traditional dance) | https://www.youtube.com/watch?v=ocznj1OyJqI |
| **Zambia** | Private | https://www.youtube.com/watch?v=OXp5p9tQmkc |
| **Zambia** | Mutomboko Ceremony 2022\| Dance of Conquest. | https://www.youtube.com/watch?v=p4a86d5Cb-8 |
| **Zambia** | Villa 1 Nyau Bronco funeral Dzivareskwa Gule Wankulu | https://www.youtube.com/watch?v=P5NvVWZdnNo |
| **Zambia** | shimunenga ceremony of ila people in maala | https://www.youtube.com/watch?v=pFoqzZP_A6o&t |
| **Zambia** | Lakes in Zambia: children playing in Lake Chifunabuli Lubwe area | https://www.youtube.com/watch?v=PkqCdien0Ag |
| **Zambia** | Juba Ja_Nsomo Kaonde Ceremony | https://www.youtube.com/watch?v=QbaPev046HI |
| **Zambia** | History of Chabuka Ceremony - Senior Chief Matanda of Ushi people of Luapula Province - Zambia | https://www.youtube.com/watch?v=QccAgERFGPY&t |
| **Zambia** | Gule wamkulu | https://www.youtube.com/watch?v=QLEMlBjhm3E |
| **Zambia** | shimunenga ceremony of ila people of maala, namwala | https://www.youtube.com/watch?v=qO45NSNs7e4 |
| **Zambia** | Lwindi ceremony | https://www.youtube.com/watch?v=QO6MjTLpeI0 |
| **Zambia** | Kulamba Traditional Ceremony of the Chewa People | https://www.youtube.com/watch?v=QQXTuj7XaVs |
| **Zambia** | Kalela dance | https://www.youtube.com/watch?v=R7z_2EE43J0&t |
| **Zambia** | Dantho Traditional ceremony II Shot and produced by Lawrence Mwamba | https://www.youtube.com/watch?v=RCdPJQUS1wQ&t |
| **Zambia** | Kwilimuna ceremony | https://www.youtube.com/watch?v=RIfiz6odmy8 |
| **Zambia** | Giving a gift to a chief | https://www.youtube.com/watch?v=rmCQVUUrx5Y&t |
| **Zambia** | Mukanda (Luchazi rites of passages) | https://www.youtube.com/watch?v=RNIi63dAX7s |
| **Zambia** | Shimunenga traditional ceremony (2020) | https://www.youtube.com/watch?v=s2R89htWQPk |
| **Zambia** | Amabila ceremony | https://www.youtube.com/watch?v=SLv4LHNqByM |
| **Zambia** | Kuomboka 07 4 | https://www.youtube.com/watch?v=sVarIxOb8jo&t |
| **Zambia** | MALAILA Traditional Ceremony of the Chishinga people of Luapula | https://www.youtube.com/watch?v=swV9AoBfXsY |

| | | |
|---|---|---|
| **Zambia** | Zambia traditional dance | https://www.youtube.com/watch?v=TBt6h6CyBcg |
| **Zambia** | AfricaWrites Archives 2008: The Kulamba Kubwalo ceremony of Central Province, Zambia | https://www.youtube.com/watch?v=TBTqVZgifxE |
| **Zambia** | Mwachelele cultural group | https://www.youtube.com/watch?v=UAtCVOUNLNo |
| **Zambia** | KULAMBA CEREMONY 2022 IN ALL IT'S SLENDOR | https://www.youtube.com/watch?v=ugmB1_5wiqY&t |
| **Zambia** | Dr Kambwili blocked from attending ukusefya pa ngwena | https://www.youtube.com/watch?v=vrbT9R9jwW8&t |
| **Zambia** | Mutomboko ceremony | https://www.youtube.com/watch?v=w1nRPWkbBGA&t |
| **Zambia** | Zengani | https://www.youtube.com/watch?v=w6ldc7xZGgM |
| **Zambia** | Chief mukumbi celebrating | https://www.youtube.com/watch?v=WeT6mJPfywY |
| **Zambia** | NCWALA CULTURAL CEREMONY ZAMBIA 2017 1 | https://www.youtube.com/watch?v=wOUY4-TyhHg&t |
| **Zambia** | Saicool - Kwenje | https://www.youtube.com/watch?v=Wst0hZYVMtY&t |
| **Zambia** | Mboanjikana cultural group | https://www.youtube.com/watch?v=wu6W23axokE |
| **Zambia** | UKUSEFYA PANG'WENA Traditional Ceremony | https://www.youtube.com/watch?v=wZmrekRfKvA |
| **Zambia** | Masks & men part 1: the soli oulen & Koma | https://www.youtube.com/watch?v=XQKKqbpHKuY |
| **Zambia** | The 2017 Kwilimuna Traditional Ceremony 23 07 2017 | https://www.youtube.com/watch?v=Y0tylB6Em80&t |
| **Zambia** | African traditional dance and drumming Zambia | https://www.youtube.com/watch?v=yEkWPb88dCk |
| **Zambia** | LWIINDI GONDE TRADITIONAL CEREMONY VIDEO | https://www.youtube.com/watch?v=YFDJwoyNfM4 |
| **Zambia** | 2022 Bene Mukuni Ceremony Part One | https://www.youtube.com/watch?v=YoxiIAYqbRc |
| **Zambia** | Likumbi lya mize traditional ceremony Zambia | https://www.youtube.com/watch?v=Z_wJ2mcZpLs&t |
| **Zambia** | Makishi dance - muchapa dance at Likumbi lya mize | https://www.youtube.com/watch?v=zIjfAjHol3U&t |
| **Zambia** | Kulamba Kubwalo Cultural Performance 4 | https://www.youtube.com/watch?v=ZUAyed3IKo0 |
| **Zambia** | Lubinda Ntongo Ceremony - (A Celebration of Unity, Gratitude, and Prosperity) | https://www.youtube.com/watch?v=zZArw7dI3eY |
| **Zimbabwe** | Shamva stoka Nyau dancers | https://www.youtube.com/shorts/7f_3P9PfEhE |
| **Zimbabwe** | Isitshikitsha, a Ndebele traditional dance from Bulawayo, Zimbabwe. | https://www.youtube.com/watch?v=2neF-bPyZKg&t |
| **Zimbabwe** | Bulawayo Traditional Dances , Lighter moments at CODEPA #263Chat | https://www.youtube.com/watch?v=5uPaZ7jRBKs&t |
| **Zimbabwe** | Nyau de tsangano | https://www.youtube.com/watch?v=9wu_9NpfN4A |
| **Zimbabwe** | Traditional dance. Muzokombz primary school, Zimbabwe | https://www.youtube.com/watch?v=ALErR9ossP0&t |
| **Zimbabwe** | Mbende jerusarema dance from murewa Zimbabwe | https://www.youtube.com/watch?v=AQigQzpy8JM |

| | | |
|---|---|---|
| **Zimbabwe** | Zimbabwean Tonga traditional dance | https://www.youtube.com/watch?v=BwubdJ8ubJs |
| **Zimbabwe** | Traditional Dance at Great Zimbabwe | https://www.youtube.com/watch?v=bY3s-AT5K6k |
| **Zimbabwe** | Traditional music at Great Zimbabwe | https://www.youtube.com/watch?v=c73eOxO4hHA |
| **Zimbabwe** | Matabele Ndebele Lobengula Zulu warriors | https://www.youtube.com/watch?v=ci4TPuyknfs&t |
| **Zimbabwe** | Mpande - Tonga rituals | https://www.youtube.com/watch?v=DNXrP6GPQqM |
| **Zimbabwe** | Funny learning Zimbabwean traditional dance | https://www.youtube.com/watch?v=FFcUAZxQGDE&t |
| **Zimbabwe** | Amabhiza at Matabeleland South Culture Week 2012 | https://www.youtube.com/watch?v=FypELBqOKGY |
| **Zimbabwe** | Tonga people - Zimbabwe | https://www.youtube.com/watch?v=JV-ESRoKAHU |
| **Zimbabwe** | Mbende Jerusarema Dance | https://www.youtube.com/watch?v=ltiDSchYzwY |
| **Zimbabwe** | Amandebele Traditional dance | https://www.youtube.com/watch?v=n7Bd91-4hhM |
| **Zimbabwe** | Zimbabwe traditional dance | https://www.youtube.com/watch?v=OaTyWQuaJ8w |
| **Zimbabwe** | Ndau festival - the pride of the Ndau people | https://www.youtube.com/watch?v=PFjgnSlvtmU |
| **Zimbabwe** | Dedza Gure wankulu - short African stories | https://www.youtube.com/watch?v=pHg6F_3ekaE&t |
| **Zimbabwe** | NYAU / GURE WANKHULU AT AMALINDA FARM ZIMBABWE | https://www.youtube.com/watch?v=rGOkLIXDOcI&t |
| **Zimbabwe** | Shona village in Masvingo, Zimbabwe | https://www.youtube.com/watch?v=RMjBriOtDPw |
| **Zimbabwe** | Zimbabwe traditional dance performance \| wedding | https://www.youtube.com/watch?v=sXWJpEsAorI&t |
| **Zimbabwe** | Ubuntu Cultural Festival ft. Claudia Ndlovu | https://www.youtube.com/watch?v=tot7IVSyNig&t |
| **Zimbabwe** | Great Zimbabwe | https://www.youtube.com/watch?v=Uq5abuVTI0E&t |
| **Zimbabwe** | Villa 2 Mupanyira nyau dancers, Mbare, Harare | https://www.youtube.com/watch?v=y0M5g03INYE&t |
| **Zimbabwe** | Zlm Dance Gala @33 Part 1 | https://www.youtube.com/watch?v=YA14ul9mDyM&t |
| **Zimbabwe** | Traditional Ndebele Dance moves | https://www.youtube.com/watch?v=zWmAgeDwz_c |

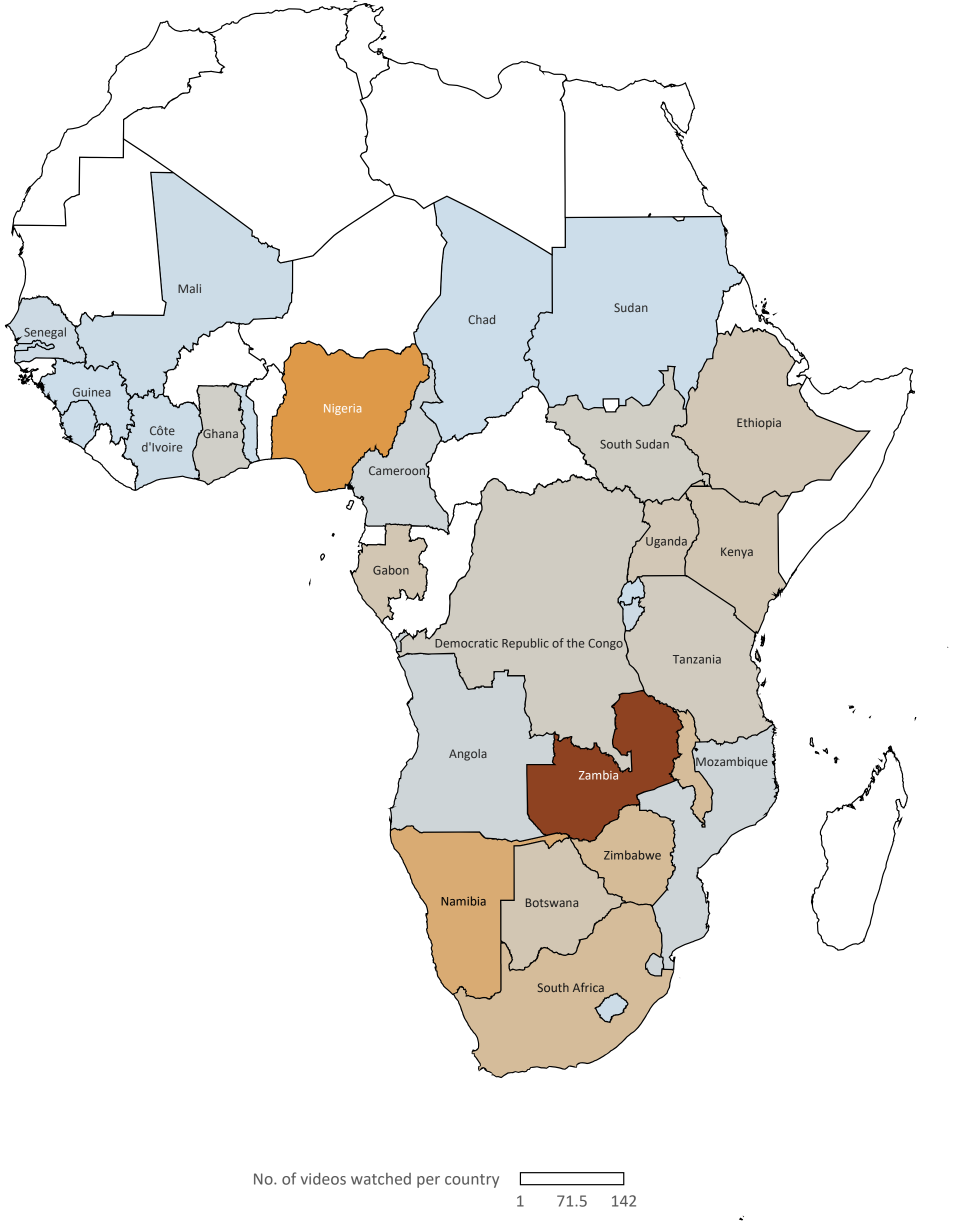


Number of videos watched per country, including videos that did not yield useable data for the review.

Data in the maps are from the above listed S3 Appendix. (Total: 555 videos for 33 countries)
