# Supplementary material for: Pan-African review of cultural uses of carnivores
Source: PLoS One. 2025 Mar 25;20(3):e0315903. doi: 10.1371/journal.pone.0315903 (PMC11936259; doi:10.1371/journal.pone.0315903)
Supplement: S1–S2 Figs — Additional supporting information on maximum adult body mass relative to the number of information sources per species per use category. (PDF) [file pone.0315903.s002.pdf]

Pan-African review of cultural uses of carnivores

Vivienne L. Williams, Marine Drouilly, Peter Coals, Gareth Whittington-Jones
(PLOS ONE)

Supporting Information

Figures: S1 and S2

Table of Contents

S1 FIG. **1**

S2A–I FIG. **3**

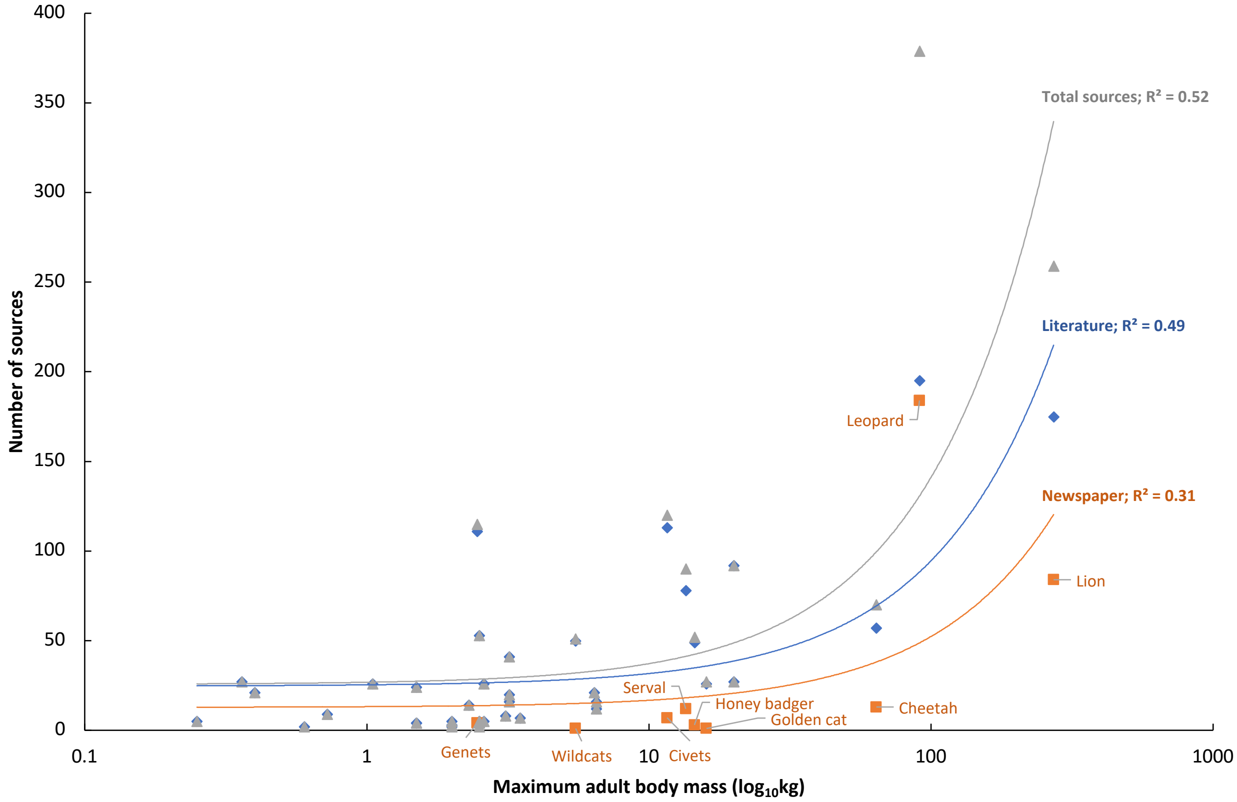

S1 Fig. Number of sources (*Newspaper, Literature, Total*) per species and morphospecies relative to the maximum adult body mass, where *Literature = journal + non-journal*, and *Total = Literature + Newspaper*. Morphospecies mass is the average for the taxa. Only taxa reported in *Newspapers* are labelled, and their corresponding values for *Literature* and *Total* are at the same x-axis value above them. See Fig 6 for grey data point labels for all taxa for 'Total'. Data are for all 11 use categories.

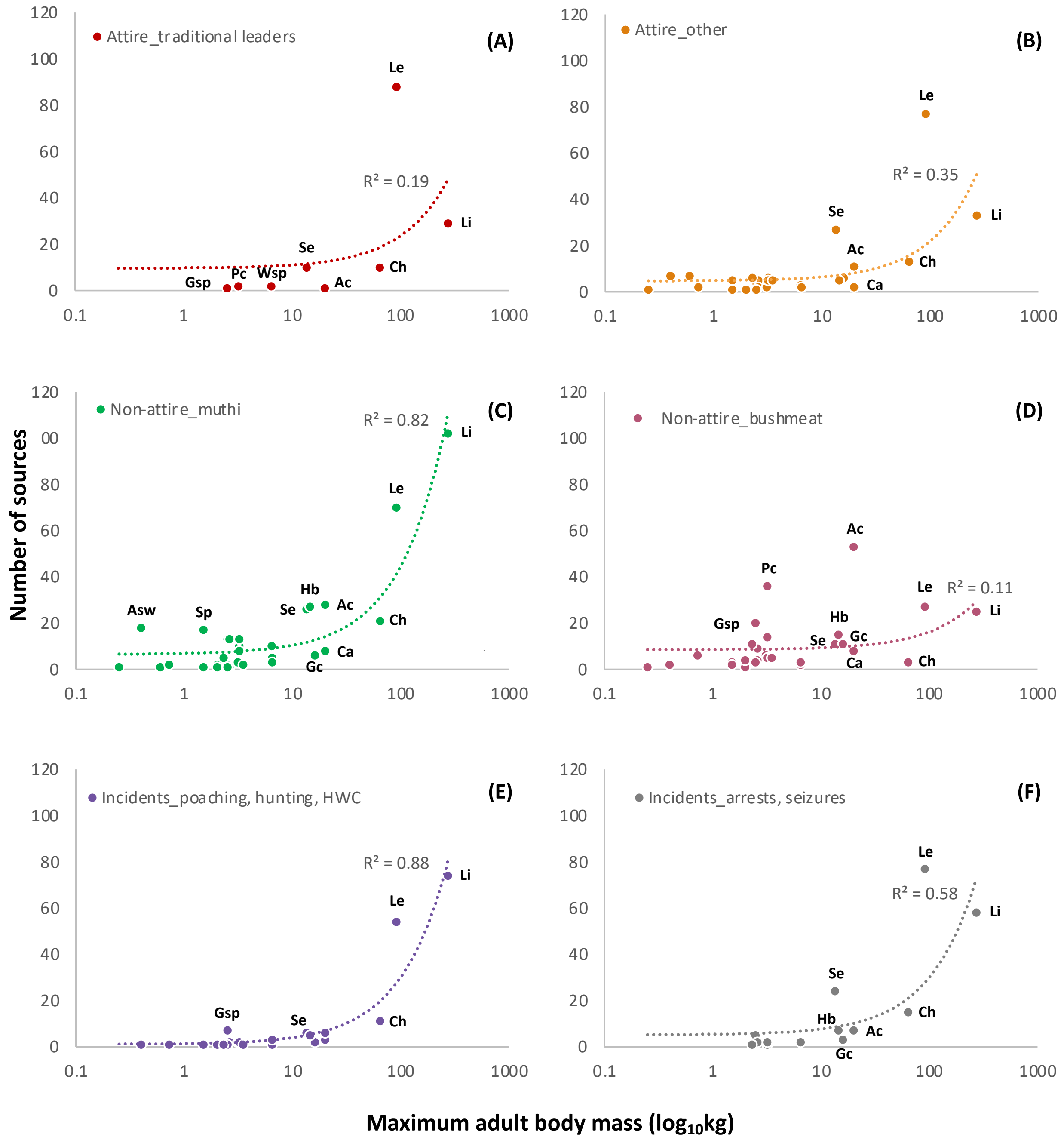

(Caption on following page)

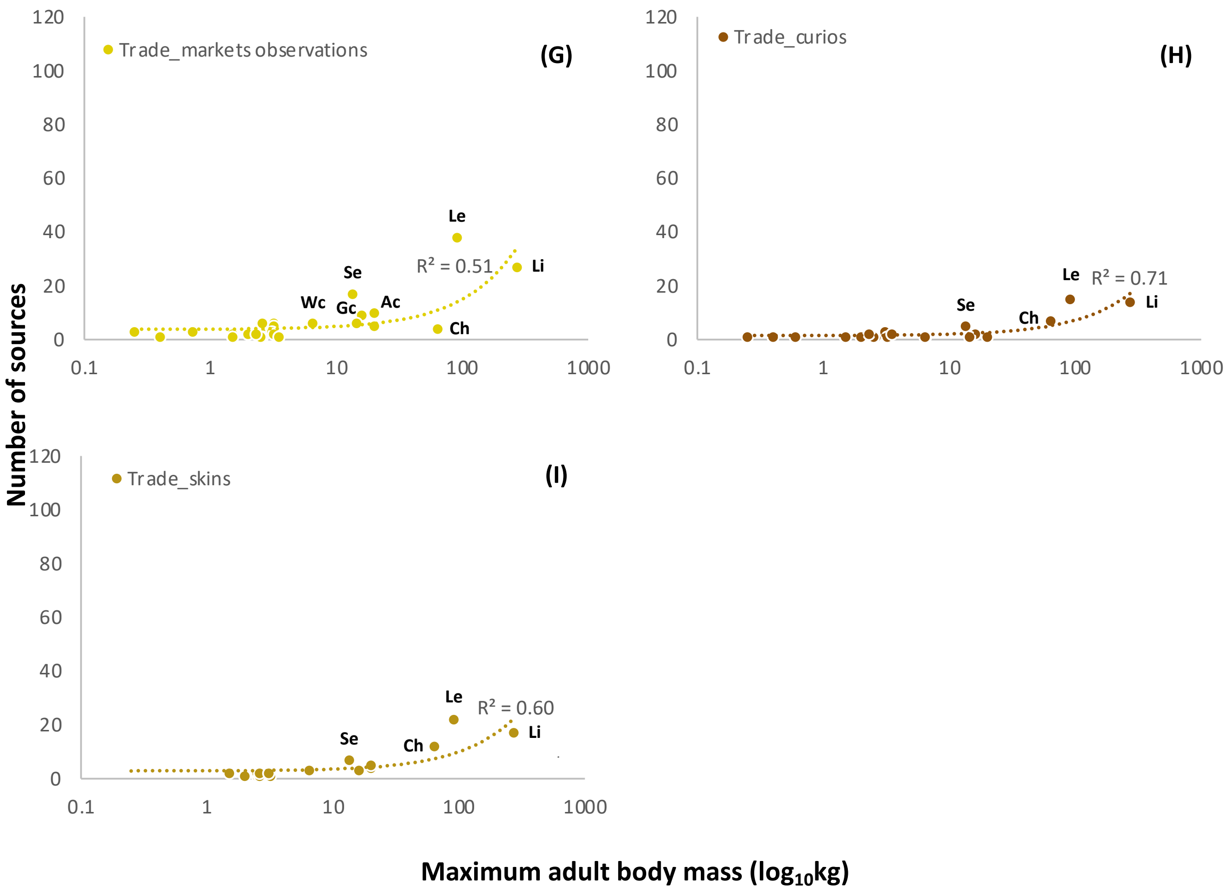

S2A–I Fig. Number of sources for categories of use, trade and incidents per species and morphospecies relative to the maximum adult body mass, where *Total = Literature + Newspaper*. Morphospecies mass is the average for the taxa. Only categories with >25 sources are graphed (hence, we excluded political leaders, religious groups, non-specific cultural use, and musk).

Abbreviations:

Ac = African civet
Asw = African striped weasel
Ca = Caracal
Ch = Cheetah
Gc = Golden cat
Gsp = Genet species
Hb = Honey badger
Le= Leopard
Li = Lion
Pc = Palm civet
Se = Serval
Sp = Striped polecat
Wc = African wild cat
Wsp = Wildcat species
