# Supplementary material for: Pan-African review of cultural uses of carnivores
Source: PLoS One. 2025 Mar 25;20(3):e0315903. doi: 10.1371/journal.pone.0315903 (PMC11936259; doi:10.1371/journal.pone.0315903)
Supplement: S1–S2 Maps — Full-size maps for 36 taxa, showing geospatial evidence for the cultural use and trade of carnivore species and morphospecies across Africa, relative to their occurrence in a country. (PDF) [file pone.0315903.s003.pdf]

Pan-African review of cultural uses of carnivores

Vivienne L. Williams, Marine Drouilly, Peter Coals, Gareth Whittington-Jones
(PLOS ONE)

Supporting Information

Maps: S1 and S2.1 to S2.36

Table of Contents

S1 MAP. AFRICAN COUNTRIES AND REGIONS. 3

S2 MAP (S2.1 TO S2.36). 4

S2.1 MAP. LEOPARD 4

S2.2 MAP. LION 5

S2.3 MAP. CHEETAH 6

S2.4 MAP. AFRICAN GOLDEN CAT 7

S2.5 MAP. CARACAL 8

S2.6 MAP. SERVAL 9

S2.7 MAP. WILDCATS *Felis lybica lybica* & *F. lybica cafra* 10

S2.8 MAP. BLACK-FOOTED CAT 11

S2.9 MAP. HONEY BADGER 12

S2.10 MAP. POLECATS *Ictonyx striatus* & *I. libycus* 13

S2.11 MAP. STRIPED POLECAT 14

S2.12 MAP. SAHARAN STRIPED POLECAT 15

S2.13 MAP. WEASELS *Poecilogale albinucha* & *Mustela nivalis* 16

S2.14 MAP. AFRICAN STRIPED WEASEL 17

S2.15 MAP. LEAST WEASEL 18

S2.16 MAP. CIVETS *Civettictis civetta* & *Nandinia binotata* 19

S2.17 MAP. CIVET unknown species 20

S2.18 MAP. AFRICAN CIVET 21

S2.19 MAP. AFRICAN PALM CIVET 22

S2.20 MAP. GENETS 14 species 23

S2.21 MAP. GENETS unknown species 24

S2.22 MAP. ETHIOPIAN GENET 25

S2.23 MAP. ANGOLAN GENET 26

S2.24 MAP. BOURLON'S GENET 27

S2.25 MAP. CRESTED GENET 28
S2.26 MAP. SMALL-SPOTTED GENET 29
S2.27 MAP. JOHNSTON'S GENET 30
S2.28 MAP. LARGE-SPOTTED GENET 31
S2.29 MAP. PARDINE GENET 32
S2.30 MAP. AQUATIC GENET 33
S2.31 MAP. KING GENET 34
S2.32 MAP. SERVALINE GENET 35
S2.33 MAP. HAUSA GENET 36
S2.34 MAP. CAPE GENET 37
S2.35 MAP. GIANT GENET 38
S2.36 MAP. LINSANGS *Poiana leightoni* & *P. richardsonii* 39

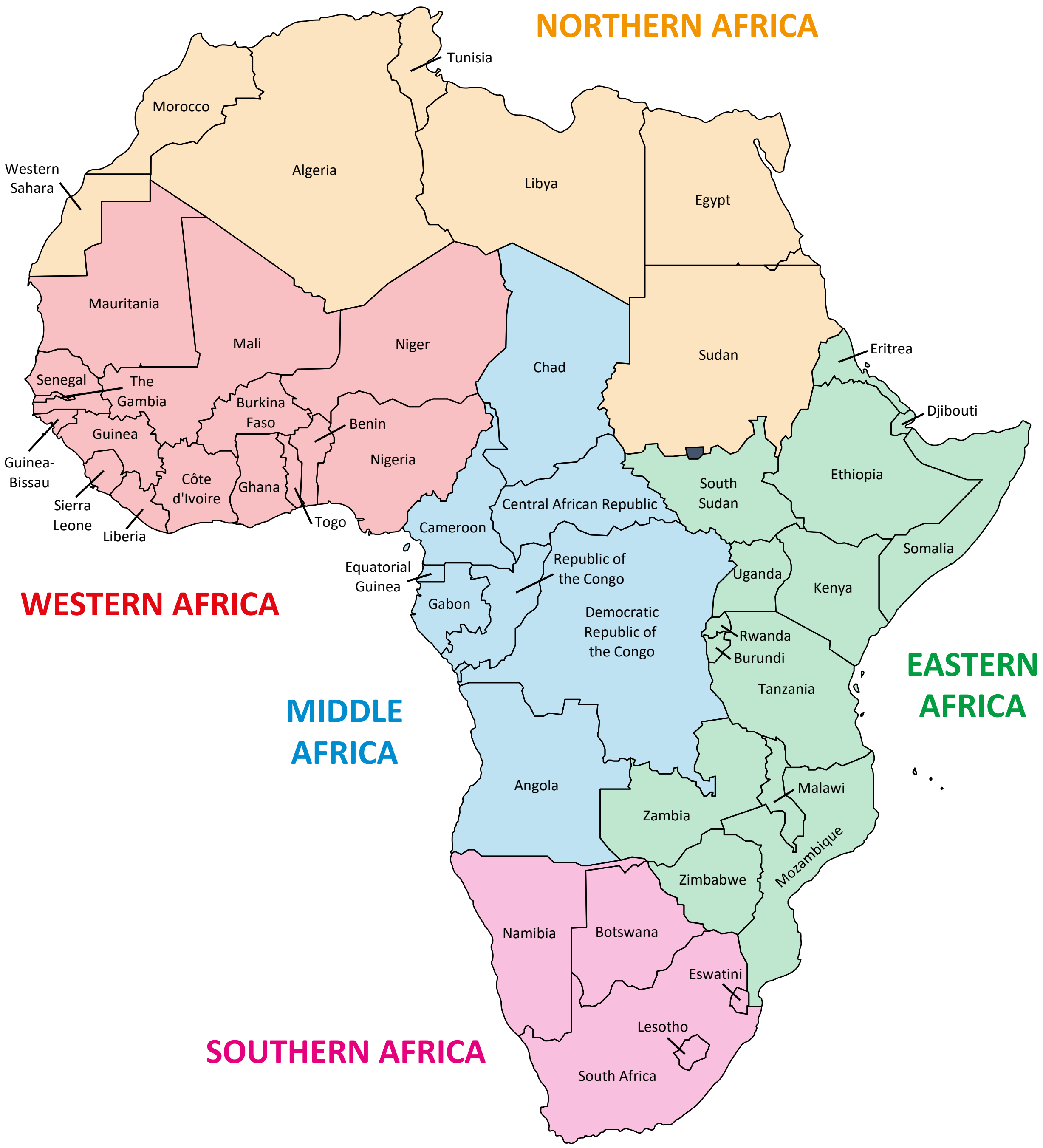

S1 Map. African Countries and regions. The regions are delineated according to the United Nations Geoscheme https://unstats.un.org/unsd/methodology/m49/. Colours for regions are consistent throughout the paper.

S2 Map (S2.1 to S2.36).

Geospatial mapping of the evidence for cultural use and trade of carnivore species and morphospecies across Africa: literature survey and video evidence (white font with asterisks) by country, with record counts. White asterisks indicate video evidence only. Country colours indicate species occurrence statuses and whether records were found during the review. $n_{countries}$ = country count; $n_{records}$ = record count.

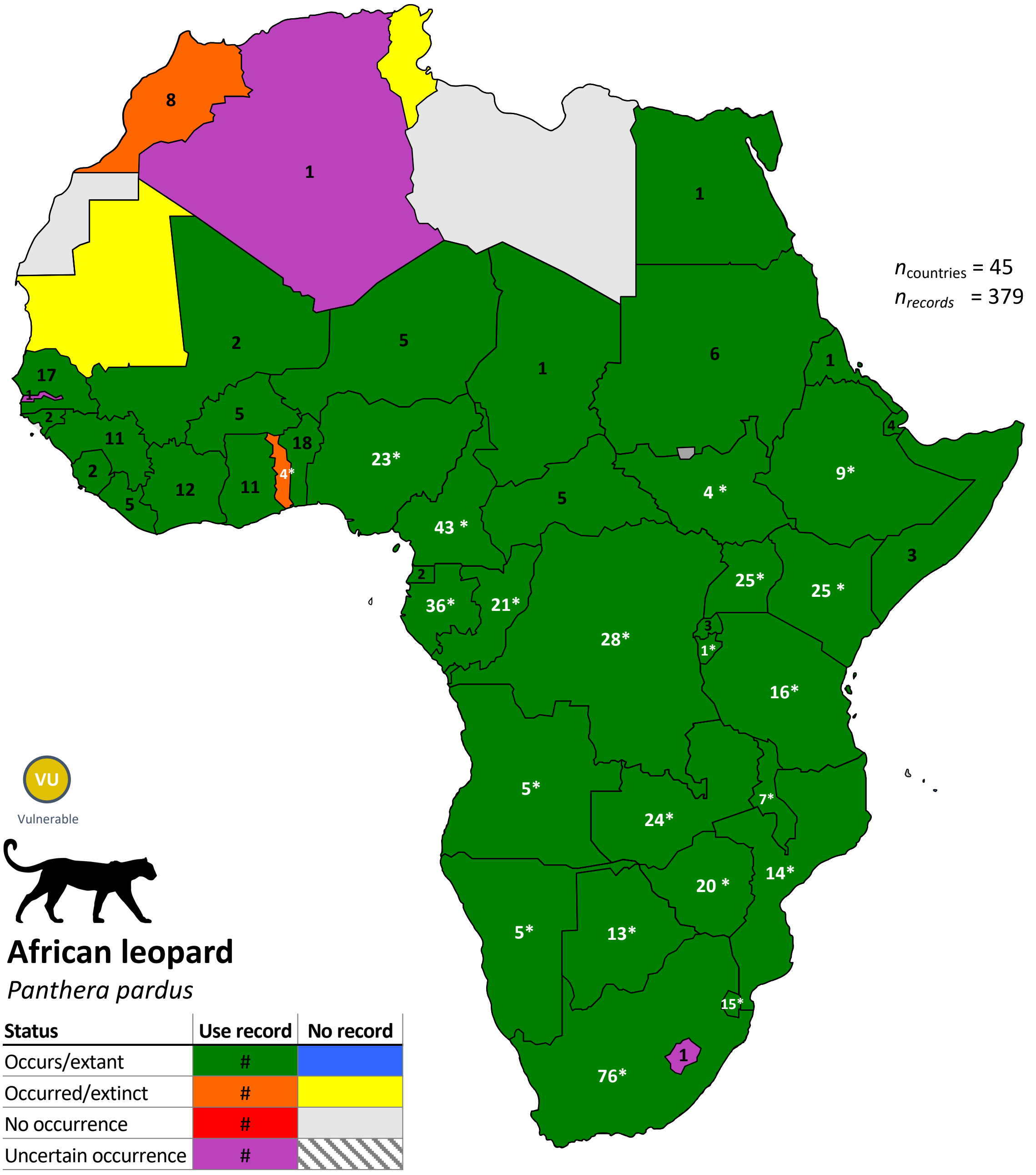

S2.1 Map. Leopard

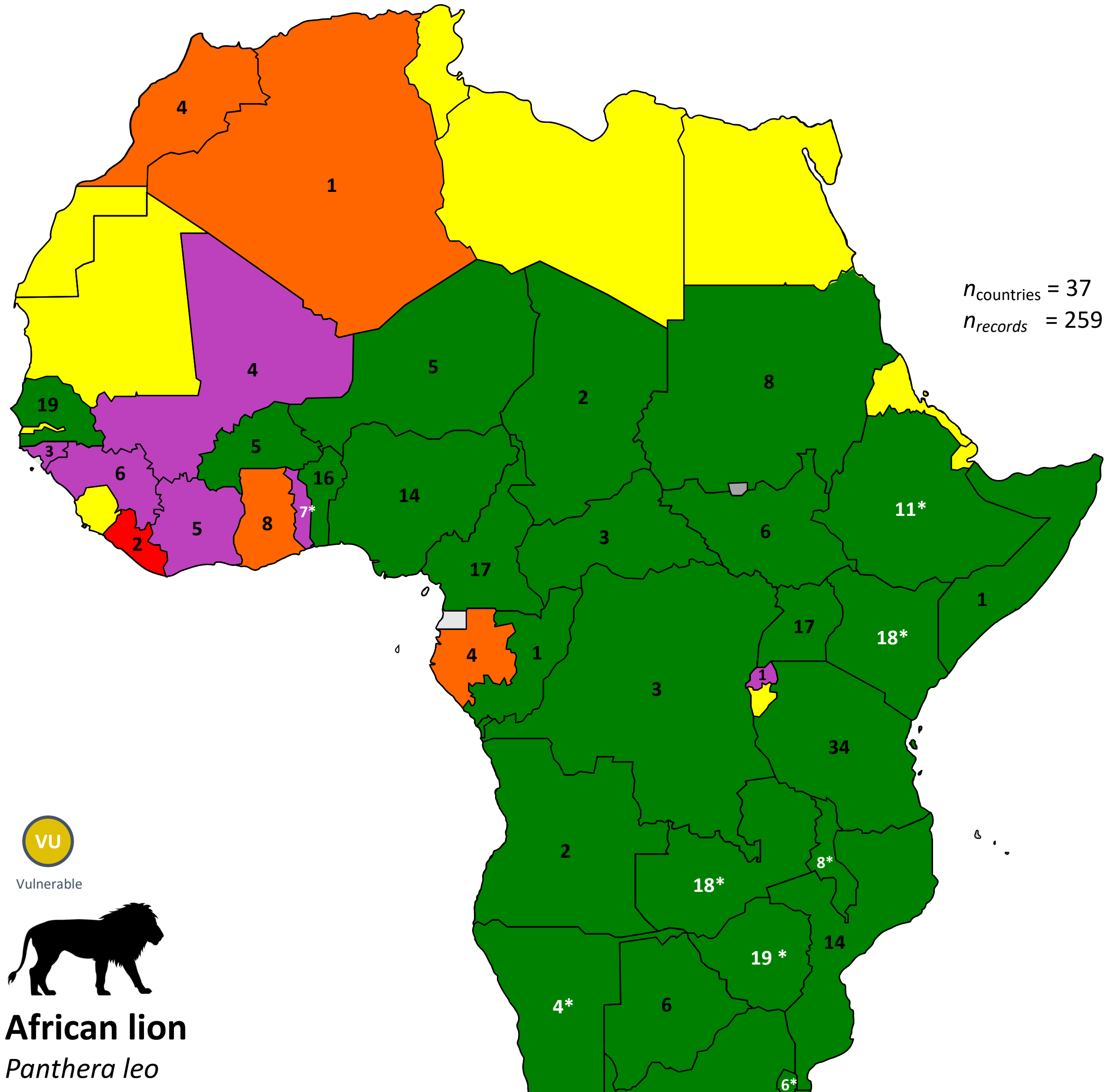

Status	Use record	No record
Occurs/extant	#	
Occurred/extinct	#	
No occurrence	#	
Uncertain occurrence	#	

S2.2 Map. Lion

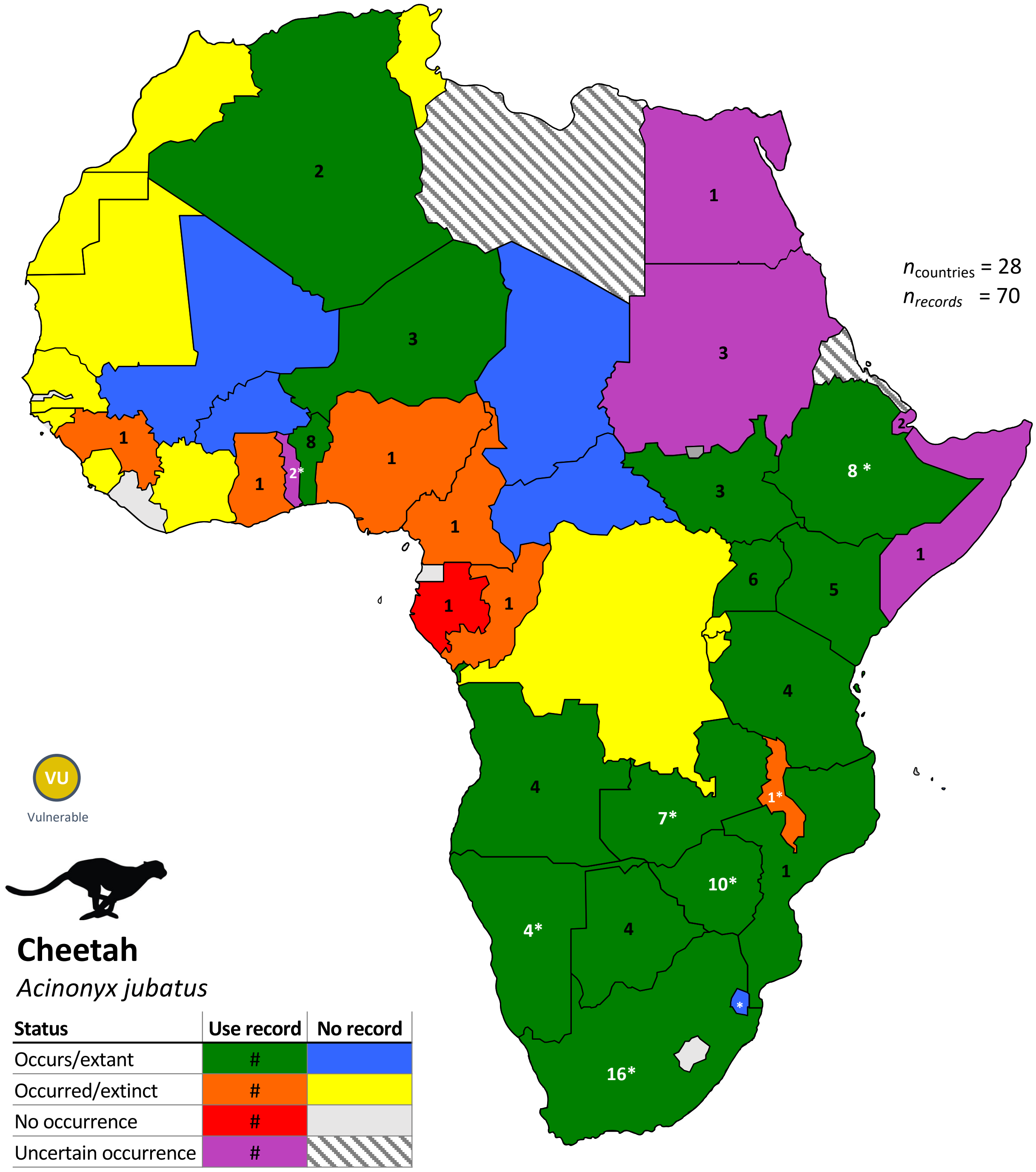

S2.3 Map. Cheetah

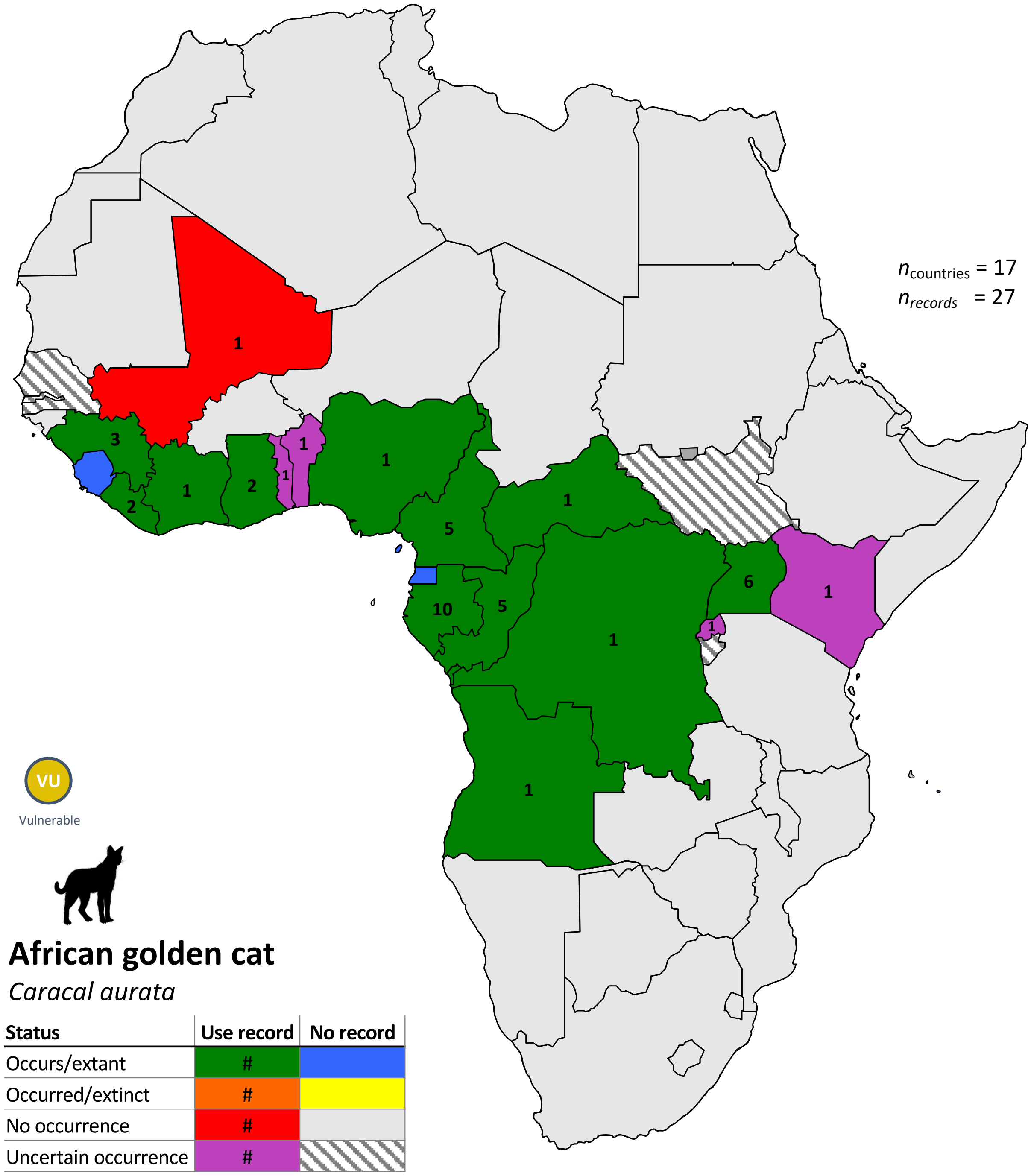

S2.4 Map. African golden cat

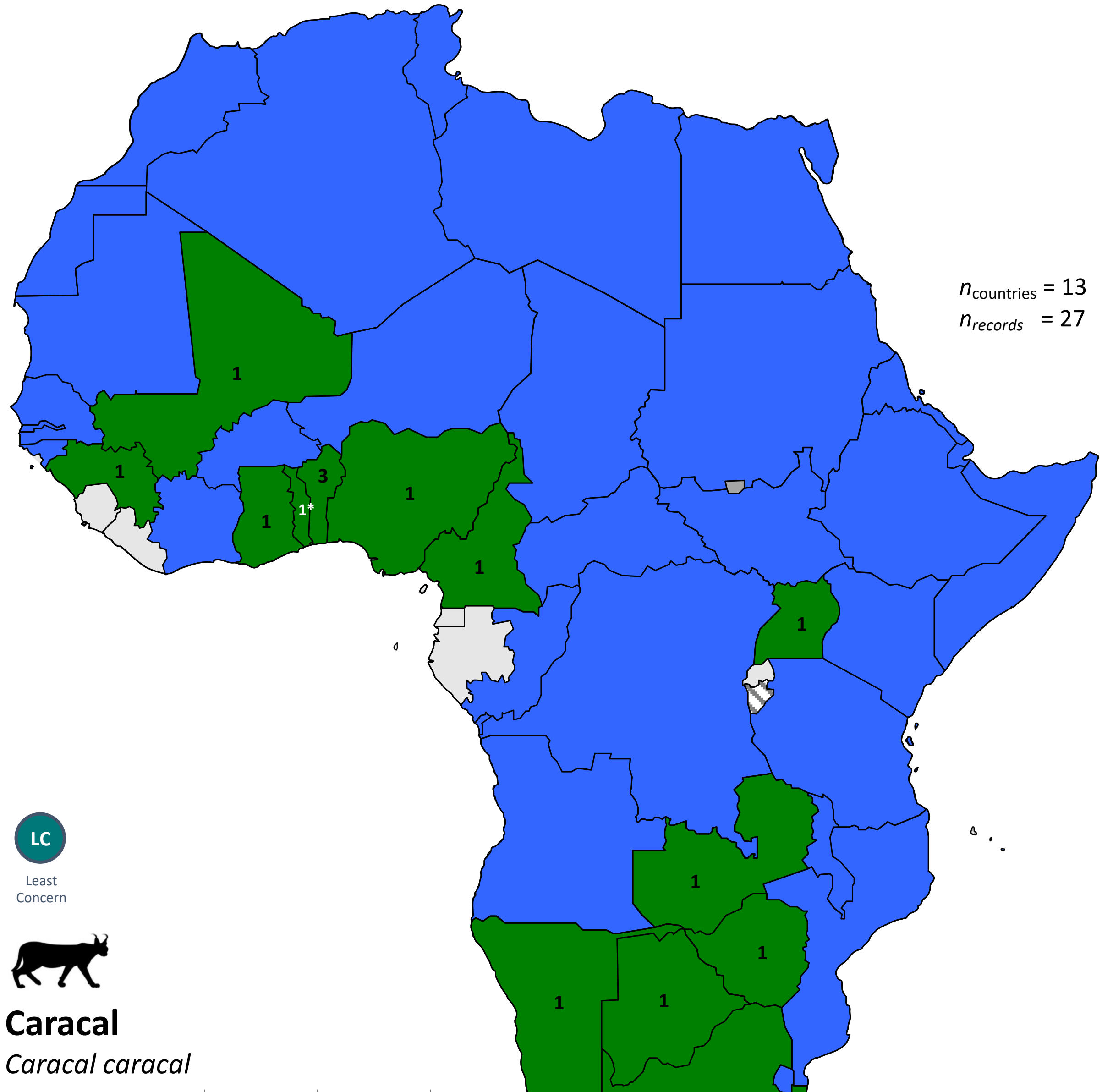

Status	Use record	No record
Occurs/extant	#	
Occurred/extinct	#	
No occurrence	#	
Uncertain occurrence	#	

S2.5 Map. Caracal

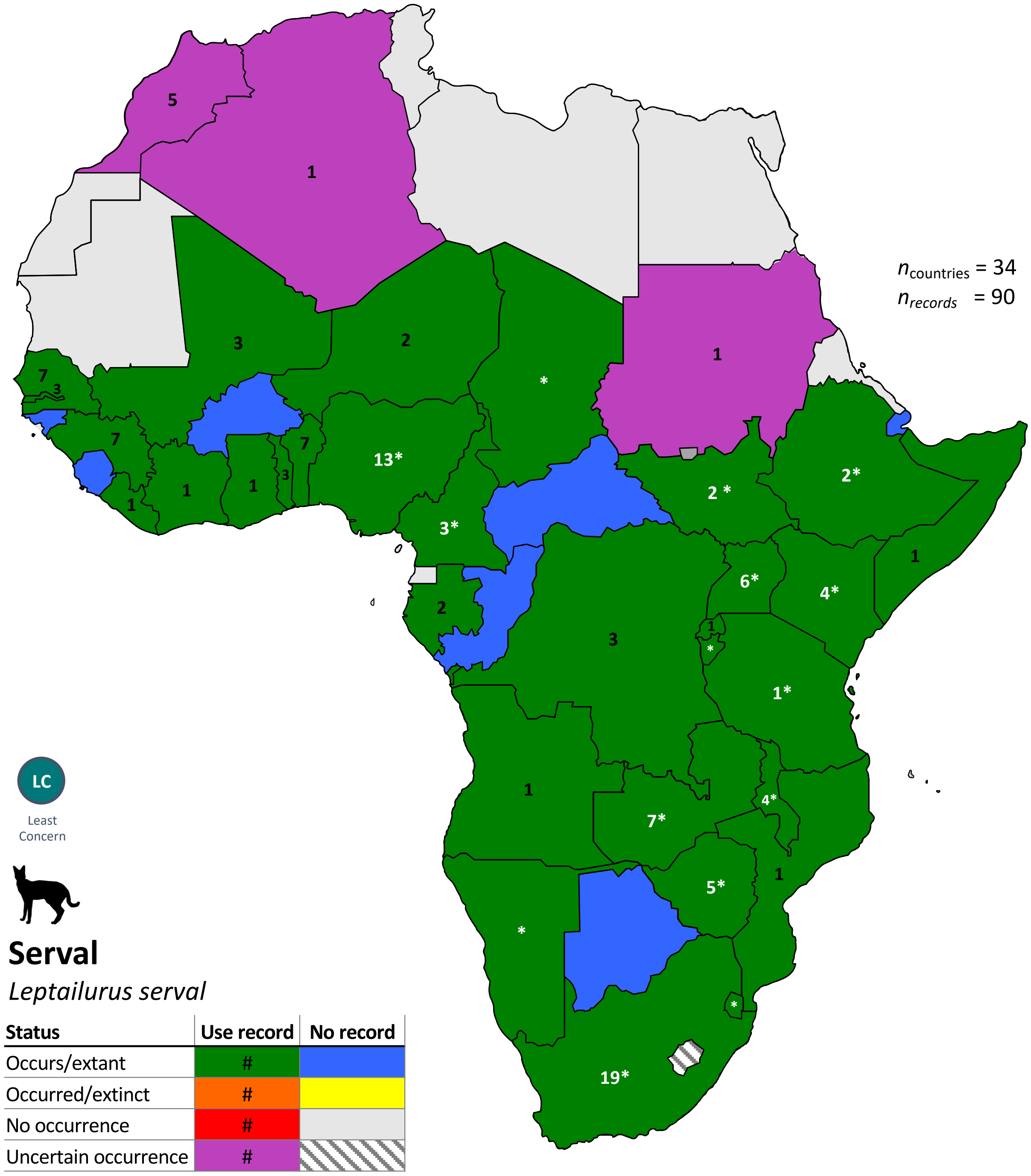

S2.6 Map. Serval

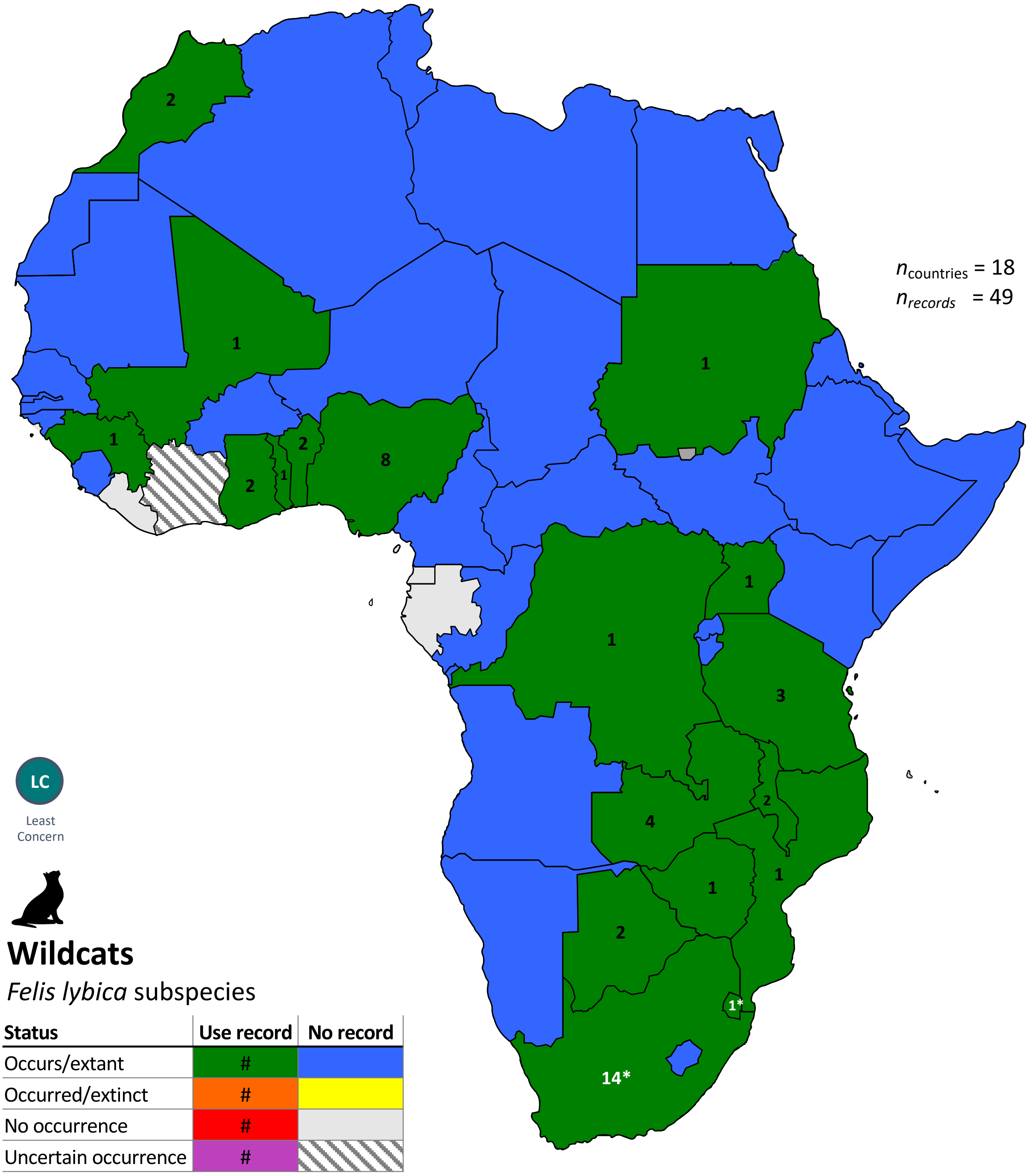

S2.7 Map. Wildcats *Felis lybica lybica* & *F. lybica cafra*

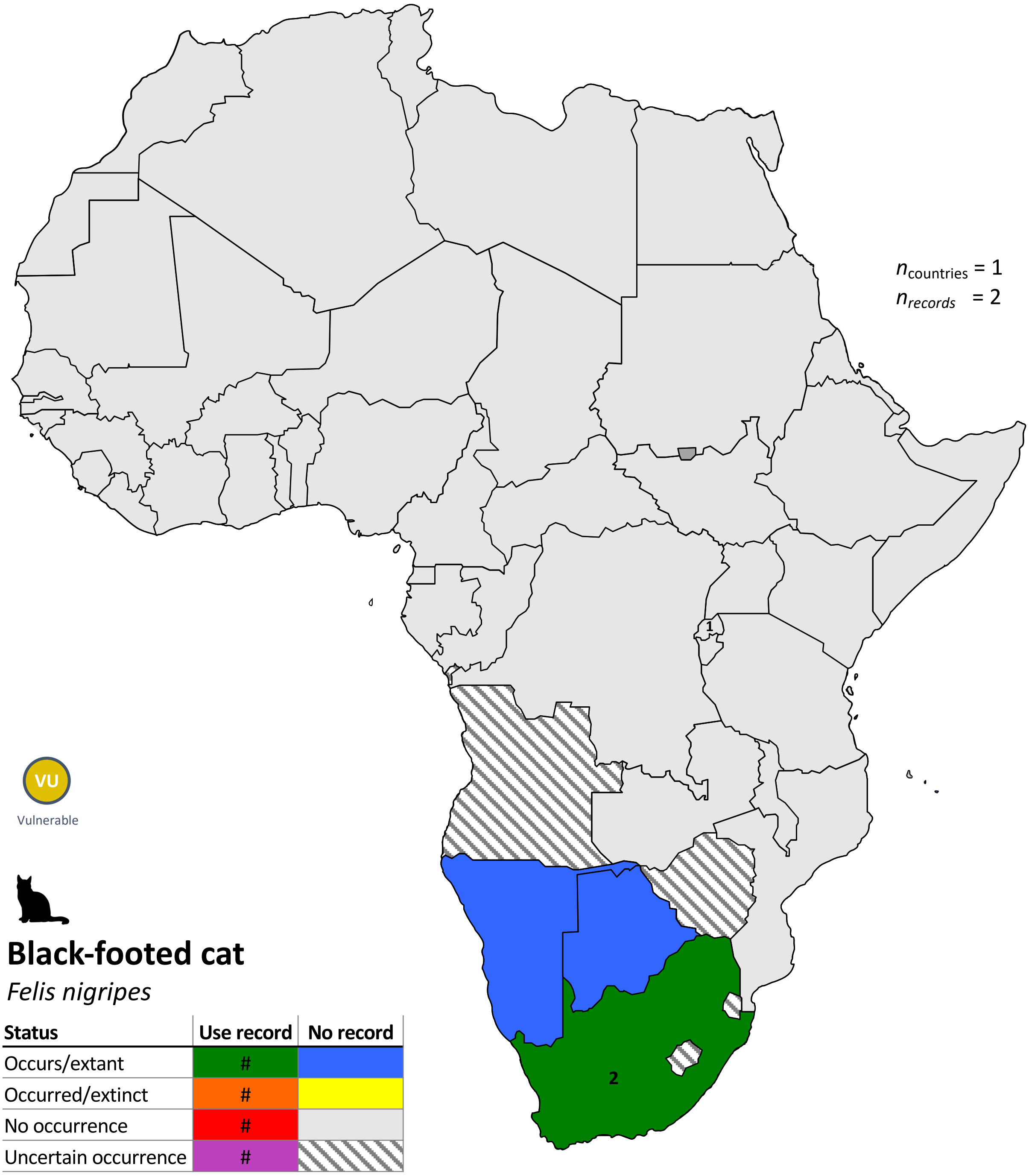

S2.8 Map. Black-footed cat

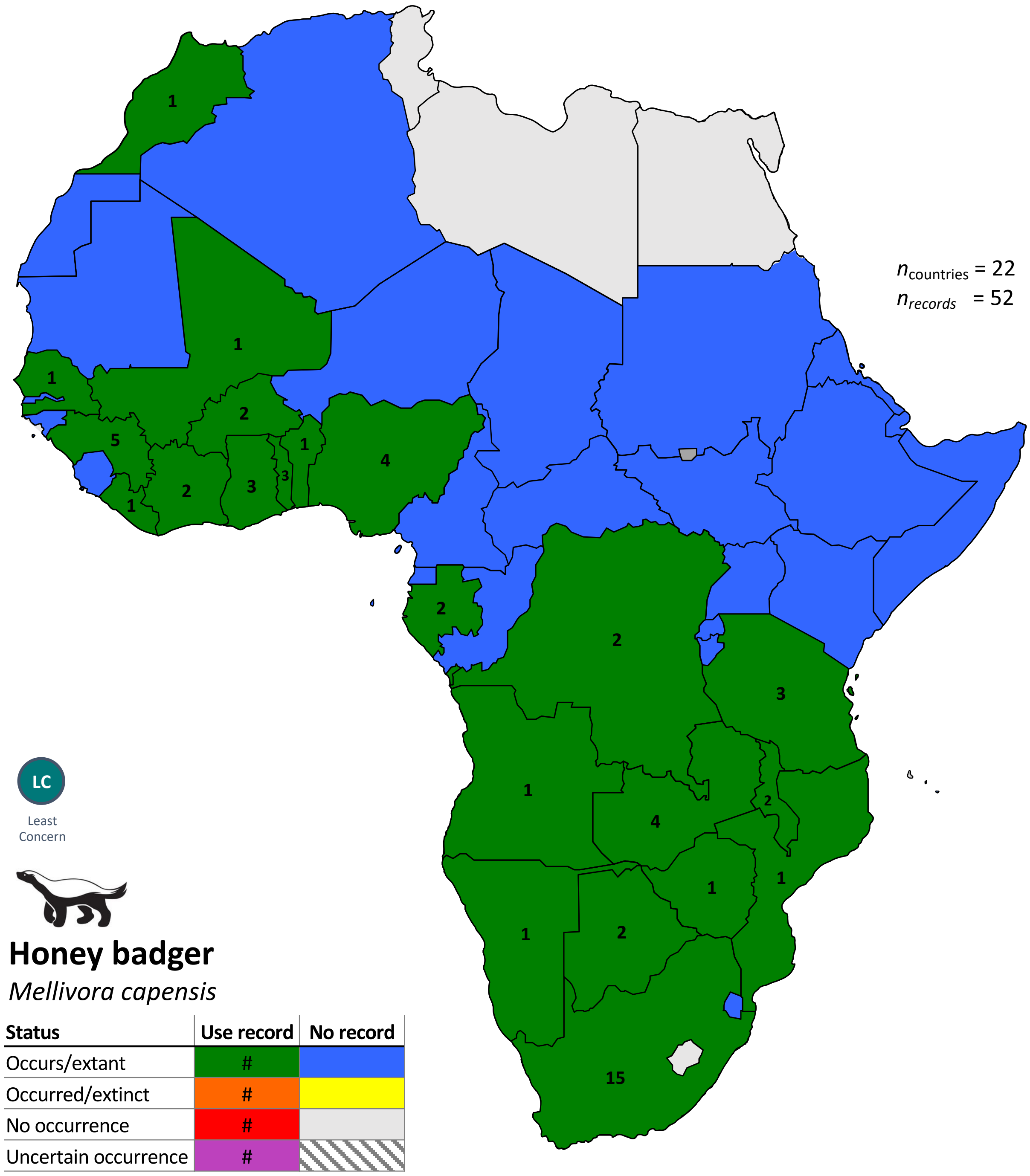

S2.9 Map. Honey badger

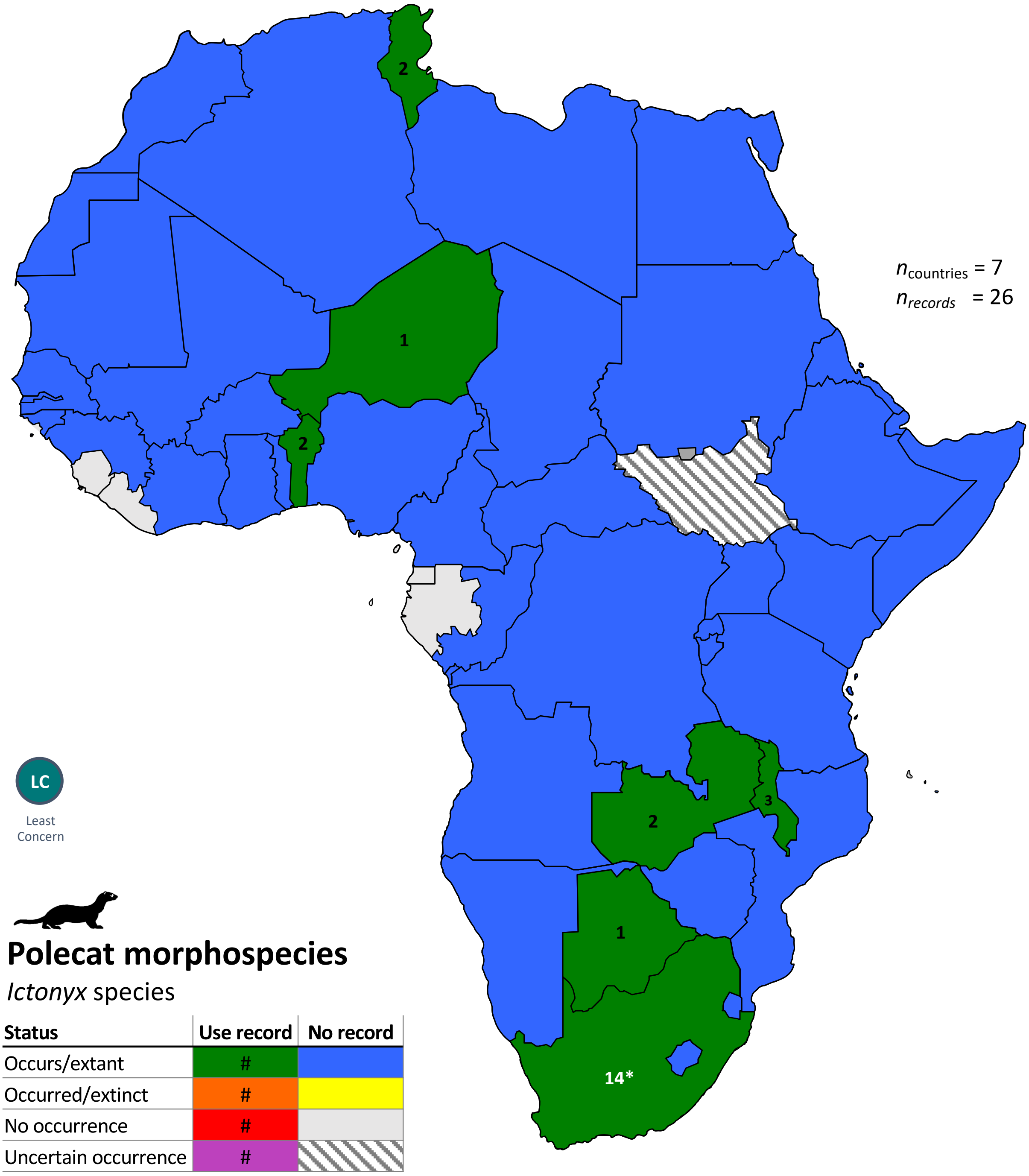

S2.10 Map. Polecats ***Ictonyx striatus*** **&** ***I. libycus***

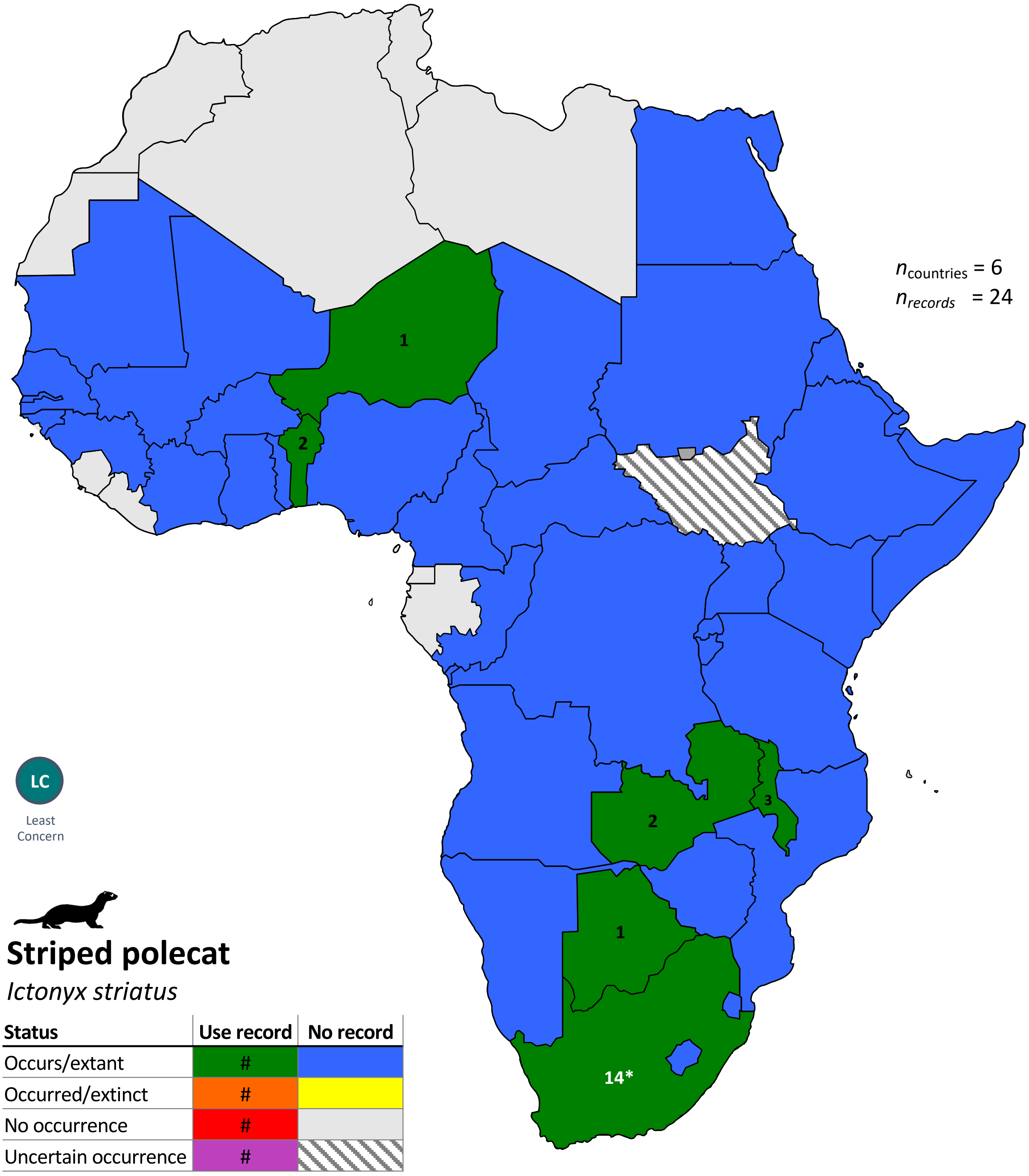

S2.11 Map. Striped polecat

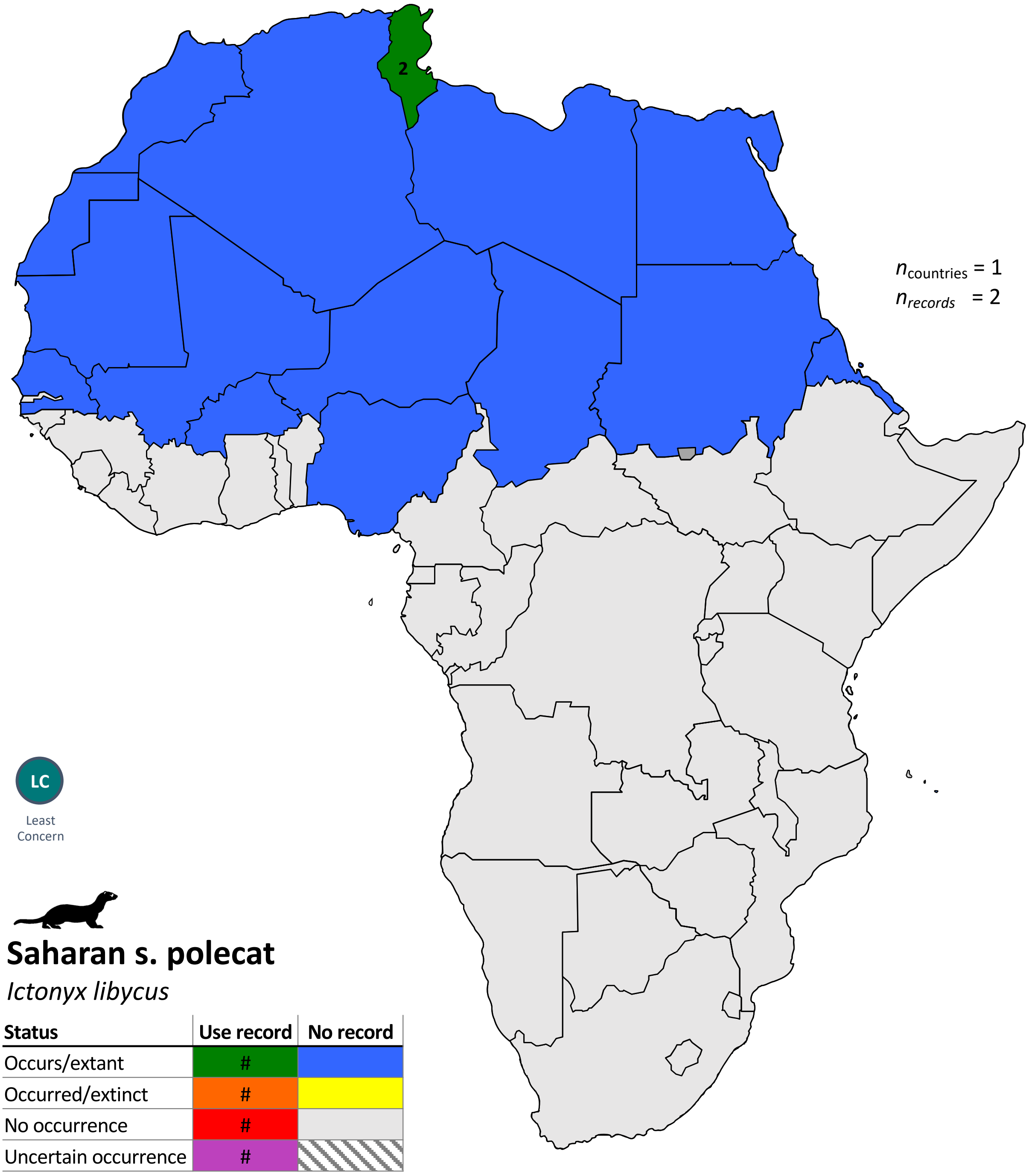

S2.12 Map. Saharan striped polecat

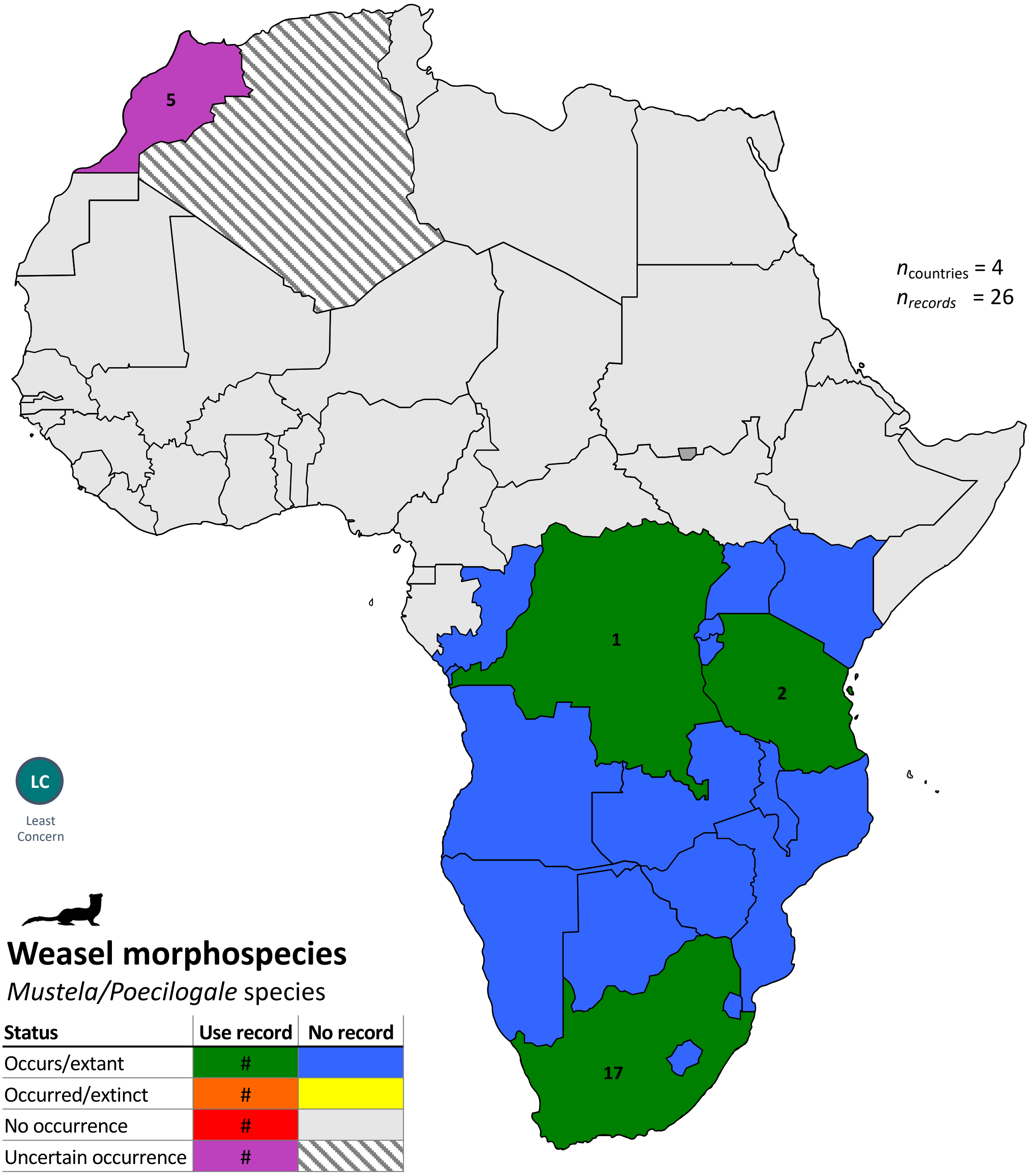

S2.13 Map. Weasels ***Poecilogale albinucha*** **&** ***Mustela nivalis***

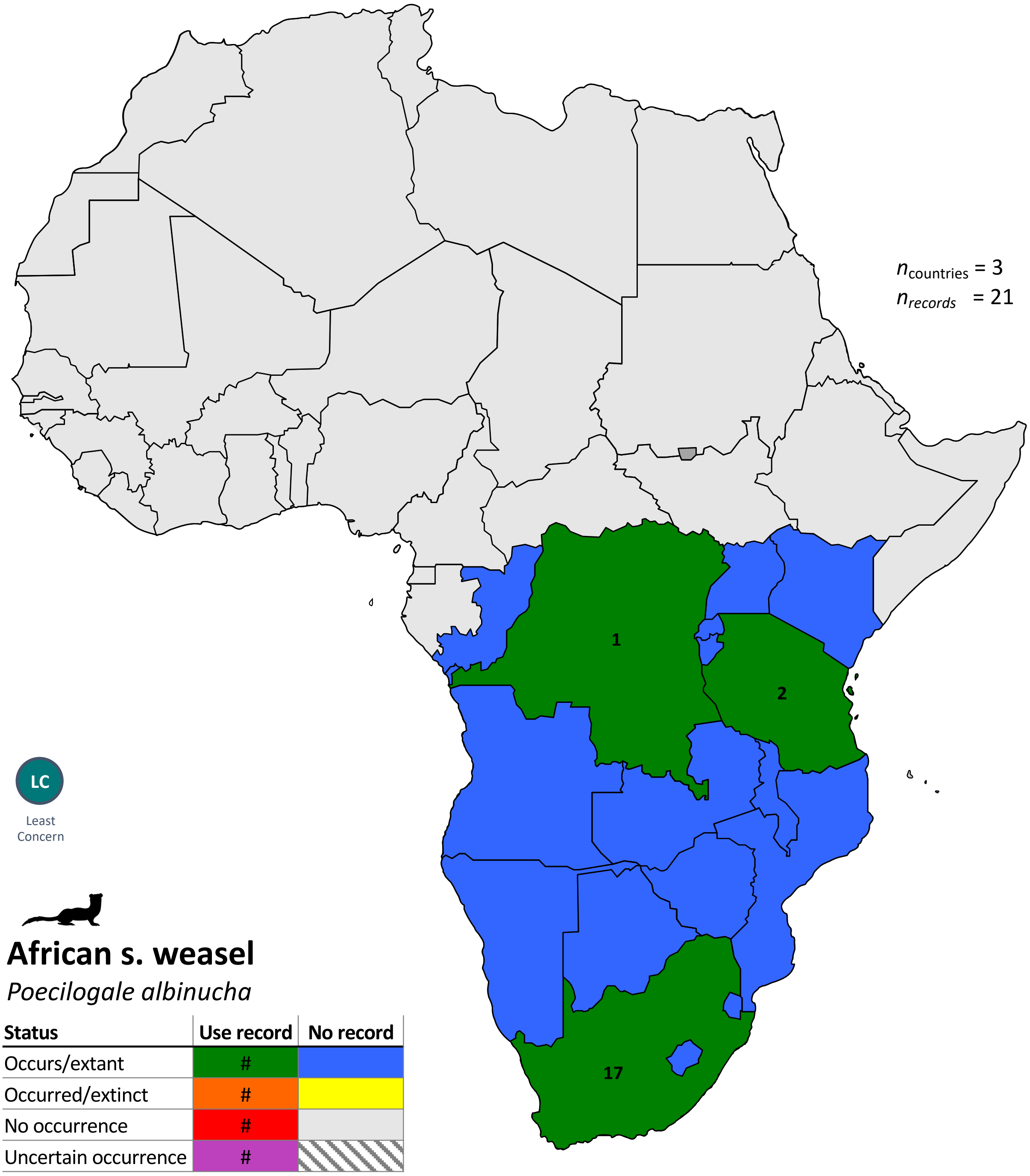

S2.14 Map. African striped weasel

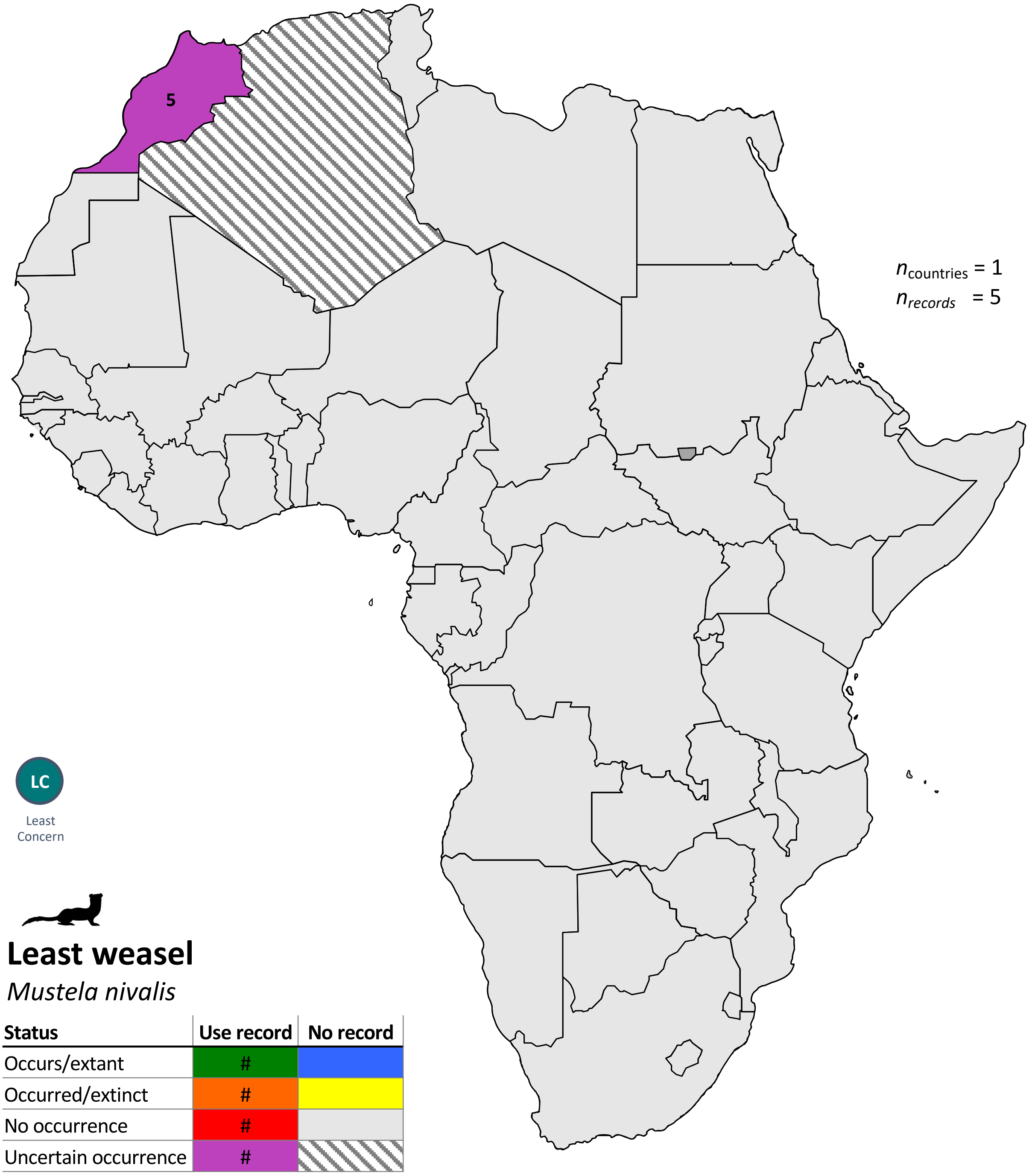

S2.15 Map. Least weasel

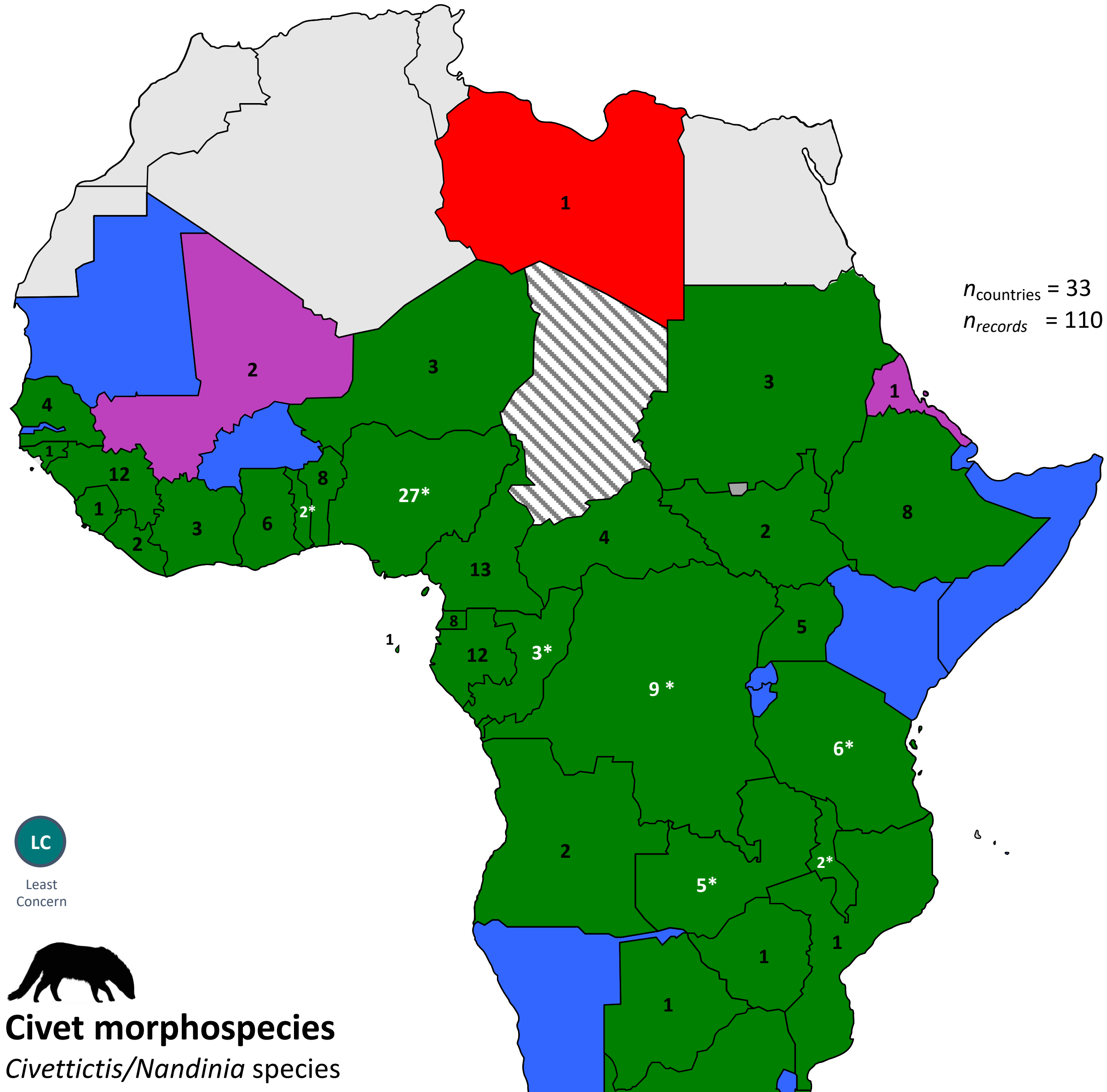

Status	Use record	No record
Occurs/extant	#	
Occurred/extinct	#	
No occurrence	#	
Uncertain occurrence	#	

S2.16 Map. Civets *Civettictis civetta* & *Nandinia binotata*

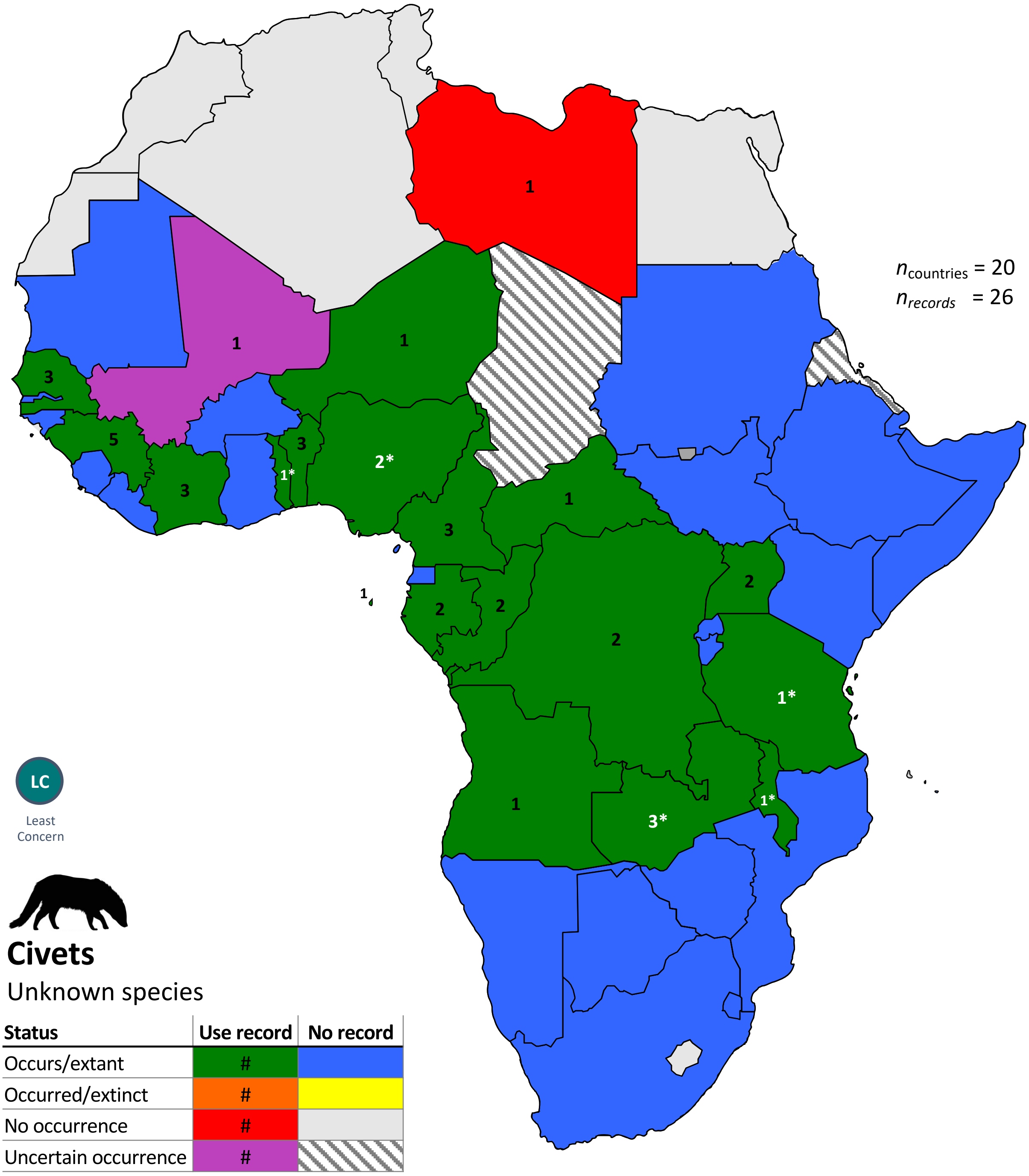

S2.17 Map. Civet unknown species

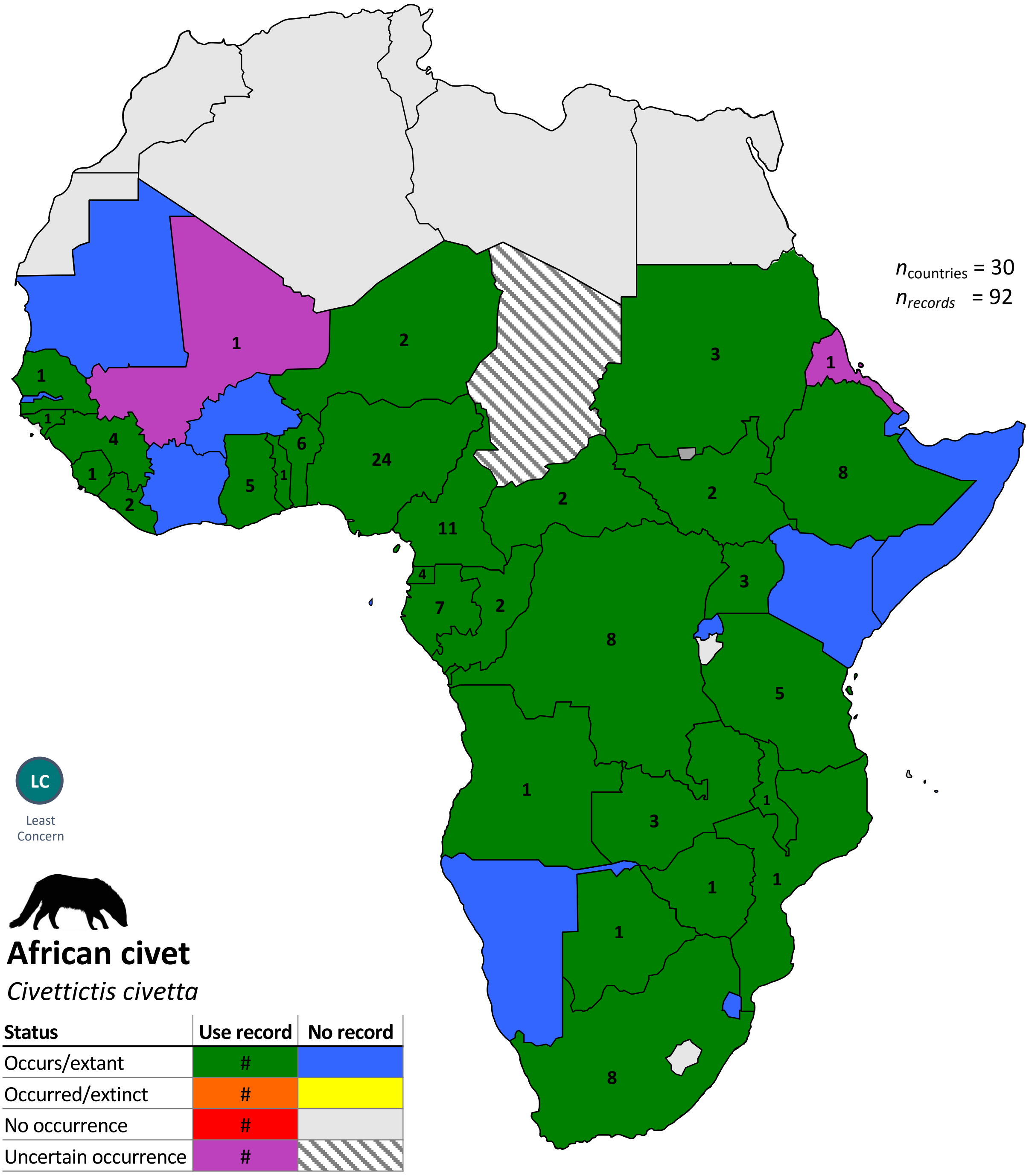

S2.18 Map. African civet

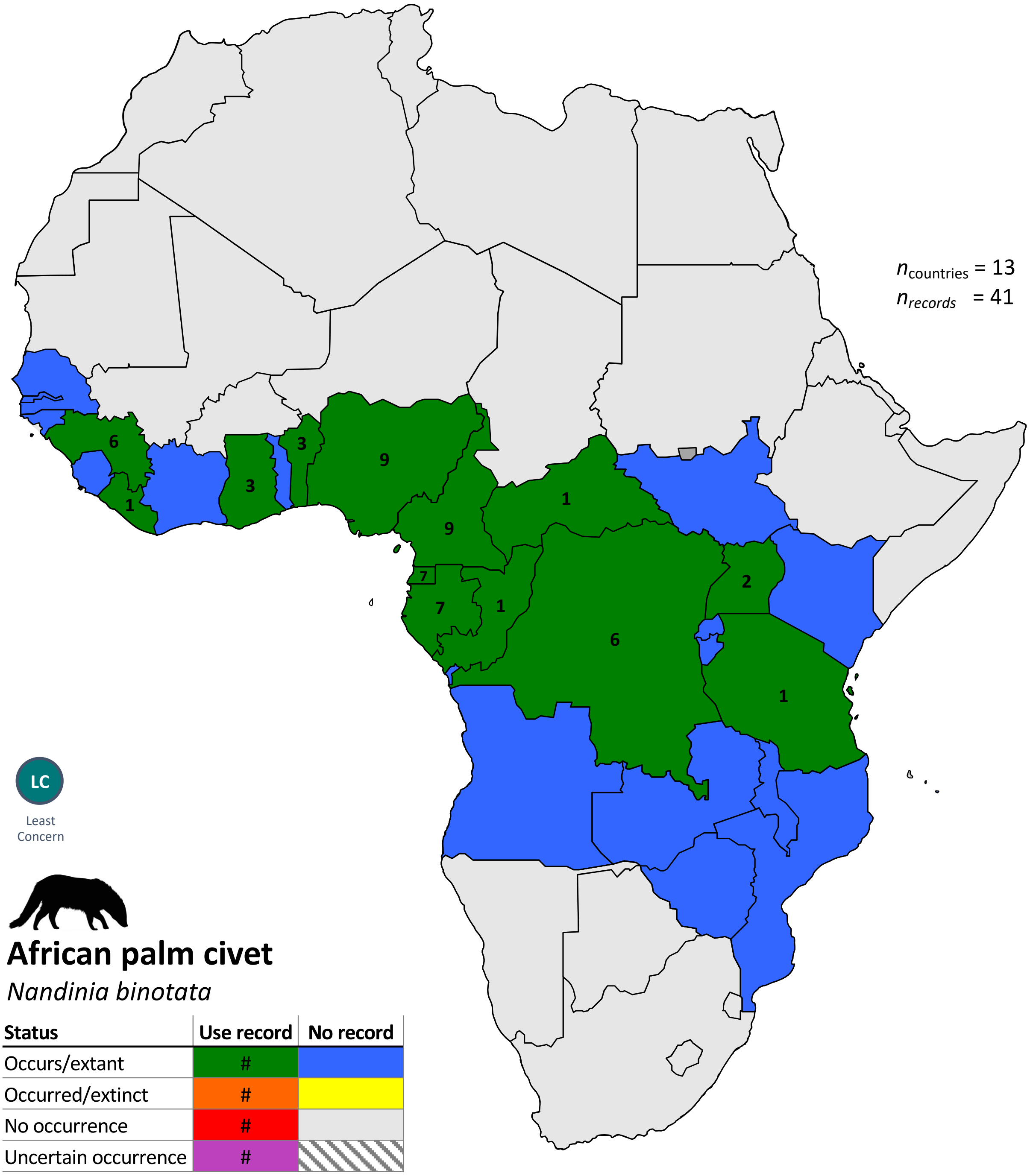

S2.19 Map. African palm civet

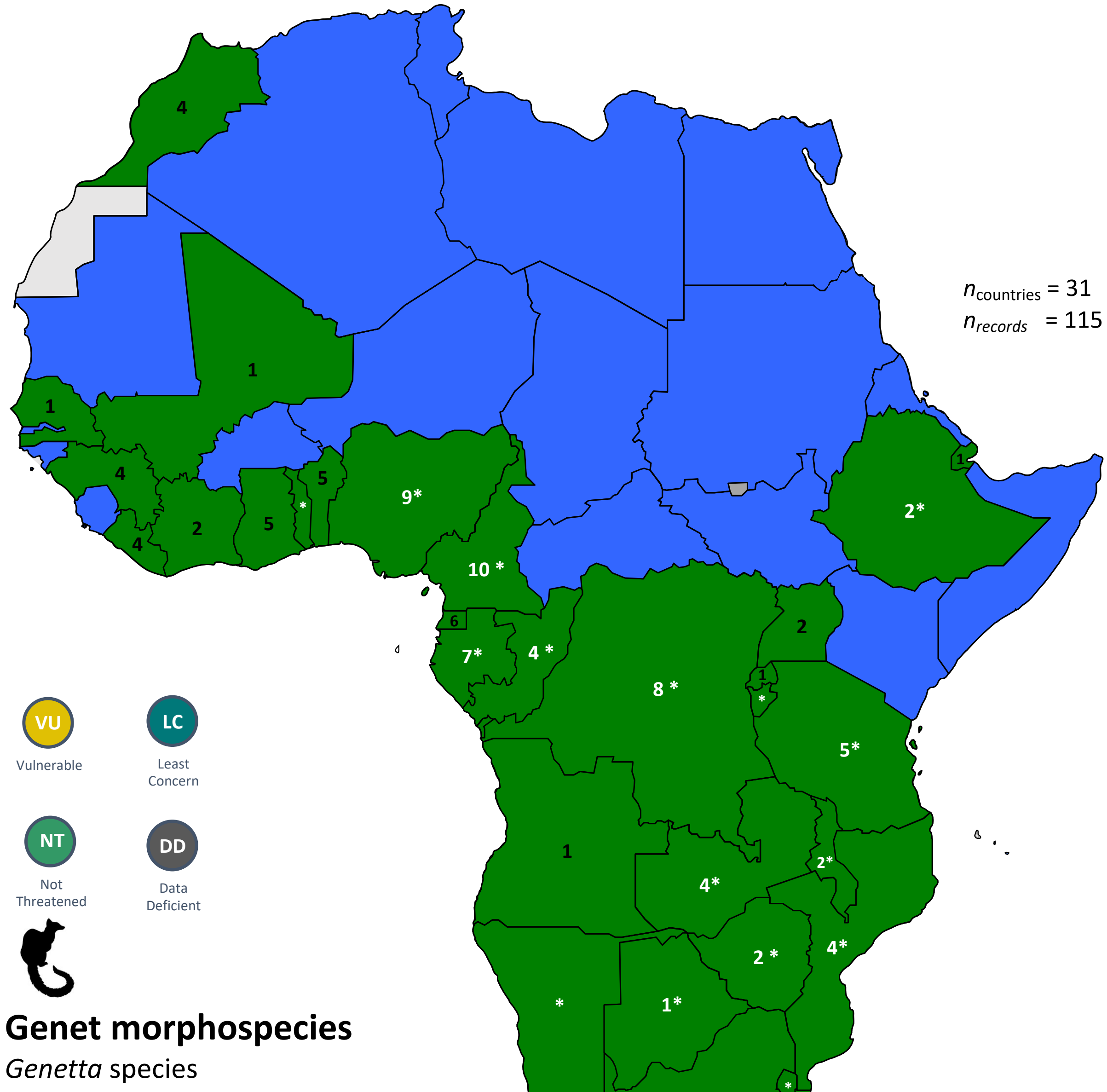

Status	Use record	No record
Occurs/extant	#	
Occurred/extinct	#	
No occurrence	#	
Uncertain occurrence	#	

S2.20 Map. Genets 14 species

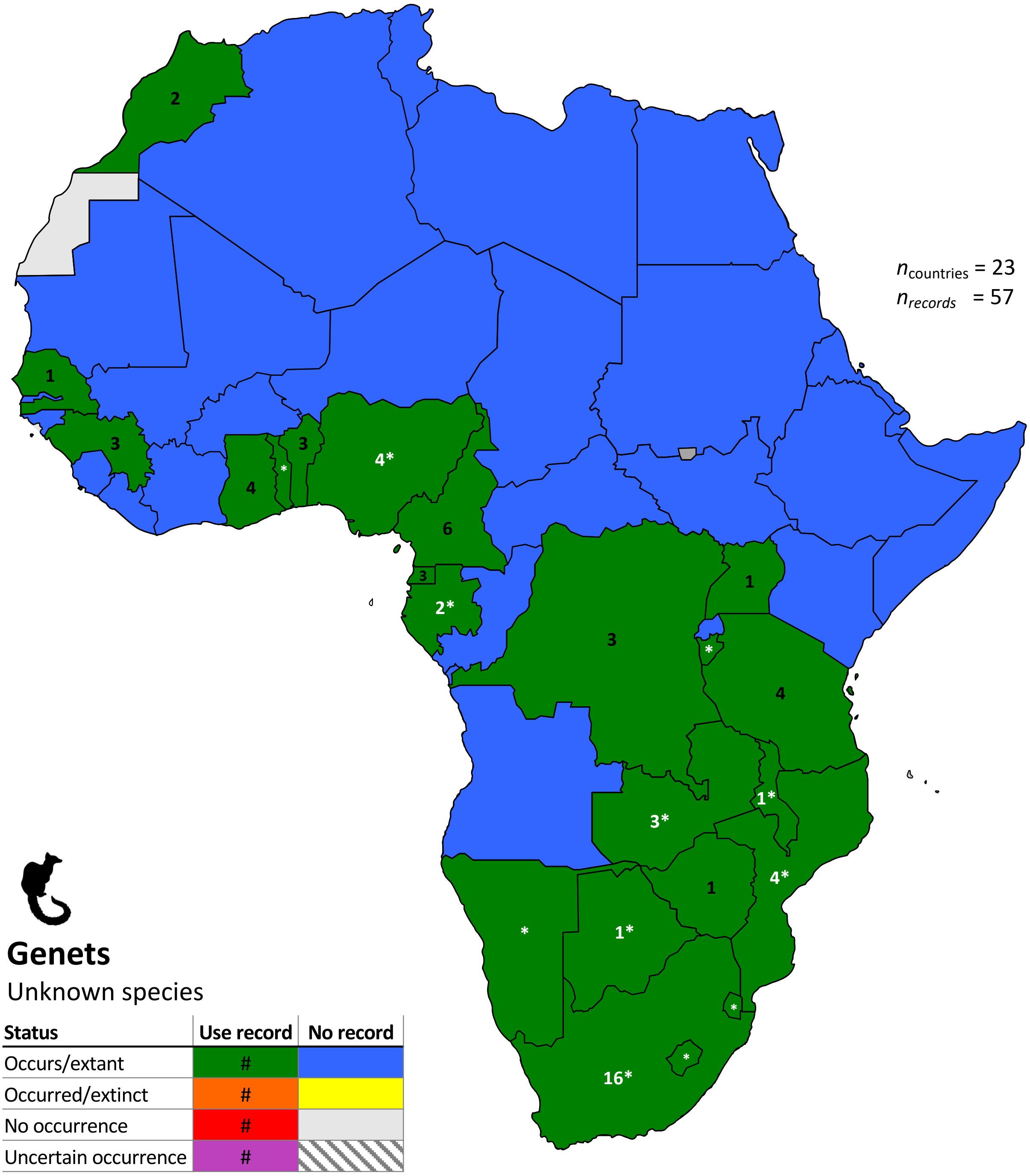

S2.21 Map. Genets unknown species

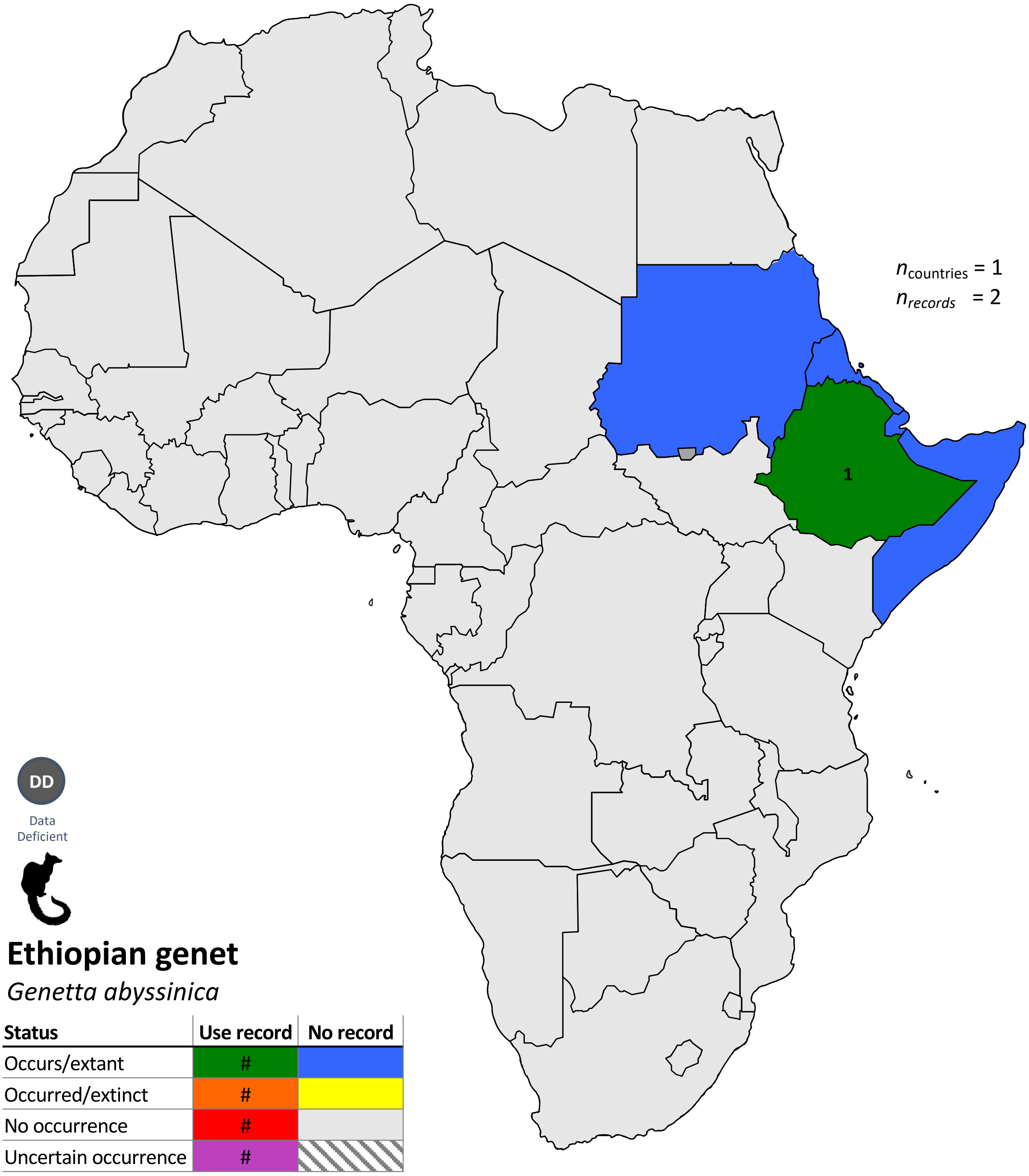

S2.22 Map. Ethiopian genet

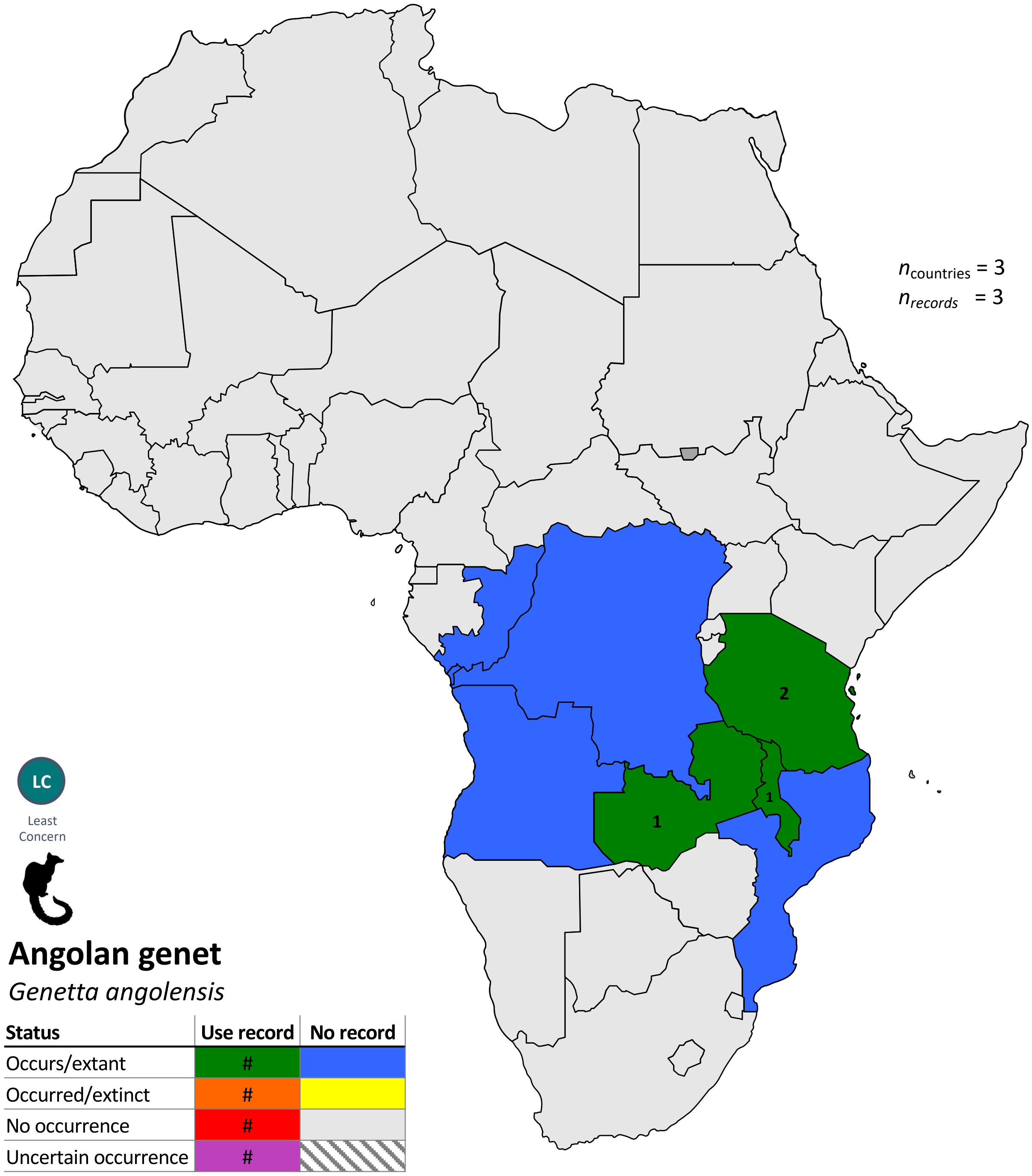

S2.23 Map. Angolan genet

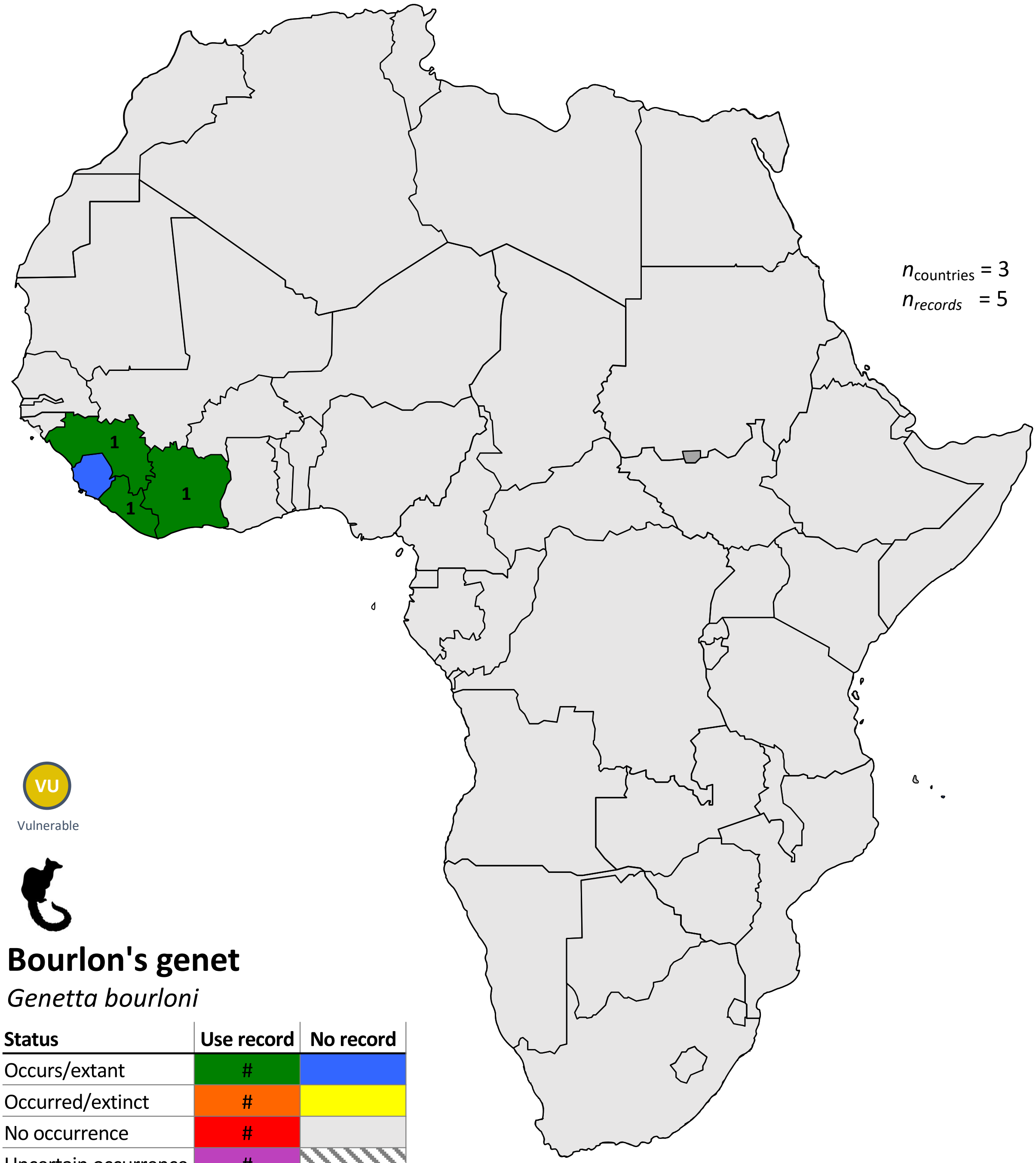

Bourlon's genet

Genetta bourloni

Status	Use record	No record
Occurs/extant	#	
Occurred/extinct	#	
No occurrence	#	
Uncertain occurrence	#	

S2.24 Map. Bourlon's genet

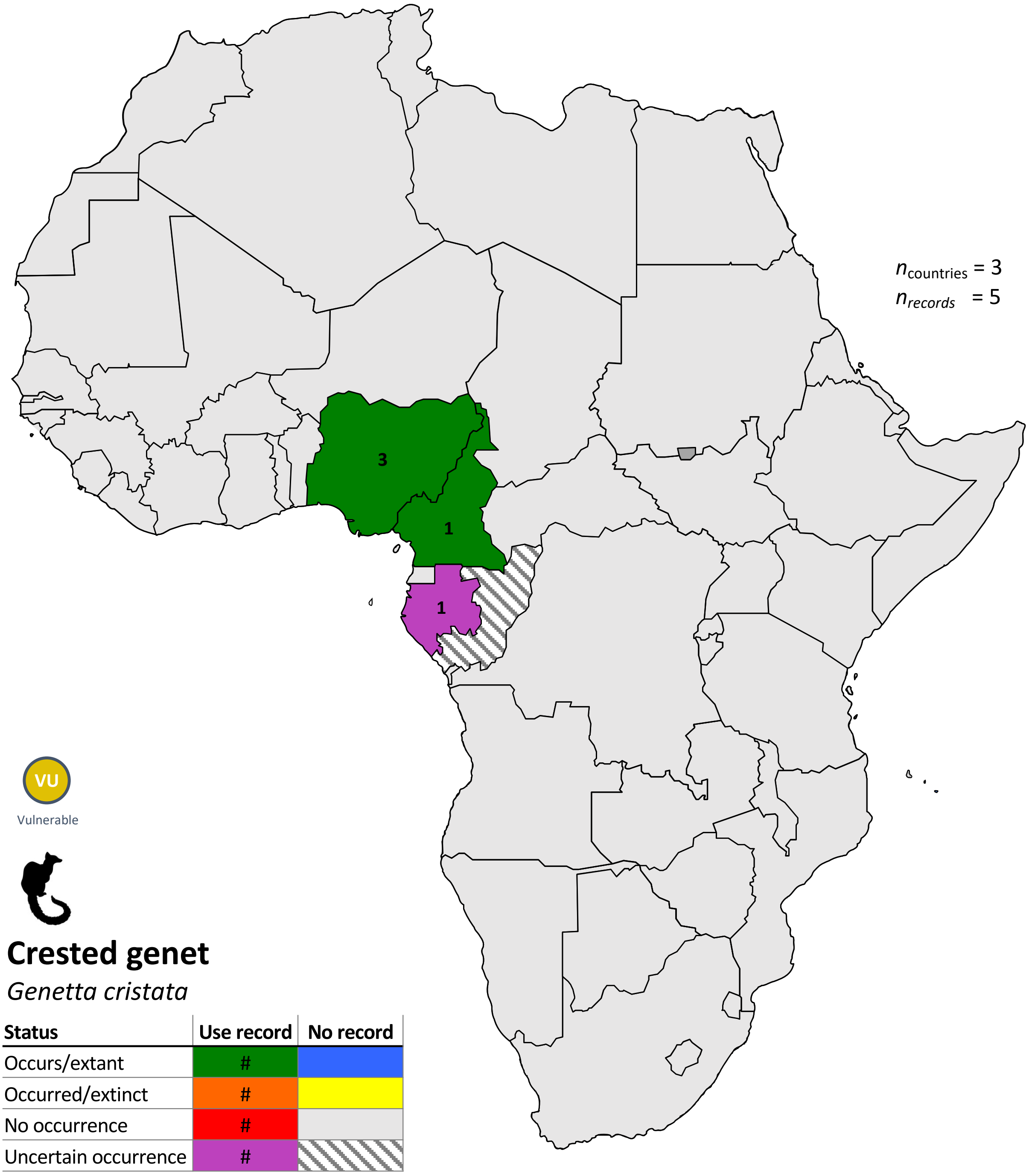

S2.25 Map. Crested genet

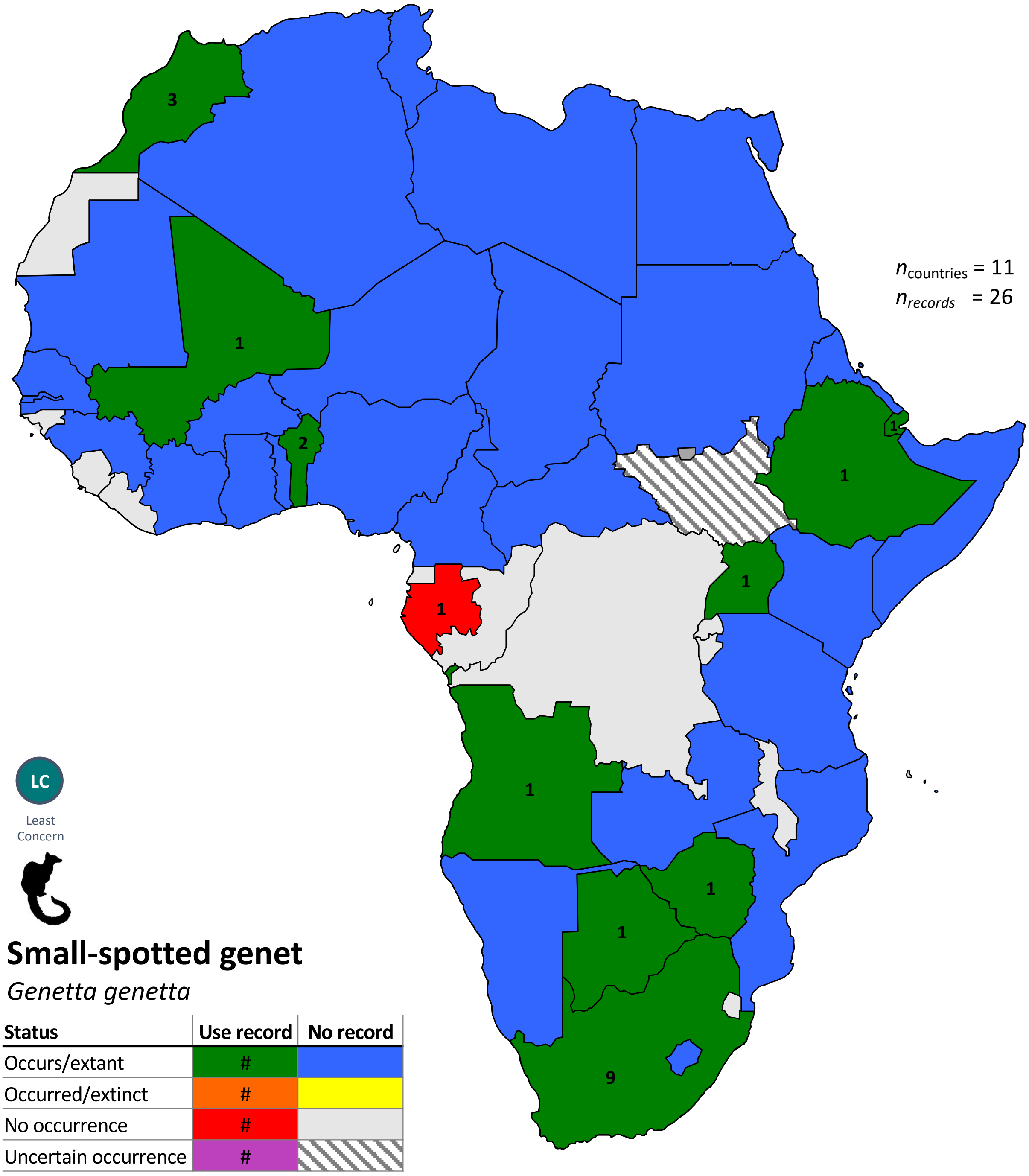

S2.26 Map. Small-spotted genet

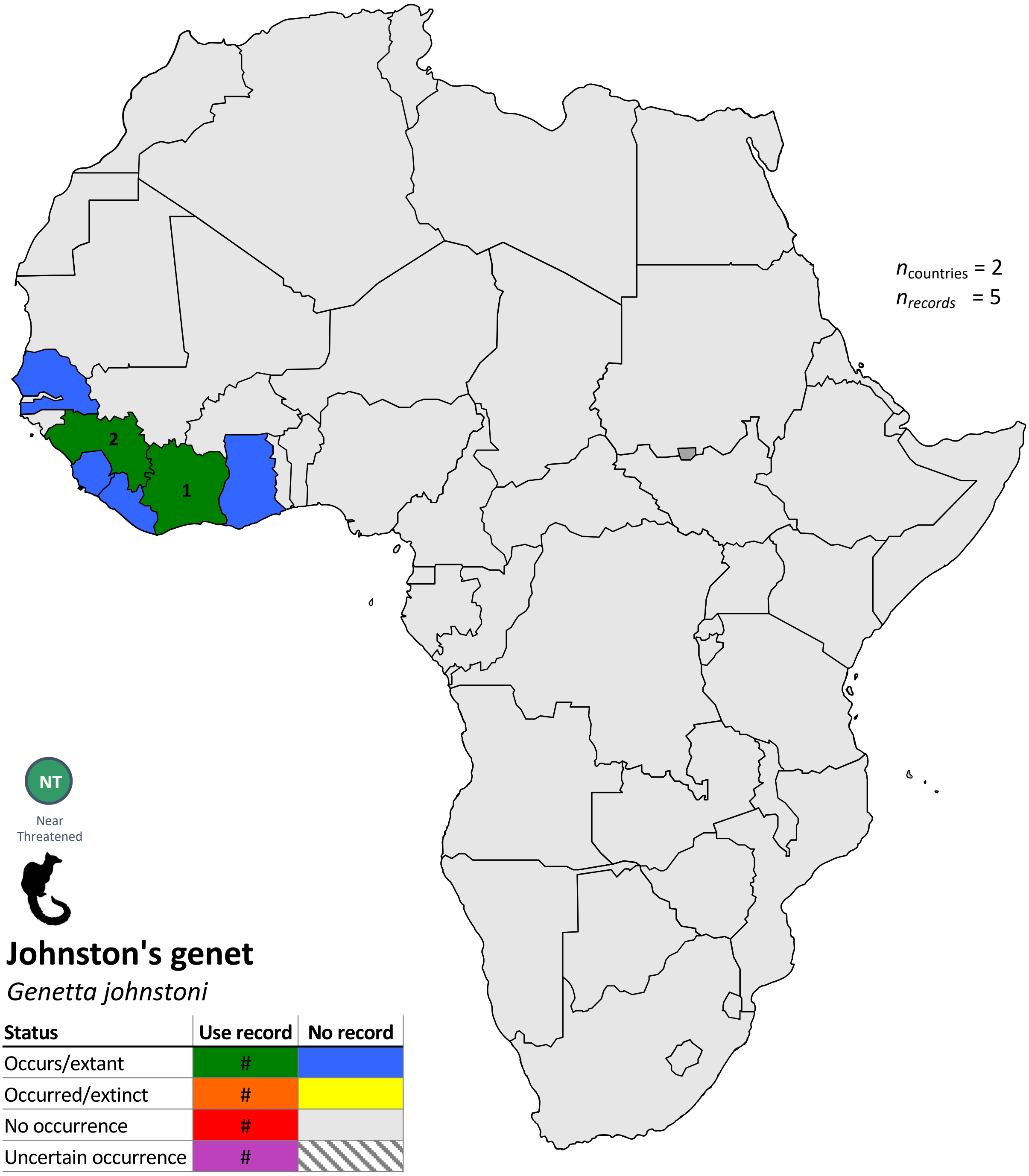

S2.27 Map. Johnston's genet

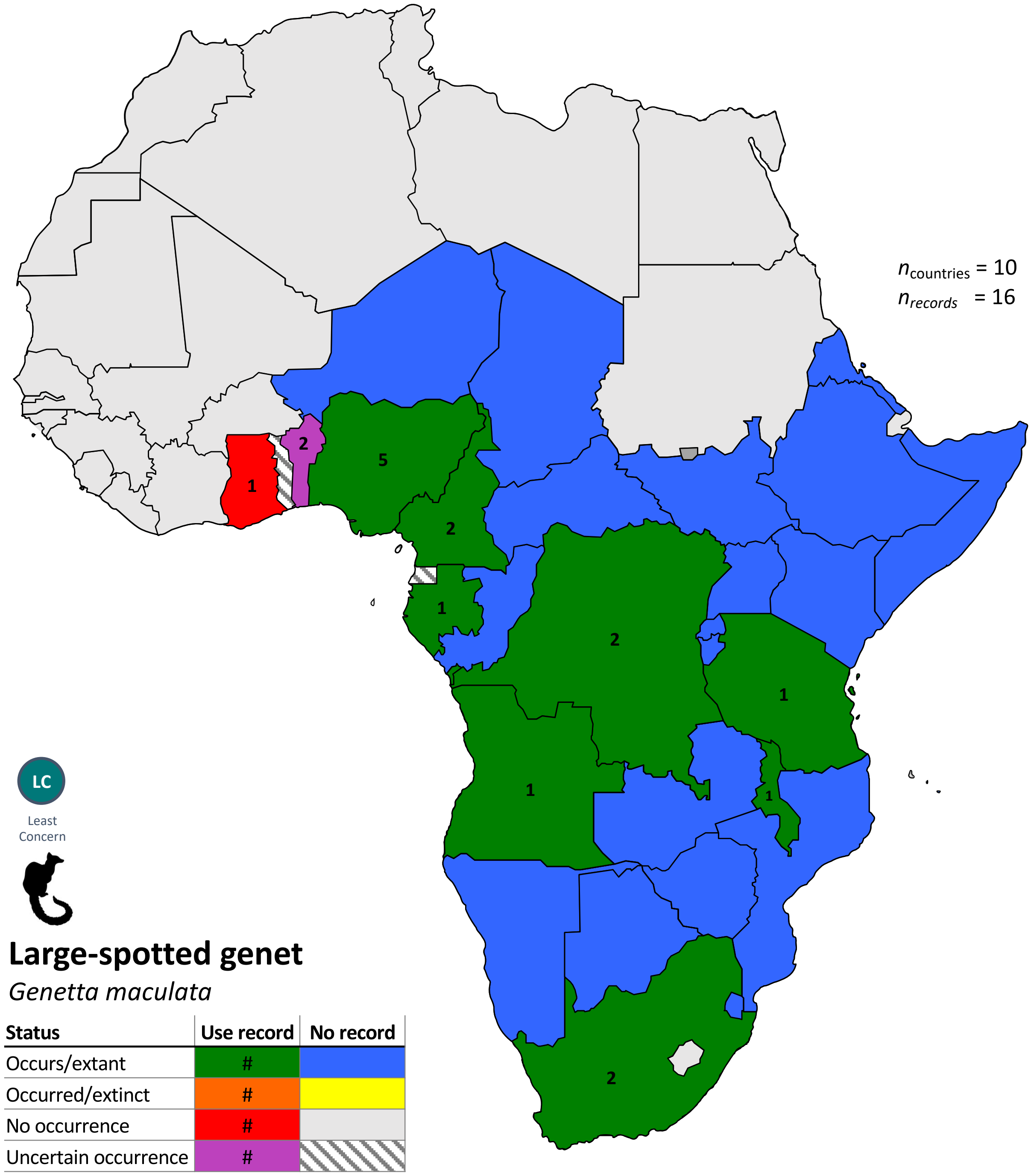

S2.28 Map. Large-spotted genet

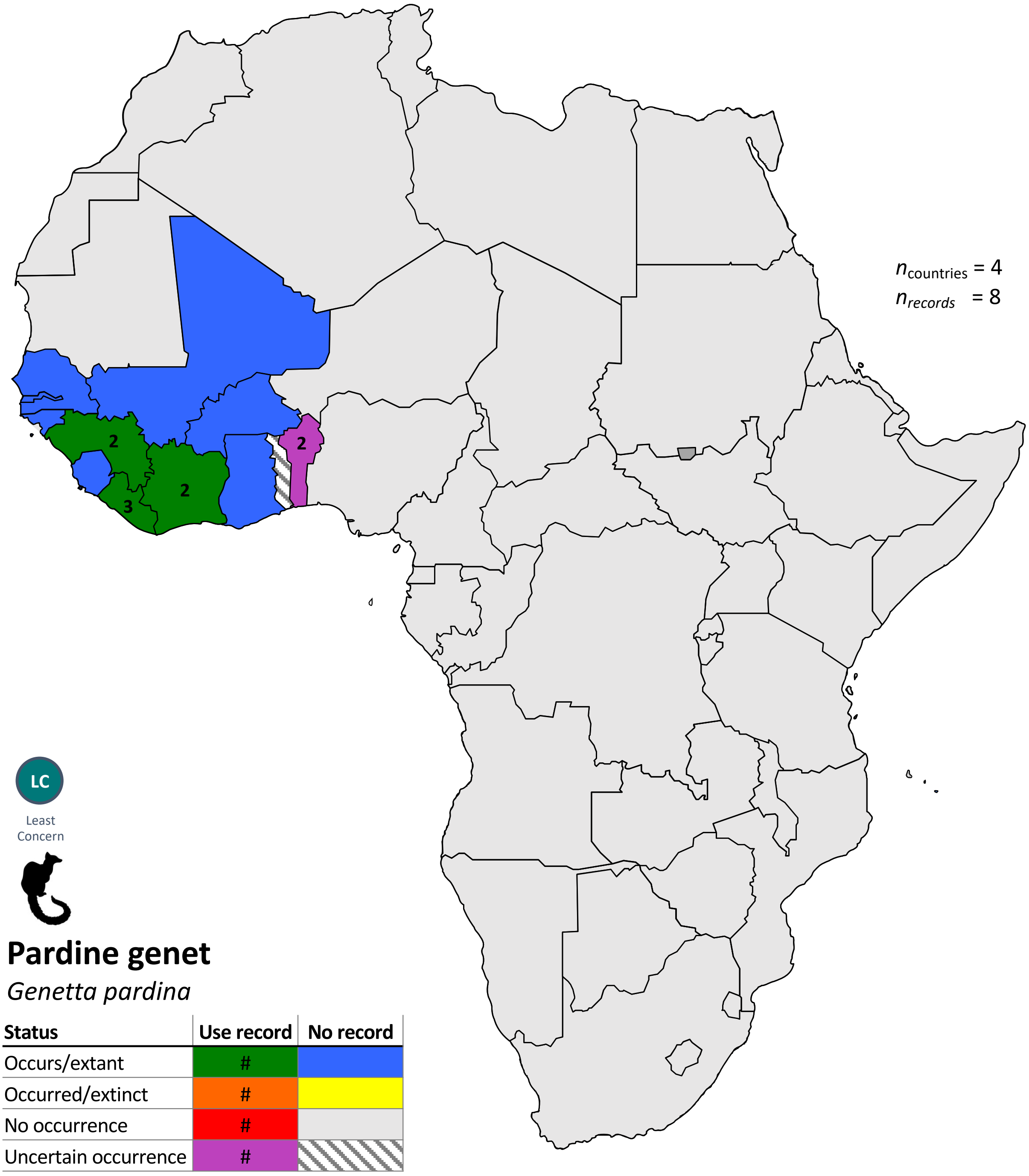

S2.29 Map. Pardine genet

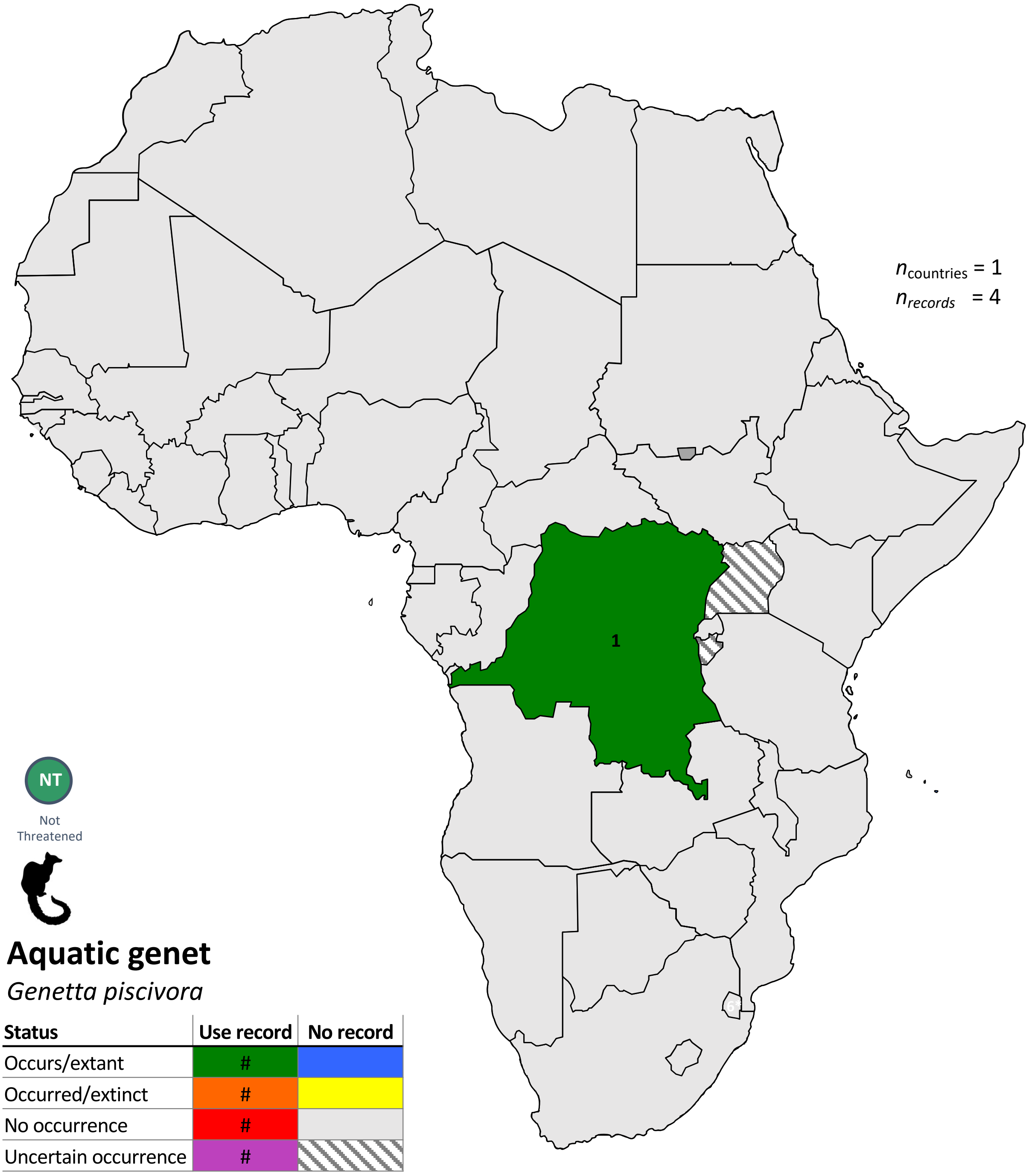

S2.30 Map. Aquatic genet

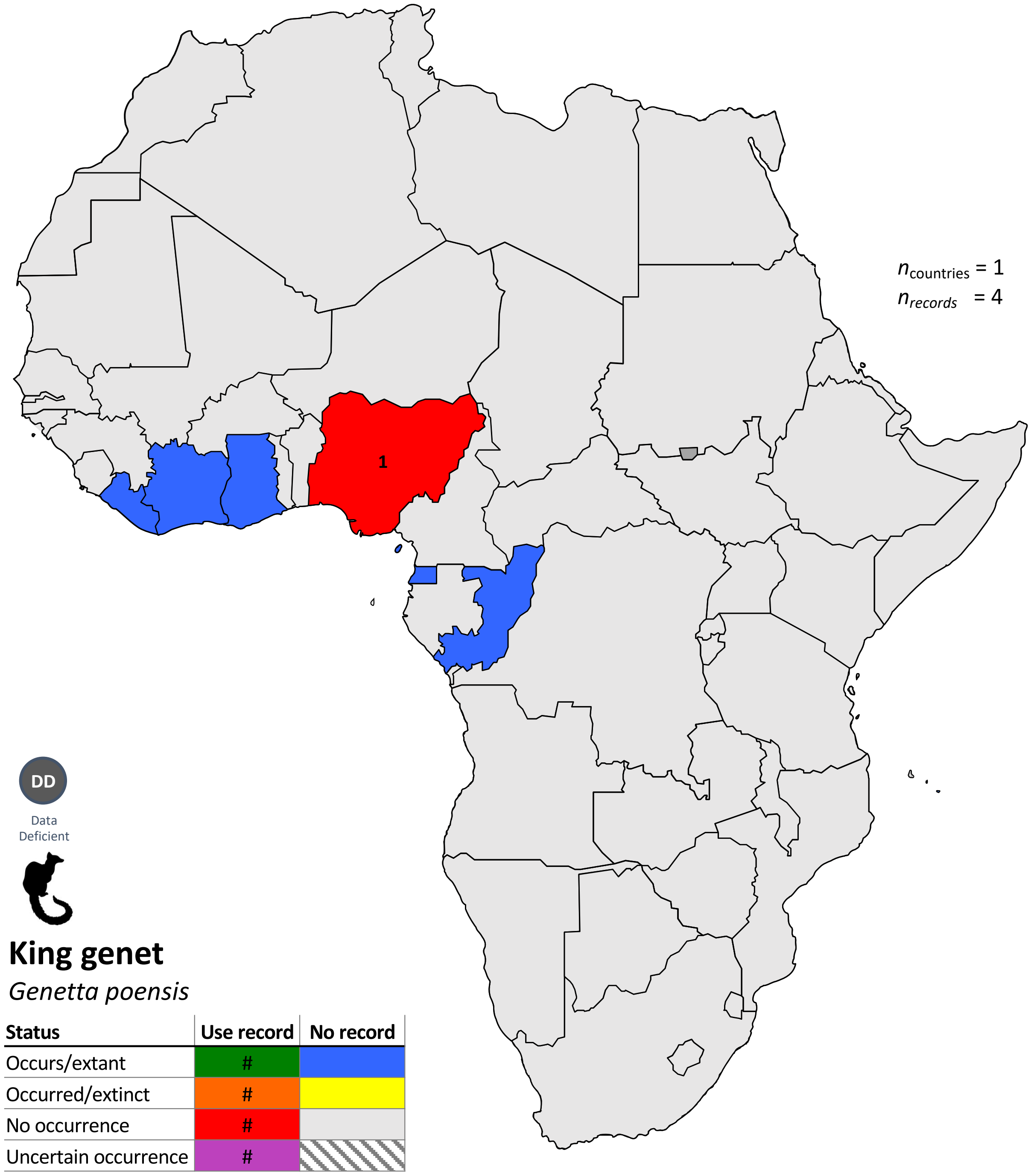

S2.31 Map. King genet

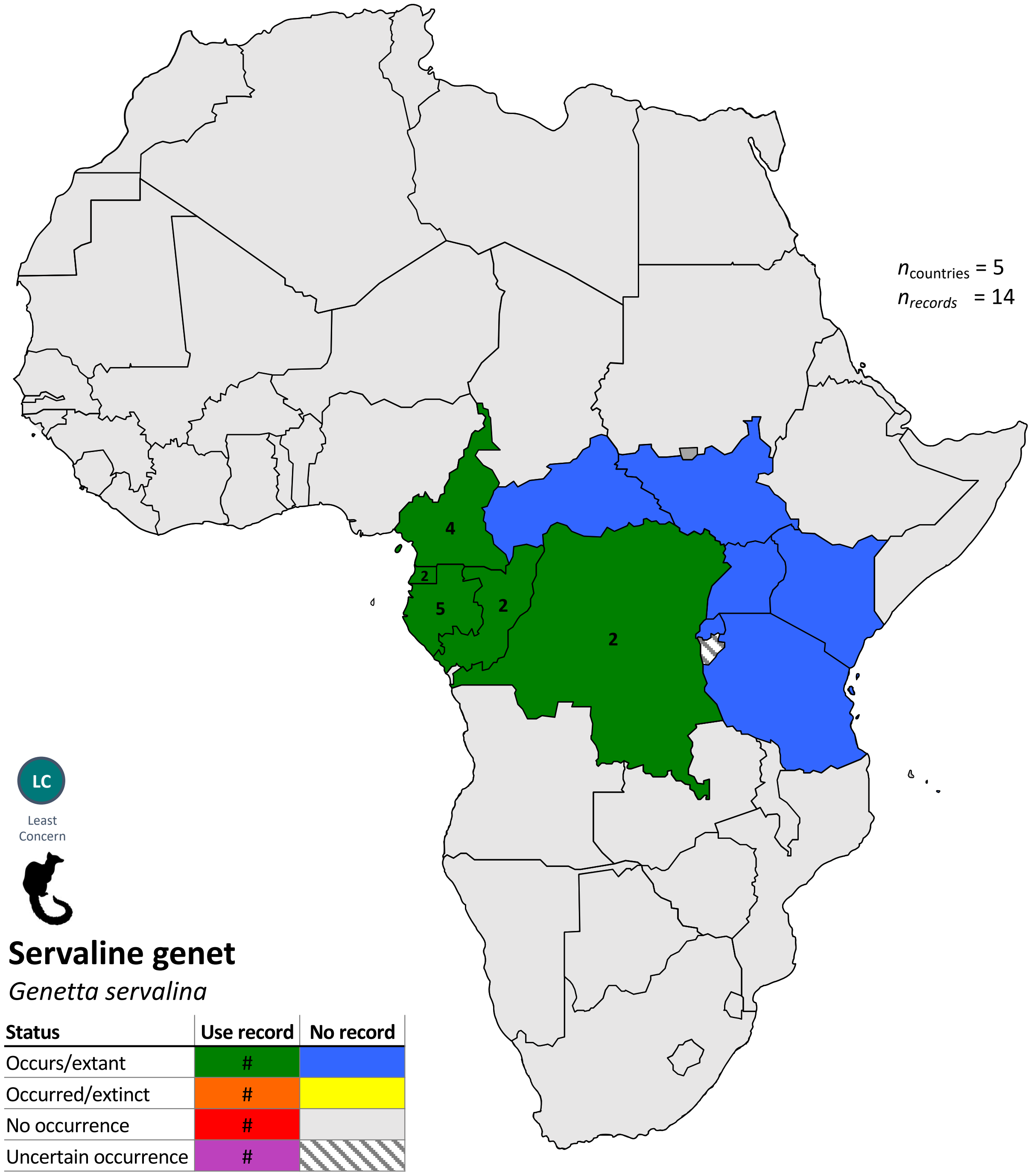

S2.32 Map. Servaline genet

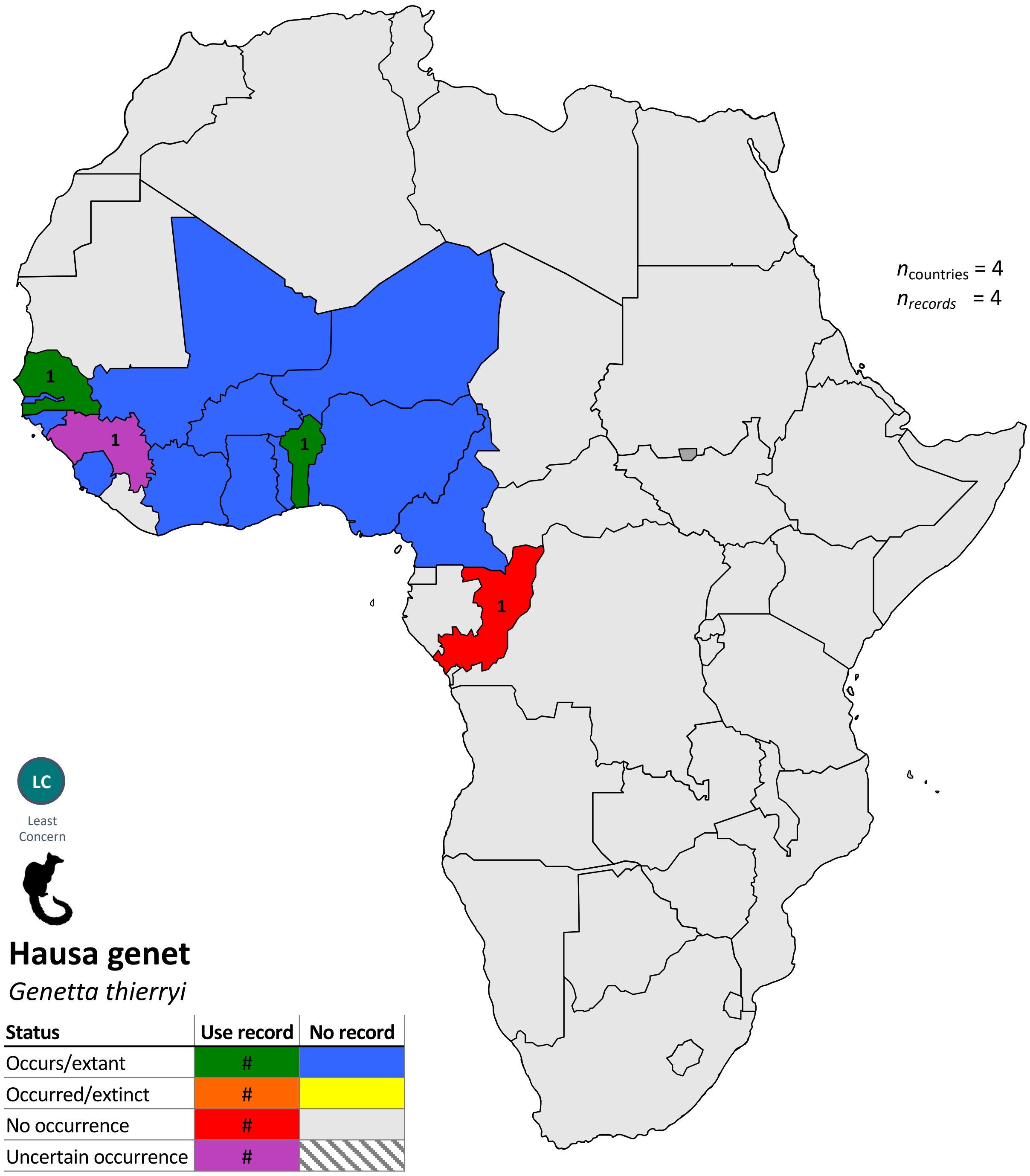

S2.33 Map. Hausa genet

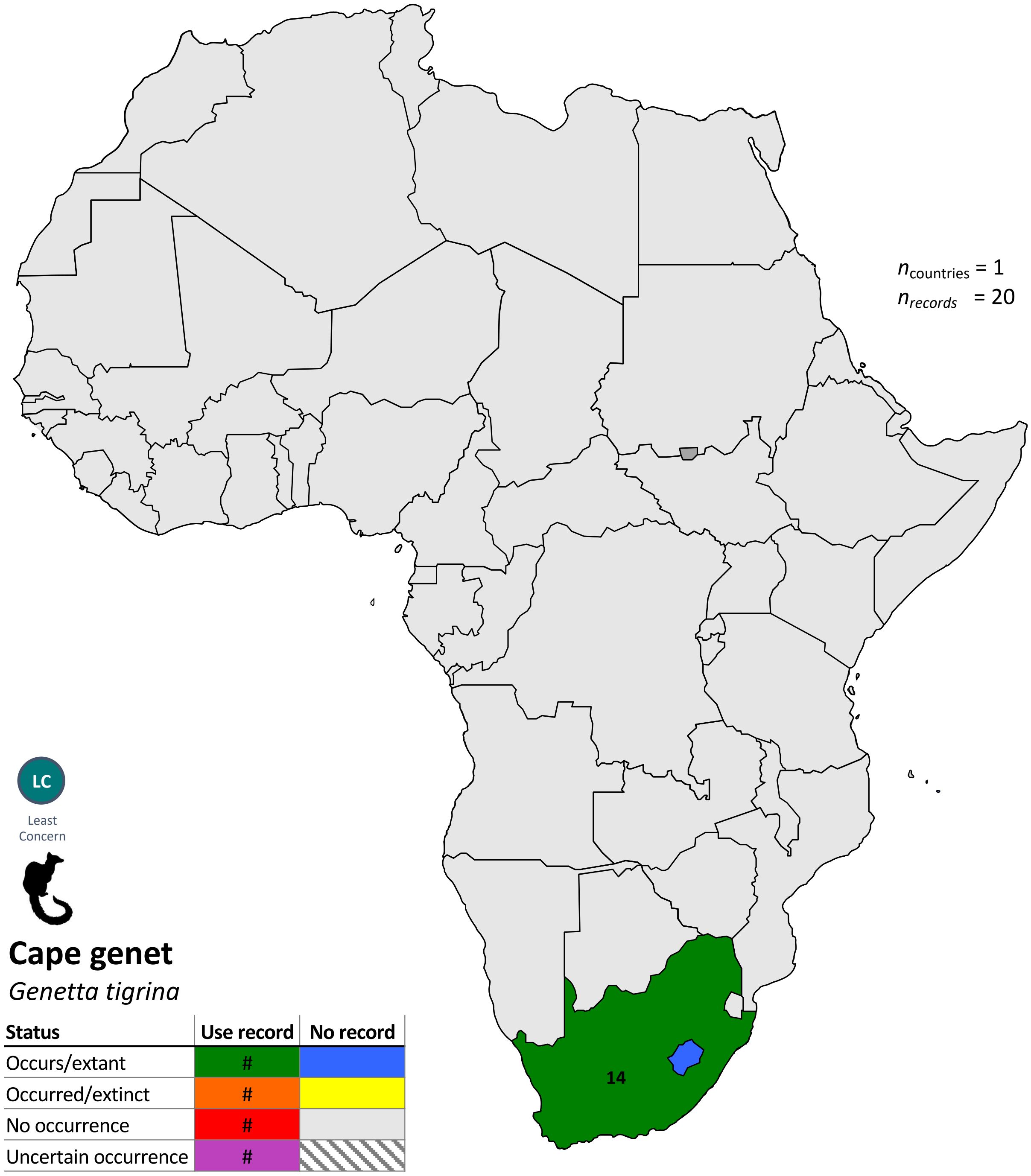

S2.34 Map. Cape genet

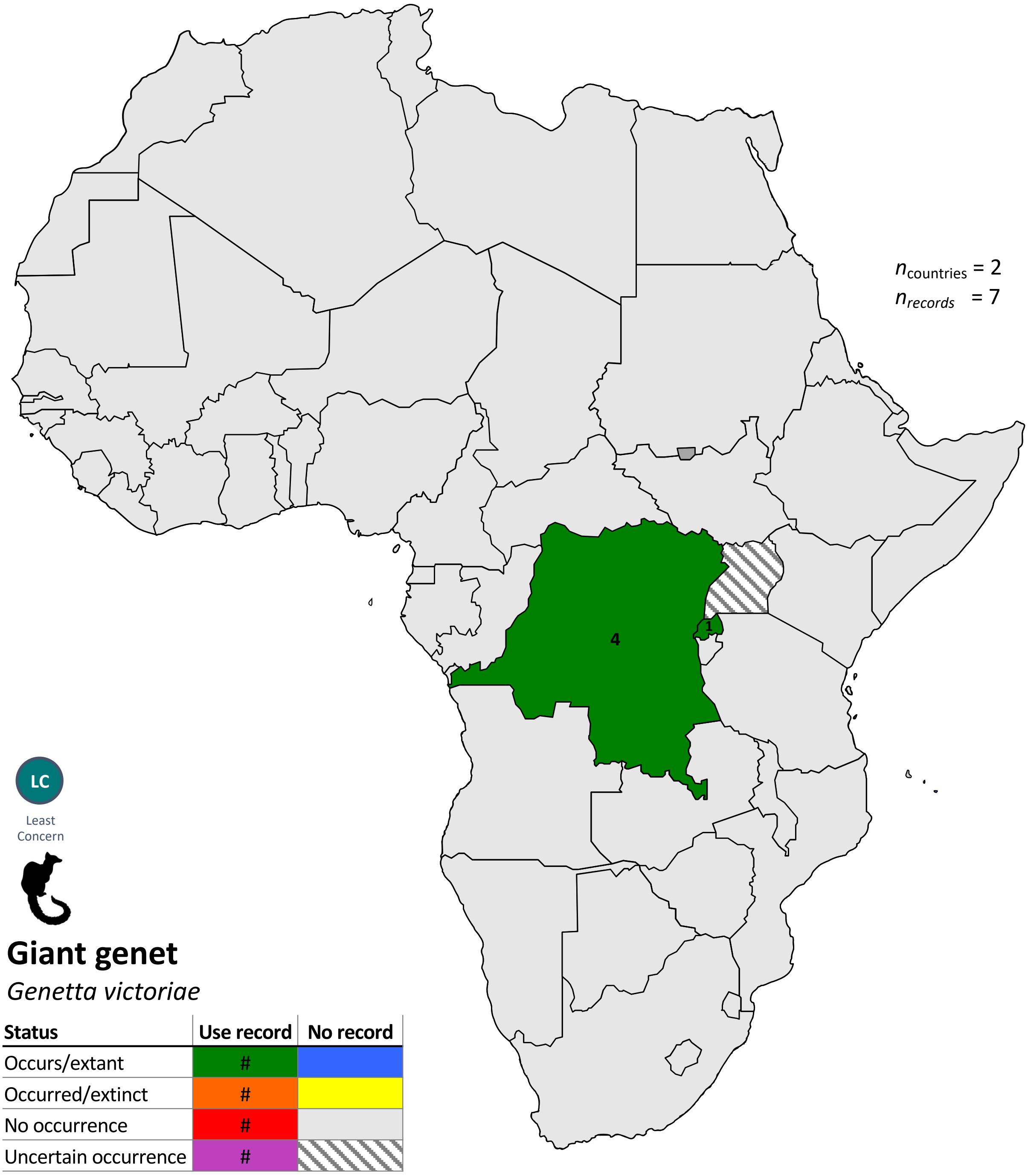

S2.35 Map. Giant genet

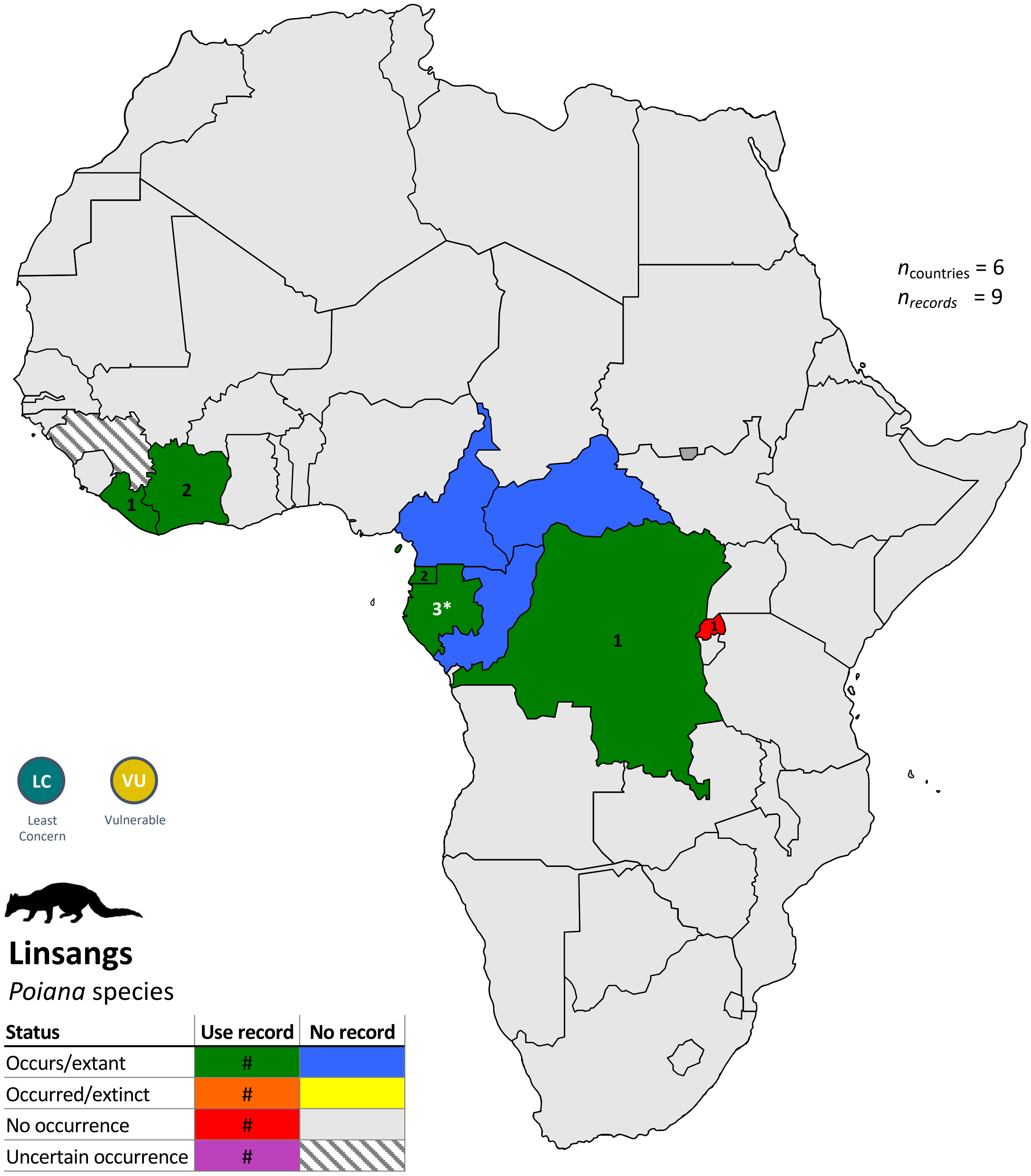

S2.36 Map. Linsangs *Poiana leightoni* & *P. richardsonii*
