# Supplementary material for: Pan-African review of cultural uses of carnivores
Source: PLoS One. 2025 Mar 25;20(3):e0315903. doi: 10.1371/journal.pone.0315903 (PMC11936259; doi:10.1371/journal.pone.0315903)
Supplement: S1–S7 Tables — Supporting information on publications numbers, body mass, YouTube results, and a summary of observations and classifications of focal taxa incorporated into traditional attire. (PDF) [file pone.0315903.s004.pdf]

Pan-African review of cultural uses of carnivores

Vivienne L. Williams, Marine Drouilly, Peter Coals, Gareth Whittington-Jones
(PLOS ONE)

Supporting Information

Tables: S1 to S7

Table of Contents

S1 TABLE. 2
S2 TABLE. 3
S3A TABLE 4
S3B TABLE 5
S3C TABLE 6
S3D TABLE 7
S4A TABLE 8
S4B TABLE 8
S5 TABLE 9
S6 TABLE 10
S7 TABLE 21

References Cited in the Supporting Tables.

ASCaRis (African Small Carnivore Research Initiative): website: https://ascaris.org/.

Kingdon, J. & Hoffmann, M. (eds) *Mammals of Africa: Volume V: Carnivores, Pangolins, Equids and Rhinoceroses*. Bloomsbury Publishing, London. (2013).

Macdonald DW, Loveridge AJ & Nowell K. *Dramatis personae*: an introduction to the wild felids. In: Macdonald DW & Loveridge AJ (eds), *Biology and Conservation of Wild Felids*. Oxford University Press, Oxford. pp 3–58. (2010).

Mkandawire, S.B., Simooya, S.M. & Monde, P.N. *Zambian Culture: Harnessing Cultural Literacy with a Focus on Selected Myths and Taboos*. UNZA Press, Lusaka (2019)

Nowell K & Jackson P. *Wild Cats: Status Survey and Conservation Action Plan*. IUCN, Gland, Switzerland. (1996).

Shange, N. (2013) Shembe Religion's Integration of African Traditional Religion and Christianity: a Sociological Case Study. MA dissertation, Rhodes University, South Africa

Stuart C & Stuart M. *Stuarts' Field Guide to Mammals of Southern Africa, including Angola, Zambia and Malawi*. 5th ed. Struik, Cape Town. (2015).

Stuart, C., Stuart, T. & De Smet, J. Felis silvestris Wildcat. In: Kingdon J, Hoffmann M (eds), *Mammals of Africa: Volume V: Carnivores, Pangolins, Equids and Rhinoceroses*. Bloomsbury Publishing, London. Pp 206–2010. (2013). L – k

S1 Table. Maximum adult body mass per species

Family	African species	Name	Max. adult body mass (kg) [a]
Felidae	*Panthera leo*	African lion	272.0 [b]
	Panthera pardus	Leopard	91.0
	Acinonyx jubatus	Cheetah	64.0
	Caracal caracal	Caracal	20.0
	Leptailurus serval	Serval	13.5
	Caracal aurata	African golden cat	16.0
	Felis lybica lybica	African wildcat	6.5
	Felis lybica cafra	Southern African wildcat	6.4
	Felis nigripes	Black-footed cat	2.5
Mustelidae	*Mellivora capensis*	Honey badger (ratel)	14.5
	Ictonyx striatus	Striped polecat (Zorilla)	1.5
	Ictonyx libycus	Saharan striped polecat (Libyan striped weasel)	0.6
	Poecilogale albinucha	African striped weasel	0.4
	Mustela nivalis	Least weasel	0.3
	Mustela subpalmata	Egyptian weasel	0.4
Nandiniidae	*Nandina binotata*	African palm civet	3.2
Viverridae	*Civettictis civetta*	African civet	20
	Genetta abyssinica	Ethiopian genet	2.0
	Genetta angolensis	Angolan genet (miombo)	2.0
	Genetta bourloni	Bourlon's genet	2.0
	Genetta cristata	Crested genet	2.5
	Genetta genetta	Small-spotted genet (common)	2.6
	Genetta johnstoni	Johnston's genet	2.6
	Genetta maculata	Common large-spotted genet (rusty-spotted)	3.2
	Genetta pardina	Pardine genet	3.1
	Genetta piscivora	Aquatic genet	1.5
	Genetta poensis	King genet	2.5
	Genetta servalina	Servaline genet	2.3
	Genetta thierryi	Hausa genet	1.5
	Genetta tigrina	South African large-spotted genet (Cape)	3.2
	Genetta victoriae	Giant genet	3.5
	Poiana leightoni	West African linsang (West African oyan)	0.7
	Poiana richardsonii	Central African linsang (Central African oyan)	0.75

[a] The maximum adult body mass for the species reported in the literature (*Source*: ASCaRIs website; Kingdon & Hoffmann, 2013; Nowell & Jackson, 1996; Stuart & Stuart, 2015).
[b] Nowell & Jackson (1996) report on a male shot near Mount Kenya weighing 272 kg. However, Stuart et al. (2013), Stuart & Stuart (2015), and Macdonald et al. (2010) all report a maximum mass of 225 kg.

S2 Table. Maximum adult body mass, and size classification, for the morphospecies/taxa investigated. Data used in Fig 6, and S1 and S2 Figs.

Size [a]	African taxa	Name / morphospecies	Max. [b,c] adult body mass (kg)
Small (<6.5kg)	*Poiana* spp. (*P. leightonii, P. richardsonii*)	African linsangs	0.7 (mean max) [d]
	Poecilogale albinucha; Mustela spp. (2 species)	Weasels	0.7 (mean max)
	Ictonyx spp. (2 species)	Polecats	1.1 (mean max)
	Genetta spp. (14 species)	Genets	2.5 (mean max)
	Nandinia subpalmata	African palm civet	3.2
	Felis spp. *(F. lybica* ssp., *F. nigripes)*	Wildcats	5.5 (mean max)
Medium (6.5–20 kg)	*Leptailurus serval*	Serval	13.5
	Mellivora capensis	Honey badger	14.5
	Caracal aurata	African golden cat	16.0
	Caracal caracal	Caracal	20.0
	Civettictis civetta	African civet	20.0
Large (>20kg)	*Acinonyx jubatus*	Cheetah	64.0
	Panthera pardus	Leopard	91.0
	Panthera leo	African Lion	272.0 [e]

[a] Body size category, as defined in Nowell & Jackson (1996) for felids.
[b] The maximum adult body mass for the species reported in the literature (ASCaRIs website https://ascaris.org/; Kingdon & Hoffmann, 2013; Nowell & Jackson, 1996; Stuart & Stuart, 2015).
[c] See S1 Table for the body mass of individual species of weasels, polecats, genets, wildcats and linsangs.
[d] Mean max = average of the maximum body mass reported in the literature for the grouped species.
[e] Nowell & Jackson report on a male shot near Mount Kenya weighing 272 kg. However, Stuart et al. (2013), Stuart & Stuart (2015), and Macdonald et al. (2010) all report a maximum body mass of 225 kg.

S3A Table: Number of publications (N=588) per species per category of traditional attire

Family	Morphotype	Species	Name	Traditional attire			
				Traditional leaders	Other	Political leaders	Shembe
Felidae	Big cat	*Panthera pardus*	Leopard	88	77	19	14
	Big cat	*Panthera leo*	African lion	29	33	5	1
	Big cat	*Acinonyx jubatus*	Cheetah	10	13	0	1
	Big cat	*Panthera* & *Acinonyx* sp.	'Big cats'	0	0	0	0
	Caracal	*Caracal aurata*	African golden cat	0	6	0	0
	Caracal	*Caracal caracal*	Caracal	0	2	0	0
	Serval	*Leptailurus serval*	Serval	10	27	1	4
	Wildcat	*Felis lybica cafra*	Southern African wildcat	2	3	1	0
	Wildcat	*Felis lybica lybica*	African wildcat	0	0	0	0
	Wildcat	*Felis lybica* subsp.	Wildcat subsp.	0	2	0	0
	Wildcat	*Felis nigripes*	Black-footed cat	0	1	0	0
Mustelidae	Polecat	*Ictonyx striatus*	Striped polecat	0	5	0	1
	Polecat	*Ictonyx lybicus*	Saharan striped polecat	0	0	0	0
	Ratel	*Mellivora capensis*	Honey badger	0	5	0	0
	Weasel	*Poecilogale albinucha*	African striped weasel	0	7	0	0
	Weasel	*Mustela nivalis*	Least weasel	0	1	0	0
Nandin-iidae	Civet	*Nandinia binotata*	African palm civet	2	6	0	0
Viverridae	Civet	*Civettictis civetta*	African civet	1	11	0	1
	Civet	*Civettictis* & *Nandinia* sp.	Civet sp.	1	3	0	0
	Genet	*Genetta* sp.	Genet sp.	1	14	0	2
	Genet	*Genetta abyssinica*	Ethiopian genet	0	1	0	0
	Genet	*Genetta angolensis*	Angolan genet	0	0	0	0
	Genet	*Genetta bourloni*	Bourlon's genet	0	1	0	0
	Genet	*Genetta cristata*	Crested genet	0	1	0	0
	Genet	*Genetta genetta*	Small-spotted genet	0	5	0	1
	Genet	*Genetta johnstoni*	Johnston's genet	0	2	0	0
	Genet	*Genetta maculata*	Common large-spotted genet	0	5	0	0
	Genet	*Genetta pardina*	Pardine genet	0	2	0	0
	Genet	*Genetta piscivora*	Aquatic genet	0	1	0	1
	Genet	*Genetta poensis*	King genet	0	1	0	0
	Genet	*Genetta servalina*	Servaline genet	0	6	0	0
	Genet	*Genetta thierryi*	Hausa genet	0	1	0	0
	Genet	*Genetta tigrina*	South African large-spotted genet	0	5	0	0
	Genet	*Genetta victoriae*	Giant genet	0	5	0	0
	Linsang	*Poiana richardsonii*	Central African linsang	0	2	0	0
			**Total* publications per category**	119	146	23	17

** Some data sources contribute to multiple taxa and use categories; hence, the sum of category values per species may not always equal the total number of data sources.*

S3B Table: Number of publications (N=588) per species per category of non-attire

Family	Morphotype	Species	Name	Non-attire		
				Muthi, Zootherapy	Bushmeat food	Musk
Felidae	Big cat	*Panthera pardus*	Leopard	70	27	0
	Big cat	*Panthera leo*	African lion	102	25	0
	Big cat	*Acinonyx jubatus*	Cheetah	21	3	0
	Big cat	*Panthera* & *Acinonyx* sp.	'Big cats'	2	0	0
	Caracal	*Caracal aurata*	African golden cat	6	11	0
	Caracal	*Caracal caracal*	Caracal	8	8	0
	Serval	*Leptailurus serval*	Serval	26	11	0
	Wildcat	*Felis lybica cafra*	Southern African wildcat	10	3	0
	Wildcat	*Felis lybica lybica*	African wildcat	5	2	0
	Wildcat	*Felis lybica* subsp.	Wildcat subsp.	3	3	0
	Wildcat	*Felis nigripes*	Black-footed cat	0	0	0
Mustelidae	Polecat	*Ictonyx striatus*	Striped polecat	17	3	0
	Polecat	*Ictonyx lybicus*	Saharan striped polecat	1	0	0
	Ratel	*Mellivora capensis*	Honey badger	27	15	0
	Weasel	*Poecilogale albinucha*	African striped weasel	18	2	0
	Weasel	*Mustela nivalis*	Least weasel	1	1	0
Nandin -iidae	Civet	*Nandinia binotata*	African palm civet	10	36	0
Viverridae	Civet	*Civettictis civetta*	African civet	28	53	8
	Civet	*Civettictis* & *Nandinia* sp.	Civet sp.	4	9	0
	Genet	*Genetta* sp.	Genet sp.	13	20	0
	Genet	*Genetta abyssinica*	Ethiopian genet	1	0	0
	Genet	*Genetta angolensis*	Angolan genet	2	1	0
	Genet	*Genetta bourloni*	Bourlon's genet	1	4	0
	Genet	*Genetta cristata*	Crested genet	2	3	0
	Genet	*Genetta genetta*	Small-spotted genet	13	9	0
	Genet	*Genetta johnstoni*	Johnston's genet	1	4	0
	Genet	*Genetta maculata*	Common large-spotted genet	8	14	0
	Genet	*Genetta pardina*	Pardine genet	3	6	0
	Genet	*Genetta piscivora*	Aquatic genet	1	3	0
	Genet	*Genetta poensis*	King genet	1	3	0
	Genet	*Genetta servalina*	Servaline genet	5	11	0
	Genet	*Genetta thierryi*	Hausa genet	0	2	0
	Genet	*Genetta tigrina*	South African large-spotted genet	13	5	0
	Genet	*Genetta victoriae*	Giant genet	2	5	0
	Linsang	*Poiana richardsonii*	Central African linsang	2	6	0
			**Total* publications per category**	175	119	8

** Some data sources contribute to multiple taxa and use categories; hence, the sum of category values per species may not always equal the total number of data sources.*

S3C Table: Number of publications (N=588) per species per category of trade

Family	Morphotype	Species	Name	Trade, markets, shops		
				Market observations	Curios, trinkets, tourism	Skins (not-specified)
Felidae	Big cat	*Panthera pardus*	Leopard	38	15	22
Felidae	Big cat	*Panthera leo*	African lion	27	14	17
Felidae	Big cat	*Acinonyx jubatus*	Cheetah	4	7	12
Felidae	Big cat	*Panthera* & *Acinonyx* sp.	'Big cats'	1	1	1
Felidae	Caracal	*Caracal aurata*	African golden cat	9	2	3
Felidae	Caracal	*Caracal caracal*	Caracal	5	0	4
Felidae	Serval	*Leptailurus serval*	Serval	17	5	7
Felidae	Wildcat	*Felis lybica cafra*	Southern African wildcat	0	1	0
Felidae	Wildcat	*Felis lybica lybica*	African wildcat	6	1	0
Felidae	Wildcat	*Felis lybica* subsp.	Wildcat subsp.	0	0	3
Felidae	Wildcat	*Felis nigripes*	Black-footed cat	0	0	1
Mustelidae	Polecat	*Ictonyx striatus*	Striped polecat	2	1	0
Mustelidae	Polecat	*Ictonyx lybicus*	Saharan striped polecat	0	1	0
Mustelidae	Ratel	*Mellivora capensis*	Honey badger	6	1	0
Mustelidae	Weasel	*Poecilogale albinucha*	African striped weasel	1	1	0
Mustelidae	Weasel	*Mustela nivalis*	Least weasel	3	1	0
**Nandin-iidae**	Civet	*Nandinia binotata*	African palm civet	6	1	1
Viverridae	Civet	*Civettictis civetta*	African civet	10	1	5
Viverridae	Civet	*Civettictis* & *Nandinia* sp.	Civet sp.	1	0	0
Viverridae	Genet	*Genetta* sp.	Genet sp.	3	1	2
Viverridae	Genet	*Genetta abyssinica*	Ethiopian genet	2	0	0
Viverridae	Genet	*Genetta angolensis*	Angolan genet	0	0	0
Viverridae	Genet	*Genetta bourloni*	Bourlon's genet	2	1	1
Viverridae	Genet	*Genetta cristata*	Crested genet	1	1	0
Viverridae	Genet	*Genetta genetta*	Small-spotted genet	6	1	1
Viverridae	Genet	*Genetta johnstoni*	Johnston's genet	1	1	2
Viverridae	Genet	*Genetta maculata*	Common large-spotted genet	5	2	1
Viverridae	Genet	*Genetta pardina*	Pardine genet	3	3	2
Viverridae	Genet	*Genetta piscivora*	Aquatic genet	1	1	0
Viverridae	Genet	*Genetta poensis*	King genet	1	1	0
Viverridae	Genet	*Genetta servalina*	Servaline genet	2	2	0
Viverridae	Genet	*Genetta thierryi*	Hausa genet	1	1	2
Viverridae	Genet	*Genetta tigrina*	South African large-spotted genet	2	1	0
Viverridae	Genet	*Genetta victoriae*	Giant genet	1	2	0
Viverridae	Linsang	*Poiana richardsonii*	Central African linsang	3	0	0
			**Total* publications per category**	60	28	50

** Some data sources contribute to multiple taxa and use categories; hence, the sum of category values per species may not always equal the total number of data sources.*

S3D Table: Number of publications (N=588) per species per category of incident and non-specified cultural use

Family	Morphotype	Species	Name	Incidents (parts removed/possession)		Cultural use (not specified)
				Poaching, hunting, HWC	Arrests, confiscation, smuggling	
Felidae	Big cat	*Panthera pardus*	Leopard	54	77	9
	Big cat	*Panthera leo*	African lion	74	58	10
	Big cat	*Acinonyx jubatus*	Cheetah	11	15	7
	Big cat	*Panthera & Acinonyx* sp.	'Big cats'	1	2	0
	Caracal	*Caracal aurata*	African golden cat	2	3	2
	Caracal	*Caracal caracal*	Caracal	3	0	5
	Serval	*Leptailurus serval*	Serval	6	24	4
	Wildcat	*Felis lybica cafra*	Southern African wildcat	1	2	2
	Wildcat	*Felis lybica lybica*	African wildcat	0	0	2
	Wildcat	*Felis lybica* subsp.	Wildcat subsp.	3	2	0
	Wildcat	*Felis nigripes*	Black-footed cat	0	0	0
Mustelidae	Polecat	*Ictonyx striatus*	Striped polecat	1	0	1
	Polecat	*Ictonyx lybicus*	Saharan striped polecat	0	0	0
	Ratel	*Mellivora capensis*	Honey badger	5	7	4
	Weasel	*Poecilogale albinucha*	African striped weasel	1	0	1
	Weasel	*Mustela nivalis*	Least weasel	0	0	0
Nandin-iidae	Civet	*Nandinia binotata*	African palm civet	2	1	2
Viverridae	Civet	*Civettictis civetta*	African civet	6	7	4
	Civet	*Civettictis & Nandinia* sp.	Civet sp.	10	13	2
	Genet	*Genetta* sp.	Genet sp.	7	5	2
	Genet	*Genetta abyssinica*	Ethiopian genet	1	0	0
	Genet	*Genetta angolensis*	Angolan genet	1	0	0
	Genet	*Genetta bourloni*	Bourlon's genet	1	0	0
	Genet	*Genetta cristata*	Crested genet	1	0	1
	Genet	*Genetta genetta*	Small-spotted genet	2	2	2
	Genet	*Genetta johnstoni*	Johnston's genet	0	0	0
	Genet	*Genetta maculata*	Common large-spotted genet	2	1	0
	Genet	*Genetta pardina*	Pardine genet	2	0	0
	Genet	*Genetta piscivora*	Aquatic genet	1	0	0
	Genet	*Genetta poensis*	King genet	1	0	0
	Genet	*Genetta servalina*	Servaline genet	1	1	0
	Genet	*Genetta thierryi*	Hausa genet	1	0	0
	Genet	*Genetta tigrina*	South African large-spotted genet	2	2	3
	Genet	*Genetta victoriae*	Giant genet	1	0	0
	Linsang	*Poiana richardsonii*	Central African linsang	1	0	0
			**Total* publications per category**	112	123	23

** Some data sources contribute to multiple taxa and use categories; hence, the sum of category values per species may not always equal the total number of data sources.*

**S4A Table:** Number of publications (N=588) per species per publication type						
**Family**	**Species**	**Name / Morphospecies**	**Journals**	**Non-journals**	**Newspapers**	**Total**
**Felidae**	*Panthera pardus*	Leopard	72	123	184	**379**
	Panthera leo	African lion	67	108	84	**259**
	Acinonyx jubatus	Cheetah	16	41	13	**70**
	Caracal aurata	African golden cat	10	16	1	**27**
	Caracal caracal	Caracal	10	17	0	**27**
	Leptailurus serval	Serval	27	51	12	**90**
	Felis lybica sspp. & *F. nigripes*	Wildcats & black-footed cat	23	27	1	**51**
**Mustelidae**	*Ictonyx* spp.	Polecats	8	18	0	**26**
	Mellivora capensis	Honey badger	14	34	4	**52**
	Poecilogale & Mustela spp.	Weasels	5	22	0	**27**
**Viverridae & Nandiniidae**	*Civettictis civetta* & *Nandinia binotata*	African palm civet	54	59	7	**120**
**Viverridae**	*Genetta* spp.	Genets	43	68	4	**115**
	Poiana spp.	African linsangs	3	6	0	**9**
		**Total* publications per category**	138	214	236	**588**

** Some data sources contribute to multiple taxa; hence, the sum of category values may not always equal the total number of data sources.*

**S4B Table:** Number of publications (N=588) per species per publication type						
**Categories**			**Journals**	**Non-journals**	**Newspapers**	**Total**
**Traditional attire**	*Traditional leaders*		23	39	57	**119**
	Other	Tribespersons	27	62	57	**146**
		Political leaders	2	2	19	**23**
		Religious	3	5	9	**17**
**Non-attire**	Traditional medicine, zootherapy, *umuthi*		62	94	19	**175**
	Bushmeat / food		52	63	4	**119**
	Musk		0	6	2	**8**
**Incidents**	Poaching, hunting, human-wildlife conflict		15	47	50	**112**
	Confiscations, arrests		4	59	60	**123**
**Trade, markets, shops**	Market observations		18	30	12	**60**
	Curios, trinkets, tourism		6	17	5	**28**
	Skins (not specified)		16	34	0	**50**
**Cultural use (not specified)**			12	11	0	**23**
		**Total* publications per category**	138	214	236	**588**

** Some data sources contribute to multiple categories; hence, the sum of category values may not always equal the total number of data sources.*

S5 Table: YouTube results.
Confirmed records for taxa worn in traditional attire at cultural events depicted in videos: 8 taxa in 23 countries and 78 events.
? = unconfirmed species presence in the video.

Country *	Leopard	Lion	Cheetah	Golden cat	Caracal	Serval	Wildcats	Honey badger	Polecats	Weasels	Civets	Genets	Linsangs	Total: taxa per country
**Angola**	1													1
**Botswana**	1											1		2
**Burundi**	1					1		?				1		3
**Cameroon**	1					1						1		3
**Chad**						1								1
**Congo**	1										1	1		3
**DRC**	1										1	1		3
**Eswatini**	1	1	1			1	1					1		6
**Ethiopia**	1	1	1			1						1		5
**Gabon**	1											1	1	3
**Ghana**	1	1				1								3
**Kenya**	1	1				1								3
**Lesotho**												1		1
**Malawi**	1	1	1			1					1	1		6
**Mozambique**	1											1		2
**Namibia**	1	1	1			1						1		5
**Nigeria**	1					1					1	1		4
**South Africa**	1	1	1			1	1		?		1	1		7
**South Sudan**	1					1								2
**Tanzania**	1					1					1			3
**Uganda**	1					1								2
**Zambia**	1	1	1			1					1	1		6
**Zimbabwe**	1	1	1			1						1		5
**Total: countries per taxa**	22	10	8	0	0	16	2	?	?	0	8	17	1	

***** Togo: there was YouTube video evidence for leopard, lion, cheetah, caracal, civet, and genet in a Fetish market in Lomé, but their specific use in traditional attire could not be confirmed. Including this record, we observed 9 taxa in 24 countries in the YouTube videos

S6 Table: Observations of focal taxa incorporated into traditional attire during cultural ceremonies, festivals, dances, and events, in 23 African countries, verified through the review of 555 YouTube videos (videos links in S3 Appendix; not all videos yielded useable data). Only events with clear evidence of these taxa are included in this table. An initial list of 20 events (indicated by ⌘ and ✪), where people incorporated carnivore products into their attire, was compiled from keyword record searches in various publications (newspaper, journal, non-journal). These events were investigated further by viewing content recommended by YouTube. The number of events were subsequently increased by viewing related video content by employing YouTube's personalized video-recommendation algorithms, along with additional keyword searches on the platform, through the snowball method. This table provides information on the country, location, time of year, tribe/ethnic group, focal species, and event descriptions. Event types are classified into categories based on their distinct characteristics and timing (column 7), while the total quantities of all focal species skins and parts worn at the event, and evident in the videos, are classified into three categories [low (L), medium (M) and high (H); column 8].

Symbols and classifications in the table

⌘ – marked events were initially identified in the Nexis newspaper article search.

✪$^{1-8}$ – nine of the most popular ceremonies in Zambia in 2019 (as per Mkandawire et al, 2019), ranked 1st to 8th (two ceremonies rank joint 5th).

Definitions for event type classifications: (i) **calendrical** or **seasonal**: important annual cultural events taking place at specific times of the year or season, like planting or harvesting (e.g., Nc'wala festivals of the ethnonymous Ngoni-Nguni people); (ii) **contingent**: events held in response to marking the transition from one phase of life to another, and encompassing rituals performed at birth, puberty, marriage, death, coronations and elections; (iii) **affliction**: rituals aimed at appeasing or warding off supernatural beings or forces believed to have brought illness, bad luck, or physical injuries to people or the community; (iv) **divinatory**: ceremonies performed by traditional healers, spiritual elders, religious leaders, and political authorities to ensure the well-being and fertility of humans, animals, and crops within their territories; (v) **initiation**: events introducing younger individuals or generations to a new phase of life or a particular lifestyle, such as to adulthood, secret societies, or the traditional healing and religious professions (e.g., Niembé initiation dance for women in Gabon); (vi) **regular** or **daily**: performed on a regular basis, sometimes even daily, such as offerings, prayers or tributes to ancestors; (vii) **private** or **secret**: conducted privately by specific members of a community or secret societies (e.g., Nyau dancers of the Gule Wamkulu Secret Society in Malawi). At any of these events, participants may incorporate carnivore skins into traditional attire – depending on the requirements of their traditions of practices. NC = Not Classified event or dance (e.g., non-annual dance festivals where purpose wasn't evident, or named dance forms performed at various ceremonies) (See S7 Table – data summary in relation to focal species).

Classification of the total quantities of all focal species combined that were evident in the videos:

- Low (L) = 10 or fewer persons observed wearing either a full skin or skin fragments of varying sizes, with typically less than 5 people in these videos wearing the focal species.
- Medium (M) = more than 10, and up to 50, persons observed wearing full skins and skin pieces of varying sizes of the focal species, often including persons wearing more than one skin (typically male event participants).
- High (H) = more than 50, and sometimes exceeding 100, participants (generally male) observed wearing full skins and skin pieces of varying sizes, with persons frequently wearing multiple full or half skins.
- **Range (R)** = events necessitating further investigation, and/or where the number of focal species skins involved is variable and could span the range from L to H; there was insufficient video footage to make determinations of quantities at these events.

Name	Country	Place	Event timing	Tribe/ethnic group	Focal taxa	Event type classification	Quantity classification	Brief description
**Installations, and burials, of Kings, chiefs and tribal leaders ⌘**	Africa-wide, but documented here in Botswana, Eswatini, Lesotho, Malawi, Nigeria, South Africa, Uganda, Zambia	Based on Kingdom and Chieftaincy locality	Year round	Depends on Kingdom and Chieftaincy	Leopard, lion, serval, genet, civet	Contingent	Individually per event they tend to be **L,** but they are collectively **H** across the continent	Leader-involved events take place across the continent all the time; the timing and frequency is contingent on specific circumstances necessitating their occurrence. New leopard skins are commonly bestowed upon a new leader following the passing of the previous chief. These ceremonies often feature tribesmen and dignitaries adorned in leopard skin attire. In South Africa, for example, lion skins are incorporated into the regalia of Nguni Kings. In Malawi, mages depict Ngoni kings draped in full lion skins, and other men wearing skirts with genet skins. In Zambia, advisors to Paramount Chiefs are known to wear lion skin. The use of leopard and lion skins to denote status is thus widespread in these cultural contexts. In Nigeria, attendees of a wake for a tribal leader wore civet skins.
**Lithathe lya Miondo ya Mbunda**	Angola	Lumbaba Nguimbo, Moxico	Aug	Mbunda	Leopard	Calendrical	**L**	Annual traditional ceremony. King and Paramount chiefs wore leopard skins, and they are also draped on, and under, the King's chair.
**Traditional dance and cultural festivals**	Botswana	Across the country	Year round	Multiple	Leopard, genet, other spotted	NC event	**R**	There are several annual cultural dance festivals e.g., Parakarungu, Guu Tjilenje, Kuru San Maitisong, Dikgafela. Dance attire may incorporate limited numbers of spotted carnivore skins, such as genets, but it depends on the tribe and dance troupe. Some poets and traditional leaders attending these events will wear leopard skin.
**'Witchcraft' divination ceremonies**	Burundi	Across the country	Year round	Not specified	Leopard, serval, genet, other (possibly honey badger)	Divination	**L**	In several videos, medical practitioners known as 'abafumu' wore serval skins, hats with carnivore fur, and possibly honey badger skin; there's also a genet skin hanging in a room. Also depicted in the videos are persons in the village wearing leopard skins around their necks as they accompany the President.
**Funerals**	Cameroon	Bangoulap	Year round	Nde	Leopard, serval, genet	Contingent	**L**	Funerals of noble men. Some of the bags seem to be made with leopard skin.
**Zheu dance**	Cameroon	Bangou village		Bamileke	Leopard	Contingent	**M**	Traditional dance. Dance performed once by a chief to honour his late father. Substantial numbers of

								people draping full skins of focal species over their shoulder, especially leopard.
**Traditional dance**	Chad	Not specified		Tupuri	Serval, other	NC events	L	Small gathering of Tupuri community performing a traditional dance. A few men have serval and other carnivores' skins on their waist.
**Traditional dance**	Congo	Lékoumou		Not specified	Genet, civet	NC dance	L	Music and traditional dance video. >10male dancers nearly all wearing small, spotted carnivore skin aprons. Women not wearing them.
**Ngoma dance**	DRC	Not specified		Pende	Leopard, genet, civet	NC events	M	Dance celebrating the arrival of a hunter bringing his kill. Most male dancers (>20) wearing one or more spotted carnivore skin aprons. Also, young leopard skin.
**Traditional dance**	DRC	Not specified		Mongo	Leopard, genet, civet	NC events	L	Leopard skin worn around the arms of a few dancers**.**
**Traditional dance**	DRC	Not specified		Pende	Genet	NC events	L	Dancers and singers, with the occasional person wearing genet skin.
**Traditional dance**	DRC	Zongo waterfall		Mongo	Genet, civet	NC events	L	Dance video showing many dancers wearing traditional dress, but only some wearing civet and genet.
**Traditional dance**	DRC	Not specified		Mongo	Genet	NC events	L	A performer wearing a genet skin**.**
**Incwala ceremony: starts with incwala lencane (small incwala) and ends with incwala lenkhulu (big incwala)** ⌘	Eswatini	Mbabane and across Eswatini	Dec	Swati	Leopard, cheetah, serval, genet	Calendrical	H	Annual kingship and first fruit ceremony. The main ritual of Kingship in Eswatini is a month-long event that involves several ceremonies and activities – including, a journey by regiments of tribesmen ('warriors') to the Indian Ocean near Maputo. During this significant event, the King, high-ranked attendees, and hundreds (if not thousands) of others wore leopard and other spotted carnivore skin clothing. Importantly, however, these tribesmen also wear their regalia at other cultural events, such as rituals, weddings, funerals, investiture of traditional leaders etc. A full range of garment types are worn (e.g., skirts, capes, headbands, armbands, etc), and each man seems to wear at least two skins.
**Umhlanga (Reed dance)** ⌘	Eswatini	Royal village	Aug/Sep	Swati	Leopard, lion, wildcat	Calendrical initiation	H	Annual ceremony for thousands of unmarried childless girls/women (none of whom are shown wearing carnivore skins). However, the event is attended by the King, high-ranked men, and dignitaries who don mostly leopard skins, as well as

								other felids, like wildcat tails and lion claw necklaces. The traditional attire worn to this event is also worn during the month-long Incwala rituals. A full range of garment types are worn (e.g., skirts, capes, headbands, armbands, etc).
**Dimi ceremony** ⌘	Ethiopia	Omo Valley	All year	Daasanach	Leopard, cheetah, serval, civet	Initiation	M	Main coming of age ceremony of the Dassenach, and a hallmark of their cultural identity. Some tribesmen wore leopard, cheetah and serval skin coats.
**Irrecha festival** ⌘	Ethiopia	South of Addis Ababa	Oct	Oromo	Lion	Calendrical	L	Annual thanksgiving ceremony. During the event, a few Oromo men were observed wearing lion skin cloaks while riding horses, with the halters possibly decorated with other carnivore skins. Although not many skins were visible, it is likely that ownership of such skins could be larger, as not all tribesmen may have attended the event.
**Bwiti**	Gabon	Moyen-Ogooué, Moughenda village	Apr	Mitsogo, Ungom, and others	Leopard, genet, linsang	Affliction	L	Religious and spiritual practice. Ngangas observed performing spiritual dances and songs around fires in the community. Some leopards, genet and linsang skins observed.
**Niembé**	Gabon			Various	Leopard, genet	Initiation	L	Initiation dance for women.
**Traditional dances (incl. Bodi, Mibambou, Ndjembe)**	Gabon	Ogooué-Lolo	Dry season	Various	Leopard, genet	NC events	R	Traditional dances in the region (including rites of passage).
**Traditional dances (incl. Mbali, Ndjobi, Ngwata)**	Gabon	Haut-Ogooué	Dry season	Various	Genet	NC events	R	Traditional dances in the region.
**Grand Durbar** ⌘	Ghana	Kumasi	Aug	Ashanti	Leopard	NC events	L	Ashanti festival. A mass gathering of tribal royalty and chiefs (a 'durbar'). Leopard skin drums reported. Grand Durbars also take place at the end of annual festivals such as the Odwira, or installations of leaders, were leaders parade afterwards.
**Hogbetsotso festival**	Ghana	Volta	Nov	Anlo	Leopard, lion, serval	Calendrical	L	Various ceremonies held during festival, including peace-making and purification. It ends with a durbar of chiefs and people. Whole lion skin and taxidermized head paraded in front of a chief. Evidence of a few people with bits of leopard and

								serval skin – but insignificant numbers compared to the number of participants.
**Lion hunting event**	Kenya	Not specified	All year	Masai	Lion	Calendrical	**L**	Gathering of lion hunters, allowing Masai warriors to demonstrate their fighting ability. A few wearing lion skins and mane garments.
**Traditional dance: performed at wedding**	Kenya	Not specified	All year	Pokot	Leopard	Contingent	**L**	Traditional dance performed at ceremonies – in this case a wedding. A few men wore leopard skin capes. No women wearing them.
**Traditional dance festivals**	Kenya	Across the country	All year	Various	Serval	NC events	**R**	Traditional dances performed by various clans (Banyabasi, Bugumbe, Bukira). Many serval skins observed.
**Traditional dance**	Lesotho		All year	Basotho	Genet	NC dances	**L**	Traditional dance performed by Basotho women wearing genet skin hats.
**Centenary celebration**	Malawi	Hora Mountain, Mzimba		Ngoni	Leopard, serval, genet, civet	NC event	**H**	The centenary celebrations of the Kingdom in the district. Leopard skins worn by traditional leaders. Also, serval and civet skins, and hundreds of genet skins. One Heritage Fur from Panthera's Furs for Life initiative.
**Gule Wamkulu dance**	Malawi, Zambia, Mozambique		Aug	Chewa	Serval	Private/secret	**L**	The 'Big Dance' of the Chewa. Head bands made from serval skin observed. Mainly performed at funerals, memorial services, initiations and other celebrations. On UNESCO's list of Intangible Cultural Heritage of Humanity.[1] Video showed the dance being performed in Malawi.
**Ingoma dance**	Malawi	Mchinji	Jul	Ngoni	Serval, genet	NC dance	**M**	Traditional dance performed by boys and girls in traditional ceremonies, such as coming of age, weddings, etc. A purpose is to instill solidarity among the youth. Tens of genet skin skirts and hats, or full genet skins on their backs observed. Serval skins also evident.
**Maseko Ngoni Lilongwe Chapter – cultural festival**	Malawi	Lilongwe	May	Maseko-Ngoni	Leopard, serval, genet	NC event	**M**	The cultural festival hosted by the Lilongwe Chapter of the Maseko-Ngoni was aimed at teaching important cultural values to children. At least 87 children, including 41 boys, attended the

[1] Gule Wamkulu dance: On UNESCO's List of Intangible Cultural Heritage https://ich.unesco.org/en/RL/gule-wamkulu-00142, which is a list of cultural practices and traditions aimed at recognising, preserving, and promoting the cultural heritage of different communities. (https://ich.unesco.org/en/lists)

								event in 2017, but it is unclear if this is a recurring event.
**Nc'wala**	Malawi	Mtenguluni Village, Chipata	Feb	Ngoni	Leopard, serval, genet	Calendrical	**H**	Annual cultural festival. Ngoni people gather to pay homage to their Chief, and God, for the gift of food. Hundreds of genet skin hats, some serval skins in hats and skirts, a few leopard skins worn by traditional leaders.
**Umhlangano wa Maseko Ngoni**	Malawi	Nkorimbo, Ntcheu	Nov	Maseko-Ngoni	Leopard, serval, genet	Calendrical	**H**	Annual celebration of culture. Dozens/hundreds of people wearing skins of different species (men and woman) were seen. A full range of garment types were evident (skirts, capes, headbands, armbands, etc).
**Umthetho cultural festival**	Malawi	Hora mountains, Mzimba	Aug	Mzimba-Ngoni	Leopard, lion, serval, genet, civet	Calendrical	**H**	Annual cultural festival. Leaders and hundreds of dancers wearing traditional dress celebrating their heritage through dance, dressing, music, language. Thousands of skins evident include leopard, lion, serval, genet and civet. A full range of garment types worn by men and women were on display (skirts, capes, headbands, armbands, etc)
**Zakumudzi umhlangano ceremony**	Malawi	Ntcheu district	Sep	Ngoni	Leopard, serval, genet, civet	Calendrical	**H**	Annual cultural festival that seeks to restore the unity, cultural heritage and unique identity of the Ngoni. Hundreds of genet skins observed, with some leopard, serval and civet.
**Batsara Batsapi festival**	Namibia	Sangwali	Aug	Mayeyi	Leopard, genet	Calendrical	**L**	Annual Mayeyi cultural festival; very little evidence of skins, apart from possibly one traditional leader in one video. Genet skin headdress, and bits of leopard skin in skirts, in another video.
**Lusata cultural festival** ⌘	Namibia	Chinchimane, Zambezi	Sep	Mafwe	Leopard, lion, cheetah, serval	Calendrical	**M**	Annual cultural festival celebrating the heritage of the Mafwe people. It includes participants from Zambia and Botswana. The chief, and a few other leaders, wore leopard skin. The chief's principal councilors (ngambela) usually wear lion skin. Lozi Kuomboka dancers also attend, and a few wore felid skins. These skins tend not to be draped and not cut and sewn into a design.
**Olufuko cultural festival**	Namibia	Outapi, Omusati	Sep	Aawambo	Leopard, genet	Calendrical initiation	**L**	Annual womanhood festival; a few traditional leaders with leopard and genet skin garments.
**Tulikonge** ⌘	Namibia	Choi	Aug	Mafwe	Leopard	Calendrical	**L**	Annual festival for the Mafwe, including people from Zambia. A few leaders, such as a chief, wore leopard garments like sashes and hats.

**Zambezi cultural festival**	Namibia	Chincimane (Zambezi region)	Jul	Mpezani-Ngoni	Leopard, serval	NC event	**L**	A cultural festival. Dances performed with people wearing serval skins. One leopard skin evident on the ground.
**Cultural festivals**	Nigeria	Plateau State	All year	Multiple	Genet, civet, linsang, other	NC events	**R**	From random selections of seven festivals to view on YouTube, not many people appear to be wearing carnivore skins – but it's hard to see and requires further investigation. There are >250 cultural groups in the country, and >70 annual festivals e.g., Nzem Berom.
**Tarok Concert**	Nigeria	Plateau State	Mar/Apr	Tarok	Leopard, genet	NC events	**L**	Concert featuring cultural dance.
**African Congregational Church events**	South Africa	Event specific	All year		Leopard	NC religious events	**M**	It appears from images that Church elders incorporate leopard skin into their robes.
**Umthayi Marula festival** ⌘	South Africa	KwaNgwanase	Feb	Tembe/Thonga	Leopard, lion, serval, genet	Calendrical	**M**, but collectively probably **H**	Annual celebration of the first harvest of the marula fruit, with many people wearing traditional attire. The usual spotted carnivore skins worn – the videos show different species. People attending this event would also attend the Shaka Day festival, and others on the Zulu-Nguni cultural calendar – hence the traditional attire is not necessarily specifically sourced for this event. Several of these festivals are held in different villages under different traditional leaders.
**Umgidi**	South Africa	Event specific	All year	Xhosa, Zulu, Shembe	Leopard, lion, serval, genet	NC dance	**H**	An umgidi is a celebratory dance in Xhosa and Zulu cultures. In Xhosa culture, the umgidi is a celebratory post-circumcision party to welcome home new initiates. In the Shembe Church, the umgidi is a sacred dance that borrows from the Zulu traditional dance style, and links the living to the dead (Shange, 2013). The purpose of the celebration, and the type of participant, will determine the extent of carnivore skin use – but can involve hundreds of Shembe men in capes, skirts, armbands and headbands.

**Umkhosi woKugubha ilembe (Shaka day)** ⌘	South Africa	South Africa	Sep	Zulu	Leopard, lion, serval, genet, maybe polecat	Calendrical	H	Annual celebration of King Shaka. Kings, traditional leaders and dignitaries don leopard skins. Additionally, the Kings wear lion skins and claws. Hundreds of men, primarily Zulu but invited guests from other tribes, participate. A full range of garment types (skirts, capes, headbands, armbands, etc).
**Umkhosi woMhlanga (Reed dance)** ⌘	South Africa	Nongoma	Sep	Zulu	Leopard, lion, serval, genet, wildcats	Calendrical initiation	H	Annual ceremony for thousands of unmarried childless girls. The event is attended by the Zulu King, high-ranked men, and dignitaries. They wore mostly leopard skins (and other felids, including wildcat tails and lion claw necklaces). Hundreds of men, primarily Zulu but invited guests from other tribes likely. A full range of garment types (skirts, capes, headbands, armbands, etc).
**Traditional dance**	South Sudan	Pibor county		Murle	Serval	NC event	L	Several videos showing men wearing mainly fabric spotted carnivore prints, with <10 wearing serval skin.
**Traditional dance**	South Sudan	Torit county		Toposa	Leopard	NC event	M	>25 male dancers with relatively new full leopard skin capes. >100 female dancers not wearing any carnivore skins.
**Serengeti cultural festival**	Tanzania	Mugumu	July	Multiple	Leopard, serval, civet	NC event	L	A few individuals in dance troupes wore spotted carnivore skin around their waist (possibly civet).
**Acholi cultural festival** ⌘	Uganda	Acholi sub-region	Dec	Acholi	Leopard, serval	Calendrical	M	Annual cultural festival celebrating Acholi culture, and showcasing dance, customs, and ceremonies. In one year, there were >205 Acholi traditional dance troupes, wearing traditional dress, many (but not all) wearing leopard and other carnivore skins. It depends on the troupe as to whether they wear carnivore skins – but the numbers are low (>10 in video link).
**Buganda ceremony**	Uganda	Buganda Kingdom		Buganda	Leopard, serval	NC event	L	A ceremony featuring the Buganda King, who is wearing a leopard skin across his chest. An old man preceding him is wearing serval skin.
**Gulu cultural festival**	Uganda	Gulu Municipality		Acholi	Leopard	Calendrical	L	Annual cultural festival celebrating the cultural heritage of the Acholi people. A traditional leader, and some other men, are seen wearing leopard skin.

**Bene Mukuni traditional ceremony** ⌘	Zambia	Kazungula District	Jul & Dec	Toka Leya	Leopard, lion, other	Calendrical	L	Annual event to commemorate tribe's journey from Congo to Zambia; attended by tribal leaders from DRC and South Africa; a few people wearing spotted skins – likely elders. Elder wearing lion mane headdress.
**Chakwela Makumbi**	Zambia	Chongwe District	Sep	Soli	Serval, genet	Calendrical	L	Annual event meaning 'bringing down the clouds', to mark the beginning of new planting season; occasional person in a dance troupe wearing skins e.g., serval and genet-skin hats.
**Chisemwa Cha Lunda**	Zambia	Mwinilunga District	Sep	Lunda	Leopard, other	Calendrical	L	Annual traditional ceremony to preserve culture and traditions of the Lunda. Several traditional leaders with leopard and other skins.
**Ingoma dance**	Zambia			Ngoni	Serval, genet	NC dance		A predominantly male dance performed by serval ethnic groups in Zambia, primarily the Ngoni, at cultural events. Hundreds of genet skin skirts, and some serval skin in headbands and elsewhere.
**Kazanga**	Zambia	Kaoma district	Jul	Nkoya	Leopard, lion	Calendrical	M	Annual harvest festival. Leopard and lion skins evident in the background.
**Kufikwila**	Zambia	Solwezi District	May	Kaonde	Serval, genet, other	NC event	L	Chief wearing felid skin; sporadic evidence of use; limited to a few leaders and dancers.
**Kulamba Kubwalo 1**	Zambia	Katete District	Aug	Chewa	Serval, genet	Calendrical	L	Annual event where subordinate chiefs pay respect to paramount chiefs; clans from Mozambique and Malawi present; occasional dancer wearing genet skins; serval evident.
**Kulamba Kubwalo 2** ✪[8]	Zambia	Chibombo District	Oct	Lenje	Leopard, serval, other	Calendrical	L	Annual harvest festival. Participants wore traditional regalia, a Chief wore leopard, and a few attendees had serval skins draped over shoulders and headbands. Probably other felids as well.
**Kuomboka festival** ⌘✪[1]	Zambia	Barotse floodplain, near Mongu	Apr; Aug/Sep	Lozi	Leopard, lion, serval, other	Calendrical	H	Annual festival of Lozi people[2]; leopard, serval and lion skins worn. Part of Panthera's Heritage Furs project. This is the main Kuomboka festival; there are two smaller festivals held under Chieftainesses in different districts. Panthera has conducted research on this event.

[2] Info from M. Lishandu and J. Dunnik (*pers. comm.*, 2021): Main Kuomboka events from the wet palaces to the dry palaces take place in Mar/Apr depending on the water levels. The Kufuluhela event (basically a reverse Kuomboka from the dry palace to the wet palace) takes place in July/August depending on the receding water levels. The same attire is worn for both the Kuomboka and respective Kufuluhela and involves the same number of paddlers. There may also be an overlap in the paddlers chosen for the respective Kuomboka and Kufuluhela events. There are also three separate Kuomboka events (and corresponding Kufuluhela - some variation in attire). One for the Litunga from Leaului to Limulunga, one for the Queen of the South from the Nalolo wet palace to the Mooyo dry palace and one for the Queen of the North from the Libonda wet palace to the Mulundumano dry palace.

**Kuomboka Libonda**	Zambia	Kalabo District	May	Lozi	Leopard, serval	Calendrical	H	A smaller annual Kuomboka festival in Kalabo under Chieftainess Mboanjikana. Panthera has conducted research on this event.
**Kuomboka Nalolo**	Zambia	Senanga District	May	Lozi	Leopard, serval	Calendrical	H	A smaller annual Kuomboka festival in Senanga under Chieftainess Litunga La Mboela. Panthera has conducted research on this event.
**Lwiindi Gonde** ✪[5]	Zambia	Monze District	Jul	Tonga	Leopard, other	Calendrical	L	Annual thanksgiving festival to appease gods and bring good rain and harvest; variable use evident in 2011 videos. The chief and several dancers wearing skins. In 2017 videos, the occasional person is seen with leopard skins draped over shoulders.
**Makishi dances**	Zambia	Northwesstern and western provinces	All year (especially Aug)	Luvale, Chokwe, Luchazi, Mbunda people	Genet	Private/secret	L	A makishi dance is a masquerade dance, representing spirits of deceased ancestors, performed at important cultural festivals, ceremonies, including the Mukanda initiation ritual, and the Likumbi Lya Mize, of the Luvale. Their costumes may include wildlife. Some attendees wore genet skins on their heads. The Makishi Masquerade is on UNESCO's list of Intangible Cultural Heritage of Humanity.[3]
**Mutomboko** ✪[4]	Zambia	Kawambwa District	Jul	Lunda	Leopard, lion	Calendrical	L	Annual event celebrating the Lunda and Luba migration from DRC to Zambia. In one video, there is a leopard skin on a chair, and the King appears to have a lion mane armband.
**Nc'wala** ⌘✪[2]	Zambia	Chipata District	Feb	Ngoni	Leopard, lion, serval, genet	Calendrical	H	Annual union of the Ngoni people from Zambia, Malawi, Tanzania to give thanks to the first harvests of the season. Hundreds of tribesmen wearing leopard skins and other spotted carnivores; likely hundreds of genet skins. Evidence of paramount chief wearing a lion cloak. A full range of garment types (skirts, capes, headbands, armbands, etc). Panthera has conducted further research on this event. Some leopard and serval, compared to hundreds of genet skins).
**Ngoni dance**	Zambia	Various		Ngoni	Serval, genet	NC dance	L	A dance by Ngoni men wearing a serval skin head band and a genet skin.
**Ntongo**	Zambia	Mufumbwe District	Jun	Kaonde	Other	Calendrical	L	Annual first fruits festival; occasional person in the audience with a felid skin hat; sporadic.

[3] Makishi dances: On UNESCO's List of Intangible Cultural Heritage https://ich.unesco.org/en/RL/makishi-masquerade-00140, which is a list of cultural practices and traditions aimed at recognising, preserving, and promoting the cultural heritage of different communities. (https://ich.unesco.org/en/lists)

**Shimunenga** ✪[5]	Zambia	Namwala District	Oct	Ila	Leopard, serval, other	Calendrical	**M**	Annual festival. Notable numbers of dancers wearing these skins around their waist, including leopard. Possibly a small festival that could be investigated further.
**Ukusefya pa Ng'wena** ⌘✪[7]	Zambia	Mungwi District	Aug	Bemba	Leopard, lion, serval, other	Calendrical	**L**	Annual festival of the crocodile. One year they requested two lions and leopards from "government" to use at the ceremony in 2004. Sporadic evidence of focal species. Some dancers appear to wear leopard and serval skins.
**Ukwilimuna**	Zambia	Mpongwe District	Jul	Lamba	Other	Calendrical	**L**	Annual celebration of harvest and cultural history; skin use sporadic and not common.
**Zengani**	Zambia	Lundazi District	Oct	Tumbuka & Ngoni	Serval, genet, other	Calendrical	**M**	Annual event bringing together Tumbuka and Ngoni tribes. Difficult to estimate how many wearing these skins. It depends on the dance troupe regalia. One video shows a group of dancers wearing spotted carnivore skins in different forms (especially genets). Some regalia consist of tails worn around the waist. Evidence of some serval.
**Traditional dance**	Zimbabwe	Not specified	All year	Ndebele	Leopard	NC event	**L**	Gathering of <10 male dancers, and a few have leopard skin in their dress**.**
**Traditional dance**	Zimbabwe	Greater Zimbabwe ruins		Shona	Leopard, lion, genet	NC event	**L**	Troupe of female dancers, including one appearing to wear leopard, lion and genet skin**.**
**Traditional dance**	Zimbabwe	Not specified			Leopard, genet		**L**	Person wearing leopard and genet skin
**Umdlalo wetonga**	Zimbabwe	Not specified	All year	Ndebele	Leopard, serval, cheetah	Contingent	**H**	Stick fighting event. Gathering of >50 men wearing traditional regalia – most including leopard, serval and cheetah**.**

S7 Table: The number of categorised cultural event types, where eight focal taxa were observed worn in traditional attire, in 23 countries, verified through the review of 555 YouTube videos. 'Other*' are spotted carnivore taxa (including, tentative evidence for polecat and honey badger) that were not identified due to poor video resolution. 'NC' (Not Classified) are events or dance types that couldn't be assigned to specific categories. Data is summarised from S6 Table, and event classifications are defined in the methods and S6 Table. The classification of cultural events is adapted from Mkandawire et al (2019).

Cultural event classification	Leopard	Lion	Cheetah	Serval	Wildcats	Civets	Genets	Linsangs	Other*	Total events
**Calendrical**	27	15	3	21		2	13		10	34
**NC event**	16	1		10		5	16	1	4	26
**NC dance type**	1	1		3		2	6			5
**Contingent**	4	1		2		1	2			4
**Initiation (calendrical)**	3	2		1	2		2			3
**Initiation (other)**	2		1	1		1	1			2
**Private/secret**				1			1			2
**Affliction**	1	1					1	1		1
**Divination**	1			1			1		1	1
Total	*55*	*21*	*4*	*40*	*2*	*11*	*43*	*2*	*15*	*78*
